# Cultural similarity and impartiality on voting bias: The case of FIFA’s World’s Best Male Football Player Award

**DOI:** 10.1371/journal.pone.0270546

**Published:** 2022-07-13

**Authors:** Michael R. Johnson, Ian P. McCarthy

**Affiliations:** 1 Beedie School of Business, Simon Fraser University, Vancouver, B.C., Canada; 2 Luiss, Rome, Italy; University of Innsbruck, AUSTRIA

## Abstract

Previous studies on voting bias in competitive awards have not fully considered the role of cultural similarity. Using data for the Best FIFA Men’s Player Award, we evaluate the extent of voting bias in this Award using three cultural similarity factors (cultural distance, cultural clusters, and collectivism), six established in-group factors (nationality, club, league, geography, ethnicity, religion, and language) and the impartiality of the voter’s country. Using statistical and econometric methods, we find that voter-player cultural similarity is positively associated with voting bias and find no evidence of impartiality when it comes to cultural or national ties. We also find that media voters are less biased than captain voters and coach voters, and that coaches are less biased than captains.

## 1. Introduction

Awards play an important role in many industries, especially sports and entertainment. Some of these awards use voting to determine which candidate should win the award. For example, the National Basketball Association (NBA) has a panel of sportswriters and broadcasters who vote for the Most Valuable Player Award, the Eurovision Song Contest has competing countries cast votes for the other countries’ songs, and members of the Academy of Motion Picture Arts and Sciences nominate and vote for Oscar winners. Given the importance of such awards, researchers have examined how voters can be unfairly in favor or against a candidate [1, 2].

From this prior research, there is ample evidence that voters are guided not only by the quality of the candidates but also by established in-group biases [3–5]. This is a bias where candidates favor someone from their group, where the group can be a family, an organization, a sports team, a political party, a gender, a race, a religion, or a nation. Even though multiple in-group factors can affect voting bias, prior studies do not carefully examine the affect of cultural similarities, instead focusing only on established in-group factors. For example, established factors such as gender bias in politics [6], shared nationality in ski jumping [7, 8]; and friendship networks in the Eurovision Song Contests [9]. Cultural similarity differs from shared nationality and other established in-group factors. Cultural similarity is a set of values shared in a group that arises from the collective programming of the minds in that group [10]. Thus, there is an opportunity to examine voting bias in terms of both established in-group factors and cultural factors.

We investigate voting bias in the Best Fédération Internationale de Football Association (FIFA) Men’s Player award (from now on called, the Award) using established in-group and cultural factors. We use seven years (2010–2016) of data for the Award. Officially, the Award is determined by “the best in their category, without distinction of championship or nationality for their achievements during the year” [11]. Thus voters are reminded to vote “according to on-field performance and overall behavior on and off the pitch” [11]. However, there are concerns about the extent to which voters prejudicially award points to players (i.e., the Award candidates) based on in-group factors such as being on the same club team or sharing the same nationality [12, 13]. As one media outlet wrote, “the levels of bias and favoritism in the voting of the Ballon d’Or make the Eurovision Song Contest look positively objective” [14].

In addition to concerns about favoritism, the Award is useful for studying voting bias for three reasons. First, we can examine how multiple in-group factors, including geographical, nationality, ethnicity, language, religion, voter type, club, league, and variables for player performance, interact to influence voter bias over time. Second, the Award is global and offers extensive information about the voters, the candidates, and how votes were cast. Each year there are three types of voters (captains of national teams, coaches of national teams, and a media person from each nation) from approximately 200 countries. Each voter has three votes to assign to a shortlist of 23 player candidates (referred to as ‘players’), awarding 5 points, 3 points, and 1 point to their selected players. The single player that receives the highest cumulative total number of points wins the Award. This international context, and the accompanying substantial cultural differences, are useful for examining the role of cultural similarity between voter and player on voting bias. We do this by unpacking cultural similarity into three factors: cultural distance, cultural clusters, and voter collectivism. We use a well-established methodology for measuring voting bias that calculates the difference between the number of points awarded to a player by a given voter and the average number of points awarded by all other voters to said player. Lastly, the Award provides a practical context for investigating how a voter’s country’s impartiality might affect voting bias.

Our results show that voting bias exists in the Award. We find that cultural distance, cultural clusters, collectivism, and geographical distance all impact voting bias. We also find that when voters and players share nationality, club, league, or are country neighbors, then voting bias is positively correlated. In terms of the bias exhibited by different voters, media voters have a significantly lower voting bias than captain voters and coach voters, and coaches have a lower voting bias than captains. Overall, our findings show that something other than player quality (i.e., relative wins, goals, and assists) can be attributed to the voting results. This analysis provides new insights into how cultural similarity and impartiality influence bias in peer voting and international award competitions.

## 2. Related literature and hypotheses development

In the context of sports, prior research has examined voting bias in the seeding of basketball teams [15, 16] and judging in sports including boxing [17, 18], figure skating [14, 19, 20], gymnastics [21, 22], and ski jumping [20]. For music awards, research has found strong evidence of cultural, political, and geographical bias in the Eurovision Song Contest, a song writing and performing award organized by the European Broadcasting Union [2, 3, 9, 23, 24]. For example, Ginsburgh and Noury [23] show that votes are not only based on the quality of the performances but also on the linguistic, political, and cultural proximities between voting countries and singers. This finding is consistent with work by Lee and colleagues [25], who show that voting is typically a deliberate and rational act carried out to attain specific goals influenced by cultural identity.

A few studies have examined voting bias in the Award (under its current or former names) which is the focus of our study. Focusing on the role of cultural diversity, Ruizendaal [26] found no evidence of the impact of this factor on voting bias. One reason for this could be that the study was limited to a single year of data and considered only 900 votes (55%) out of the possible 1623 total votes in 2013. However, this study did find that a shared nationality between voter and player was a significant factor in voting bias in the 2013 Award. Konijnenburg [27] investigated three years of the Award, finding further evidence of nationalistic voting, and concluded that other cultural and geographic factors could further explain such biases. Then Coupe and colleagues [4], using a five-year data set, found that voters are four times more likely to vote for candidates with whom they share the national team or the same league team and three times more likely to vote for a candidate with whom they share the same nationality.

Voting bias can be explained using social identity theories [28, 29] and homophily [30], where people classify themselves and others using in-group factors such as religious affiliation, organizational membership, gender, nationality, culture, and age. These in-group factors underlie what psychologists call ‘ingroup-outgroup bias’ [5, 31], where members in a group will discriminate against those outside the group to enhance a more positive identity for those inside the group. Such factors impact voting bias via ‘in-group love’, where members of a group are motivated to serve, help and maintain the in-group, and by ‘out-group hate’, where members not in the group can be the target of unfair or competitive actions [32].

Prior research on awards has found that the basis for voter-player similarity includes factors such as gender [33, 34], race [35, 36], nationality [3, 4], and political and cultural connections [23, 37]. Social identity theory asserts that groups of people will coalesce around a cause or subject with which they can identify and draw a group identity, which leaves those who are not part of that group outside the group [29]. Therefore, for the Award, the greater the sense of in-group identity a voter experiences with a player–through physical, cultural, geographical, and other factors–the greater the degree to which biased voting occurs. Consequently, based on this literature, we propose our first hypothesis:

***Hypothesis 1*:** *Voters are more likely to choose players with similar in-group factors (i*.*e*., *nationality*, *club*, *league*, *geography*, *ethnicity*, *language*, *and religion)*.

Given that cultural similarity is a recognized determinant in social identity theory for explaining intergroup behavior such as bias, we now develop a hypothesis for how three characteristics of cultural similarity (cultural distance, cultural clusters, and collectivism) impact voting bias in the Award. Our fundamental premise is that cultural distance, and cultural clusters are an important element of voters’ calculations (conscious or unconscious) of their similarity to the players, which influences the likelihood of voting bias. Furthermore, voters from countries with strong collectivist cultures will emphasize the group rather than the individual and will more likely favor those within or close to their cultural group.

Hofstede’s [10, 38] research on cultural distance is widely used in business and economic research to measure how cultures are similar or different. He identified six values that constitute cultural distance: power distance, individualism-collectivism, uncertainty avoidance, masculinity-femininity, long-term orientation, and indulgence [38]. Together, these six dimensions of cultural distance create (or reduce) distance between a voter and a player because they reflect differences (or similarities) in language, ethnicity, religion, and social network and norms [39]. In essence, cultural distance represents a priori knowledge, and in the context of international competitions, voters consciously or unconsciously incorporate a “calculation” of cultural similarity with players that translates into voting bias. Consequently, we suggest that the international character of the Award and, thus, the heterogeneity and cultural proximity of participating countries play an important role in motivating biases. Such biases form from voters recognizing players (consciously or unconsciously) as being culturally less ‘different’; thus, voters are more willing to vote for them.

Our second factor used to characterize cultural similarity between voters and players is cultural clusters. GLOBE researchers [45] used data from 62 countries to identify ten distinct clusters (i.e., regions) that exhibit similarities and differences between cultural groups [40, 41], as identified in S1 Appendix. Mensah and Chen [40] extended this classification of the GLOBE study using 194 countries based on factors including race, religion, language, geographic proximities, and colonial heritage. We anticipate that voters and players from the same cultural cluster will have a greater voting bias than voters and players from different cultural clusters. In S1 Appendix we assigned the following countries (that have participated in the FIFA’s World’s Best Male Football Player Award) to the United Kingdom: England, Wales, Ireland and Scotland.

Our third factor for characterizing cultural similarity is collectivism. Hofstede (43, p. 348) defines collectivism as “a preference for a tightly-knit social framework in which individuals can expect their relatives, clan, or other in-group to look after them, in exchange for unquestioning loyalty.” In contrast, individualism is “a preference for a loosely knit framework in society in which individuals are supposed to take care of themselves and their immediate families only” (43, p.348). Consequently, collectivist cultures are more likely to exhibit loyalty and support to those in their in-group [39, 42, 43]. In such cultures, there is a strong feeling of involvement in each other’s lives and a strong feeling of loyalty and responsibility to the group. In this regard, individualism is supportive of the individual (or “I”), and collectivism finds importance in the group identity (or “we”) in their decision-making.

The GLOBE data set divides collectivism into two dimensions: collectivism I (institutional collectivism) and collectivism II (in-group collectivism). The first form of collectivism is the extent to which society encourages individuals to be integrated into groups; the second is the degree to which individuals express loyalties to their families or in-groups [44, 45]. We hypothesize that a voter from a country that is more collective (i.e., a lower degree of individualism) would more likely prejudicially allocate points to players with similar cultural backgrounds. Based on this discussion, we put forward our second hypothesis:

***Hypothesis 2***
  - *2A*: *An increase in cultural distance between a voter and a player will decrease voting bias*.
  - *2B*: *Voters and players that reside within the same cultural cluster will have higher voting bias relative to those that do not live within the same cultural clusters*.
  - *2C*: *Voters from collectivist cultures will have a greater voting bias than those from individualistic cultures*.

Our final hypothesis explores how the impartiality of a voter’s country impacts voting bias within the cultural clusters defined in hypothesis 2B. An impartial government (e.g., a high quality government) will treat individuals within its country in an impartial manner regardless of race, ethnicity, family/political ties, or social standing [46]. Impartial institutions generally believe that decisions should be based on quality rather than personal connections. In other words, the quality of government represents societal values, which condition individual decision-making and voting behavior in aggregate [9, 47–49]. As Charron [9] states, ‘political institutions reflect a certain culture of citizens that they represent, and the relationship is reciprocal; political institutions impact aggregate individual behavior’. For the Award, this means that voters from countries with quality governments (more impartial institutions) would be more likely to vote based on quality over favoritism, even if it is outside their cultural group, compared to voters from more partial countries. Hence, our final hypothesis is:

***Hypothesis 3*:** *The impartiality of a voter’s country reduces cultural voting bias*.

**Fig 1:**
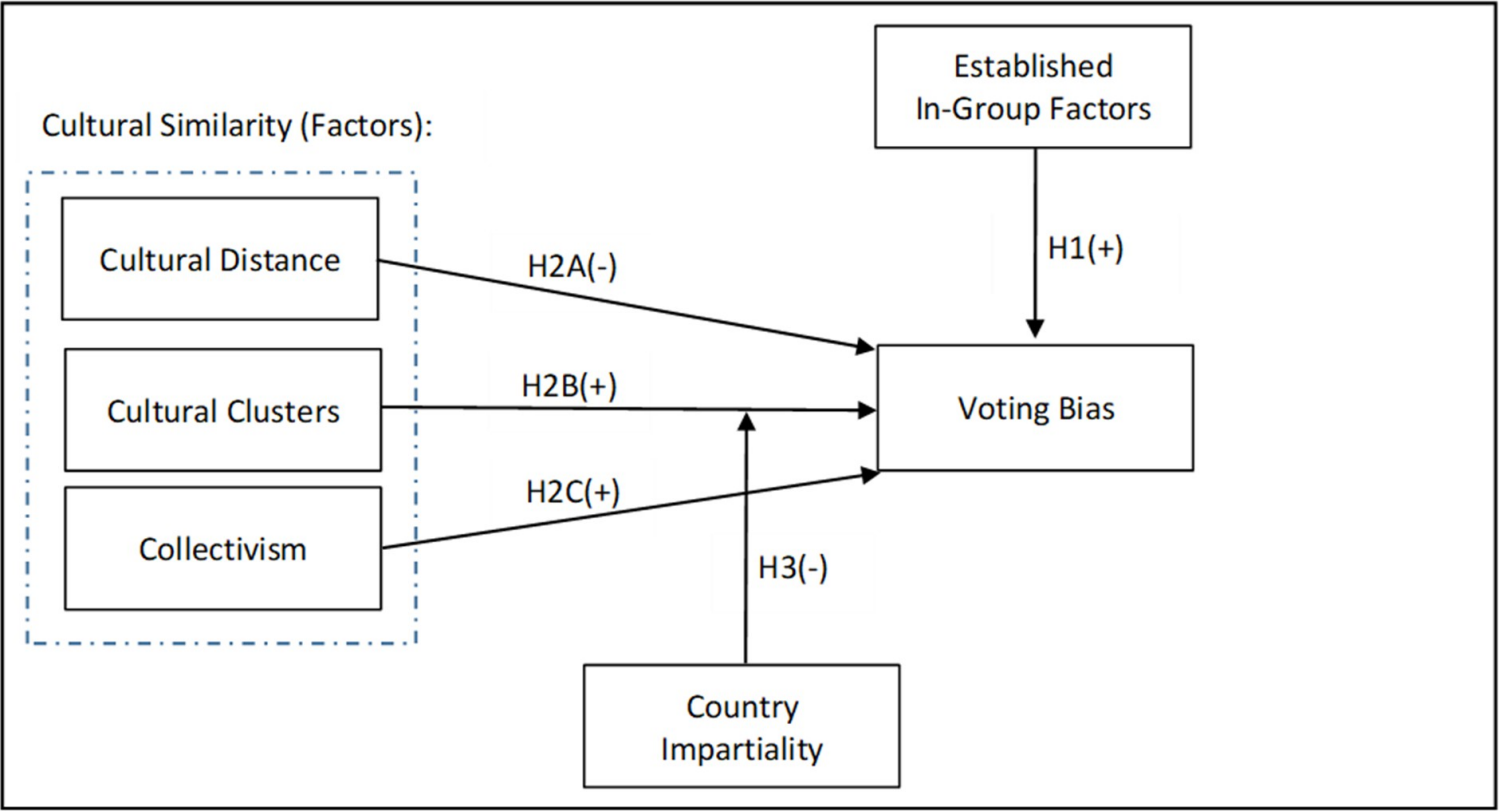
Research design and hypotheses.

Fig 1 illustrates the hypotheses in our study.

## 3. Data and model

We use data provided by FIFA to investigate voting bias in the 2010 through 2016 Award years. The dataset consists of 10,305 votes from 3,435 voters spanning seven years. As shown in Table 1, for each year, every participating country has a captain, a coach, and a journalist provide votes awarding 5 points, 3 points, and 1 point to any three of the 20-some shortlisted players to determine the best football player of the year.

**Table 1:** FIFA dataset used to investigate voting bias.

	2010	2011	2012	2013	2014	2015	2016	Total
Number of Shortlisted Players	23	23	23	23	23	23	22	67
Number of Voting Countries	187	191	197	207	203	197	187	
Voters								
Captains*	136	153	170	184	182	162	159	
Coaches	136	156	170	184	181	165	163	
Media	154	156	169	173	181	171	130	
Total	426	465	509	541	544	498	452	3,435
*Number of shortlisted Captains	4	3	4	6	5	7	7	
Total Votes	1,278	1,395	1,527	1,623	1,632	1,494	1,356	10,305

We also incorporate the GLOBE data set [50] on cultural dimensions to contrast our empirical findings with Hofstede’s dimensions [51]. The number of countries available in the GLOBE data set is somewhat smaller than Hofstede (58 versus 165 respectively); however, the sample size of 3,306 votes (1102 voters x 3 votes each) across the seven years still provides a useful comparison and validation for our analysis.

### 3.1 Dependent variable: Defining a measure of voting bias

We draw upon research on voting bias in the Eurovision Song Contest [1, 3, 9] and research on the Award [26] to calculate voting bias. For our study, voting bias is not simply the prejudiced awarding of a high number of points from a voter to a player; it is the number of points awarded to a player compared to the average number of points awarded by all other voters. In this regard, voting bias is relative and is defined as follows:

$$\mathrm{Voting}\;\mathrm{bias}\;(b_{jk})=\mathrm{Points}\;(p_{jk})-{\bar{q}}_{k,-j}$$ Eq (1)

where *b*_*jk*_ represents the voting bias between voter *j* (captain, coach, or media) and player *k*; *p*_*jk*_ is the number of points awarded by voter *j* to player *k* (and will take on the form of 5, 3, or 1 points awarded); and, ${\bar{q}}_{k,-j}$ represents the average number of points awarded from *all other voters* to a player *k* (i.e., excluding voter *j*’s points). The average number of points awarded by all other voters is defined as:

$${\bar{q}}_{k,-j}=\frac{1}{n_{jk}}\sum_{i\neq j}p_{ik}$$ Eq (2)

where *n*_*jk*_ is the total number of voters awarding points to player *k* (less the one vote from voter *j*). Note that for every vote and the corresponding points awarded, we calculate the average points awarded from all other voters, excluding the points given from voter *j* to player *k*. This specification of the dependent variable has the advantage of being a continuous variable that includes a proxy of player quality (i.e., Eq 2) thereby avoiding problems of endogeneity and simultaneity [3, 9]. A large voting bias is an overvaluation of a player *relative to all other voters*, and a low voting bias is an undervaluation of a player *relative to all other voters*.

### 3.2 Explanatory variables: Potential determinants of voting bias

To investigate our first hypothesis, we collected data on voter and player characteristics related to the six established in-group factors (nationality, club, league, geography, ethnicity, religion, and language) that could be determinants of voting bias. Data for nationality, league, and the football club for players and captains was collected using https://www.footballdatabase.eu/. For geography, we captured data on the physical distance between the capital city of the voter’s country and the capital city of the player’s country [52]. We use a binary independent variable to designate if a voter and a player live in neighboring countries and if a voter and a player share the same nationality. Using economic data from Alesina and colleagues [53], we test the relationship between the extent of ethnicity, religion, and language fractionalizations within a voter’s country and the degree of voting bias. This relationship is in the form of country indices (ranging from 0 to 1), representing the degree to which a country is fractionalized relative to these three variables. We anticipate that as a country becomes more fractionalized (or diversified), it would lead to a lower voting bias. More fractionalization reflects a lower probability that two randomly selected people from a given country will not share such characteristics (ethnicity, religion, and language backgrounds) [54].

#### 3.2.1 Cultural similarity

For our second hypothesis, we use the three factors related to cultural similarity: cultural distance, collectivism, and cultural clusters. For cultural distance, we use Hofstede’s six cultural dimensions and the nine cultural dimensions of GLOBE. Hofstede’s dimensions (power distance, individualism, masculinity, uncertainty avoidance, long-term orientation, and indulgence) are on a scale of 0 to 100. In contrast, the GLOBE dimensions (performance orientation, uncertainty avoidance, humane orientation, institutional collectivism, in-group collectivism, assertiveness, gender egalitarianism, future orientation, power distance) are on a scale of 1 to 7. We use both sets of cultural dimensions in different ways to investigate cultural variation and its impact on voting bias. The first technique involves calculating Euclidean distance to represent the cultural distance between voters and players. This calculation requires several steps. First, each player and voter are assigned a value for each of the six Hofstede dimensions (*i*) according to their respective nationality. Next, we determine the squared Euclidean distance between each voter (*j*) and each selected player (*k*) based on Hofstede’s values (*i*) of the six dimensions and divide by the number of distance relationships (*n* x (*n*– 1)) multiplied by the number of cultural dimensions (e.g., 6). Finally, we take the square root of the term as shown in Eq 3. Eq 3 calculates the Euclidean distance between a single voter and their selected player using the six dimensions to generate an aggregate index of cultural distance between a voter and a selected player.


$$Cultural\;Distance({CD}_{jk})=\sqrt{\frac{\sum_{i=1}^{6}{\left(I_{i,j}-I_{i,k}\right)}^{2}}{n(n-1)6}}$$ Eq (3)

The calculation of cultural distance using the GLOBE cultural dimensions is the same, except there are nine instead of six dimensions. The technique (Eq 4) is used to investigate the individual proximities between a voter’s index and a player’s index based on each of the six (or nine in GLOBE) cultural dimensions:

$${Dimension}_{i}={|I}_{j}-I_{k}|$$ Eq (4)

Where *i* represents one of the six dimensions of Hofstede’s cultural diversity indices (Power, Individualism, etc.), *I*_*j*_ represents the respective index of the voter, and *I*_*k*_ represents the respective index of the player. Lastly, Eq 5 represents a voter’s collectivism measured by subtracting a voter’s individualism (Hofstede) cultural dimension from 100.


$${Voter\;Collectivism}_{i}=100-I_{j}$$ Eq (5)

Voter collectivism is a measure ranging from a value of 0 (representing maximum individualism) to a value of 100 for maximum collectivism. As discussed, we hypothesize that a voter from a more collective society (i.e., a lower degree of individualism) would have greater voting bias than a voter with lower collectivism. Likewise, we investigate the relationship between two cultural dimensions of the GLOBE study associated with collectivism (in-group and institutional collectivism) and voting bias.

Our final measure of cultural similarity utilizes cultural clusters defined in research through the GLOBE study [40, 45]. As previously discussed, GLOBE researchers divided data from 62 countries, which was later extended to 194 countries by Mensah and colleagues [40], into ten distinct ‘cultural societies’ to allow for analysis of similarities and differences between cultural groups. Using categorical variables, we represent the ten distinct cultural societies (shown in S1 Appendix) to investigate if voting bias is positive and significant across clusters. An indicator variable (*CC*_*jk*_) is used to identify voters and players from countries that are defined within the same cultural cluster or not. This indicator reflects voting bias between voters and players who share the same cultural cluster.

#### 3.2.2 Impartiality–Quality of government

For our third hypothesis, we measure impartiality using ‘quality of government’ data from the International Country Risk Guide (ICRG) [53]. This measure combines three characteristics of public sector practices: the extent to which corruption is prevalent, the strength of the ‘rule of law’, and the level of governmental effectiveness. The mean value of the ICRG variables is scaled from zero to one, and higher values indicate a higher quality of government. This variable is a proxy for impartiality or ‘quality of government’ [55].

We use an interaction term of impartiality with cultural clusters to test our final hypothesis (similar to Charron [9]). If cultural voting bias is present, cultural clusters (*CC*_*jk*_) is expected to be positive and significant, implying that positive voting bias exists between voters and players who share the same cultural cluster. According to our hypothesis and based on similar research conducted by Charron [9], the interaction term (cultural clusters *CC*_*jk*_ x impartiality *Im*_*j*_) should be negative and significant, implying that voting bias across countries defined by their cultural clusters diminishes significantly as *impartiality* (*Im*_*j*_) increases. This finding is consistent with similar results of Charron [9] that demonstrated that despite showing overwhelming evidence of voting bias in the Eurovision Song Contest within friendship groups, voting bias is considerably less among friendship groups from countries with impartial governments compared to friendship groups from countries with highly partial government institutions.

#### 3.2.3 Performance variables

We collected six performance variables for this study, three of which were found to be valuable in this study. The three variables used are: the percentage of games won, the goals per game, and the assists per game. These data were collected from websites specializing in providing football statistics: footballdatabase.eu and squawka.com. Each variable is a performance metric based on a player’s performance in all games (league, continental cups, national cups, and international matches). Table 2 lists the established in-group and cultural factors used in our study. It should be noted that the player performance metrics for *poss*_*kt*_, *defence*_*kt*_, *and attack*_*kt*_ come from Squawka (http://www.squawka.com) and are used as additional performance metrics as only three years of performance data is available. The Squawka Player Performance Score uses data from the Premier League’s official data provider Opta and uses an algorithm to rate players based on real-time play components; attack, defence and possession. The attack score takes all the attacking events into consideration (shots, crosses, take-ons, etc.), the defence scores defensive actions (e.g tackles, interceptions, etc.) and the possession score analyzes passes, through-balls, etc. The algorithm scores each event independently.

**Table 2:** In-group and cultural factors.

*Club*_*jk*_	One if voter and player are playing on the same football club at the time of the voting, zero otherwise.
*League*_*jk*_	One if voter and player are playing on the same football league at the time of the voting, zero otherwise.
*Coach*_*j*_	One if voter *j* is voting as a coach, zero otherwise. Reference category is captains.
*Media*_*j*_	One if voter *j* is voting as a media, zero otherwise. Reference category is captains.
*Nat*_*jk*_	One if voter and player share the same nationality, zero otherwise.
*Dist*_*jk*_	The physical distance (in kilometers) between the capital city of the voter’s country and the capital city of the player’s country.
*Nbr*_*jk*_	One if voter and player live in neighbouring countries, zero otherwise.
*Im*_*j*_	The country index for the quality of government (impartiality) of voter *j*.
*Ethnic*_*j*_	The country index for ethnicity fractionalization of voter *j*.
*Language*_*j*_	The country index for language fractionalization of voter *j*.
*Religion*_*j*_	The country index for religion fractionalization of voter *j*.
**Cultural Factors**	
*CD*_*jk*_	Proximity between voter *j* and player *k* in terms of cultural distance (Eq 3)
*CC*_*jk*_	One if voter and player share the same cultural cluster, zero otherwise.
*VC*_*j*_	Voter’s cultural index of collectivism (Eq 5).
*pdi*_*jk*_	Proximity between power distance indices of voter *j* and player *k* (Eq 4).
*idv*_*jk*_	Proximity between individualism indices of voter *j* and player *k* (Eq 4).
*mas*_*jk*_	Proximity between masculinity indices of voter *j* and player *k* (Eq 4).
*uai*_*jk*_	Proximity between uncertainty avoidance indices of voter *j* and player *k* (Eq 4).
*lvs*_*jk*_	Proximity between long term versus short term orientation indices of voter *j* and player *k* (Eq 4).
*ivr*_*jk*_	Proximity between indulgence versus restraint indices of voter *j* and player *k* (Eq 4).
**Players’ Performance Metrics**	
*wpcent*_*kt*_	The percentage of games won for player *k* in year *t*.
*gpg*_*kt*_	The goals per game for player *k* in year *t*.
*apg*_*kt*_	The assists per game for player *k* in year *t*.
*poss*_*kt*_	The ball possession score for player *k* in year *t*.
*defence*_*kt*_	The defence score for player *k* in year *t*.
*attack*_*kt*_	The attack score for player *k* in year *t*.

### 3.3 Statistical models

We test our three hypotheses using econometric methods. The first method employs a fixed-effects regression model to predict the percentage of points earned for each shortlisted player over the 7-year time frame using only the performance characteristics collected for this study. In this way, we control for the individual heterogeneity of the players and test if individual heterogeneity (or unobserved factors not related to performance) is a significant factor in the competition. This method provides a statistical model to test our first hypothesis of whether voting bias exists. Two general assumptions justify using the fixed-effect model to investigate the presence of voting bias. The first assumption is that the performance variables (percentage of games won, goals per game, and assists per game) can sufficiently capture a player’s performance. Second, the percentage of votes that a nominee receives each year is determined largely by the performance of that nominee in that calendar year. Therefore, any unobserved effects (i.e., individual player heterogeneity) influence voting results that are not related to performance. In other words, if individual heterogeneity is statistically significant, something other than performance contributes to the percentage of points received, thus allowing us to infer the possibility of voting bias. The player fixed-effect model can be represented by:

$${\mathrm{Percentage}\;\mathrm{of}\;\mathrm{Player}\;\mathrm{Votes}\;\mathrm{received}}_{kt}=\beta_{1}{\mathrm{wpcent}}_{kt}+\beta_{2}gpg_{kt}+\beta_{3}apg_{kt}+a_{k}+u_{kt}$$ Eq (6)

where the dependent variable is the percentage of votes player *k* received in year *t*; “*wpcent*_*kt*_”, “*gpg*_*kt*_” and “*apg*_*kt*_” are performance variables (percentage of games won, number of goals scored per game and number of assists per game, respectively); and “*a*_*k*_” is the “player fixed effect” which is a constant representing the unobserved effect on the percentage of votes received by player *k* due to “being” player *k*. In this way, we interpret the unobserved effect as the potential for voting bias associated with individual players.

The second method uses ordinary least squares (OLS) to estimate parameter estimates with the dependent variable represented as the bias calculation, as shown by Eqs 1 and 2 in section 3. This method addresses the remaining hypotheses and provides an overall model to understand better how explanatory variables impact voting bias. The general model specification is defined as:

$$Voting\;Bias_{jkt}=\alpha_{j}+X_{jkt}\beta_{j}+\varepsilon_{jkt}$$ Eq (7)

where the dependent variable is defined by Eqs 1 and 2, representing voting bias between voter *j* and player *k* in year *t* and *X*_*jkt*_ represents various explanatory and control variables. The benefit of this specification is that the dependent variable is continuous (rather than categorical), allowing us to investigate our hypotheses and easier interpret the marginal effects of the explanatory variables. Other studies used nonlinear regression methods to investigate voting bias in this competition making the interpretation of marginal effects challenging (see [4] for a good discussion on this topic). The key assumption and perhaps limitation of our specification is that the explanatory variables impact voting bias of all the voters in a similar way across voters. The coefficients of the covariates thus reflect the average influence of the factor on voting bias in the awards competition. Estimations were run independently based on the number of points awarded (5 points, 3 points, or 1 point) due to voting bias having significantly different distributions based on the number of points awarded. This study used different control variables to explore the relationship between voting bias and the potential in-group factors. We control for voter type (captain, coaches, and media), the year of the competition, and player performance (goals and assists per game).

## 4. Empirical analysis

### 4.1 Descriptive statistics

We now briefly discuss the bivariate correlations between some variables to investigate the relationships between voting bias and established in-group factors. We then present the results for our three hypotheses.

Table 1 provides an overview of the data used in this study. Descriptive statistics and bivariate correlations of the independent variables with voting bias are quite consistent using both the Hofstede and GLOBE datasets for cultural dimensions. Both cultural distance (*CD*_*jk*_) and geographical distance are negatively correlated with voting bias and statistically significant across all points values, separately. This finding indicates that voting bias decreases on average as the cultural or geographical distances between a voter and a player increases. Likewise, nationality, club, league, and being from a neighboring country are positively correlated and statistically significant with voting bias. This result indicates that increasing similarity between these characteristics increases voting bias on average. These relationships become even more pronounced when correlations are calculated based on individual points awarded (5, 3, and 1 points), providing further evidence to warrant the specification of regression models using individual points awarded.

### 4.2 Hypothesis 1 (Voting Bias?)

We use a player-specific fixed-effect regression model to investigate the presence of voting bias. Two panels were constructed for investigating the player fixed-effect model: one is constructed by players with at least three nominations from 2010 to 2016 (22 players with a total of 95 votes); one is constructed by players with at least four nominations during the same period (15 players with a total of 74 votes). The two panels show similar results. The adjusted R-square is approximately 75% for all ten models (five for each panel), indicating that performance and players’ heterogeneity explain a high proportion of the variability in the votes received. The statistical value of rho shows that variances due to individual heterogeneity are approximately 60% to 70%. This finding shows that something other than wins, goals, and assists can be attributed to the voting results.

The premise that there is no individual player-specific heterogeneity is firmly rejected in all models (*P* < 0.0001), further providing statistical evidence of the potential for voting bias in the selection of players (and support for our first hypothesis). The analysis also shows that goals per game is the most significant of the three-performance factors utilized. Goals per game is positive (estimated coefficients of 8.69 and 9.82, respectively) and statistically significant (*P* < 0.10) across both panels. Assists per game has a positive sign which corresponds to our hypothesis but is not statistically significant (0.15 < *P* < 0.10). Interestingly, the coefficient for the percentage of games won indicates that a higher percentage of games won leads to a lower percentage of the total votes awarded in any given year. The negative sign is not consistent with the expectation that a higher percentage of games won would lead to a higher percentage of votes received. An explanation is that this factor is not closely related enough to an individual players’ performance, such as goals and assists, since the percentage of games won is the entire team’s performance. For this reason, we do not use this performance measure in other estimations.

Given this evidence, we also investigate factors that are potential determinants of voting bias. We use estimate regression models using OLS with the dependent and independent variables represented as shown in Section 3. As mentioned, voting bias is highly dependent on the number of points awarded (5, 3, or 1 point) due to the nature of the calculation of voting bias used within this study. Therefore, as shown, we have selected to control for the number of points awarded by running regression models for 5, 3, and 1 points awarded separately. Results from the first set of regression models for voting bias are presented in Table 3. This table demonstrates the relationship between several potential determinant factors and voting bias for the case of 5 points awarded. The regression models for voting bias with 3 and 1 points awarded follow a similar pattern to the models explained here. For all regression models, we check for the presence of multicollinearity in our data. Variance inflation factors (VIF) are smaller than 2, thus showing no concerns regarding multicollinearity. Specification tests demonstrate no omitted variables, yet heteroskedastic error terms are present. Therefore all OLS estimations are performed using Huber White robust standard errors.

**Table 3:** Hypothesis 1 (Voting Bias?): Regression models on voting bias for 5 points awarded using both Hofstede and GLOBE data sets.

	Models 1–6: Hofstede’s Data						Models 7–12: GLOBE Data					
	Model 1	Model 2	Model 3	Model 4^**λ**^	Model 5	Model 6	Model 7	Model 8	Model 9	Model 10^**λ**^	Model 11	Model 12
Media	-0.320***	-0.325***	-0.361***	-0.316***	-0.339***	-0.312***	-0.452***	-0.428***	-0.447***	-0.429***	-0.425***	-0.382***
Coach	-0.055	-0.087*	-0.069	-0.065	-0.071	-0.028	-0.031	-0.088	-0.060	-0.033	-0.082	-0.061
2011	-2.180***	-2.240***	-2.094***	-2.230***	-2.069***	-0.590***	-2.018***	-2.057***	-1.848***	-2.052***	-1.890***	-0.386***
2012	-1.370***	-1.185***	-1.068***	-1.154***	-1.338***	-0.382***	-1.040***	-0.928***	-0.822***	-0.823***	-1.102***	-0.151
2013	-0.549***	-0.358***	-0.344***	-0.333***	-0.617***	0.162**	-0.480***	-0.370***	-0.358***	-0.302***	-0.631***	0.273**
2014	-0.881***	-0.879***	-0.886***	-0.883***	-1.006***	-0.172**	-1.009***	-1.047***	-1.058***	-0.986***	-1.132***	-0.168
2015	-1.549***	-1.441***	-1.291***	-1.410***	-1.464***	-0.623***	-1.588***	-1.507***	-1.287***	-1.456***	-1.483***	-0.525***
2016	-1.201***	-1.014***	-0.976***	-1.008***	-1.292***	-0.314***	-1.157***	-1.012***	-0.966***	-0.932***	-1.236***	-0.151
Nationality		1.242***			1.122***	0.965***		1.036***			0.863***	0.709***
Geographical Distance			-0.536***		-0.506***	-0.061*			-0.068***		-0.052***	-0.011*
Neighbouring Countries				0.225*	-0.028	0.075				0.327**	0.191	0.165
Ethnicity				-0.193**	-0.277**	0.063				-0.279*	-0.213	0.158
Language				-0.146*	0.154	-0.014				-0.263**	0.245	0.098
Religion				-0.144*	-0.054	-0.059				-0.369***	-0.227*	-0.241**
Goals/game						-1.992***						-2.086***
Assists/game						-1.821***						-1.911***
Constant	3.714***	4.250***	4.635***	4.321***	4.166***	4.985***	3.785***	4.301***	4.766***	4.453***	4.204***	5.032***
Observations	3103	3103	3103	2755	2656	2656	1099	1099	1079	1083	1063	1063
R^2^	0.257	0.314	0.324	0.290	0.335	0.656	0.225	0.294	0.322	0.261	0.318	0.693
Adjusted R^2^	0.255	0.312	0.322	0.288	0.332	0.654	0.220	0.288	0.317	0.254	0.308	0.688
F	208.1	216.8	264.5	183.8	149.0	470.9	71.80	76.51	92.41	70.54	55.60	230.4

***P <0.01

**P <0.05

* *P* <0.1 ^Ψ^ Using Eq 3. ^Ω^ Using Eq 4. ^**λ**^Ethnicity, Language and Religion variables run independently to avoid multicollinearity.

The regression models demonstrate consistent results using both Hofstede and GLOBE data sets. Model one and seven are considered the baseline models containing only the control variables. As shown, voter type and year both have significant impacts on voting bias. Interestingly, media voters have a significantly lower voting bias than captain voters (and coaches). Coaches also tend to have a lower voting bias than captains on average. However, their coefficients are not significant. Nationality is positive and statistically significant across all models indicating its significant role in voting bias in the competition. Interestingly, we also find that physical geographical characteristics are statistically significant with voting bias. As geographical distance decreases between a voter and player, voting bias is positive and significant across models using both Hofstede and GLOBE data sets (Hofstede: *P* < 0.001, *P* < 0.01 and *P* < 0.05 across points 5, 3 and 1 respectively; GLOBE: *P* < 0.001, *P* < 0.01 and *P* < 0.01 across points 5, 3 and 1 respectively). Likewise, if a voter and a player come from neighboring counties, voting bias is positive and significant across all points and data sets, but to a lesser extent for 1 point awarded. Table 3 also demonstrates the importance of the diversity of ethnicity, language, and religion on voting bias. These models demonstrate a negative and statistically significant relationship with voting bias, indicating that voters from countries with increased diversity among these variables tend to exhibit a lower voting bias on average.

Interestingly, not shown in Table 3 (due to space considerations), captains demonstrate a consistent pattern of voting bias with respect to teammates in their own clubs for 3 and 1 points awarded but not 5 points awarded. All coefficients are negative but only statistically significant at 3 and 1 points provided (*P* < 0.05 and *P* < 0.001 for points 3 and 1, respectively, for both data sets). This finding may indicate that captains are trying not to disclose blatant voting bias, given their close association with teammates. When voting for players within the same league, results demonstrate positive and statistically significant voting bias across all points awarded.

Based on these findings, we find sufficient evidence to support our first hypothesis that there exists voting bias in the Award, which can be attributed to the factors discussed.

### 4.3 Hypotheses 2 (Cultural Bias)

Regression models were developed for our second hypothesis regarding the affect of cultural similarity on voting bias in the Award. These models examine the relationships between the variables defined in section 3.2 for both Hofstede and GLOBE data sets. Table 4 provides the results of voting bias for 5 points awarded. Results are consistent for 3 and 1 points awarded across both data sets. Once again, specification tests demonstrate no concerns regarding multicollinearity, yet heteroskedastic error terms are present; therefore, all OLS regressions are performed using Huber White robust standard errors.

**Table 4:** Hypothesis 2 (Cultural): Regression models on voting bias for 5 points awarded using both Hofstede and GLOBE data sets.

	Models 1–5: Hofstede Data						Models 6–10: GLOBE Data				
Variables	Model 1	Model 2	Model 3	Model 4	Model 5	Variables	Model 6	Model 7	Model 8	Model 9^λ^	Model 10
Media	-0.318***	-0.313***	-0.309***	-0.312***	-0.721***		-0.452***	-0.379***	-0.363***	-0.429***	-0.452***
Coach	-0.042	-0.024	-0.026	-0.022	-0.122**		-0.031	-0.0372	-0.0385	-0.033	-0.0964
2011	-0.913***	-0.587***	-0.591***	-0.573***	-2.137***		-2.018***	-0.364***	-0.352***	-2.052***	-1.881***
2012	-0.428***	-0.376***	-0.372***	-0.354***	-1.028***		-1.040***	-0.113	-0.135	-0.823***	-0.932***
2013	0.273***	0.230***	0.221***	0.226***	-0.281***		-0.480***	0.364***	0.364***	-0.302***	-0.388***
2014	-0.293***	-0.104	-0.117*	-0.116*	-0.732***		-1.009***	-0.0901	-0.104	-0.986***	-0.864***
2015	-0.656***	-0.610***	-0.621***	-0.605***	-1.332***		-1.588***	-0.570***	-0.592***	-1.456***	-1.430***
2016	-0.273***	-0.241***	-0.252***	-0.233***	-0.865***		-1.157***	-0.21*	-0.212*	-0.932***	-1.112***
Cultural Distance (*CD*_*jk*_)		-1.123***				Cultural Distance (*CD*_*jk*_)		-0.472***			
Cultural clusters (*CC*_*jk*_)			0.358***			Cultural clusters (*CC*_*jk*_)			0.384***		
Voter Collectivism (*VC*_*j*_)				0.160**	0.202**	Voter Collectivism I (*VC*_*j*_)				0.106***	
						Voter Collectivism II (*VC*_*j*_)				0.207***	
Power Distance ^Ω^					-0.311*	Uncertainty Avoidance ^Ω^					0.164*
Individualism ^Ω^					0.651***	Future Orientation ^Ω^					0.175
Masculinity ^Ω^					0.488**	Power Distance ^Ω^					0.252
Uncertainty Avoidance ^Ω^					-0.676***	Institutional Collectivism ^Ω^					-0.601***
Long-term Orientation ^Ω^					-0.117	Humane Orientation ^Ω^					0.364**
Indulgence ^Ω^					-0.388**	Performance Orientation ^Ω^					-0.284**
						In-Group Collectivism ^Ω^					-0.351**
						Gender Egalitarianism ^Ω^					-0.493***
						Assertiveness ^Ω^					0.128*
Goals/game	-1.535***	-2.055***	-2.054***	-2.078***			-1.646***	-2.062***	-2.076***	-1.615***	
Assists/game	-2.502***	-1.940***	-1.867***	-1.851***			-2.748***	-2.287***	-2.360***	-2.870***	
Constant	5.531***	5.227***	4.968***	4.909***	4.293***		5.555***	5.215***	4.992***	4.474***	4.611***
Observations	3103	3103	3103	3103	3103		1062	1062	1062	1099	1062
R^2^	0.515	0.638	0.642	0.636	0.338		0.563	0.690	0.697	0.617	0.361
Adjusted R^2^	0.513	0.637	0.641	0.634	0.335		0.559	0.687	0.694	0.613	0.350
F	750.8	757.7	760.4	745.0	106.8		278.7	325.3	323.2	239.4	48.54

***P<0.01

**P <0.05

* *P* <0.1 ^Ψ^ Using Eq 3. ^Ω^ Using Eq 4. ^**λ**^Voter Collectivism I and II were run independently to avoid multicollinearity.

Model 1 and 6 establish a baseline with only control variables, while independent variables are integrated step-wise in the subsequent models. Models 2 to 5 and 7 to 10 test our second hypothesis using an independent variable for cultural distance, cultural clusters, and voter collectivism.

Our findings support the hypothesis that an increase in cultural distance leads to a decrease in voting bias. All coefficients for cultural distance (*CD*_*jk*_) are negative and statistically significant at the 1% level except for the case of 1 point awarded for both data sets (*P* = 0.08 and *P* = 0.03 for 1 point using Hofstede and GLOBE data sets, respectively). These results are consistent when controlling for players and voters with the same nationality. Likewise, we find evidence that cultural clusters (*CC*_*jk*_) are positive and significant (for all points awarded using both data sets). This finding demonstrates a higher degree of voting bias when awarding points to players from the same cultural society (the ‘in-group’) than awarding points to different cultural societies (the ‘outside group’). We also find that voting bias across cultural clusters (or ‘societies’) with positive and statistically significant coefficients on all points awarded.

With hypothesis 2, we also propose that higher voter collectivism (*VC*_*j*_) would positively influence voter bias. As shown in Table 4, our hypothesis is supported when 5 points are awarded for both Hofstede and GLOBE data sets. This indicates that voters from countries where responsibilities and social behaviors are driven by greater loyalty to their in-groups, tend to demonstrate a greater degree of voting bias (on average) than voters from more individualist countries. When lower points are awarded, 3 and 1 points, our findings are inconsistent with respect to voter collectivism. For Hofstede, voter collectivism is insignificant for 3 points awarded (estimated coefficient of 0.012 with *P* = 0.54) but significant for one point awarded (estimated coefficient of 0.17 and *P* = 0.011). For the GLOBE data set, both types of collectivism are insignificant for 3 and 1 points awarded. This finding could be due to the Hofstede data set being three times larger than GLOBE, thereby providing stronger evidence of voter collectivism. Interestingly, for the GLOBE dataset, the coefficient for collectivism II (in-group collectivism) consistently demonstrates a stronger influence on voting bias than collectivism I (institutional collectivism) through various models. When both forms of collectivism are run concurrently in the same model, collectivism I becomes insignificant (estimated coefficient of 0.05 and *P* = .31) and collectivism II remains positive and significant (estimated coefficient of 0.18 and *P* = 0.006).

Lastly, models 5 and 10 further test the individual effects of specific cultural dimensions from both Hofstede and GLOBE on voting bias (using Eq 4). We find that five of the six cultural dimensions for Hofstede and seven of the nine cultural dimensions for GLOBE are statistically significant using Eq 4. Two specific coefficients in model 10 (“institution collectivism” and “in-group collectivism”) are noteworthy for discussion as they are related to our second hypothesis. Both coefficients are negative and statistically significant. They are the absolute deviation between a voter and player’s cultural dimension for collectivism increases for both institutional and in-group collectivism (i.e., they become further apart), voting bias significantly decreases (*P* < 0.01) for all points awarded. This further supports our hypothesis that as voters and players become more culturally diverse, especially with respect to in-group collectivism, voting bias decreases on average.

As shown previously in Table 3, we also investigated the culturally related variables (i.e., the country level fragmentation indices relating to ethnicity, language, and religion). As the ethnicity, language, and religion compositions of a voter’s country become more fragmented (or fractionalized), voting bias significantly decreases for 5 points and 3 points awarded (and to a lesser extent for 1 point awarded–showing only the diversity of language having a negative and significant impact on voting bias). If a country has greater diversity with respect to cultural, ethnicity, and religion, there is sufficient evidence to show that voting bias decreases on average, compared to less diverse countries with respect to these characteristics.

Despite some inconsistency for voter collectivism at lower points awarded, we believe our findings provide sufficient evidence to support our second hypothesis that cultural factors are positively associated with voting bias in the Award.

### 4.4 Hypothesis 3 (Impartiality)

Although countries in cultural clusters exhibit a higher degree of voting bias than countries that are not in the cluster, they are expected to do so to a lesser extent as impartiality increases across countries within cultural clusters [9, 55]. Using the ICRG index and ‘Quality of Government’ as a proxy for impartiality, we model an interaction term (cultural clusters *CC*_*jk*_ x impartiality *Im*_*j*_) that is expected to be negative and significant, which implies that the level of voting bias in countries of cultural clusters diminishes as *impartiality Im*_*j*_ (the quality of governments) increases. As shown in Table 5, our results reveal no evidence to support this relationship. We find voting bias and impartiality to be positive and significant, as demonstrated by the interaction term for all points awarded using both Hofstede and GLOBE data sets.

**Table 5:** Hypothesis 3 (Impartiality): Regression models on voting bias for 5, 3 and 1 points awarded using both Hofstede and GLOBE data sets.

	Hofstede Data			GLOBE Data		
Variables	5 Points	3 Points	1 Point	5 Points	3 Points	1 Point
Media	-0.309***	-0.101**	-0.039	-0.350***	-0.081***	-0.106*
Coach	0.040	-0.093**	0.024	-0.027	0.017	0.012
2011	-0.589***	0.416***	0.356***	-0.356***	-0.084**	0.249**
2012	-0.314***	0.090	0.068	-0.120	0.093**	0.088
2013	0.225***	0.310***	0.109*	0.346***	0.139***	0.182*
2014	-0.159**	0.419***	0.226***	-0.114	0.201***	0.113
2015	-0.590***	0.300***	0.588***	-0.589***	-0.075*	0.452***
2016	-0.260**	0.266***	0.328***	-0.206*	0.107***	0.177*
Cultural clusters (*CC*_*jk*_)	0.358***	0.146***	0.297***	0.384***	0.115***	0.352***
Impartiality (*I*_*j*_)	-0.079	-0.025	-0.126	-0.232**	0.028	-0.213**
Cultural clusters (*CC*_*jk*_) * Impartiality (*I*_*j*_)	0.769***	0.354***	0.536***	0.671***	0.136**	0.480***
Goals/game	-2.031***	-2.010***	-1.418***	-2.068***	-0.796***	-1.603***
Assists/game	-1.873***	-0.548**	-0.614***	-2.279***	0.765***	-0.701**
Constant	4.968***	2.703***	0.705***	4.951***	2.655***	0.779***
Observations	2287	2287	2287	1046	1017	928
R^2^	0.669	0.476	0.349	0.701	0.523	0.399
Adjusted R^2^	0.667	0.474	0.346	0.697	0.518	0.392
F	670.9	262.0	113.4	319.5	117.5	59.38

Note: All models run with OLS using Huber White robust standard errors. Interaction effects were run separately to determine individual effects of interaction on cultural clusters.

***P <0.01

**P <0.05

*P <0.1.

The coefficient for impartiality is insignificant using Hofstede across all points awarded but is significant and negative using the GLOBE data set for 5 and 1 points awarded. This means that as the quality of government improves, there is some evidence to demonstrate that voting bias decreases on average. However, the effect of a voter and a player being in the same cultural cluster is stronger, not weaker, as the quality of government of the voter’s country increases. We find consistent results when using nationality within the interaction term instead of cultural clusters. In accordance with our third hypothesis, we would anticipate witnessing some evidence of impartiality in voting bias for voters and players who share the same nationality, but this is not the case again.

Based on these findings, there is insufficient evidence to support our final hypothesis of greater impartiality regarding cultural voting bias (i.e., demonstrating greater meritocracy over favoritism even within groups with strong cultural ties). We find the opposite, with evidence of a moderating effect of cultural and national influences on impartiality.

## 5. Conclusion

In this paper, we examine how cultural factors and established in-group factors impact voting bias in one of the world’s most prominent sporting awards. Our study and results constitute the most comprehensive study on voting bias in the Award and are robust, using both Hofstede and GLOBE datasets for cultural dimensions.

Consistent with previous literature on voting bias in awards and social identity theories, we find that nationality, the league, and the club are all important determinants of the voting bias in the Award. We add to existing research by showing that voter-player similarity in cultural distance, cultural clusters, and collectivism strongly influence voting bias. Furthermore, we find that countries with a greater diversity of ethnicity, language, and religion, demonstrate lower voting bias. We also find an increase in voting bias between voters and players of neighboring countries and between voters and players whose capital cities are in close geographical proximity. However, we found no evidence of impartiality in voting bias for voters and players that share the same cultural cluster or nationality.

In terms of voter type, voters from the media have the least amount of voting bias for all points awarded compared to captains and coaches, and this difference is consistently significant. Captains, on the other hand, have the greatest relative degree of voting bias compared to coaches or media, but this degree is only significant for the 5 points awarded. Captains are relatively closer to players in the context of voting, and this supports the theory that the proximity of the relationship through in-group and cultural factors may play an important role in voting bias.

Given the propensity for voting bias in awards, our research highlights the importance of having awards provide criteria concerning what constitutes quality. For the Award this would include total goals, goals per min, total assists, pass completions, player of the match, and saves. Such criteria could counter bias by better grounding, driving, and assessing voting choices. Also, if an award is relatively transparent concerning the voter-player factor similarities, as was the case with the Award, then this has two potential impacts. First, without such transparency, the award is more likely to escalate the effects of in-group bias while inviting irresponsible, naïve, and foolish voting decisions. Second, the information from such transparency could be used to analyze how votes were cast, like our study. Award organizers could use the analysis to identify and adjust any highly inappropriate bias while reporting and declaring the levels and types of bias exhibited. Such transparency and reporting would help make voters more aware of the propensity and impact of bias. Also, like studies of fake news that expose readers to simple accuracy reminders before consuming news to enhance truth discernment in participants’ [56], similar pre-voting mechanisms for bias could be developed for voters in awards. This mechanism would help voters recognize their biases before voting and mitigate how their biases impact voting choices.

Furthermore, our analysis of voting bias also provides a logic to help design awards where voters are required to only vote on candidates who are sufficiently dissimilar according to one or more in-group factors. For example, the NBA altered the voting process for its MVP awards in 2017 by removing media representatives with broadcasting ties that were closely associated with individual teams in a primary role [57]. Today, the NBA panel comprises 100 independent media members who have no direct ties to individual teams. Interestingly, if only media representatives voted during the seven years of the current study, it would have resulted in different winners in 2010 (Sneijder instead of Messi) and 2013 (Ribery instead of Ronaldo).

Finally, an important limitation of our study, which is also an opportunity for future research, is to control for voter taste for players who play in certain positions and play for the prestigious clubs and national teams. For player position, voters prefer forwards and midfielders over defenders and goalies, as creating and scoring goals in football is typically more entertaining and appreciated than tackling and saving shots. Similarly, there are halo effects [58] where the bias for a player is because of the reputation and performance of their team. For example, a defender who plays for F.C. Barcelona is likely to be more visible to voters and deemed of higher quality than a comparable defender who plays for West Ham United.

## Supporting information

S1 Appendix GLOBE’s Ten “Societal Clusters”. (DOCX)

S1 Data Supporting info voting data 31-05-22. (DOCX)
