# Supplementary material for: Cultural similarity and impartiality on voting bias: The case of FIFA’s World’s Best Male Football Player Award
Source: PLoS One. 2022 Jul 13;17(7):e0270546. doi: 10.1371/journal.pone.0270546 (PMC9278779; doi:10.1371/journal.pone.0270546)
Supplement: S1 Appendix — (DOCX) [file pone.0270546.s001.docx]

**Appendix 1. GLOBE’s Ten “Societal Clusters”**

| Societal Clusters | Countries |
| --- | --- |
| Sub-Saharan Africa | Angola, Antigua and Barbuda, Bahamas, Barbados, Belize, Benin, Botswana, Burkina, Faso, Burundi, Cameroon, Cape Verde, Central African Republic, Chad, Comoros, Congo, Congo DR, Cote d’Ivoire, Djibouti, Dominica, Equatorial Guinea, Eritrea, Ethiopia, Gabon, Gambia, Ghana, Grenada, Guinea, Guinea-Bissau, Haiti, Jamaica  Kenya, Lesotho, Liberia, Madagascar, Malawi, Mali, Mozambique, Namibia, Niger, Nigeria, Rwanda, Senegal, Seychelles, Sierra Leone, Somalia, South Africa, South Sudan, Swaziland, Tanzania, Togo, Trinidad and Tobago, Uganda, Zambia, Zimbabwe |
| Anglo-Saxon | Australia, Canada, Ireland, New Zealand, United Kingdom, USA |
| Confucian Asia | China PR, Japan, Mongolia, North Korea, Singapore, Korea Republic, Taiwan, Vietnam |
| Eastern Europe | Albania, Belarus, Bosnia, Bulgaria, Croatia, Cyprus, Czech Republic, Georgia, Greece  Hungary, Kazakhstan, Kyrgyzstan, FYR Macedonia, Montenegro, Poland, Russia, Serbia, Slovakia, Slovenia, Ukraine |
| Germanic Europe | Austria, Belgium, Germany, Liechtenstein, Luxembourg, The Netherlands, Switzerland |
| Latin America | Argentina, Bolivia, Brazil, Chile, Colombia, Costa Rica, Cuba, Dominican Republic, Ecuador, El Salvador, Guatemala, Honduras, Mexico, Nicaragua, Panama, Paraguay, Peru, Uruguay, Venezuela |
| Latin Europe | Andorra, France, Israel, Italy, Malta, Moldova, Monaco, Portugal, Romania, San Marino, Spain |
| Middle East | Algeria, Azerbaijan, Bahrain, Egypt, Iran, Iraq, Jordan, Kuwait, Lebanon, Libya, Mauritania, Morocco, Oman, Qatar, Saudi Arabia, Sudan, Syria, Tunisia, Turkey, Turkmenistan, United Arab Emirates, Uzbekistan, Yemen |
| Nordic Europe | Denmark, Estonia, Finland, Iceland, Latvia, Lithuania, Norway, Sweden |
| Southern Asia | Afghanistan, Bangladesh, Bhutan, Cambodia, Fiji, Guyana, India, Indonesia, Malaysia  Maldives, Mauritius, Myanmar, Nepal, Pakistan, Palau, Papua New Guinea, Philippines, Samoa, Sri Lanka, Suriname, Tajikistan, Thailand, Timor-Leste, Tonga,  New Caledonia |
