# Supplementary material for: Cultural similarity and impartiality on voting bias: The case of FIFA’s World’s Best Male Football Player Award
Source: PLoS One. 2022 Jul 13;17(7):e0270546. doi: 10.1371/journal.pone.0270546 (PMC9278779; doi:10.1371/journal.pone.0270546)
Supplement: S1 Data — (DOCX) [file pone.0270546.s002.docx]

**FIFA BALLON D'OR 2010**

**Votes by captain (men)**

| **Country** | **Name** | **First (5 points)** | **Second (3 points)** | **Third (1 point)** |
| --- | --- | --- | --- | --- |
| Albania | Lala Altin | Messi Lionel | Sneijder Wesley | Iniesta Andrés |
| Algeria | Ziani Karim | Xavi | Messi Lionel | Iniesta Andrés |
| Andorra | Sonejee Oscar | Xavi | Sneijder Wesley | Iniesta Andrés |
| Anguilla | Kelsick Romare | Puyol Carles | Messi Lionel | Júlio César |
| Antigua and Barbuda | Dublin George | Maicon | Sneijder Wesley | Robben Arjen |
| Armenia | Hovsepyan Sargis | Iniesta Andrés | Casillas Iker | Drogba Didier |
| Aruba | Escalona Maurice | Sneijder Wesley | Robben Arjen | Villa David |
| Australia | Neill Lucas | Xavi | Özil Mesut | Forlán Diego |
| Austria | Janko Marc | Iniesta Andrés | Xavi | Schweinsteiger Bastian |
| Azerbaijan | Sadigov Rashad | Sneijder Wesley | Xavi | Forlán Diego |
| Bahamas | Gavin Christie | Messi Lionel | Drogba Didier | Cristiano Ronaldo |
| Bangladesh | Hoque Aminul | Casillas Iker | Özil Mesut | Messi Lionel |
| Barbados | Forde Norman | Forlán Diego | Sneijder Wesley | Xavi |
| Belarus | Zhevnov Yury | Messi Lionel | Cristiano Ronaldo | Casillas Iker |
| Belgium | Vermaelen Thomas | Fábregas Cesc | Messi Lionel | Drogba Didier |
| Belize | Smith Elroy | Cristiano Ronaldo | Messi Lionel | Forlán Diego |
| Bermuda | Ming Damon | Messi Lionel | Drogba Didier | Forlán Diego |
| Bosnia-Herzegovina | Spahic Emir | Xavi | Iniesta Andrés | Casillas Iker |
| Botswana | Mompati Apache Thuma | Xavi | Messi Lionel | Forlán Diego |
| Brazil | De Souza Robson | Messi Lionel | Xavi | Forlán Diego |
| British Virgin Islands | Davis Andy | Drogba Didier | Messi Lionel | Fábregas Cesc |
| Bulgaria | Petrov Stiliyan | Sneijder Wesley | Xavi | Özil Mesut |
| Burundi | Niyonkuru Vladimir | Eto'o Samuel | Messi Lionel | Alves Daniel |
| Cambodia | Ouk Mic | Casillas Iker | Özil Mesut | Villa David |
| Cameroon | Samuel Eto'o Fils | Sneijder Wesley | Xavi | Iniesta Andrés |
| Canada | Stalteri Paul | Xavi | Messi Lionel | Cristiano Ronaldo |
| Cape Verde Islands | Aguiar Claudio | Messi Lionel | Cristiano Ronaldo | Xavi |
| Chad | Djerabe Armand | Iniesta Andrés | Cristiano Ronaldo | Messi Lionel |
| China PR | Du Wei | Xavi | Messi Lionel | Forlán Diego |
| Chinese Taipei | Po-Liang Chen | Messi Lionel | Cristiano Ronaldo | Xavi |
| Colombia | Yepes Mario | Alonso Xabi | Messi Lionel | Forlán Diego |
| Comoros | Captain of the national team | Messi Lionel | Alonso Xabi | Eto'o Samuel |
| Cook Islands | Pareanga John | Messi Lionel | Forlán Diego | Puyol Carles |
| Costa Rica | Ruiz Bryan | Messi Lionel | Iniesta Andrés | Sneijder Wesley |
| Croatia | Srna Darijo | Messi Lionel | Drogba Didier | Özil Mesut |
| Cuba | Colome Jaine | Messi Lionel | Alonso Xabi | Alves Daniel |
| Cyprus | Okkas Ioannis | Messi Lionel | Iniesta Andrés | Forlán Diego |
| Czech Republic | Rosicky Tomas | Messi Lionel | Xavi | Sneijder Wesley |
| Djibouti | Charmare Miad Nour | Xavi | Iniesta Andrés | Sneijder Wesley |
| Dominica | Glenson Prince | Schweinsteiger Bastian | Casillas Iker | invalid vote |
| Ecuador | Corozo Walter Ayovi | Eto'o Samuel | Messi Lionel | Özil Mesut |
| El Salvador | Dagoberto Portillo Gamero | Xavi | Messi Lionel | Casillas Iker |
| England | Rio Ferdinand | Sneijder Wesley | Iniesta Andrés | Xavi |

| Equatorial Guinea | Captain of the national team | Casillas Iker | Drogba Didier | Iniesta Andrés |
| --- | --- | --- | --- | --- |
| Estonia | Raio Piiroja | Forlán Diego | Iniesta Andrés | Messi Lionel |
| Finland | Hyypiä Sami | Puyol Carles | Schweinsteiger Bastian | Forlán Diego |
| France | Diarra Alou | Iniesta Andrés | Xavi | Sneijder Wesley |
| FYR Macedonia | Pandev Goran | Sneijder Wesley | Maicon | Júlio César |
| Gabon | Cousin Daniel | Messi Lionel | Robben Arjen | Iniesta Andrés |
| Gambia | Pa Dembo Touray | Iniesta Andrés | Forlán Diego | Gyan Asamoah |
| Georgia | Kakha Kaladze | Messi Lionel | Iniesta Andrés | Júlio César |
| Germany | Lahm Philipp | Iniesta Andrés | Sneijder Wesley | Messi Lionel |
| Greece | Karagkounis Georgios | Iniesta Andrés | Forlán Diego | Júlio César |
| Guam | Gadia Dominic | Iniesta Andrés | Sneijder Wesley | Drogba Didier |
| Guatemala | Cabrera Gustavo | Forlán Diego | Casillas Iker | Alonso Xabi |
| Guinea | Zayatte Kamil | Sneijder Wesley | Messi Lionel | Casillas Iker |
| Guyana | Lowe Howard | Villa David | Drogba Didier | Fábregas Cesc |
| Honduras | Valladares Bonilla Noel Eduardo | Messi Lionel | Cristiano Ronaldo | Drogba Didier |
| Hong Kong | Haigiang Li | Villa David | Iniesta Andrés | Casillas Iker |
| Hungary | Gera Zoltan | Messi Lionel | Iniesta Andrés | Forlán Diego |
| Iceland | Hreidarsson Hermann | Messi Lionel | Iniesta Andrés | Sneijder Wesley |
| India | Bhaichung Bhutia | Sneijder Wesley | Alonso Xabi | Iniesta Andrés |
| Iran | Nekounam Javad | Messi Lionel | Xavi | Iniesta Andrés |
| Israel | Yossi Benayoun | Drogba Didier | Alonso Xabi | Messi Lionel |
| Italy | Buffon Gianluigi | Iniesta Andrés | Sneijder Wesley | Casillas Iker |
| Jamaica | Shavar Thomas | Forlán Diego | Drogba Didier | Maicon |
| Japan | Hasebe Makoto | Xavi | Messi Lionel | Özil Mesut |
| Jordan | Hassoneh Alshekh | Xavi | Messi Lionel | Iniesta Andrés |
| Kazakhstan | Nurbol Zhumaskaliev | Messi Lionel | Xavi | Özil Mesut |
| Korea Republic | Park Ji Sung | Sneijder Wesley | Alonso Xabi | Forlán Diego |
| Kuwait | Nawaf Al Khaldi | Sneijder Wesley | Iniesta Andrés | Messi Lionel |
| Latvia | Gorkss Kaspars | Xavi | Forlán Diego | Messi Lionel |
| Lebanon | Alsaadi Ali | Messi Lionel | Sneijder Wesley | Xavi |
| Lesotho | Makepe Basia | Xavi | Messi Lionel | Iniesta Andrés |
| Liberia | Bill Pautae | Messi Lionel | Eto'o Samuel | Drogba Didier |
| Liechtenstein | Frick Mario | Messi Lionel | Eto'o Samuel | Iniesta Andrés |
| Lithuania | Danilevicius Tomas | Iniesta Andrés | Sneijder Wesley | Forlán Diego |
| Luxembourg | Strasser Jeff | Messi Lionel | Iniesta Andrés | Cristiano Ronaldo |
| Macau | Cheang Cheng Ieong | Sneijder Wesley | Iniesta Andrés | Casillas Iker |
| Madagascar | Razafindrabe Eric | Xavi | Iniesta Andrés | Messi Lionel |
| Malawi | Elvis Kafoteka | Xavi | Forlán Diego | Gyan Asamoah |
| Malaysia | Norhafiz Zamani Misbah | Xavi | Sneijder Wesley | Puyol Carles |
| Malta | Mifsud Michael | Messi Lionel | Klose Miroslav | Xavi |
| Mauritania | Ba Mohamed Yacoub Deina | Iniesta Andrés | Messi Lionel | Sneijder Wesley |
| Mauritius | Canasamy Johan Clyde | Puyol Carles | Iniesta Andrés | Xavi |
| Mexico | Diez de Bonilla Gerardo Torrado | Forlán Diego | Alonso Xabi | Messi Lionel |
| Moldova | Epurenanu Alexandru | Messi Lionel | Sneijder Wesley | Iniesta Andrés |
| Mongolia | Lumbengarav Donorov | Messi Lionel | Robben Arjen | Forlán Diego |
| Montserrat | Clifford Joseph | Messi Lionel | Cristiano Ronaldo | Casillas Iker |
| Mozambique | Celso Abdul | Xavi | Messi Lionel | Iniesta Andrés |

| Myanmar | Zaw Lin Tun | Casillas Iker | Sneijder Wesley | Müller Thomas |
| --- | --- | --- | --- | --- |
| Namibia | Jacobs Quinton | Messi Lionel | Xavi | Iniesta Andrés |
| Netherlands | Van Bommel Mark | invalid vote | invalid vote | Schweinsteiger Bastian |
| New Caledonia | Wajoka Pierre | Messi Lionel | Forlán Diego | Puyol Carles |
| Nicaragua | Solorzano David | Messi Lionel | Casillas Iker | Xavi |
| Nigeria | Yobo Joseph | Messi Lionel | Sneijder Wesley | Maicon |
| Northern Ireland | Hughes Aaron | Xavi | Messi Lionel | Schweinsteiger Bastian |
| Oman | Rabee Mohammed | Iniesta Andrés | Sneijder Wesley | Forlán Diego |
| Peru | Vilchez Walter | Forlán Diego | Messi Lionel | Xavi |
| Philippines | Borromeo Alexander Charles | Cristiano Ronaldo | Messi Lionel | Forlán Diego |
| Poland | Zewlakow Michal | Messi Lionel | Casillas Iker | Xavi |
| Portugal | Cristiano Ronaldo | Xavi | Casillas Iker | Sneijder Wesley |
| Romania | Chivu Christian | Sneijder Wesley | Xavi | Robben Arjen |
| Russia | Arshavin Andrey | Messi Lionel | Cristiano Ronaldo | Xavi |
| San Marino | Selva Andy | Messi Lionel | Iniesta Andrés | Drogba Didier |
| São Tomé e Príncipe | Da Costa Paulino | Eto'o Samuel | Drogba Didier | Gyan Asamoah |
| Scotland | Fletcher Darren | Xavi | Messi Lionel | Sneijder Wesley |
| Senegal | Niang Mamadou | Messi Lionel | Cristiano Ronaldo | Iniesta Andrés |
| Serbia | Stankovic Dejan | Sneijder Wesley | Eto'o Samuel | Maicon |
| Singapore | Shahril Ishak | Forlán Diego | Puyol Carles | Müller Thomas |
| Slovakia | Hamsik Marek | Messi Lionel | Robben Arjen | Cristiano Ronaldo |
| Slovenia | Koren Robert | Iniesta Andrés | Messi Lionel | Cristiano Ronaldo |
| Solomon Islands | Rukumana Eddie | Schweinsteiger Bastian | Villa David | Drogba Didier |
| Somalia | Ali Yassin | Messi Lionel | Xavi | Cristiano Ronaldo |
| Spain | Casillas Iker | Robben Arjen | Sneijder Wesley | Cristiano Ronaldo |
| Sri Lanka | Ruwantilake Rohana | Iniesta Andrés | Forlán Diego | Robben Arjen |
| St. Kitts and Nevis | Leader Thrizen | Iniesta Andrés | Messi Lionel | Sneijder Wesley |
| Suriname | Garden Derrick | Messi Lionel | Xavi | Iniesta Andrés |
| Swaziland | Mtsetfwa Mxolisi | Messi Lionel | Xavi | Puyol Carles |
| Sweden | Ibrahimovic Zlatan | Iniesta Andrés | Sneijder Wesley | Xavi |
| Switzerland | Frei Alexander | Xavi | Schweinsteiger Bastian | Casillas Iker |
| Tajikistan | Rabiev Yusuf | Xavi | Iniesta Andrés | Messi Lionel |
| Thailand | Nattaporn Phanrit | Villa David | Forlán Diego | Klose Miroslav |
| Trinidad and Tobago | Clyde Leon | Sneijder Wesley | Messi Lionel | Özil Mesut |
| Tunisia | Yahia Alaeddine | Xavi | Puyol Carles | Casillas Iker |
| Turkey | Emre Belozoglu | Casillas Iker | Özil Mesut | Puyol Carles |
| Turks and Caicos Islands | Shearer Phil | Xavi | Sneijder Wesley | Iniesta Andrés |
| Uganda | Sekaggya Ibrahim | Messi Lionel | Iniesta Andrés | Forlán Diego |
| Ukraine | Shevchenko Andriy | Iniesta Andrés | Messi Lionel | Eto'o Samuel |
| Uruguay | Lugano Diego | Sneijder Wesley | Xavi | Messi Lionel |
| USA | Bocanegra Carlos | Xavi | Iniesta Andrés | Sneijder Wesley |
| Uzbekistan | Ahmedov Odil | Messi Lionel | Puyol Carles | Eto'o Samuel |
| Vanuatu | Poida Moise | Messi Lionel | Müller Thomas | Eto'o Samuel |
| Venezuela | Lucena Franklin | Messi Lionel | Xavi | Forlán Diego |
| Vietnam | Nguyen Minh Phuong | Xavi | Sneijder Wesley | Iniesta Andrés |
| Zambia | Katongo Cris | Alonso Xabi | Iniesta Andrés | Puyol Carles |

**Votes by coach (men)**

| **Country** | **Name** | **First (5 points)** | **Second (3 points)** | **Third (1 point)** |
| --- | --- | --- | --- | --- |
| Albania | Kuze Josip | Messi Lionel | Iniesta Andrés | Maicon |
| Algeria | Benchikha Abdelhak | Cristiano Ronaldo | Casillas Iker | Forlán Diego |
| Andorra | Alvarez de Eulate Koldo | Xavi | Iniesta Andrés | Sneijder Wesley |
| Anguilla | Johnson Colin | Messi Lionel | Cristiano Ronaldo | Sneijder Wesley |
| Antigua and Barbuda | Rowan Benjamin | Drogba Didier | Iniesta Andrés | Messi Lionel |
| Armenia | Minasyan Vardan | Xavi | Messi Lionel | Sneijder Wesley |
| Aruba | Rasmijn Marco | Sneijder Wesley | Robben Arjen | Villa David |
| Australia | Osieck Holger | Sneijder Wesley | Villa David | Özil Mesut |
| Austria | Constantini Dietmar | Iniesta Andrés | Villa David | Klose Miroslav |
| Azerbaijan | Vogts Berti | Forlán Diego | Müller Thomas | Casillas Iker |
| Bahamas | James Paul | Messi Lionel | Forlán Diego | Drogba Didier |
| Bangladesh | Rubcic Robert | Iniesta Andrés | Messi Lionel | Forlán Diego |
| Barbados | Jordan Thomas | Forlán Diego | Sneijder Wesley | Xavi |
| Belarus | Stange Bernd | Messi Lionel | Forlán Diego | Xavi |
| Belgium | Leekens Georges | Messi Lionel | Iniesta Andrés | Özil Mesut |
| Belize | De la Paz Herrera José | Messi Lionel | Cristiano Ronaldo | Iniesta Andrés |
| Bermuda | Scott Morton | Xavi | Maicon | Forlán Diego |
| Bosnia-Herzegovina | Susic Safet | Cristiano Ronaldo | Messi Lionel | Iniesta Andrés |
| Brazil | Menezes Luiz Antonio | Messi Lionel | Xavi | Robben Arjen |
| British Virgin Islands | Williams Avondale | Messi Lionel | Iniesta Andrés | Xavi |
| Bulgaria | Matthäus Lothar | Messi Lionel | Iniesta Andrés | Müller Thomas |
| Burkina Faso | Paulo Duarte | Cristiano Ronaldo | Messi Lionel | Alonso Xabi |
| Burundi | Amrouche Adel | Iniesta Andrés | Drogba Didier | Lahm Philipp |
| Cambodia | Lee Tae Hoon | Özil Mesut | Messi Lionel | Xavi |
| Cameroon | Lazaro Javier Clemente | Xavi | Iniesta Andrés | Puyol Carles |
| Canada | Hart Stephen | Sneijder Wesley | Xavi | Casillas Iker |
| Cape Verde Islands | Antunes Lucio | Cristiano Ronaldo | Messi Lionel | Xavi |
| Chad | Al Khashab Sherif | Iniesta Andrés | Cristiano Ronaldo | Messi Lionel |
| China PR | Gao Hongbo | Xavi | Messi Lionel | Forlán Diego |
| Chinese Taipei | Chih-Tsung Lo | Messi Lionel | Alonso Xabi | Eto'o Samuel |
| Colombia | Gómez Dario | Iniesta Andrés | Messi Lionel | Robben Arjen |
| Cook Islands | Tillotson Maurice | Messi Lionel | Forlán Diego | Puyol Carles |
| Costa Rica | La Volpe Ricardo | Messi Lionel | invalid vote | Cristiano Ronaldo |
| Croatia | Bilic Slaven | Messi Lionel | Xavi | Sneijder Wesley |
| Cuba | Gonzalez Raul | Messi Lionel | Alonso Xabi | Forlán Diego |
| Cyprus | Anastasiades Angelos | Messi Lionel | Iniesta Andrés | Alonso Xabi |
| Czech Republic | Bilek Michal | Messi Lionel | Cristiano Ronaldo | Xavi |
| Djibouti | Mahfoud Omar | Sneijder Wesley | Iniesta Andrés | Xavi |
| Ecuador | Rueda Reinaldo | Xavi | Messi Lionel | Müller Thomas |
| El Salvador | Jose Luis Rugamas | Messi Lionel | Xavi | Cristiano Ronaldo |
| England | Capello Fabio | Iniesta Andrés | Xavi | Maicon |
| Equatorial Guinea | Casto Nopo | Casillas Iker | Cristiano Ronaldo | Drogba Didier |
| Estonia | Rüütli Tarmo | Forlán Diego | Iniesta Andrés | Messi Lionel |
| Fiji | Dutt Yogendra | Messi Lionel | Casillas Iker | Drogba Didier |
| Finland | Baxter Stuart | Messi Lionel | Iniesta Andrés | Cristiano Ronaldo |

| France | Blanc Laurent | Xavi | Sneijder Wesley | Iniesta Andrés |
| --- | --- | --- | --- | --- |
| FYR Macedonia | Jonuz Mirsad | Xavi | Sneijder Wesley | Maicon |
| Gabon | Rohr Gernot | Iniesta Andrés | Müller Thomas | Messi Lionel |
| Gambia | Put Paul | Iniesta Andrés | Messi Lionel | Forlán Diego |
| Georgia | Temur Ketsbaia | Xavi | Robben Arjen | Forlán Diego |
| Germany | Löw Joachim | Xavi | Messi Lionel | Forlán Diego |
| Ghana | Appiah James | Messi Lionel | invalid vote | Cristiano Ronaldo |
| Greece | Costa Santos Fernando | Messi Lionel | Cristiano Ronaldo | Sneijder Wesley |
| Guam | Tsukitate Norio | Iniesta Andrés | Messi Lionel | Schweinsteiger Bastian |
| Guatemala | Ever Hugo Almeida | Cristiano Ronaldo | Messi Lionel | Casillas Iker |
| Guinea | Dussuyer Michel | Iniesta Andrés | Schweinsteiger Bastian | Sneijder Wesley |
| Guyana | Dover Wayne | Drogba Didier | Sneijder Wesley | Iniesta Andrés |
| Honduras | Juan De Dios Castillo Gonzalez | Messi Lionel | Xavi | Eto'o Samuel |
| Hong Kong | Tsang Wai-Chung | Xavi | Puyol Carles | Lahm Philipp |
| Hungary | Sandor Egervari | Xavi | Sneijder Wesley | Iniesta Andrés |
| Iceland | Johannesson Olafur | Iniesta Andrés | Sneijder Wesley | Xavi |
| India | Houghton Robert | Cristiano Ronaldo | Messi Lionel | Villa David |
| Iran | Ghotbi Afshin | Xavi | Messi Lionel | Sneijder Wesley |
| Israel | Fernandez Luis | Iniesta Andrés | Xavi | Eto'o Samuel |
| Italy | Prandelli Cesare | Sneijder Wesley | Iniesta Andrés | Messi Lionel |
| Jamaica | Whitmore Theodore | Messi Lionel | Cristiano Ronaldo | Xavi |
| Japan | Zaccheroni Alberto | Messi Lionel | Sneijder Wesley | Robben Arjen |
| Jordan | Adnan Hamad Alabbasi | Messi Lionel | Xavi | Cristiano Ronaldo |
| Korea Republic | Cho Kwang Rae | Messi Lionel | Alonso Xabi | Drogba Didier |
| Kuwait | Tufegddzic Goran | Sneijder Wesley | Iniesta Andrés | Messi Lionel |
| Latvia | Starkov Aleksander | Iniesta Andrés | Xavi | Forlán Diego |
| Lebanon | Rustom Emile | Messi Lionel | Xavi | Sneijder Wesley |
| Lesotho | Notsi Leslie | Xavi | Messi Lionel | Iniesta Andrés |
| Liechtenstein | Zaugg Bidu | Xavi | Messi Lionel | Iniesta Andrés |
| Lithuania | Zutautas Raimondas | Xavi | Casillas Iker | Cristiano Ronaldo |
| Luxembourg | Holtz Luc | Iniesta Andrés | Messi Lionel | Sneijder Wesley |
| Macau | Leung Sui Wing | Messi Lionel | Cristiano Ronaldo | Casillas Iker |
| Madagascar | Rajaonarisamba Franck | Messi Lionel | Xavi | Cristiano Ronaldo |
| Malawi | Kinna Phiri | Messi Lionel | Sneijder Wesley | Forlán Diego |
| Malaysia | Rajagobal K. | Iniesta Andrés | Xavi | Özil Mesut |
| Malta | Buttigieg John | Puyol Carles | Iniesta Andrés | invalid vote |
| Mauritania | Sall Moustapha | Xavi | Messi Lionel | Forlán Diego |
| Mauritius | Patel Akbar | Villa David | Müller Thomas | Özil Mesut |
| Mexico | De La Torre Menchaca Jose Manuel | Forlán Diego | Alonso Xabi | Messi Lionel |
| Moldova | Balint Gavril Pele | Messi Lionel | Xavi | Forlán Diego |
| Mongolia | Erdenebat Sandagdori | Villa David | Iniesta Andrés | Forlán Diego |
| Montenegro | Zlatko Kranjcar | Messi Lionel | Cristiano Ronaldo | Forlán Diego |
| Montserrat | Dyer Kenneth | Messi Lionel | Robben Arjen | Forlán Diego |
| Mozambique | Chissano Joao | Xavi | Messi Lionel | Iniesta Andrés |
| Myanmar | Tin Myint Aung | Xavi | Messi Lionel | Sneijder Wesley |
| Namibia | Isaacs Brian | Xavi | Messi Lionel | Cristiano Ronaldo |
| Netherlands | Van Marwijk Bert | invalid vote | Messi Lionel | Xavi |

| New Caledonia | Coursimault Christophe | Messi Lionel | Forlán Diego | Puyol Carles |
| --- | --- | --- | --- | --- |
| New Zealand | Ricki Herbert | Messi Lionel | Müller Thomas | Sneijder Wesley |
| Nicaragua | Llena Leon Enrique | Messi Lionel | Iniesta Andrés | Özil Mesut |
| Nigeria | Samson Siasia | Iniesta Andrés | Sneijder Wesley | Forlán Diego |
| Northern Ireland | Worthington Nigel | Iniesta Andrés | Messi Lionel | Özil Mesut |
| Oman | Le Roy Claude | Iniesta Andrés | Sneijder Wesley | Forlán Diego |
| Peru | Markarian Sergio | Forlán Diego | Messi Lionel | Xavi |
| Philippines | MCMenemy Simon | Villa David | Müller Thomas | Forlán Diego |
| Poland | Smuda Franciszek | Iniesta Andrés | Xavi | Eto'o Samuel |
| Portugal | Bento Paulo | Xavi | Messi Lionel | Iniesta Andrés |
| Romania | Lucescu Razvan | Sneijder Wesley | Puyol Carles | Eto'o Samuel |
| Russia | Advocaat D.N. | Messi Lionel | Sneijder Wesley | Schweinsteiger Bastian |
| San Marino | Mazza Giampaolo | Iniesta Andrés | Sneijder Wesley | Eto'o Samuel |
| São Tomé e Príncipe | Coach of the national team | Messi Lionel | Eto'o Samuel | Júlio César |
| Scotland | Levein Craig | Messi Lionel | Iniesta Andrés | Puyol Carles |
| Senegal | Traoré Amara | Xavi | Iniesta Andrés | Sneijder Wesley |
| Serbia | Petrovic Vladimir | Messi Lionel | Iniesta Andrés | Xavi |
| Seychelles | Morrison Andrew | Drogba Didier | Iniesta Andrés | Xavi |
| Singapore | Avramovic Radojko | Sneijder Wesley | Iniesta Andrés | Forlán Diego |
| Slovakia | Weiss Vladimir | Messi Lionel | Cristiano Ronaldo | Forlán Diego |
| Slovenia | Kek Matjaz | Iniesta Andrés | Messi Lionel | Forlán Diego |
| Solomon Islands | Moli Jacob | Iniesta Andrés | Müller Thomas | Sneijder Wesley |
| Somalia | Abdulla Hussien | Messi Lionel | Özil Mesut | Iniesta Andrés |
| Spain | Del Bosque Vicente | Messi Lionel | Cristiano Ronaldo | Schweinsteiger Bastian |
| Sri Lanka | Chandrasiri D. | Alonso Xabi | Júlio César | Messi Lionel |
| Suriname | Baal Delano | Messi Lionel | Iniesta Andrés | Xavi |
| Swaziland | Zwane Musa | Cristiano Ronaldo | Messi Lionel | Gyan Asamoah |
| Sweden | Hamren Erik | Sneijder Wesley | Puyol Carles | Forlán Diego |
| Switzerland | Hitzfeld Ottmar | Messi Lionel | Sneijder Wesley | Forlán Diego |
| Tahiti | Etaeta Eddy | Xavi | Iniesta Andrés | Messi Lionel |
| Tajikistan | Kodirov Pulod | Messi Lionel | Cristiano Ronaldo | Maicon |
| Thailand | Robson Bryan | Villa David | Forlán Diego | Klose Miroslav |
| Trinidad and Tobago | Latapy Russell | Messi Lionel | Sneijder Wesley | Özil Mesut |
| Tunisia | Marchand Bertrand | Xavi | Messi Lionel | Forlán Diego |
| Turkey | Guus Hiddink | Xavi | Sneijder Wesley | Messi Lionel |
| Turks and Caicos Islands | Brough Gary | Messi Lionel | Cristiano Ronaldo | Fábregas Cesc |
| Uganda | Williamson Robert | Iniesta Andrés | Gyan Asamoah | Sneijder Wesley |
| Ukraine | Kalitvintsev Yuriy | Xavi | Sneijder Wesley | Iniesta Andrés |
| Uruguay | Tabárez Oscar | Messi Lionel | Xavi | Müller Thomas |
| USA | Bradley Robert | Xavi | Sneijder Wesley | Messi Lionel |
| Uzbekistan | Abramov Vadim | Messi Lionel | Iniesta Andrés | Xavi |
| Vanuatu | Sokomanu Toroi | Messi Lionel | Müller Thomas | Cristiano Ronaldo |
| Venezuela | Farias Cesar | Forlán Diego | Maicon | Messi Lionel |
| Vietnam | Calisto Henrique | Cristiano Ronaldo | Messi Lionel | Xavi |

**Votes by media**

| **Country** | **Name** | **First (5 points)** | **Second (3 points)** | **Third (1 point)** |
| --- | --- | --- | --- | --- |
| Albania | Dizdari Besnik | Iniesta Andrés / Spain | Forlán Diego / Uruguay | Sneijder Wesley / Netherlands |
| Algeria | Ouahib Yazid | Messi Lionel / Argentina | Iniesta Andrés / Spain | Xavi / Spain |
| Andorra | Rodriguez "Tonono" Manuel | Messi Lionel / Argentina | Xavi / Spain | Robben Arjen / Netherlands |
| Angola | Goncalves Mateus | Sneijder Wesley / Netherlands | Iniesta Andrés / Spain | Forlán Diego / Uruguay |
| Argentina | Sacco Enrique | Messi Lionel / Argentina | Iniesta Andrés / Spain | Forlán Diego / Uruguay |
| Armenia | Baghdasarian Souren | Sneijder Wesley / Netherlands | Iniesta Andrés / Spain | Forlán Diego / Uruguay |
| Australia | Foster Craig | Xavi / Spain | Sneijder Wesley / Netherlands | Messi Lionel / Argentina |
| Austria | Kowatsch-Schwarz Walter | Iniesta Andrés / Spain | Forlán Diego / Uruguay | Messi Lionel / Argentina |
| Azerbaijan | Mövsümov Rasim | Iniesta Andrés / Spain | Xavi / Spain | Özil Mesut / Germany |
| Bahrain | Ashoor Abdullah | Xavi / Spain | Sneijder Wesley / Netherlands | Messi Lionel / Argentina |
| Bangladesh | Mahamud Raihan | Messi Lionel / Argentina | Cristiano Ronaldo / Portugal | Schweinsteiger Bastian / Germany |
| Belarus | Nikolaev Sergey | Sneijder Wesley / Netherlands | Villa David / Spain | Forlán Diego / Uruguay |
| Belgium | Dubois Michel | Sneijder Wesley / Netherlands | Iniesta Andrés / Spain | Messi Lionel / Argentina |
| Benin | Sagbo René | Iniesta Andrés / Spain | Cristiano Ronaldo / Portugal | Forlán Diego / Uruguay |
| Bolivia | Rivera Carlos Enrique | Forlán Diego / Uruguay | Iniesta Andrés / Spain | Messi Lionel / Argentina |
| Bosnia-Herzegovina | Buric Ahmed | Messi Lionel / Argentina | Casillas Iker / Spain | Iniesta Andrés / Spain |
| Botswana | Mathala Oaitse | Sneijder Wesley / Netherlands | Xavi / Spain | Forlán Diego / Uruguay |
| Brazil | Machado Cleber | Xavi / Spain | Iniesta Andrés / Spain | Sneijder Wesley / Netherlands |
| Bulgaria | Savov / Paytashev Michel / Roumen | Forlán Diego / Uruguay | Xavi / Spain | Sneijder Wesley / Netherlands |
| Burkina Faso | Hien Victorien Marie | Forlán Diego / Uruguay | Iniesta Andrés / Spain | Robben Arjen / Netherlands |
| Burundi | Hatungimana Désiré | Iniesta Andrés / Spain | Eto'o Samuel / Cameroon | Messi Lionel / Argentina |
| Cambodia | Chamroeun Ung | Casillas Iker / Spain | Messi Lionel / Argentina | Gyan Asamoah / Ghana |
| Cameroon | Mbeze Brice | Iniesta Andrés / Spain | Sneijder Wesley / Netherlands | Eto'o Samuel / Cameroon |
| Canada | Davidson Neil | Xavi / Spain | Messi Lionel / Argentina | Forlán Diego / Uruguay |
| Central African Republic | Koumbobacko Albert Stanislas | Eto'o Samuel / Cameroon | Iniesta Andrés / Spain | Forlán Diego / Uruguay |
| Chad | Topona Eric | Gyan Asamoah / Ghana | Messi Lionel / Argentina | Eto'o Samuel / Cameroon |
| Chile | Diaz Danilo | Iniesta Andrés / Spain | Sneijder Wesley / Netherlands | Forlán Diego / Uruguay |
| China PR | Ming Luo | Sneijder Wesley / Netherlands | Xavi / Spain | Iniesta Andrés / Spain |
| Colombia | Andrade Paché | Iniesta Andrés / Spain | Messi Lionel / Argentina | Forlán Diego / Uruguay |
| Comoros | Boina Abdou | Sneijder Wesley / Netherlands | Forlán Diego / Uruguay | Müller Thomas / Germany |
| Congo | Golden Eloue James | Sneijder Wesley / Netherlands | Iniesta Andrés / Spain | Forlán Diego / Uruguay |
| Congo DR | Katambwe Wa Kazadi de Sikasso | Alonso Xabi / Spain | Casillas Iker / Spain | Messi Lionel / Argentina |
| Costa Rica | Calvo Castro Rodrigo Antonio | Xavi / Spain | Iniesta Andrés / Spain | Forlán Diego / Uruguay |
| Côte d'Ivoire | Khalil Adam | Xavi / Spain | Sneijder Wesley / Netherlands | Iniesta Andrés / Spain |
| Croatia | Reic Zdravko | Iniesta Andrés / Spain | Sneijder Wesley / Netherlands | Forlán Diego / Uruguay |
| Cuba | Hernandez Miguel | Forlán Diego / Uruguay | Casillas Iker / Spain | Messi Lionel / Argentina |
| Cyprus | Gavrielides Michel | Iniesta Andrés / Spain | Xavi / Spain | Forlán Diego / Uruguay |
| Czech Republic | Hrabe Stanislav | Forlán Diego / Uruguay | Casillas Iker / Spain | Messi Lionel / Argentina |
| Denmark | Larsen Niels Jörgen | Xavi / Spain | Sneijder Wesley / Netherlands | Messi Lionel / Argentina |
| Djibouti | Ibrahime Ibrahim Mohamed | Iniesta Andrés / Spain | Sneijder Wesley / Netherlands | Xavi / Spain |
| Dominican Republic | Bauger Jorge Rolando | Xavi / Spain | Sneijder Wesley / Netherlands | Messi Lionel / Argentina |
| Ecuador | Zavala Garcia Fabricio | Iniesta Andrés / Spain | Sneijder Wesley / Netherlands | Forlán Diego / Uruguay |
| Egypt | Mazhar Inas | Iniesta Andrés / Spain | Messi Lionel / Argentina | Forlán Diego / Uruguay |
| El Salvador | Posada Flores Mario Ernesto | Xavi / Spain | Sneijder Wesley / Netherlands | Iniesta Andrés / Spain |

| England | Winter Henry | Sneijder Wesley / Netherlands | Iniesta Andrés / Spain | Schweinsteiger Bastian / Germany |
| --- | --- | --- | --- | --- |
| Equatorial Guinea | Monsuy David | Xavi / Spain | Sneijder Wesley / Netherlands | Iniesta Andrés / Spain |
| Estonia | Luik Margus | Sneijder Wesley / Netherlands | Forlán Diego / Uruguay | Xavi / Spain |
| Finland | Einiö Matti | Puyol Carles / Spain | Messi Lionel / Argentina | Sneijder Wesley / Netherlands |
| France | Chaumier Denis | Messi Lionel / Argentina | Iniesta Andrés / Spain | Eto'o Samuel / Cameroon |
| FYR Macedonia | Timkovski Boro | Sneijder Wesley / Netherlands | Iniesta Andrés / Spain | Forlán Diego / Uruguay |
| Gabon | Loundou James Angelo | Xavi / Spain | Sneijder Wesley / Netherlands | Messi Lionel / Argentina |
| Gambia | Keita Nanama | Cristiano Ronaldo / Portugal | Messi Lionel / Argentina | Eto'o Samuel / Cameroon |
| Georgia | Potskhveria / Bzikadze Zurab / Vakhtang | Villa David / Spain | Forlán Diego / Uruguay | Klose Miroslav / Germany |
| Germany | Wild Karl Heinz | Sneijder Wesley / Netherlands | Messi Lionel / Argentina | Robben Arjen / Netherlands |
| Ghana | Oti Adjei Michael | Sneijder Wesley / Netherlands | Iniesta Andrés / Spain | Forlán Diego / Uruguay |
| Greece | Staramopoulos Manos | Sneijder Wesley / Netherlands | Xavi / Spain | Iniesta Andrés / Spain |
| Guatemala | Aguilar Francisco | Iniesta Andrés / Spain | Casillas Iker / Spain | Sneijder Wesley / Netherlands |
| Guinea | Diallo Ibrahima | Sneijder Wesley / Netherlands | Iniesta Andrés / Spain | Forlán Diego / Uruguay |
| Haiti | Nere Enock | Iniesta Andrés / Spain | Forlán Diego / Uruguay | Sneijder Wesley / Netherlands |
| Honduras | Garcia Francisco Antonio Rivas | Xavi / Spain | Forlán Diego / Uruguay | Müller Thomas / Germany |
| Hungary | Imre Mathias | Sneijder Wesley / Netherlands | Iniesta Andrés / Spain | Xavi / Spain |
| Iceland | Sigurdsson Vidir | Forlán Diego / Uruguay | Xavi / Spain | Villa David / Spain |
| India | Sarkar Dhiman | Xavi / Spain | Forlán Diego / Uruguay | Casillas Iker / Spain |
| Indonesia | Saleh Nurdin | Xavi / Spain | Sneijder Wesley / Netherlands | Forlán Diego / Uruguay |
| Iran | Rahmani Siamak | Xavi / Spain | Sneijder Wesley / Netherlands | Messi Lionel / Argentina |
| Iraq | Abdul Emam Sami | Iniesta Andrés / Spain | Alonso Xabi / Spain | Sneijder Wesley / Netherlands |
| Israel | Klieger / Jacobi Noah / Nadav | Xavi / Spain | Messi Lionel / Argentina | Sneijder Wesley / Netherlands |
| Italy | Condo Paolo | Sneijder Wesley / Netherlands | Iniesta Andrés / Spain | Müller Thomas / Germany |
| Jamaica | Boyd Audley | Xavi / Spain | Sneijder Wesley / Netherlands | Messi Lionel / Argentina |
| Japan | Tamura Shuichi | Xavi / Spain | Sneijder Wesley / Netherlands | Forlán Diego / Uruguay |
| Kazakhstan | Tulegenov Geniy | Forlán Diego / Uruguay | Iniesta Andrés / Spain | Sneijder Wesley / Netherlands |
| Kenya | Nyende Charles | Villa David / Spain | Iniesta Andrés / Spain | Eto'o Samuel / Cameroon |
| Korea DPR | Dong Gyu Ri | Casillas Iker / Spain | Iniesta Andrés / Spain | Müller Thomas / Germany |
| Korea Republic | Kim Hanseok | Iniesta Andrés / Spain | Villa David / Spain | Messi Lionel / Argentina |
| Kyrgyzstan | Louzanov Pavel | Iniesta Andrés / Spain | Cristiano Ronaldo / Portugal | Sneijder Wesley / Netherlands |
| Latvia | Karpoushkin Valery | Forlán Diego / Uruguay | Sneijder Wesley / Netherlands | Iniesta Andrés / Spain |
| Lebanon | Fawaz Mohamed | Xavi / Spain | Iniesta Andrés / Spain | Forlán Diego / Uruguay |
| Lesotho | Matjama Thabang | Sneijder Wesley / Netherlands | Robben Arjen / Netherlands | Schweinsteiger Bastian / Germany |
| Liberia | Carter J. Burgess | Sneijder Wesley / Netherlands | Casillas Iker / Spain | Messi Lionel / Argentina |
| Libya | Cutajar / Beilleid Hussein Joseph / Mjuftah | Iniesta Andrés / Spain | Casillas Iker / Spain | Forlán Diego / Uruguay |
| Liechtenstein | Hasler Ernst | Sneijder Wesley / Netherlands | Xavi / Spain | Villa David / Spain |
| Lithuania | Janonis Giedrius | Xavi / Spain | Sneijder Wesley / Netherlands | Forlán Diego / Uruguay |
| Luxembourg | Hiegel / Nadin Didier / Christophe | Iniesta Andrés / Spain | Sneijder Wesley / Netherlands | Messi Lionel / Argentina |
| Madagascar | Rabary Clément | Iniesta Andrés / Spain | Özil Mesut / Germany | Sneijder Wesley / Netherlands |
| Malawi | Kanjere Peter | Sneijder Wesley / Netherlands | Xavi / Spain | Forlán Diego / Uruguay |
| Malaysia | Hashim Rizal | Sneijder Wesley / Netherlands | Xavi / Spain | Forlán Diego / Uruguay |
| Maldives | Ali Shimaz | Messi Lionel / Argentina | Villa David / Spain | Drogba Didier / Cote d'Ivoire |
| Mali | Bobo Tounkara Souleymane | Iniesta Andrés / Spain | Sneijder Wesley / Netherlands | Forlán Diego / Uruguay |
| Malta | Camenzuli Charles | Messi Lionel / Argentina | Villa David / Spain | Sneijder Wesley / Netherlands |
| Mauritania | Ould El Hacen Mohamed | Özil Mesut / Germany | Villa David / Spain | Forlán Diego / Uruguay |
| Mauritius | Hydoo Azmaal | Xavi / Spain | Iniesta Andrés / Spain | Sneijder Wesley / Netherlands |

| Mexico | Aguilera Salvador | Iniesta Andrés / Spain | Messi Lionel / Argentina | Forlán Diego / Uruguay |
| --- | --- | --- | --- | --- |
| Moldova | Donets Sergei | Forlán Diego / Uruguay | Sneijder Wesley / Netherlands | Casillas Iker / Spain |
| Mongolia | Baljinnyam Erdenebat | Iniesta Andrés / Spain | Sneijder Wesley / Netherlands | Müller Thomas / Germany |
| Montenegro | Mitrovic Danilo | Messi Lionel / Argentina | Xavi / Spain | Sneijder Wesley / Netherlands |
| Morocco | Badri Mostafa | Sneijder Wesley / Netherlands | Xavi / Spain | Eto'o Samuel / Cameroon |
| Namibia | Nikodemus Sheefeni | Messi Lionel / Argentina | Iniesta Andrés / Spain | Eto'o Samuel / Cameroon |
| Netherlands | van den Nieuwenhof Frans | Iniesta Andrés / Spain | Xavi / Spain | Sneijder Wesley / Netherlands |
| New Caledonia | Chohin Christophe | Sneijder Wesley / Netherlands | Forlán Diego / Uruguay | Iniesta Andrés / Spain |
| New Zealand | Watson Gordon Glen | Sneijder Wesley / Netherlands | Xavi / Spain | Iniesta Andrés / Spain |
| Nicaragua | Rosales Cruz Osman | Iniesta Andrés / Spain | Messi Lionel / Argentina | Forlán Diego / Uruguay |
| Niger | Ganoua Mohamed Silimane | Sneijder Wesley / Netherlands | Iniesta Andrés / Spain | Forlán Diego / Uruguay |
| Nigeria | Audu Samm | Sneijder Wesley / Netherlands | Messi Lionel / Argentina | Gyan Asamoah / Ghana |
| Northern Ireland | Fullerton / Taggart Jackie / Joel | Xavi / Spain | Sneijder Wesley / Netherlands | Messi Lionel / Argentina |
| Norway | Sandven Arild | Messi Lionel / Argentina | Forlán Diego / Uruguay | Sneijder Wesley / Netherlands |
| Oman | Al-Barhi Saleh | Xavi / Spain | Iniesta Andrés / Spain | Casillas Iker / Spain |
| Palestine | Iraqi Mohamed | Sneijder Wesley / Netherlands | Xavi / Spain | Forlán Diego / Uruguay |
| Panama | Estrada Campo Elias | Forlán Diego / Uruguay | Iniesta Andrés / Spain | Messi Lionel / Argentina |
| Paraguay | Da Rosa Ruben Dario | Xavi / Spain | Messi Lionel / Argentina | Forlán Diego / Uruguay |
| Peru | Salinas Carlos | Forlán Diego / Uruguay | Sneijder Wesley / Netherlands | Iniesta Andrés / Spain |
| Poland | Adamczyk Zbigniew | Iniesta Andrés / Spain | Sneijder Wesley / Netherlands | Messi Lionel / Argentina |
| Portugal | Rita Joaquim | Xavi / Spain | Messi Lionel / Argentina | Sneijder Wesley / Netherlands |
| Puerto Rico | Arce Luis Santiago | Messi Lionel / Argentina | Cristiano Ronaldo / Portugal | Forlán Diego / Uruguay |
| Qatar | Alkhalifi Majed M. | Xavi / Spain | Iniesta Andrés / Spain | Forlán Diego / Uruguay |
| Republic of Ireland | Kelly / Magee Paul / Jimmy | Xavi / Spain | Sneijder Wesley / Netherlands | Messi Lionel / Argentina |
| Romania | Timofte Radu | Sneijder Wesley / Netherlands | Iniesta Andrés / Spain | Xavi / Spain |
| Russia | Kleshchev Konstantin | Sneijder Wesley / Netherlands | Xavi / Spain | Forlán Diego / Uruguay |
| Rwanda | Mugabe Bonnie | Iniesta Andrés / Spain | Sneijder Wesley / Netherlands | Gyan Asamoah / Ghana |
| San Marino | Zunino Marco | Robben Arjen / Netherlands | Özil Mesut / Germany | Iniesta Andrés / Spain |
| Saudi Arabia | Alsolami Rajallah | Iniesta Andrés / Spain | Sneijder Wesley / Netherlands | Messi Lionel / Argentina |
| Scotland | Greechan John | Xavi / Spain | Forlán Diego / Uruguay | Messi Lionel / Argentina |
| Senegal | Goloko Aliou | Iniesta Andrés / Spain | Sneijder Wesley / Netherlands | Gyan Asamoah / Ghana |
| Serbia | Velichkovic Jovan | Iniesta Andrés / Spain | Sneijder Wesley / Netherlands | Forlán Diego / Uruguay |
| Seychelles | Govinden Gérard | Iniesta Andrés / Spain | Sneijder Wesley / Netherlands | Forlán Diego / Uruguay |
| Sierra Leone | Fajah Barrie Mohamed | Sneijder Wesley / Netherlands | Messi Lionel / Argentina | Xavi / Spain |
| Slovakia | Surin Peter | Xavi / Spain | Messi Lionel / Argentina | Forlán Diego / Uruguay |
| Slovenia | Stare Andrej | Forlán Diego / Uruguay | Villa David / Spain | Sneijder Wesley / Netherlands |
| South Africa | Gleeson Mark | Xavi / Spain | Iniesta Andrés / Spain | Messi Lionel / Argentina |
| Spain | Aguilar Paco | Xavi / Spain | Messi Lionel / Argentina | Sneijder Wesley / Netherlands |
| Sudan | Abu Elgassim Muzamil | Iniesta Andrés / Spain | Messi Lionel / Argentina | Sneijder Wesley / Netherlands |
| Suriname | Romeo Desney | Sneijder Wesley / Netherlands | Iniesta Andrés / Spain | Xavi / Spain |
| Swaziland | Dlamini Kenneth | Messi Lionel / Argentina | Forlán Diego / Uruguay | Sneijder Wesley / Netherlands |
| Sweden | Ysten Henrik | Sneijder Wesley / Netherlands | Xavi / Spain | Forlán Diego / Uruguay |
| Switzerland | Dupuis Pierre-Alain | Iniesta Andrés / Spain | Sneijder Wesley / Netherlands | Forlán Diego / Uruguay |
| Tahiti | Huc Olivier | Messi Lionel / Argentina | Iniesta Andrés / Spain | Drogba Didier / Cote d'Ivoire |
| Tajikistan | Bouriev Alaveddine | Messi Lionel / Argentina | Sneijder Wesley / Netherlands | Casillas Iker / Spain |
| Tanzania | Wambura Boniface | Villa David / Spain | Sneijder Wesley / Netherlands | Gyan Asamoah / Ghana |
| Thailand | Patoommawatana Urai | Sneijder Wesley / Netherlands | Xavi / Spain | Iniesta Andrés / Spain |

| Togo | Ayena Mathias | Iniesta Andrés / Spain | Sneijder Wesley / Netherlands | Gyan Asamoah / Ghana |
| --- | --- | --- | --- | --- |
| Trinidad and Tobago | Liburd Lasana | Messi Lionel / Argentina | Sneijder Wesley / Netherlands | Robben Arjen / Netherlands |
| Tunisia | Dhaifallah Abdesslam | Iniesta Andrés / Spain | Messi Lionel / Argentina | Sneijder Wesley / Netherlands |
| Turkey | Manav Selçuk | Xavi / Spain | Iniesta Andrés / Spain | Özil Mesut / Germany |
| Turkmenistan | Vershinin Alexander | Iniesta Andrés / Spain | Messi Lionel / Argentina | Xavi / Spain |
| Uganda | Musisi Kiyingi Fred | Iniesta Andrés / Spain | Forlán Diego / Uruguay | Sneijder Wesley / Netherlands |
| Ukraine | Linnyk Igor | Xavi / Spain | Sneijder Wesley / Netherlands | Messi Lionel / Argentina |
| United Arab Emirates | Mouadhen Dafrallah | Iniesta Andrés / Spain | Xavi / Spain | Sneijder Wesley / Netherlands |
| Uruguay | Pineyrua Ricardo | Messi Lionel / Argentina | Forlán Diego / Uruguay | Iniesta Andrés / Spain |
| USA | Kennedy Paul | Xavi / Spain | Messi Lionel / Argentina | Sneijder Wesley / Netherlands |
| Uzbekistan | Rtveladze Grigory | Sneijder Wesley / Netherlands | Forlán Diego / Uruguay | Villa David / Spain |
| Venezuela | Blavia Francisco | Xavi / Spain | Messi Lionel / Argentina | Forlán Diego / Uruguay |
| Vietnam | Anh Ngoc Truong | Xavi / Spain | Sneijder Wesley / Netherlands | Forlán Diego / Uruguay |
| Wales | Abbandonato Paul | Messi Lionel / Argentina | Villa David / Spain | Alonso Xabi / Spain |
| Zambia | Lungu Chapadongo | Iniesta Andrés / Spain | Forlán Diego / Uruguay | Messi Lionel / Argentina |
| Zimbabwe | Mabika Charles | Casillas Iker / Spain | Sneijder Wesley / Netherlands | Iniesta Andrés / Spain |
|  |  |  |  |  |

**FIFA BALLON D'OR 2011**

| **Vote** | **Country** | **Name** | **First (5 points)** | **Second (3 points)** | **Third (1 point)** |
| --- | --- | --- | --- | --- | --- |
| Captain | Albania | Lorik Cana | Messi Lionel | Cristiano Ronaldo | Xavi |
| Captain | Algeria | Antar Yahia | Messi Lionel | Cristiano Ronaldo | Müller Thomas |
| Captain | Andorra | Óscar Sonejee | Messi Lionel | Dani Alves | Xavi |
| Captain | Argentina | Lionel Messi | Xavi | Iniesta Andrés | Agüero Sergio |
| Captain | Armenia | Sargis Hovsepyan | Messi Lionel | Cristiano Ronaldo | Iniesta Andrés |
| Captain | Australia | Lucas Edward Neill | Cristiano Ronaldo | Iniesta Andrés | Schweinsteiger Bastian |
| Captain | Austria | Marc Janko | Iniesta Andrés | Cristiano Ronaldo | Xavi |
| Captain | Azerbaijan | Rashad Sadygov | Messi Lionel | Cristiano Ronaldo | Özil Mesut |
| Captain | Bangladesh | Biplob Bhattacharjee | Messi Lionel | Xavi | Cristiano Ronaldo |
| Captain | Belarus | Yuri Zhevnov | Cristiano Ronaldo | Messi Lionel | Iniesta Andrés |
| Captain | Belgium | Timmy Simons | Iniesta Andrés | Messi Lionel | Cristiano Ronaldo |
| Captain | Bermuda | Khano Smith | Messi Lionel | Cristiano Ronaldo | Piqué Gerard |
| Captain | Bhutan | Pema | Cristiano Ronaldo | Xabi Alonso | Messi Lionel |
| Captain | Bolivia | Ronald Raldes B. | Messi Lionel | Cristiano Ronaldo | Suárez Luis |
| Captain | Bosnia-Herzegovina | Emir Spahic | Xavi | Messi Lionel | Cristiano Ronaldo |
| Captain | Brazil | Thiago Emiliano Da Silva | Cristiano Ronaldo | Messi Lionel | Neymar |
| Captain | British Virgin Islands | Andy Davis | Fàbregas Cesc | Messi Lionel | Cristiano Ronaldo |
| Captain | Brunei Darussalam | Azwan Mohd Salleh | Messi Lionel | Xavi | Iniesta Andrés |
| Captain | Bulgaria | Stiliyan Petrov | Cristiano Ronaldo | Messi Lionel | Xavi |
| Captain | Burkina Faso | Mahamoudou Kere | Eto'o Samuel | Messi Lionel | Schweinsteiger Bastian |
| Captain | Burundi | Nahayo | Neymar | Abidal Éric | Benzema Karim |
| Captain | Cameroon | Samuel Eto'o | Messi Lionel | Xavi | Iniesta Andrés |
| Captain | Canada | Dwayne De Rosario | Messi Lionel | Iniesta Andrés | Xavi |
| Captain | Cape Verde Islands | Claudio Aguiar | Messi Lionel | Cristiano Ronaldo | Xavi |
| Captain | Cayman Islands | Ian Lindo | Messi Lionel | Cristiano Ronaldo | Neymar |
| Captain | Chad | Mahamat Habib Saleh | Cristiano Ronaldo | Xabi Alonso | Iniesta Andrés |
| Captain | Chile | Claudio Bravo | Messi Lionel | Iniesta Andrés | Cristiano Ronaldo |
| Captain | China PR | Ll Weifeng | Casillas Iker | Xavi | Iniesta Andrés |
| Captain | Chinese Taipei | Chen Po-Liang | Messi Lionel | Cristiano Ronaldo | Xavi |
| Captain | Colombia | Mario Yepes | Messi Lionel | Iniesta Andrés | Cristiano Ronaldo |
| Captain | Congo | Barel Mouko | Cristiano Ronaldo | Messi Lionel | Rooney Wayne |
| Captain | Cook Islands | Tony Jamieson | Messi Lionel | Casillas Iker | Xavi |
| Captain | Costa Rica | Bryan Ruiz | Iniesta Andrés | Messi Lionel | Cristiano Ronaldo |
| Captain | Croatia | Darijo Srna | Messi Lionel | Cristiano Ronaldo | Rooney Wayne |

| **Vote** | **Country** | **Name** | **First (5 points)** | **Second (3 points)** | **Third (1 point)** |
| --- | --- | --- | --- | --- | --- |
| Captain | Cuba | Yenier Marquez | Messi Lionel | Cristiano Ronaldo | Casillas Iker |
| Captain | Cyprus | Giannakis 'Ioannis' Okkas | Messi Lionel | Cristiano Ronaldo | Rooney Wayne |
| Captain | Czech Republic | Tomas Rosicky | Messi Lionel | Cristiano Ronaldo | Iniesta Andrés |
| Captain | Denmark | Christian Poulsen | Messi Lionel | Cristiano Ronaldo | Suárez Luis |
| Captain | Djibouti | Mohamed Kadar Daher | Messi Lionel | Cristiano Ronaldo | Piqué Gerard |
| Captain | Dominican Republic | Kervin De Jesus Severino | Messi Lionel | Sneijder Wesley | Xavi |
| Captain | Ecuador | Walter Ayovi | Messi Lionel | Cristiano Ronaldo | Xavi |
| Captain | Egypt | Ahmed Hassan | Messi Lionel | Cristiano Ronaldo | Eto'o Samuel |
| Captain | El Salvador | Luis Alonso Anaya | Messi Lionel | Cristiano Ronaldo | Xavi |
| Captain | England | John Terry | Xavi | Messi Lionel | Rooney Wayne |
| Captain | Eritrea | Daniel Gordon | Messi Lionel | Cristiano Ronaldo | Schweinsteiger Bastian |
| Captain | Estonia | Raio Piiroja | Casillas Iker | Villa David | Forlán Diego |
| Captain | Ethiopia | Degu Debebe | Xavi | Cristiano Ronaldo | Iniesta Andrés |
| Captain | Faroe Islands | Fróði Benjaminsen | Messi Lionel | Cristiano Ronaldo | Rooney Wayne |
| Captain | Finland | Niklas Moisander | Messi Lionel | Cristiano Ronaldo | Xavi |
| Captain | France | Hugo Lloris | Messi Lionel | Cristiano Ronaldo | Casillas Iker |
| Captain | FYR Macedonia | Goran Pandev | Eto'o Samuel | Sneijder Wesley | Abidal Éric |
| Captain | Georgia | Kakha Kaladze | Messi Lionel | Cristiano Ronaldo | Sneijder Wesley |
| Captain | Germany | Philipp Lahm | Messi Lionel | Xavi | Cristiano Ronaldo |
| Captain | Ghana | John Mensah | Eto'o Samuel | Messi Lionel | Iniesta Andrés |
| Captain | Greece | Giorgos Karagounis | Cristiano Ronaldo | Suárez Luis | Iniesta Andrés |
| Captain | Guam | Dominic Gadia | Xavi | Casillas Iker | Messi Lionel |
| Captain | Guinea | Kamil Zayatte | Messi Lionel | Eto'o Samuel | Sneijder Wesley |
| Captain | Guinea-Bissau | Bruno Fernandes | Messi Lionel | Cristiano Ronaldo | Xavi |
| Captain | Honduras | Noel Valladares | Messi Lionel | Cristiano Ronaldo | Iniesta Andrés |
| Captain | Hong Kong | Chan Wai Ho | Iniesta Andrés | Xavi | Messi Lionel |
| Captain | Hungary | Zoltán Gera | Xavi | Cristiano Ronaldo | Iniesta Andrés |
| Captain | Iceland | Hermann Hreiðarsson | Messi Lionel | Cristiano Ronaldo | Iniesta Andrés |
| Captain | India | Climax Lawrence | Xavi | Messi Lionel | Cristiano Ronaldo |
| Captain | Indonesia | Bambang Pamungkas | Messi Lionel | Cristiano Ronaldo | Iniesta Andrés |
| Captain | Iran | Javad Nekounam | Messi Lionel | Xavi | Iniesta Andrés |
| Captain | Israel | Yossi Benayoun | Messi Lionel | Cristiano Ronaldo | Xavi |
| Captain | Italy | Gianluigi Buffon | Messi Lionel | Rooney Wayne | Iniesta Andrés |
| Captain | Jamaica | Shavar Thomas | Cristiano Ronaldo | Messi Lionel | Forlán Diego |
| Captain | Japan | Makoto Hasebe | Messi Lionel | Dani Alves | Xavi |
| Captain | Jordan | Hatem Aqel | Messi Lionel | Cristiano Ronaldo | Iniesta Andrés |

| **Vote** | **Country** | **Name** | **First (5 points)** | **Second (3 points)** | **Third (1 point)** |
| --- | --- | --- | --- | --- | --- |
| Captain | Kazakhstan | Kairat Nurdauletov | Messi Lionel | Xavi | Cristiano Ronaldo |
| Captain | Kyrgyzstan | Azamat Baimatov | Cristiano Ronaldo | Messi Lionel | Xabi Alonso |
| Captain | Laos | Saynakhongvieng Phommapanya | Xavi | Messi Lionel | Piqué Gerard |
| Captain | Latvia | Kaspars Gorkšs | Messi Lionel | Iniesta Andrés | Cristiano Ronaldo |
| Captain | Lebanon | Roda Antar | Messi Lionel | Xabi Alonso | Rooney Wayne |
| Captain | Liechtenstein | Mario Frick | Messi Lionel | Xavi | Cristiano Ronaldo |
| Captain | Lithuania | Tomas Danilevicius | Messi Lionel | Iniesta Andrés | Cristiano Ronaldo |
| Captain | Luxembourg | René Peters | Messi Lionel | Xavi | Iniesta Andrés |
| Captain | Macau | Geofredo De Sousa Cheung | Casillas Iker | Cristiano Ronaldo | Messi Lionel |
| Captain | Madagascar | Johan Paul | Messi Lionel | Cristiano Ronaldo | Xavi |
| Captain | Malawi | Peter Mponda | Messi Lionel | Cristiano Ronaldo | Xavi |
| Captain | Malaysia | Safiq Rahim | Xavi | Messi Lionel | Iniesta Andrés |
| Captain | Maldives | Ali Ashfaq | Messi Lionel | Iniesta Andrés | Xavi |
| Captain | Malta | Michael Mifsud | Xavi | Rooney Wayne | Messi Lionel |
| Captain | Mauritius | Colin Bell | Messi Lionel | Xavi | Cristiano Ronaldo |
| Captain | Mexico | Gerardo Torrado | Xavi | Messi Lionel | Özil Mesut |
| Captain | Moldova | Epureanu Alexandru | Messi Lionel | Cristiano Ronaldo | Xavi |
| Captain | Mongolia | Donorov Lumbengarav | Messi Lionel | Villa David | Müller Thomas |
| Captain | Montserrat | Anthony Griffith | Messi Lionel | Cristiano Ronaldo | Xavi |
| Captain | Morocco | Houssine Kharja | Cristiano Ronaldo | Messi Lionel | Fàbregas Cesc |
| Captain | Myanmar | Aye San | Messi Lionel | Cristiano Ronaldo | Iniesta Andrés |
| Captain | Netherlands | Mark Van Bommel | Sneijder Wesley | Messi Lionel | Cristiano Ronaldo |
| Captain | New Caledonia | Wajoka Pierre | Messi Lionel | Iniesta Andrés | Cristiano Ronaldo |
| Captain | New Zealand | Tim Brown | Agüero Sergio | Xabi Alonso | Rooney Wayne |
| Captain | Nicaragua | David Solorzano | Cristiano Ronaldo | Özil Mesut | Forlán Diego |
| Captain | Nigeria | Joseph Yobo | Messi Lionel | Cristiano Ronaldo | Rooney Wayne |
| Captain | Northern Ireland | Aaron Hughes | Messi Lionel | no vote | no vote |
| Captain | Norway | Brede Hangeland | Messi Lionel | Iniesta Andrés | Suárez Luis |
| Captain | Pakistan | Jaffar Khan | Müller Thomas | Iniesta Andrés | Schweinsteiger Bastian |
| Captain | Palestine | Ramzi Saleh | Cristiano Ronaldo | Messi Lionel | Casillas Iker |
| Captain | Panama | Felipe Baloy | Messi Lionel | Xavi | Cristiano Ronaldo |
| Captain | Papua New Guinea | David Muta | Messi Lionel | Xavi | Fàbregas Cesc |
| Captain | Paraguay | Justo Villar | Messi Lionel | Suárez Luis | Xavi |
| Captain | Peru | Claudio Pizarro | Messi Lionel | Cristiano Ronaldo | Iniesta Andrés |
| Captain | Philippines | Alexander Charles Borromeo | Messi Lionel | Cristiano Ronaldo | Rooney Wayne |
| Captain | Poland | Jakub Blaszczykowski | Messi Lionel | Cristiano Ronaldo | Xavi |

| **Vote** | **Country** | **Name** | **First (5 points)** | **Second (3 points)** | **Third (1 point)** |
| --- | --- | --- | --- | --- | --- |
| Captain | Portugal | Nuno Gomes | Cristiano Ronaldo | Nani | Neymar |
| Captain | Puerto Rico | Marco Velez | Messi Lionel | Cristiano Ronaldo | Iniesta Andrés |
| Captain | Qatar | Bilal Rajab | Messi Lionel | no vote | no vote |
| Captain | Republic of Ireland | Robbie Keane | Iniesta Andrés | Messi Lionel | Sneijder Wesley |
| Captain | Romania | Razvan Rat | Messi Lionel | Iniesta Andrés | Cristiano Ronaldo |
| Captain | Russia | Andrey Arshavin | Messi Lionel | Cristiano Ronaldo | Fàbregas Cesc |
| Captain | Rwanda | Olivier Karekezi | Messi Lionel | Iniesta Andrés | Xavi |
| Captain | San Marino | Andy Selva | Messi Lionel | Iniesta Andrés | Cristiano Ronaldo |
| Captain | São Tomé e Príncipe | D. Das Neves | Messi Lionel | Eto'o Samuel | Xavi |
| Captain | Saudi Arabia | Mohammed Noor | Cristiano Ronaldo | Eto'o Samuel | Messi Lionel |
| Captain | Scotland | Darren Fletcher | Messi Lionel | Cristiano Ronaldo | Xavi |
| Captain | Senegal | Mamadou Niang | Messi Lionel | Cristiano Ronaldo | Iniesta Andrés |
| Captain | Serbia | Nikola Zigic | Messi Lionel | Iniesta Andrés | Cristiano Ronaldo |
| Captain | Seychelles | Don Anacoura | Messi Lionel | Cristiano Ronaldo | Özil Mesut |
| Captain | Singapore | Mohd Shahril Ishak | Messi Lionel | Cristiano Ronaldo | Agüero Sergio |
| Captain | Slovakia | Marek Hamsík | Messi Lionel | Cristiano Ronaldo | Iniesta Andrés |
| Captain | Slovenia | Samir Handanovic | Cristiano Ronaldo | Messi Lionel | Xavi |
| Captain | Solomon Islands | Henry Fa'Arodo | Messi Lionel | Forlán Diego | Abidal Éric |
| Captain | Somalia | Yasin A. Igal | Messi Lionel | Xavi | Iniesta Andrés |
| Captain | South Africa | Steven Pienaar | Messi Lionel | Cristiano Ronaldo | Neymar |
| Captain | Spain | Iker Casillas | Cristiano Ronaldo | Özil Mesut | Messi Lionel |
| Captain | Sri Lanka | Rohana Ruwanthilake | Messi Lionel | Fàbregas Cesc | Cristiano Ronaldo |
| Captain | St. Lucia | Guy George | Messi Lionel | invalid vote | invalid vote |
| Captain | Sudan | Haitham Mustafa | Messi Lionel | Cristiano Ronaldo | Iniesta Andrés |
| Captain | Swaziland | Siyabonga Mdluli | Cristiano Ronaldo | Messi Lionel | Villa David |
| Captain | Sweden | Zlatan Ibrahimovic | Messi Lionel | Rooney Wayne | Iniesta Andrés |
| Captain | Switzerland | Gökhan Inler | Messi Lionel | Cristiano Ronaldo | Xavi |
| Captain | Tahiti | Samin X. | Messi Lionel | Cristiano Ronaldo | Casillas Iker |
| Captain | Tajikistan | Khurshed Mahmudov | Messi Lionel | Cristiano Ronaldo | Xavi |
| Captain | Tanzania | Henri Joseph | Messi Lionel | Cristiano Ronaldo | Xavi |
| Captain | Thailand | Datsakorn Thonglao | Messi Lionel | Cristiano Ronaldo | Rooney Wayne |
| Captain | Tonga | Folio Moeaki | Dani Alves | Agüero Sergio | Rooney Wayne |
| Captain | Trinidad and Tobago | Kenwyne Jones | Messi Lionel | Cristiano Ronaldo | Xavi |
| Captain | Tunisia | Karim Hagui | Messi Lionel | Xavi | Cristiano Ronaldo |
| Captain | Turkey | Hamit Altintop | Cristiano Ronaldo | Messi Lionel | Özil Mesut |
| Captain | Turks and Caicos Islands | Philip Shearer | Messi Lionel | Cristiano Ronaldo | Xavi |

| **Vote** | **Country** | **Name** | **First (5 points)** | **Second (3 points)** | **Third (1 point)** |
| --- | --- | --- | --- | --- | --- |
| Captain | Ukraine | Andriy Shevchenko | Messi Lionel | Cristiano Ronaldo | Xavi |
| Captain | Uruguay | Diego Lugano | Messi Lionel | Cristiano Ronaldo | Xavi |
| Captain | US Virgin Islands | Reid Klopp | Messi Lionel | Cristiano Ronaldo | Iniesta Andrés |
| Captain | USA | Carlos Bocanegra | Messi Lionel | Xavi | Cristiano Ronaldo |
| Captain | Uzbekistan | Server Djeparov | Messi Lionel | Xavi | Cristiano Ronaldo |
| Captain | Vanuatu | Robert Yelou | Messi Lionel | Schweinsteiger Bastian | Sneijder Wesley |
| Captain | Venezuela | Juan Arango | Iniesta Andrés | Xavi | Forlán Diego |
| Captain | Vietnam | Nguyen Thanh Luang | Messi Lionel | Cristiano Ronaldo | Rooney Wayne |
| Captain | Wales | Aaron Ransey | Messi Lionel | Cristiano Ronaldo | Xavi |
| Captain | Yemen | Moad Abdulkhlek | Iniesta Andrés | Messi Lionel | Müller Thomas |
| Captain | Zimbabwe | Tinashe Nengomasha | Iniesta Andrés | Rooney Wayne | Benzema Karim |
| Coach | Albania | Dzemal Mustedanagic | Cristiano Ronaldo | Abidal Éric | Messi Lionel |
| Coach | Algeria | Vahid Halilhodzic | Messi Lionel | Cristiano Ronaldo | Özil Mesut |
| Coach | Andorra | Koldo Álvarez | Messi Lionel | Cristiano Ronaldo | Xavi |
| Coach | Argentina | Alejandro Sabella | Messi Lionel | Xavi | Iniesta Andrés |
| Coach | Armenia | Vardan Minasyan | Messi Lionel | Iniesta Andrés | Cristiano Ronaldo |
| Coach | Australia | Holger Osieck | Messi Lionel | Schweinsteiger Bastian | Cristiano Ronaldo |
| Coach | Austria | Marcel Koller | Iniesta Andrés | Messi Lionel | Schweinsteiger Bastian |
| Coach | Azerbaijan | Hans Hubert Vogts | Messi Lionel | Schweinsteiger Bastian | Rooney Wayne |
| Coach | Bangladesh | Nikola Ilievski | Messi Lionel | Cristiano Ronaldo | Casillas Iker |
| Coach | Belarus | Bernd Stange | Messi Lionel | Xavi | Rooney Wayne |
| Coach | Belgium | Georges Leekens | Messi Lionel | Cristiano Ronaldo | Iniesta Andrés |
| Coach | Bermuda | Maurice Lowe | Messi Lionel | Cristiano Ronaldo | Xavi |
| Coach | Bhutan | Hiroaki Matsuyama | Iniesta Andrés | Müller Thomas | Neymar |
| Coach | Bolivia | Gustavo Quinteros | Messi Lionel | Cristiano Ronaldo | Suárez Luis |
| Coach | Bosnia-Herzegovina | Safet Sušic | Messi Lionel | Cristiano Ronaldo | Casillas Iker |
| Coach | Brazil | Mano Menezes | Messi Lionel | Cristiano Ronaldo | Neymar |
| Coach | British Virgin Islands | Avondale Williams | Messi Lionel | Cristiano Ronaldo | Xavi |
| Coach | Brunei Darussalam | Dayem Haji Ali | Messi Lionel | Cristiano Ronaldo | Agüero Sergio |
| Coach | Bulgaria | Luboslav Penev | Cristiano Ronaldo | Messi Lionel | Iniesta Andrés |
| Coach | Burkina Faso | Paulo Duarte | Cristiano Ronaldo | Messi Lionel | Eto'o Samuel |
| Coach | Burundi | Adel Amrouche | Benzema Karim | Özil Mesut | Eto'o Samuel |
| Coach | Cameroon | Denis Lavagne | Eto'o Samuel | Messi Lionel | Iniesta Andrés |
| Coach | Canada | Stephen Hart | Messi Lionel | Cristiano Ronaldo | Iniesta Andrés |
| Coach | Cape Verde Islands | Lucio Antunes | Messi Lionel | Cristiano Ronaldo | Nani |
| Coach | Cayman Islands | Carl Brown | Messi Lionel | Cristiano Ronaldo | Rooney Wayne |

| **Vote** | **Country** | **Name** | **First (5 points)** | **Second (3 points)** | **Third (1 point)** |
| --- | --- | --- | --- | --- | --- |
| Coach | Chad | Mahamat Oumar | Messi Lionel | Cristiano Ronaldo | Forlán Diego |
| Coach | Chile | Claudio Borghi | Iniesta Andrés | Messi Lionel | Xavi |
| Coach | China PR | Jose Antonio Camacho | Casillas Iker | Xavi | Iniesta Andrés |
| Coach | Chinese Taipei | Lee Tae-Ho | Messi Lionel | Cristiano Ronaldo | Rooney Wayne |
| Coach | Colombia | Leonel Alvarez | Cristiano Ronaldo | Messi Lionel | Xavi |
| Coach | Congo | Noel Minga | Messi Lionel | Rooney Wayne | Cristiano Ronaldo |
| Coach | Congo DR | Claude Le Roy | Messi Lionel | Cristiano Ronaldo | Abidal Éric |
| Coach | Cook Islands | Shane Rufer | Cristiano Ronaldo | Messi Lionel | Eto'o Samuel |
| Coach | Costa Rica | Jorge Luis Pinto | Messi Lionel | Cristiano Ronaldo | Iniesta Andrés |
| Coach | Croatia | Slaven Bilic | Messi Lionel | Xavi | Cristiano Ronaldo |
| Coach | Cuba | Raul Gonzales Triana | Messi Lionel | Cristiano Ronaldo | Casillas Iker |
| Coach | Cyprus | Nikos Nioplias | Messi Lionel | Cristiano Ronaldo | Rooney Wayne |
| Coach | Czech Republic | Michal Bilek | Messi Lionel | Cristiano Ronaldo | Iniesta Andrés |
| Coach | Denmark | Morten Olsen | Xavi | Messi Lionel | Iniesta Andrés |
| Coach | Djibouti | Omar Mahfoud | Messi Lionel | Cristiano Ronaldo | Xavi |
| Coach | Dominican Republic | Clemente Domingo Hernandez | Messi Lionel | Forlán Diego | Xavi |
| Coach | Ecuador | Reinaldo Rueda | Messi Lionel | Xavi | Forlán Diego |
| Coach | Egypt | Fathy Nossein | Messi Lionel | Cristiano Ronaldo | Eto'o Samuel |
| Coach | El Salvador | Ruben Israel | Forlán Diego | Messi Lionel | Suárez Luis |
| Coach | England | Fabio Capello | Messi Lionel | Cristiano Ronaldo | Rooney Wayne |
| Coach | Eritrea | Negash Teklit | Messi Lionel | Cristiano Ronaldo | Rooney Wayne |
| Coach | Estonia | Tarmo Rüütli | Messi Lionel | Cristiano Ronaldo | Rooney Wayne |
| Coach | Ethiopia | Sewnet Bishaw | Messi Lionel | Cristiano Ronaldo | Iniesta Andrés |
| Coach | Faroe Islands | Lars Olsen | Messi Lionel | Cristiano Ronaldo | Rooney Wayne |
| Coach | Finland | Mixu Paatelainen | Messi Lionel | Cristiano Ronaldo | Xavi |
| Coach | France | Laurent Blanc | Messi Lionel | Piqué Gerard | Forlán Diego |
| Coach | FYR Macedonia | John Toshack | Xavi | Cristiano Ronaldo | Eto'o Samuel |
| Coach | Georgia | Timour Ketsbaia | Messi Lionel | Cristiano Ronaldo | Rooney Wayne |
| Coach | Germany | Joachim Loew | Xavi | Messi Lionel | Iniesta Andrés |
| Coach | Ghana | Goran Stevanovic | Messi Lionel | Cristiano Ronaldo | Iniesta Andrés |
| Coach | Greece | Fernando Santos | Cristiano Ronaldo | Messi Lionel | Rooney Wayne |
| Coach | Guinea | Michel Dussuyer | Messi Lionel | Cristiano Ronaldo | Eto'o Samuel |
| Coach | Guinea-Bissau | Luis Norton De Matos | Cristiano Ronaldo | Messi Lionel | Rooney Wayne |
| Coach | Honduras | Luis Fernando Suárez | Messi Lionel | Cristiano Ronaldo | Iniesta Andrés |
| Coach | Hong Kong | Liu Chun Fai | Messi Lionel | Cristiano Ronaldo | Casillas Iker |
| Coach | Hungary | Sándor Egervári | Messi Lionel | Xavi | Özil Mesut |

| **Vote** | **Country** | **Name** | **First (5 points)** | **Second (3 points)** | **Third (1 point)** |
| --- | --- | --- | --- | --- | --- |
| Coach | Iceland | Ólafur Jóhannesson | Messi Lionel | Cristiano Ronaldo | Xavi |
| Coach | India | Savio Medeira | Messi Lionel | Xavi | Cristiano Ronaldo |
| Coach | Indonesia | Wim Rijsbergen | Messi Lionel | Cristiano Ronaldo | Özil Mesut |
| Coach | Iran | Carlos Queiroz | Messi Lionel | Cristiano Ronaldo | Xavi |
| Coach | Israel | Luis Fernandez | Messi Lionel | Cristiano Ronaldo | Rooney Wayne |
| Coach | Italy | Cesare Prandelli | Messi Lionel | Müller Thomas | Schweinsteiger Bastian |
| Coach | Jamaica | Theodore Whitmore | Messi Lionel | Cristiano Ronaldo | Xavi |
| Coach | Japan | Alberto Zaccheroni | Iniesta Andrés | Cristiano Ronaldo | Messi Lionel |
| Coach | Jordan | Adnan Hamad | Messi Lionel | Cristiano Ronaldo | Xavi |
| Coach | Kazakhstan | Miroslav Beranek | Messi Lionel | Cristiano Ronaldo | Xavi |
| Coach | Kyrgyzstan | Murat Djumakeev | Messi Lionel | Schweinsteiger Bastian | Xabi Alonso |
| Coach | Laos | Hans-Peter Schaller | Xavi | Messi Lionel | Schweinsteiger Bastian |
| Coach | Latvia | Aleksandrs Starkovs | Messi Lionel | Cristiano Ronaldo | Villa David |
| Coach | Lebanon | Theo Buecker | Messi Lionel | Xabi Alonso | Müller Thomas |
| Coach | Liberia | Roberto Landi | Messi Lionel | Iniesta Andrés | Agüero Sergio |
| Coach | Liechtenstein | Bidu Zaugg | Messi Lionel | Cristiano Ronaldo | Xavi |
| Coach | Lithuania | R. Zutautas | Messi Lionel | Cristiano Ronaldo | Xavi |
| Coach | Luxembourg | Luc Holtz | Messi Lionel | Xavi | Cristiano Ronaldo |
| Coach | Macau | Leung Sui Wing | Casillas Iker | Cristiano Ronaldo | Messi Lionel |
| Coach | Madagascar | Franck Rajaonarisamba | Messi Lionel | Xavi | Cristiano Ronaldo |
| Coach | Malawi | Kinnah Phiri | Messi Lionel | Xavi | Cristiano Ronaldo |
| Coach | Malaysia | Rajagobal Krishnasamy | Messi Lionel | Cristiano Ronaldo | Suárez Luis |
| Coach | Maldives | Istvan Urbanyi | Messi Lionel | Rooney Wayne | Iniesta Andrés |
| Coach | Malta | Robert Gatt | Xavi | Messi Lionel | Cristiano Ronaldo |
| Coach | Mauritania | Birama Gaye | Messi Lionel | Cristiano Ronaldo | Forlán Diego |
| Coach | Mauritius | Akbar Patel | Messi Lionel | Xavi | Müller Thomas |
| Coach | Mexico | José Manuel De La Torre | Messi Lionel | Xavi | Rooney Wayne |
| Coach | Moldova | Gavril Balint | Messi Lionel | Cristiano Ronaldo | Sneijder Wesley |
| Coach | Mongolia | Erdenebat Sandagdorj | Messi Lionel | Xavi | Cristiano Ronaldo |
| Coach | Montserrat | Kenneth Dyer | Messi Lionel | Cristiano Ronaldo | Villa David |
| Coach | Morocco | Eric Gerets | Messi Lionel | Cristiano Ronaldo | Xavi |
| Coach | Mozambique | Gert Engels | Messi Lionel | Cristiano Ronaldo | Müller Thomas |
| Coach | Myanmar | Stefan Hansson | Messi Lionel | Cristiano Ronaldo | Iniesta Andrés |
| Coach | Netherlands | Bert Van Marwijk | Messi Lionel | Sneijder Wesley | Özil Mesut |
| Coach | New Caledonia | Christophe Coursimault | Messi Lionel | Iniesta Andrés | Cristiano Ronaldo |
| Coach | New Zealand | Ricki Herbert | Messi Lionel | Müller Thomas | Suárez Luis |

| **Vote** | **Country** | **Name** | **First (5 points)** | **Second (3 points)** | **Third (1 point)** |
| --- | --- | --- | --- | --- | --- |
| Coach | Nicaragua | Enrique Llena | Messi Lionel | Özil Mesut | Iniesta Andrés |
| Coach | Nigeria | Stephen Keshi | Cristiano Ronaldo | Eto'o Samuel | Suárez Luis |
| Coach | Northern Ireland | Nigel Worthington | Messi Lionel | Cristiano Ronaldo | Rooney Wayne |
| Coach | Norway | Egil Olsen | Messi Lionel | Rooney Wayne | Suárez Luis |
| Coach | Pakistan | Zavisa Milosavljevic | Rooney Wayne | Messi Lionel | Casillas Iker |
| Coach | Palestine | Jamal Mahmoud | Casillas Iker | Sneijder Wesley | Müller Thomas |
| Coach | Panama | Julio Cesar Dely | Messi Lionel | Xavi | Forlán Diego |
| Coach | Papua New Guinea | Frank Farina | Messi Lionel | Suárez Luis | Sneijder Wesley |
| Coach | Paraguay | Francisco Arce | Messi Lionel | Özil Mesut | Cristiano Ronaldo |
| Coach | Peru | Sergio Markarian | Messi Lionel | Forlán Diego | Suárez Luis |
| Coach | Philippines | Hans Michael Weiss | Özil Mesut | Messi Lionel | Müller Thomas |
| Coach | Poland | Franciszek Smuda | Messi Lionel | Xavi | Iniesta Andrés |
| Coach | Portugal | Paulo Bento | Cristiano Ronaldo | Nani | Messi Lionel |
| Coach | Puerto Rico | Jeaustin Campos | Cristiano Ronaldo | Messi Lionel | Xavi |
| Coach | Qatar | Sebastiao Lazaroni | Messi Lionel | Neymar | Iniesta Andrés |
| Coach | Republic of Ireland | Giovanni Trapattoni | Xavi | Rooney Wayne | Iniesta Andrés |
| Coach | Romania | Victor Piturca | Messi Lionel | Cristiano Ronaldo | Xavi |
| Coach | Russia | Dick Advocaat | Messi Lionel | Sneijder Wesley | Xavi |
| Coach | Rwanda | Milutin Sredojevic | Messi Lionel | Cristiano Ronaldo | Neymar |
| Coach | San Marino | Mazza Giampaolo | Messi Lionel | Cristiano Ronaldo | Eto'o Samuel |
| Coach | São Tomé e Príncipe | Gustave Nyoumba | Messi Lionel | Eto'o Samuel | Iniesta Andrés |
| Coach | Saudi Arabia | Frank Rijkaard | Messi Lionel | Iniesta Andrés | Xavi |
| Coach | Scotland | Craig Levein | Messi Lionel | Iniesta Andrés | Cristiano Ronaldo |
| Coach | Senegal | Amara Traore | Eto'o Samuel | Messi Lionel | Iniesta Andrés |
| Coach | Serbia | Radovan Curcic | Messi Lionel | Xavi | Cristiano Ronaldo |
| Coach | Seychelles | Ralph Jean-Louis | Messi Lionel | Neymar | Özil Mesut |
| Coach | Singapore | Radojko Avramovic | Iniesta Andrés | Messi Lionel | Forlán Diego |
| Coach | Slovakia | Vladimir Weiss | Messi Lionel | Cristiano Ronaldo | Iniesta Andrés |
| Coach | Slovenia | Slavisa Stojanovic | Messi Lionel | Cristiano Ronaldo | Rooney Wayne |
| Coach | Solomon Islands | Jacob Moli | Iniesta Andrés | Müller Thomas | Suárez Luis |
| Coach | Somalia | Yusuf A. Nur | Messi Lionel | Xavi | Iniesta Andrés |
| Coach | South Africa | Pitso Mosimane | Messi Lionel | Cristiano Ronaldo | Xavi |
| Coach | Spain | Vicente Del Bosque | Messi Lionel | Cristiano Ronaldo | Müller Thomas |
| Coach | Sri Lanka | Jang Jung | Messi Lionel | Iniesta Andrés | Suárez Luis |
| Coach | St. Lucia | Alain Providence | Messi Lionel | invalid vote | invalid vote |
| Coach | Sudan | Mohamed Abdalla Ahmed | Messi Lionel | Cristiano Ronaldo | Iniesta Andrés |

| **Vote** | **Country** | **Name** | **First (5 points)** | **Second (3 points)** | **Third (1 point)** |
| --- | --- | --- | --- | --- | --- |
| Coach | Swaziland | Caleb Ngwenya | Messi Lionel | Xavi | Rooney Wayne |
| Coach | Sweden | Erik Hamren | Messi Lionel | Xavi | Rooney Wayne |
| Coach | Switzerland | Ottmar Hitzfeld | Messi Lionel | Cristiano Ronaldo | Schweinsteiger Bastian |
| Coach | Tahiti | Eddy Etaeta | Messi Lionel | Cristiano Ronaldo | Rooney Wayne |
| Coach | Tajikistan | Alimjan Rafikov | Messi Lionel | Cristiano Ronaldo | Rooney Wayne |
| Coach | Tanzania | Jan Poulsen | Messi Lionel | Xavi | Cristiano Ronaldo |
| Coach | Thailand | Winfred Schefer | Xavi | Messi Lionel | Iniesta Andrés |
| Coach | Tonga | Chris Williams | Iniesta Andrés | Messi Lionel | Xavi |
| Coach | Trinidad and Tobago | Otto Pfister | Messi Lionel | Cristiano Ronaldo | Eto'o Samuel |
| Coach | Tunisia | Sami Trabelsi | Messi Lionel | Forlán Diego | Casillas Iker |
| Coach | Turkey | Guus Hiddink | Messi Lionel | Sneijder Wesley | Xavi |
| Coach | Turks and Caicos Islands | Gary Brough | Messi Lionel | Cristiano Ronaldo | Xavi |
| Coach | Ukraine | Oleg Blokhin | Messi Lionel | Cristiano Ronaldo | Xavi |
| Coach | Uruguay | Óscar Tabárez | Messi Lionel | Cristiano Ronaldo | Piqué Gerard |
| Coach | US Virgin Islands | Terrence Jones | Messi Lionel | Cristiano Ronaldo | Rooney Wayne |
| Coach | USA | Juergen Klinsmann | Messi Lionel | Cristiano Ronaldo | Forlán Diego |
| Coach | Uzbekistan | Vadim Abramov | Messi Lionel | Cristiano Ronaldo | Agüero Sergio |
| Coach | Vanuatu | Saby Natonga | Messi Lionel | Cristiano Ronaldo | Forlán Diego |
| Coach | Venezuela | Cesar Farias | Forlán Diego | Iniesta Andrés | Suárez Luis |
| Coach | Vietnam | Falko Goetz | Iniesta Andrés | Messi Lionel | Schweinsteiger Bastian |
| Coach | Wales | Gary Speed | Messi Lionel | Xavi | Iniesta Andrés |
| Coach | Yemen | Ameen Al Sunaini | Messi Lionel | Xavi | Cristiano Ronaldo |
| Coach | Zimbabwe | Norman Mapeza | Messi Lionel | Cristiano Ronaldo | Xavi |
| Media | Albania | Besnik Dizdari | Messi Lionel | Cristiano Ronaldo | Forlán Diego |
| Media | Algeria | Yazid Ouahib | Messi Lionel | Xavi | Iniesta Andrés |
| Media | Andorra | Manuel Rodriguez "Tonono" | Messi Lionel | Xavi | Cristiano Ronaldo |
| Media | Angola | Mateus Goncalves | Messi Lionel | Cristiano Ronaldo | Rooney Wayne |
| Media | Argentina | Enrique Sacco | Messi Lionel | Xavi | Cristiano Ronaldo |
| Media | Armenia | Souren Baghdasarian | Messi Lionel | Cristiano Ronaldo | Xavi |
| Media | Australia | Craig Foster | Messi Lionel | Xavi | Cristiano Ronaldo |
| Media | Austria | Walter Kowatsch-Schwarz | Messi Lionel | Cristiano Ronaldo | Xavi |
| Media | Azerbaijan | Rasim Mövsümov | Xavi | Messi Lionel | Cristiano Ronaldo |
| Media | Bahrain | Abdullah Ashoor | Messi Lionel | Cristiano Ronaldo | Rooney Wayne |
| Media | Bangladesh | Raihan Mahamud | Messi Lionel | Cristiano Ronaldo | Forlán Diego |
| Media | Barbados | Ezra Stuart | Messi Lionel | Cristiano Ronaldo | Rooney Wayne |
| Media | Belarus | Sergey Nikolaev | Messi Lionel | Cristiano Ronaldo | Xavi |

| **Vote** | **Country** | **Name** | **First (5 points)** | **Second (3 points)** | **Third (1 point)** |
| --- | --- | --- | --- | --- | --- |
| Media | Belgium | Michel Dubois | Messi Lionel | Iniesta Andrés | Cristiano Ronaldo |
| Media | Belize | Ruben Morales Iglesias | Messi Lionel | Cristiano Ronaldo | Forlán Diego |
| Media | Benin | René Sagbo | Messi Lionel | Cristiano Ronaldo | Xavi |
| Media | Bolivia | Carlos Enrique Rivera | Forlán Diego | Suárez Luis | Messi Lionel |
| Media | Bosnia-Herzegovina | Ahmed Buric | Messi Lionel | Agüero Sergio | Rooney Wayne |
| Media | Botswana | Oaitse Mathala | Messi Lionel | Cristiano Ronaldo | Xavi |
| Media | Brazil | Cleber Machado | Messi Lionel | Cristiano Ronaldo | Neymar |
| Media | Bulgaria | Michel Savov / Roumen Paytashev | Messi Lionel | Cristiano Ronaldo | Suárez Luis |
| Media | Burkina Faso | Victorien Marie Hien | Messi Lionel | Cristiano Ronaldo | Müller Thomas |
| Media | Burundi | Désiré Hatungimana | Messi Lionel | Casillas Iker | Eto'o Samuel |
| Media | Cambodia | Ung Chamroeun | Neymar | Messi Lionel | Fàbregas Cesc |
| Media | Cameroon | Brice Mbeze | Messi Lionel | Rooney Wayne | Suárez Luis |
| Media | Canada | Neil Davidson | Messi Lionel | Villa David | Rooney Wayne |
| Media | Cayman Islands | Ron Shillingford | Messi Lionel | Cristiano Ronaldo | Iniesta Andrés |
| Media | Central African Republic | Albert Stanislas Koumbobacko | Messi Lionel | Cristiano Ronaldo | Eto'o Samuel |
| Media | Chad | Eric Topona | Cristiano Ronaldo | Messi Lionel | Villa David |
| Media | Chile | Danilo Diaz | Messi Lionel | Suárez Luis | Neymar |
| Media | China PR | Luo Ming | Messi Lionel | Xavi | Cristiano Ronaldo |
| Media | Colombia | Paché Andrade | Messi Lionel | Cristiano Ronaldo | Suárez Luis |
| Media | Comoros | Abdou Boina | Messi Lionel | Cristiano Ronaldo | Iniesta Andrés |
| Media | Congo | James Golden Eloue | Messi Lionel | Cristiano Ronaldo | Eto'o Samuel |
| Media | Costa Rica | Rodrigo Antonio Calvo Castro | Messi Lionel | Suárez Luis | Neymar |
| Media | Côte d'Ivoire | Adam Khalil | Messi Lionel | Cristiano Ronaldo | Abidal Éric |
| Media | Croatia | Zdravko Reic | Messi Lionel | Cristiano Ronaldo | Iniesta Andrés |
| Media | Cuba | Miguel Hernandez | Messi Lionel | Cristiano Ronaldo | Forlán Diego |
| Media | Cyprus | Michel Gavrielides | Messi Lionel | Xavi | Forlán Diego |
| Media | Czech Republic | Stanislav Hrabe | Messi Lionel | Cristiano Ronaldo | Xavi |
| Media | Denmark | Niels Jörgen Larsen | Messi Lionel | Cristiano Ronaldo | Iniesta Andrés |
| Media | Djibouti | Ibrahim Mohamed Ibrahime | Benzema Karim | Eto'o Samuel | Messi Lionel |
| Media | Dominican Republic | Jorge Rolando Bauger | Messi Lionel | Neymar | Xavi |
| Media | Ecuador | Fabricio Zavala Garcia | Messi Lionel | Cristiano Ronaldo | Xavi |
| Media | Egypt | Inas Mazhar | Messi Lionel | Cristiano Ronaldo | Neymar |
| Media | El Salvador | Mario Ernesto Posada Flores | Xavi | Messi Lionel | Cristiano Ronaldo |
| Media | England | Henry Winter | Messi Lionel | Cristiano Ronaldo | Xavi |
| Media | Equatorial Guinea | David Monsuy | Forlán Diego | Rooney Wayne | Abidal Éric |
| Media | Ethiopia | Mensur Abdulkeni | Messi Lionel | Xavi | Cristiano Ronaldo |

| **Vote** | **Country** | **Name** | **First (5 points)** | **Second (3 points)** | **Third (1 point)** |
| --- | --- | --- | --- | --- | --- |
| Media | Finland | Matti Einiö | Messi Lionel | Cristiano Ronaldo | Suárez Luis |
| Media | France | Denis Chaumier | Messi Lionel | Xavi | Cristiano Ronaldo |
| Media | FYR Macedonia | Boro Timkovski | Messi Lionel | Cristiano Ronaldo | Xavi |
| Media | Gabon | James Angelo Loundou | Messi Lionel | Cristiano Ronaldo | Rooney Wayne |
| Media | Georgia | Zurab Potskhveria / Vakhtang Bzikadze | Cristiano Ronaldo | Messi Lionel | Casillas Iker |
| Media | Germany | Karl Heinz Wild | Messi Lionel | Xabi Alonso | Özil Mesut |
| Media | Ghana | Michael Oti Adjei | Messi Lionel | Cristiano Ronaldo | Xavi |
| Media | Greece | Manos Staramopoulos | Messi Lionel | Xavi | Forlán Diego |
| Media | Grenada | Michael Bascombe | Villa David | Suárez Luis | Messi Lionel |
| Media | Guatemala | Francisco Aguilar | Messi Lionel | Suárez Luis | Xavi |
| Media | Haiti | Enock Nere | Messi Lionel | Cristiano Ronaldo | Xavi |
| Media | Honduras | Francisco Antonio Rivas Garcia | Xavi | Messi Lionel | Cristiano Ronaldo |
| Media | Hungary | Mathias Imre | Messi Lionel | Xavi | Cristiano Ronaldo |
| Media | Iceland | Vidir Sigurdsson | Messi Lionel | Cristiano Ronaldo | Suárez Luis |
| Media | India | Dhiman Sarkar | Messi Lionel | Cristiano Ronaldo | Suárez Luis |
| Media | Indonesia | Nurdin Saleh | Messi Lionel | Cristiano Ronaldo | Suárez Luis |
| Media | Iran | Siamak Rahmani | Messi Lionel | Rooney Wayne | Cristiano Ronaldo |
| Media | Iraq | Sami Abdul Emam | Messi Lionel | Cristiano Ronaldo | Rooney Wayne |
| Media | Israel | Noah Klieger / Nadav Jacobi | Messi Lionel | Xavi | Cristiano Ronaldo |
| Media | Italy | Paolo Condo | Messi Lionel | Cristiano Ronaldo | Iniesta Andrés |
| Media | Japan | Shuichi Tamura | Messi Lionel | Iniesta Andrés | Cristiano Ronaldo |
| Media | Jordan | Mohamed Kadre Hassan | Messi Lionel | Rooney Wayne | Cristiano Ronaldo |
| Media | Kazakhstan | Geniy Tulegenov | Messi Lionel | Xavi | Cristiano Ronaldo |
| Media | Kenya | Charles Nyende | Messi Lionel | Nani | Suárez Luis |
| Media | Korea DPR | Ri Dong Gyu | Messi Lionel | Cristiano Ronaldo | Iniesta Andrés |
| Media | Korea Republic | Hanseok Kim | Messi Lionel | Cristiano Ronaldo | Iniesta Andrés |
| Media | Kuwait | Ahmed Abdulaziz | Messi Lionel | Cristiano Ronaldo | Xavi |
| Media | Kyrgyzstan | Pavel Louzanov | Cristiano Ronaldo | Messi Lionel | Rooney Wayne |
| Media | Latvia | Valery Karpoushkin | Iniesta Andrés | Cristiano Ronaldo | Forlán Diego |
| Media | Lebanon | Mohamed Fawaz | Messi Lionel | Neymar | Cristiano Ronaldo |
| Media | Lesotho | Thabang Matjama | Messi Lionel | Iniesta Andrés | Sneijder Wesley |
| Media | Liberia | J. Burgess Carter | Messi Lionel | Cristiano Ronaldo | Agüero Sergio |
| Media | Libya | Joseph Cutajar / Mjuftah Beilleid Hussein | Messi Lionel | Iniesta Andrés | Rooney Wayne |
| Media | Liechtenstein | Ernst Hasler | Messi Lionel | Xavi | Özil Mesut |
| Media | Lithuania | Giedrius Janonis | Messi Lionel | Cristiano Ronaldo | Xavi |
| Media | Luxembourg | Didier Hiegel / Christophe Nadin | Messi Lionel | Cristiano Ronaldo | Rooney Wayne |

| **Vote** | **Country** | **Name** | **First (5 points)** | **Second (3 points)** | **Third (1 point)** |
| --- | --- | --- | --- | --- | --- |
| Media | Madagascar | Clément Rabary | Messi Lionel | Rooney Wayne | Benzema Karim |
| Media | Malawi | Peter Kanjere | Messi Lionel | Casillas Iker | Forlán Diego |
| Media | Malaysia | Rizal Hashim | Messi Lionel | Cristiano Ronaldo | Suárez Luis |
| Media | Maldives | Shimaz Ali | Messi Lionel | Forlán Diego | Villa David |
| Media | Mali | Souleymane Bobo Tounkara | Messi Lionel | Cristiano Ronaldo | Iniesta Andrés |
| Media | Malta | Charles Camenzuli | Messi Lionel | Neymar | Villa David |
| Media | Mauritania | Mohamed Ould El Hacen | Messi Lionel | Cristiano Ronaldo | Benzema Karim |
| Media | Mauritius | Azmaal Hydoo | Messi Lionel | Cristiano Ronaldo | Rooney Wayne |
| Media | Mexico | Salvador Aguilera | Messi Lionel | Cristiano Ronaldo | Neymar |
| Media | Moldova | Sergei Donets | Messi Lionel | Cristiano Ronaldo | Rooney Wayne |
| Media | Montenegro | Danilo Mitrovic | Messi Lionel | Cristiano Ronaldo | Iniesta Andrés |
| Media | Morocco | Mostafa Badri | Xavi | Messi Lionel | Cristiano Ronaldo |
| Media | Mozambique | Alexandre Zandamela | Messi Lionel | Cristiano Ronaldo | Villa David |
| Media | Namibia | Sheefeni Nikodemus | Messi Lionel | Neymar | Cristiano Ronaldo |
| Media | Netherlands | Frans Van Den Nieuwenhof | Messi Lionel | Cristiano Ronaldo | Piqué Gerard |
| Media | New Caledonia | Christophe Chohin | Messi Lionel | Dani Alves | Abidal Éric |
| Media | New Zealand | Gordon Glen Watson | Messi Lionel | Cristiano Ronaldo | Iniesta Andrés |
| Media | Niger | Mohamed Silimane Ganoua | Messi Lionel | Cristiano Ronaldo | Iniesta Andrés |
| Media | Nigeria | Samm Audu | Messi Lionel | Cristiano Ronaldo | Forlán Diego |
| Media | Northern Ireland | Jackie Fullerton / Joel Taggart | Messi Lionel | Iniesta Andrés | Abidal Éric |
| Media | Norway | Arild Sandven | Messi Lionel | Iniesta Andrés | Xavi |
| Media | Oman | Saleh Al-Barhi | Messi Lionel | Cristiano Ronaldo | Xavi |
| Media | Palestine | Mohamed Iraqi | Messi Lionel | Iniesta Andrés | Suárez Luis |
| Media | Panama | Campo Elias Estrada | Messi Lionel | Cristiano Ronaldo | Suárez Luis |
| Media | Paraguay | Ruben Dario Da Rosa | Messi Lionel | Cristiano Ronaldo | Neymar |
| Media | Peru | Carlos Salinas | Messi Lionel | Cristiano Ronaldo | Neymar |
| Media | Poland | Maciej Iwanski | Messi Lionel | Cristiano Ronaldo | Suárez Luis |
| Media | Portugal | Joaquim Rita | Messi Lionel | Cristiano Ronaldo | Xavi |
| Media | Puerto Rico | Luis Santiago Arce | Messi Lionel | Cristiano Ronaldo | Iniesta Andrés |
| Media | Qatar | Majed M. Alkhalifi | Messi Lionel | Cristiano Ronaldo | Xavi |
| Media | Republic of Ireland | Paul Kelly / Jimmy Magee | Messi Lionel | Xavi | Cristiano Ronaldo |
| Media | Romania | Emmanuel Rossu | Messi Lionel | Xavi | Nani |
| Media | Russia | Konstantin Kleshchev | Messi Lionel | Cristiano Ronaldo | Xavi |
| Media | Rwanda | Bonnie Mugabe | Messi Lionel | Suárez Luis | Forlán Diego |
| Media | San Marino | Marco Zunino | Messi Lionel | Neymar | Suárez Luis |
| Media | Saudi Arabia | Rajallah Alsolami | Messi Lionel | Cristiano Ronaldo | Neymar |

| **Vote** | **Country** | **Name** | **First (5 points)** | **Second (3 points)** | **Third (1 point)** |
| --- | --- | --- | --- | --- | --- |
| Media | Scotland | John Greechan | Messi Lionel | Xavi | Abidal Éric |
| Media | Senegal | Aliou Goloko | Messi Lionel | Forlán Diego | Rooney Wayne |
| Media | Serbia | Vladimir Novak | Messi Lionel | Cristiano Ronaldo | Xavi |
| Media | Seychelles | Gérard Govinden | Messi Lionel | Cristiano Ronaldo | Xavi |
| Media | Sierra Leone | Mohamed Fajah Barrie | Messi Lionel | Cristiano Ronaldo | Xavi |
| Media | Slovakia | Peter Surin | Messi Lionel | Cristiano Ronaldo | Suárez Luis |
| Media | Slovenia | Andrej Stare | Messi Lionel | Cristiano Ronaldo | Xavi |
| Media | South Africa | Mark Gleeson | Messi Lionel | Cristiano Ronaldo | Iniesta Andrés |
| Media | Spain | Paco Aguilar | Messi Lionel | Xavi | Cristiano Ronaldo |
| Media | Sudan | Muzamil Abu Elgassim | Messi Lionel | Iniesta Andrés | Cristiano Ronaldo |
| Media | Suriname | Desney Romeo | Messi Lionel | Cristiano Ronaldo | Suárez Luis |
| Media | Swaziland | Kenneth Dlamini | Messi Lionel | Suárez Luis | Neymar |
| Media | Sweden | Henrik Ysten | Messi Lionel | Cristiano Ronaldo | Iniesta Andrés |
| Media | Switzerland | Pierre-Alain Dupuis | Messi Lionel | Xavi | Cristiano Ronaldo |
| Media | Tahiti | Olivier Huc | Messi Lionel | Iniesta Andrés | Cristiano Ronaldo |
| Media | Tajikistan | Alaveddine Bouriev | Cristiano Ronaldo | Messi Lionel | Rooney Wayne |
| Media | Tanzania | Boniface Wambura | Messi Lionel | Agüero Sergio | Villa David |
| Media | Thailand | Urai Patoommawatana | Messi Lionel | Cristiano Ronaldo | Xavi |
| Media | Togo | Mathias Ayena | Messi Lionel | Cristiano Ronaldo | Eto'o Samuel |
| Media | Trinidad and Tobago | Lasana Liburd | Messi Lionel | Cristiano Ronaldo | Suárez Luis |
| Media | Tunisia | Abdesslam Dhaifallah | Messi Lionel | Cristiano Ronaldo | Abidal Éric |
| Media | Turkey | Selçuk Manav | Messi Lionel | Cristiano Ronaldo | Özil Mesut |
| Media | Turkmenistan | Alexander Vershinin | Messi Lionel | Xavi | Iniesta Andrés |
| Media | Uganda | Fredrick Musisi Kiyingi | Messi Lionel | Cristiano Ronaldo | Suárez Luis |
| Media | Ukraine | Igor Linnyk | Messi Lionel | Xavi | Rooney Wayne |
| Media | United Arab Emirates | Dafrallah Mouadhen | Messi Lionel | Xavi | Rooney Wayne |
| Media | Uruguay | Ricardo Pineyrua | Messi Lionel | Iniesta Andrés | Suárez Luis |
| Media | USA | Paul Kennedy | Messi Lionel | Cristiano Ronaldo | Xavi |
| Media | Uzbekistan | Grigoriy Rtveladze | Cristiano Ronaldo | Rooney Wayne | Suárez Luis |
| Media | Venezuela | Francisco Blavia | Xavi | Messi Lionel | Cristiano Ronaldo |
| Media | Vietnam | Truong Anh Ngoc | Messi Lionel | Cristiano Ronaldo | Xavi |
| Media | Wales | Paul Abbandonato | Messi Lionel | Cristiano Ronaldo | Piqué Gerard |
| Media | Yemen | Abdel Al Hababi | Messi Lionel | Cristiano Ronaldo | Xavi |
| Media | Zambia | Chapadongo Lungu | Messi Lionel | Cristiano Ronaldo | Rooney Wayne |
| Media | Zimbabwe | Charles Mabika | Messi Lionel | Cristiano Ronaldo | Casillas Iker |

**FIFA** **BALLON** **D'OR** **2012**

| **Vote** | **Country** | **Name** | **First** **(5** **points)** | **Second** **(3** **points)** | **Third** **(1** **point)** |
| --- | --- | --- | --- | --- | --- |
| Captain | Albania | Cana Lorik | Cristiano Ronaldo | Messi Lionel | Iniesta Andrés |
| Captain | Algeria | Bougherra Madjid | Benzema Karim | Messi Lionel | Cristiano Ronaldo |
| Captain | American Samoa | Amisone Liatama | Cristiano Ronaldo | Messi Lionel | Agüero Sergio |
| Captain | Andorra | Sonejee Masand Oscar | Iniesta Andrés | Messi Lionel | Cristiano Ronaldo |
| Captain | Anguilla | Girdon Connor | Messi Lionel | Cristiano Ronaldo | van Persie Robin |
| Captain | Antigua and Barbuda | Dublin George | Messi Lionel | Cristiano Ronaldo | Iniesta Andrés |
| Captain | Argentina | Messi Lionel | Iniesta Andrés | Xavi | Agüero Sergio |
| Captain | Armenia | Berezovski Roman | Messi Lionel | Cristiano Ronaldo | Casillas Iker |
| Captain | Aruba | Baten Raymond | Iniesta Andrés | Özil Mesut | Busquets Sergio |
| Captain | Australia | Neill Lucas | Cristiano Ronaldo | van Persie Robin | Messi Lionel |
| Captain | Austria | Fuchs Christian | Xavi | Iniesta Andrés | Balotelli Mario |
| Captain | Azerbaijan | Sadikhov Rashad | Messi Lionel | Cristiano Ronaldo | Iniesta Andrés |
| Captain | Bahamas | Leslie St. Fleur | Messi Lionel | Rooney Wayne | Touré Yaya |
| Captain | Bangladesh | Sujon Md. | Messi Lionel | Iniesta Andrés | Cristiano Ronaldo |
| Captain | Barbados | Williams Rashida | Cristiano Ronaldo | Iniesta Andrés | Messi Lionel |
| Captain | Belarus | Veremko Siarhei | Messi Lionel | Cristiano Ronaldo | Xavi |
| Captain | Belgium | Kompany Vincent | Messi Lionel | Xavi | Falcao Radamel |
| Captain | Belize | Gaynair Ian | Cristiano Ronaldo | Messi Lionel | Casillas Iker |
| Captain | Bermuda | Nusum John Barry | Iniesta Andrés | Messi Lionel | Cristiano Ronaldo |
| Captain | Bhutan | Tshering Pasang | Messi Lionel | Cristiano Ronaldo | Iniesta Andrés |
| Captain | Bolivia | Raldes Ronald | Messi Lionel | Cristiano Ronaldo | Casillas Iker |
| Captain | Bosnia-Herzegovina | Emir Spahic | Messi Lionel | Falcao Radamel | Xavi |
| Captain | Botswana | Thuma Mompati | Messi Lionel | Cristiano Ronaldo | Xavi |
| Captain | Brazil | Da Silva Thiago Emiliano | Messi Lionel | Cristiano Ronaldo | Ibrahimovic Zlatan |
| Captain | Brunei Darussalam | Haji Kamis Rosmin | Messi Lionel | Iniesta Andrés | Casillas Iker |
| Captain | Bulgaria | Popov Ivelin | Messi Lionel | Cristiano Ronaldo | Iniesta Andrés |
| Captain | Burkina Faso | Dagano Moumouni | Messi Lionel | Iniesta Andrés | Cristiano Ronaldo |
| Captain | Burundi | Nahayo Valery | Messi Lionel | Iniesta Andrés | Cristiano Ronaldo |
| Captain | Cambodia | Sok Ngon Keo | Iniesta Andrés | Pirlo Andrea | Xavi |
| Captain | Cameroon | Eto'o Fils Samuel | Messi Lionel | Touré Yaya | Xavi |
| Captain | Canada | Mckenna Kevin | Messi Lionel | Cristiano Ronaldo | Xavi |
| Captain | Cape Verde Islands | Neves Fernando | Cristiano Ronaldo | Drogba Didier | Messi Lionel |
| Captain | Cayman Islands | Lindo Ian | Messi Lionel | Cristiano Ronaldo | Iniesta Andrés |
| Captain | Chad | Koulara Armel | Messi Lionel | Cristiano Ronaldo | Iniesta Andrés |

| **Vote** | **Country** | **Name** | **First** **(5** **points)** | **Second** **(3** **points)** | **Third** **(1** **point)** |
| --- | --- | --- | --- | --- | --- |
| Captain | China PR | Zheng Zhi | Xavi | Messi Lionel | Alonso Xabi |
| Captain | Colombia | Yepes Mario Alberto | Falcao Radamel | Cristiano Ronaldo | Messi Lionel |
| Captain | Comoros | Mroivili Mahamoud | Iniesta Andrés | Ramos Sergio | van Persie Robin |
| Captain | Congo | Andzouana Kevin | Cristiano Ronaldo | Iniesta Andrés | Messi Lionel |
| Captain | Congo DR | Mputu Mabi Trésor | Falcao Radamel | Messi Lionel | Cristiano Ronaldo |
| Captain | Costa Rica | Ruiz Bryan | Messi Lionel | Iniesta Andrés | Cristiano Ronaldo |
| Captain | Croatia | Srna Darijo | Messi Lionel | Casillas Iker | Ramos Sergio |
| Captain | Cuba | Molina Odelin | Alonso Xabi | Messi Lionel | Cristiano Ronaldo |
| Captain | Curaçao | Bernardus Ashar | Messi Lionel | Drogba Didier | Iniesta Andrés |
| Captain | Cyprus | Constantinou Michael | Iniesta Andrés | Drogba Didier | Xavi |
| Captain | Czech Republic | Rosický Tomáš | Messi Lionel | Cristiano Ronaldo | van Persie Robin |
| Captain | Denmark | Agger Daniel | Pirlo Andrea | Iniesta Andrés | Messi Lionel |
| Captain | Djibouti | Mohamed Kader Ahmed | Ibrahimovic Zlatan | Messi Lionel | Touré Yaya |
| Captain | Dominican Republic | Barmettler Heinz | Cristiano Ronaldo | Messi Lionel | Neymar |
| Captain | Ecuador | Ayovi Walter | Xavi | Messi Lionel | Neymar |
| Captain | Egypt | Elhadary Essam | Messi Lionel | Cristiano Ronaldo | Pirlo Andrea |
| Captain | El Salvador | Portillo Dagoberto | Messi Lionel | Alonso Xabi | Iniesta Andrés |
| Captain | England | Gerrard Steven | Messi Lionel | Cristiano Ronaldo | Alonso Xabi |
| Captain | Eritrea | Goitom Daniel | Cristiano Ronaldo | Iniesta Andrés | Falcao Radamel |
| Captain | Estonia | Klavan Ragnar | van Persie Robin | Messi Lionel | Pirlo Andrea |
| Captain | Ethiopia | Debebe Degu | Cristiano Ronaldo | Messi Lionel | Xavi |
| Captain | Faroe Islands | Benjaminsen Fróði | Messi Lionel | Cristiano Ronaldo | Iniesta Andrés |
| Captain | Fiji | Waqa Taniela | Messi Lionel | Cristiano Ronaldo | Falcao Radamel |
| Captain | Finland | Moisander Niklas | Messi Lionel | Cristiano Ronaldo | Ibrahimovic Zlatan |
| Captain | France | Lloris Hugo | Casillas Iker | Falcao Radamel | Drogba Didier |
| Captain | FYR Macedonia | Pandev Goran | Messi Lionel | van Persie Robin | Xavi |
| Captain | Georgia | Kankava Jaba | Rooney Wayne | Messi Lionel | Xavi |
| Captain | Germany | Lahm Philipp | Iniesta Andrés | Messi Lionel | Cristiano Ronaldo |
| Captain | Ghana | Gyan Asamoah | Messi Lionel | Touré Yaya | Rooney Wayne |
| Captain | Greece | Salpingidis Dimitrios | Messi Lionel | Cristiano Ronaldo | Falcao Radamel |
| Captain | Grenada | Marshall Marc | Messi Lionel | Falcao Radamel | Neymar |
| Captain | Guam | Cunliffe Jason | Iniesta Andrés | Messi Lionel | Alonso Xabi |
| Captain | Guatemala | Ruiz Gutierrez Carlos Humberto | Cristiano Ronaldo | Casillas Iker | Messi Lionel |
| Captain | Guinea | Zayatte Kamil | Cristiano Ronaldo | Messi Lionel | Iniesta Andrés |
| Captain | Guyana | Nurse Chris | Drogba Didier | Messi Lionel | Cristiano Ronaldo |
| Captain | Honduras | Valladares Noel | Messi Lionel | Cristiano Ronaldo | Falcao Radamel |

| **Vote** | **Country** | **Name** | **First** **(5** **points)** | **Second** **(3** **points)** | **Third** **(1** **point)** |
| --- | --- | --- | --- | --- | --- |
| Captain | Hong Kong | Chan Wai Ho | Messi Lionel | Cristiano Ronaldo | Drogba Didier |
| Captain | Hungary | Gera Zoltán | Cristiano Ronaldo | Messi Lionel | van Persie Robin |
| Captain | Iceland | Gunnarsson Aron Einar | Cristiano Ronaldo | Messi Lionel | Ibrahimovic Zlatan |
| Captain | India | Chhetri Sunil | Iniesta Andrés | Messi Lionel | Cristiano Ronaldo |
| Captain | Italy | Buffon Gianluigi | Pirlo Andrea | Messi Lionel | Cristiano Ronaldo |
| Captain | Jamaica | Thomas Shavar | Alonso Xabi | Messi Lionel | Neymar |
| Captain | Japan | Hasebe Makoto | Messi Lionel | Cristiano Ronaldo | Pirlo Andrea |
| Captain | Jordan | Deeb Amer | Messi Lionel | Iniesta Andrés | Cristiano Ronaldo |
| Captain | Kazakhstan | Nurdauletov Kairat | Xavi | Messi Lionel | Cristiano Ronaldo |
| Captain | Korea DPR | Ri Myong Guk | Messi Lionel | Drogba Didier | Özil Mesut |
| Captain | Korea Republic | Ha Daesung | Xavi | Busquets Sergio | Özil Mesut |
| Captain | Kuwait | Al-khaldi Nawaf | Messi Lionel | Cristiano Ronaldo | Falcao Radamel |
| Captain | Kyrgyzstan | Baimatov Azamat | Cristiano Ronaldo | Messi Lionel | Casillas Iker |
| Captain | Laos | Phaphouvaninh Vixay | Messi Lionel | Cristiano Ronaldo | Balotelli Mario |
| Captain | Latvia | Gorkšs Kaspars | Messi Lionel | van Persie Robin | Iniesta Andrés |
| Captain | Lebanon | Antar Roda | Messi Lionel | Cristiano Ronaldo | Falcao Radamel |
| Captain | Liberia | Gebro George Duncan | Messi Lionel | Cristiano Ronaldo | Drogba Didier |
| Captain | Liechtenstein | Stocklasa Martin | Messi Lionel | Falcao Radamel | Alonso Xabi |
| Captain | Lithuania | Danilevicius Tomas | Ibrahimovic Zlatan | Rooney Wayne | Piqué Gerard |
| Captain | Luxembourg | Peters René | Messi Lionel | Xavi | Iniesta Andrés |
| Captain | Macau | Cheng Ieong Paulo Cheang | Cristiano Ronaldo | Messi Lionel | Pirlo Andrea |
| Captain | Madagascar | Rajoarimanana Yvan | Xavi | Messi Lionel | Iniesta Andrés |
| Captain | Malawi | Chavula Moses | Messi Lionel | Cristiano Ronaldo | Neymar |
| Captain | Maldives | Ashfaq Ali | Messi Lionel | Iniesta Andrés | Cristiano Ronaldo |
| Captain | Mali | Coulibaly Adama | Drogba Didier | Ibrahimovic Zlatan | Messi Lionel |
| Captain | Malta | Mifsud Michael | Messi Lionel | Iniesta Andrés | Buffon Gianluigi |
| Captain | Mauritania | Baghayoko Moussa | Messi Lionel | Iniesta Andrés | Cristiano Ronaldo |
| Captain | Mauritius | Bell Colin | Messi Lionel | Cristiano Ronaldo | Rooney Wayne |
| Captain | Mexico | Rodriguez Pinedo Francisco Javier | Messi Lionel | Falcao Radamel | Cristiano Ronaldo |
| Captain | Moldova | Epureanu Alexandru | Messi Lionel | Cristiano Ronaldo | Iniesta Andrés |
| Captain | Mongolia | Donorov Lumbengarav | Messi Lionel | Cristiano Ronaldo | Iniesta Andrés |
| Captain | Montenegro | Vucinic Mirko | Buffon Gianluigi | Pirlo Andrea | Ibrahimovic Zlatan |
| Captain | Montserrat | Mendes Junior | Messi Lionel | Iniesta Andrés | Cristiano Ronaldo |
| Captain | Morocco | Lamyaghri Nadir | Messi Lionel | Casillas Iker | Cristiano Ronaldo |
| Captain | Mozambique | Rafael Joao | Cristiano Ronaldo | Drogba Didier | Neymar |
| Captain | Myanmar | Khin Maung Lwin | Casillas Iker | Messi Lionel | Falcao Radamel |

| **Vote** | **Country** | **Name** | **First** **(5** **points)** | **Second** **(3** **points)** | **Third** **(1** **point)** |
| --- | --- | --- | --- | --- | --- |
| Captain | Namibia | Ketjijere Ronald | Drogba Didier | Cristiano Ronaldo | Alonso Xabi |
| Captain | Netherlands | Sneijder Wesley | van Persie Robin | Messi Lionel | Cristiano Ronaldo |
| Captain | New Caledonia | Dokunengo Olivier | Cristiano Ronaldo | Messi Lionel | Falcao Radamel |
| Captain | New Zealand | Nelsen Ryan | Messi Lionel | Cristiano Ronaldo | Xavi |
| Captain | Nicaragua | Solorzano David | Messi Lionel | Xavi | Agüero Sergio |
| Captain | Niger | Ouwo Moussa Maazou | Messi Lionel | Cristiano Ronaldo | Touré Yaya |
| Captain | Nigeria | Yobo Joseph | Messi Lionel | Cristiano Ronaldo | Neymar |
| Captain | Northern Ireland | Davis Steve | Messi Lionel | Cristiano Ronaldo | Iniesta Andrés |
| Captain | Norway | Hangeland Brede | Messi Lionel | Pirlo Andrea | Touré Yaya |
| Captain | Pakistan | Khan Jaffar | Casillas Iker | Iniesta Andrés | Xavi |
| Captain | Palestine | Fahed Attal | Messi Lionel | Casillas Iker | Balotelli Mario |
| Captain | Panama | Felipe Baloy | Messi Lionel | Cristiano Ronaldo | Neymar |
| Captain | Papua New Guinea | Muta David | Messi Lionel | Cristiano Ronaldo | van Persie Robin |
| Captain | Philippines | Caligdong Emelio | Messi Lionel | Xavi | Iniesta Andrés |
| Captain | Poland | Blaszczykowski Jakub | Messi Lionel | Cristiano Ronaldo | Iniesta Andrés |
| Captain | Portugal | Alves Bruno | Cristiano Ronaldo | Falcao Radamel | van Persie Robin |
| Captain | Puerto Rico | Velez Marco | Messi Lionel | Iniesta Andrés | Falcao Radamel |
| Captain | Republic of Ireland | Keane Robbie | Messi Lionel | Cristiano Ronaldo | Iniesta Andrés |
| Captain | Romania | Rat Razvan | Messi Lionel | Ibrahimovic Zlatan | Cristiano Ronaldo |
| Captain | Russia | Denisov Igor | Messi Lionel | Cristiano Ronaldo | Ibrahimovic Zlatan |
| Captain | Rwanda | Ndoli Jean Claude | Messi Lionel | Cristiano Ronaldo | Falcao Radamel |
| Captain | Samoa | Setefano Andrew | Alonso Xabi | Pirlo Andrea | Neuer Manuel |
| Captain | San Marino | Andy Selva | Pirlo Andrea | Messi Lionel | Cristiano Ronaldo |
| Captain | São Tomé e Príncipe | Neves Derilson | Messi Lionel | Cristiano Ronaldo | Iniesta Andrés |
| Captain | Saudi Arabia | Osama Hawsawi | Messi Lionel | Xavi | Iniesta Andrés |
| Captain | Scotland | Fletcher Darren | Cristiano Ronaldo | Messi Lionel | van Persie Robin |
| Captain | Senegal | Mohamed Diame | Messi Lionel | Cristiano Ronaldo | Touré Yaya |
| Captain | Serbia | Ivanovic Branislav | Cristiano Ronaldo | Drogba Didier | Messi Lionel |
| Captain | Seychelles | Barbe Denis | Messi Lionel | Cristiano Ronaldo | Falcao Radamel |
| Captain | Singapore | Ishak Shahril | Cristiano Ronaldo | Messi Lionel | Pirlo Andrea |
| Captain | Slovakia | Skrtel Martin | Cristiano Ronaldo | Falcao Radamel | Messi Lionel |
| Captain | Slovenia | Handanovic Samir | Messi Lionel | Cristiano Ronaldo | Xavi |
| Captain | Somalia | Abdikarim Nur | Messi Lionel | Cristiano Ronaldo | Casillas Iker |
| Captain | South Africa | Khumalo Bongani | Touré Yaya | Xavi | Iniesta Andrés |
| Captain | South Sudan | Ritchard Jastin | Drogba Didier | Iniesta Andrés | Agüero Sergio |
| Captain | Spain | Casillas Iker | Ramos Sergio | Cristiano Ronaldo | Xavi |

| **Vote** | **Country** | **Name** | **First** **(5** **points)** | **Second** **(3** **points)** | **Third** **(1** **point)** |
| --- | --- | --- | --- | --- | --- |
| Captain | Sri Lanka | Bandara Nipuna | Messi Lionel | Neymar | van Persie Robin |
| Captain | Suriname | Aloema Ronny | Messi Lionel | Cristiano Ronaldo | Falcao Radamel |
| Captain | Swaziland | Jerrone Ntshalintshali | Messi Lionel | Cristiano Ronaldo | van Persie Robin |
| Captain | Sweden | Ibrahimovic´ Zlatan | Xavi | Pirlo Andrea | Messi Lionel |
| Captain | Switzerland | Inler Gökhan | Messi Lionel | Iniesta Andrés | Cristiano Ronaldo |
| Captain | Syria | Mosab Balhous | Messi Lionel | Xavi | Iniesta Andrés |
| Captain | Tahiti | Vallar Nicolas | Xavi | Pirlo Andrea | Messi Lionel |
| Captain | Tajikistan | Makhmudov Khurshed | Messi Lionel | Cristiano Ronaldo | Xavi |
| Captain | Thailand | Panupong Wongsa | Cristiano Ronaldo | Iniesta Andrés | van Persie Robin |
| Captain | Trinidad and Tobago | Williams Jan Michael | Messi Lionel | Cristiano Ronaldo | Iniesta Andrés |
| Captain | Tunisia | Mathlouthi Aymen | Messi Lionel | van Persie Robin | Cristiano Ronaldo |
| Captain | Turkey | Belozoglu Emre | Falcao Radamel | Özil Mesut | Cristiano Ronaldo |
| Captain | Turkmenistan | Bayramov Vladimir | Cristiano Ronaldo | Ibrahimovic Zlatan | Messi Lionel |
| Captain | Turks and Caicos Islands | Davilmar Rodney | Messi Lionel | Cristiano Ronaldo | Falcao Radamel |
| Captain | Uganda | Mwesigwa Andrew | Messi Lionel | Pirlo Andrea | Cristiano Ronaldo |
| Captain | Ukraine | Tymoschuk Anatolii | Messi Lionel | Iniesta Andrés | Falcao Radamel |
| Captain | United Arab Emirates | Aljneibi Ismaeil Matar | Iniesta Andrés | Xavi | Pirlo Andrea |
| Captain | Uruguay | Lugano Diego | Messi Lionel | Ibrahimovic Zlatan | Falcao Radamel |
| Captain | US Virgin Islands | Klopp Reid | Messi Lionel | Cristiano Ronaldo | Alonso Xabi |
| Captain | USA | Bocanegra Carlos | Messi Lionel | Cristiano Ronaldo | Falcao Radamel |
| Captain | Uzbekistan | Djeparov Server | Messi Lionel | Iniesta Andrés | Cristiano Ronaldo |
| Captain | Vanuatu | Jean Robert Yelou | Messi Lionel | Cristiano Ronaldo | Drogba Didier |
| Captain | Venezuela | Arango Juan | Iniesta Andrés | Xavi | Messi Lionel |
| Captain | Vietnam | Nguyen Minh Duc | Messi Lionel | Cristiano Ronaldo | Iniesta Andrés |
| Captain | Wales | Ashley Williams | Messi Lionel | Xavi | van Persie Robin |
| Captain | Yemen | Awad Salim | Pirlo Andrea | Agüero Sergio | Messi Lionel |
| Captain | Zambia | Christopher Katongo | Messi Lionel | Alonso Xabi | Iniesta Andrés |
| Captain | Zimbabwe | Matongorere Nelson | Cristiano Ronaldo | Piqué Gerard | Messi Lionel |
| Coach | Albania | De Biasi Giovanni | Messi Lionel | Cristiano Ronaldo | Alonso Xabi |
| Coach | Algeria | Halilhodzic Vahid | Messi Lionel | Cristiano Ronaldo | Drogba Didier |
| Coach | American Samoa | Lalogafuafua Iofi | Messi Lionel | Cristiano Ronaldo | Agüero Sergio |
| Coach | Andorra | Alvarez De Eulate Jesus Luis | Messi Lionel | Xavi | Iniesta Andrés |
| Coach | Anguilla | Colin Girdon | Messi Lionel | Cristiano Ronaldo | van Persie Robin |
| Coach | Antigua and Barbuda | Curtis Thomas | Cristiano Ronaldo | Messi Lionel | Iniesta Andrés |
| Coach | Argentina | Sabella Alejandro | Messi Lionel | Agüero Sergio | Falcao Radamel |
| Coach | Armenia | Minasyan Vardan | Messi Lionel | Pirlo Andrea | Cristiano Ronaldo |

| **Vote** | **Country** | **Name** | **First** **(5** **points)** | **Second** **(3** **points)** | **Third** **(1** **point)** |
| --- | --- | --- | --- | --- | --- |
| Coach | Aruba | Beeldsnijder Herbert | Messi Lionel | Cristiano Ronaldo | Falcao Radamel |
| Coach | Australia | Osieck Holger | Messi Lionel | Iniesta Andrés | Cristiano Ronaldo |
| Coach | Austria | Koller Marcel | Messi Lionel | Iniesta Andrés | Cristiano Ronaldo |
| Coach | Azerbaijan | Vogts Hans Hubert | Iniesta Andrés | Cristiano Ronaldo | Xavi |
| Coach | Bahamas | Kevin Davies | Messi Lionel | Casillas Iker | Cristiano Ronaldo |
| Coach | Bangladesh | Bari A. K. M. Saiful | Messi Lionel | Cristiano Ronaldo | Iniesta Andrés |
| Coach | Barbados | Forde Colin | Messi Lionel | Cristiano Ronaldo | Iniesta Andrés |
| Coach | Belarus | Kandratsyeu Heorhi | Messi Lionel | Cristiano Ronaldo | Xavi |
| Coach | Belgium | Wilmots Marc | Messi Lionel | Falcao Radamel | Ibrahimovic Zlatan |
| Coach | Belize | Sherrier Le Roy | Messi Lionel | Cristiano Ronaldo | Agüero Sergio |
| Coach | Bermuda | Bascome Andrew | Alonso Xabi | Cristiano Ronaldo | Messi Lionel |
| Coach | Bhutan | Kazunori Ohara | Messi Lionel | Casillas Iker | Cristiano Ronaldo |
| Coach | Bolivia | Azkargorta Xabier | Messi Lionel | Cristiano Ronaldo | Alonso Xabi |
| Coach | Bosnia-Herzegovina | Safet Sušic | Cristiano Ronaldo | Messi Lionel | Ramos Sergio |
| Coach | Botswana | Tshosane Stanley | Messi Lionel | Cristiano Ronaldo | van Persie Robin |
| Coach | Brazil | Venker Menezes Luiz Antonio | Cristiano Ronaldo | Messi Lionel | Falcao Radamel |
| Coach | Brunei Darussalam | Oh Son Kwon | Messi Lionel | Cristiano Ronaldo | Alonso Xabi |
| Coach | Bulgaria | Penev Lyuboslav | Iniesta Andrés | Messi Lionel | Falcao Radamel |
| Coach | Burkina Faso | Put Paul | Messi Lionel | Cristiano Ronaldo | Iniesta Andrés |
| Coach | Burundi | Lotfy Mohammed Nasseem | Messi Lionel | Cristiano Ronaldo | Iniesta Andrés |
| Coach | Cambodia | Sochetra Hok | Messi Lionel | Neymar | Rooney Wayne |
| Coach | Cameroon | Akono Jean Paul | Messi Lionel | Cristiano Ronaldo | Iniesta Andrés |
| Coach | Canada | Hart Stephen | Messi Lionel | Cristiano Ronaldo | Falcao Radamel |
| Coach | Cape Verde Islands | Antunes Ulisses | Cristiano Ronaldo | Messi Lionel | Drogba Didier |
| Coach | Cayman Islands | Tinoco Marcos | Messi Lionel | Cristiano Ronaldo | Neymar |
| Coach | Chad | Mahamat Oumar Yaya | Messi Lionel | Cristiano Ronaldo | Iniesta Andrés |
| Coach | China PR | Camacho Jose | Iniesta Andrés | Xavi | Casillas Iker |
| Coach | Colombia | Pekerman José | Messi Lionel | Falcao Radamel | Cristiano Ronaldo |
| Coach | Comoros | Ali Mbae | Xavi | Iniesta Andrés | Neymar |
| Coach | Congo | Ngatsono Barthelemy | Messi Lionel | Cristiano Ronaldo | Casillas Iker |
| Coach | Congo DR | Le Roy Claude | Falcao Radamel | Messi Lionel | Cristiano Ronaldo |
| Coach | Costa Rica | Pinto Jorge Luis | Falcao Radamel | Alonso Xabi | Iniesta Andrés |
| Coach | Croatia | Štimac Igor | Cristiano Ronaldo | Messi Lionel | Casillas Iker |
| Coach | Cuba | Benites Walter | Messi Lionel | Cristiano Ronaldo | Alonso Xabi |
| Coach | Curaçao | Siliee Etienne | Messi Lionel | Drogba Didier | Ibrahimovic Zlatan |
| Coach | Cyprus | Nioplias Nikolaos | Cristiano Ronaldo | Messi Lionel | Falcao Radamel |

| **Vote** | **Country** | **Name** | **First** **(5** **points)** | **Second** **(3** **points)** | **Third** **(1** **point)** |
| --- | --- | --- | --- | --- | --- |
| Coach | Czech Republic | Bílek Michal | Messi Lionel | Cristiano Ronaldo | Iniesta Andrés |
| Coach | Denmark | Olsen Morten | Iniesta Andrés | Messi Lionel | Xavi |
| Coach | Djibouti | Ahmed Arid | Messi Lionel | Cristiano Ronaldo | Falcao Radamel |
| Coach | Dominican Republic | Hernadez Clemente Domingo | Cristiano Ronaldo | Messi Lionel | Neymar |
| Coach | Ecuador | Rueda Rivera Reinaldo | Falcao Radamel | Messi Lionel | Neuer Manuel |
| Coach | Egypt | Bradley Bob | Cristiano Ronaldo | Messi Lionel | Pirlo Andrea |
| Coach | El Salvador | Castillo Juan De Dios | Messi Lionel | Cristiano Ronaldo | Falcao Radamel |
| Coach | England | Hodgson Roy | Messi Lionel | Cristiano Ronaldo | Falcao Radamel |
| Coach | Eritrea | Teklit Negash | Messi Lionel | Cristiano Ronaldo | Iniesta Andrés |
| Coach | Estonia | Rüütli Tarmo | Messi Lionel | Cristiano Ronaldo | Pirlo Andrea |
| Coach | Ethiopia | Bishaw Sewnet | Cristiano Ronaldo | Messi Lionel | Xavi |
| Coach | Faroe Islands | Olsen Lars | Messi Lionel | Cristiano Ronaldo | Ibrahimovic Zlatan |
| Coach | Fiji | Buzzetti Juan Carlos | Messi Lionel | Cristiano Ronaldo | van Persie Robin |
| Coach | Finland | Paatelainen Mixu | Messi Lionel | Xavi | Cristiano Ronaldo |
| Coach | France | Deschamps Didier | Messi Lionel | Cristiano Ronaldo | Xavi |
| Coach | FYR Macedonia | Janevski Cedomir | Messi Lionel | Casillas Iker | Pirlo Andrea |
| Coach | Georgia | Ketsbaia Temur | Cristiano Ronaldo | Messi Lionel | Iniesta Andrés |
| Coach | Germany | Löw Joachim | Özil Mesut | Neuer Manuel | Xavi |
| Coach | Ghana | Appiah James Akwesi | Messi Lionel | van Persie Robin | Cristiano Ronaldo |
| Coach | Greece | Santos Fernando | Cristiano Ronaldo | Messi Lionel | Falcao Radamel |
| Coach | Grenada | De Bellotte Alister | Messi Lionel | Neymar | Falcao Radamel |
| Coach | Guam | White Gary | Messi Lionel | Alonso Xabi | Cristiano Ronaldo |
| Coach | Guatemala | Almeida Almada Ever Hugo | Cristiano Ronaldo | Casillas Iker | Messi Lionel |
| Coach | Guinea | Dussuyer Michel | Messi Lionel | Cristiano Ronaldo | Iniesta Andrés |
| Coach | Guyana | Shabazz Jamaal | Drogba Didier | Messi Lionel | Cristiano Ronaldo |
| Coach | Honduras | Suarez Luis | Messi Lionel | Pirlo Andrea | Iniesta Andrés |
| Coach | Hong Kong | Kim Pan Gon | Messi Lionel | Cristiano Ronaldo | Alonso Xabi |
| Coach | Hungary | Egervári Sándor | Messi Lionel | Iniesta Andrés | Falcao Radamel |
| Coach | Iceland | Lagerback Lars | Messi Lionel | Alonso Xabi | Ibrahimovic Zlatan |
| Coach | India | Koevermans Wim | Iniesta Andrés | Messi Lionel | Cristiano Ronaldo |
| Coach | Italy | Prandelli Cesare | Pirlo Andrea | Buffon Gianluigi | Messi Lionel |
| Coach | Jamaica | Whitmore Theodore | Messi Lionel | Touré Yaya | Cristiano Ronaldo |
| Coach | Japan | Zaccheroni Alberto | Cristiano Ronaldo | Messi Lionel | Pirlo Andrea |
| Coach | Jordan | Hamad Adnan | Messi Lionel | Cristiano Ronaldo | Iniesta Andrés |
| Coach | Kazakhstan | Beranek Miroslav | Messi Lionel | Cristiano Ronaldo | Pirlo Andrea |
| Coach | Korea DPR | Yun Jong Su | Messi Lionel | Rooney Wayne | Özil Mesut |

| **Vote** | **Country** | **Name** | **First** **(5** **points)** | **Second** **(3** **points)** | **Third** **(1** **point)** |
| --- | --- | --- | --- | --- | --- |
| Coach | Korea Republic | Choi Kanghee | Messi Lionel | Cristiano Ronaldo | Iniesta Andrés |
| Coach | Kuwait | Tufegdžic Goran | Messi Lionel | Xavi | Falcao Radamel |
| Coach | Kyrgyzstan | Dvoryankov Sergey | Cristiano Ronaldo | Messi Lionel | Iniesta Andrés |
| Coach | Laos | Kimura Kokichi | Messi Lionel | Cristiano Ronaldo | van Persie Robin |
| Coach | Latvia | Starkovs Aleksandrs | Messi Lionel | Cristiano Ronaldo | Xavi |
| Coach | Lebanon | Bucker Johannes Theodor | Messi Lionel | Neuer Manuel | Özil Mesut |
| Coach | Liberia | Smith J. Kaetu | Messi Lionel | Cristiano Ronaldo | Drogba Didier |
| Coach | Liechtenstein | Pauritsch Rene | Messi Lionel | Iniesta Andrés | Falcao Radamel |
| Coach | Lithuania | Laszlo Csaba | Messi Lionel | Xavi | Özil Mesut |
| Coach | Luxembourg | Holtz Luc | Messi Lionel | Iniesta Andrés | Cristiano Ronaldo |
| Coach | Macau | Sui Wing Leung | Cristiano Ronaldo | Messi Lionel | Pirlo Andrea |
| Coach | Madagascar | Mosa Xx | Cristiano Ronaldo | Neymar | Messi Lionel |
| Coach | Malawi | Phiri Kinnah | Messi Lionel | Cristiano Ronaldo | Falcao Radamel |
| Coach | Maldives | Istvan Urbanyi | Iniesta Andrés | Cristiano Ronaldo | Messi Lionel |
| Coach | Mali | Carteron Patrice | Drogba Didier | Ibrahimovic Zlatan | Messi Lionel |
| Coach | Malta | Ghedin Pietro | Buffon Gianluigi | Falcao Radamel | Messi Lionel |
| Coach | Mauritania | Neveu Patrice | Messi Lionel | van Persie Robin | Cristiano Ronaldo |
| Coach | Mauritius | Patel Akbar | Messi Lionel | Cristiano Ronaldo | Drogba Didier |
| Coach | Mexico | De La Torre Menchaca Jose Manuel | Messi Lionel | Cristiano Ronaldo | Falcao Radamel |
| Coach | Moldova | Caras Ion | Messi Lionel | Cristiano Ronaldo | Iniesta Andrés |
| Coach | Mongolia | Sandagdorj Erdenebat | Cristiano Ronaldo | Messi Lionel | Iniesta Andrés |
| Coach | Montenegro | Brnovic Branko | Messi Lionel | Iniesta Andrés | Cristiano Ronaldo |
| Coach | Montserrat | Dyer Kenny | Messi Lionel | Xavi | Iniesta Andrés |
| Coach | Morocco | Taoussi Rachid | Messi Lionel | Cristiano Ronaldo | Ibrahimovic Zlatan |
| Coach | Mozambique | Engels Gerts | Cristiano Ronaldo | Messi Lionel | Özil Mesut |
| Coach | Myanmar | Sung Wha Park | Messi Lionel | Cristiano Ronaldo | Iniesta Andrés |
| Coach | Namibia | Kaanjuka Bernhard | Drogba Didier | Messi Lionel | Iniesta Andrés |
| Coach | Netherlands | Van Gaal Louis | Messi Lionel | Cristiano Ronaldo | Falcao Radamel |
| Coach | New Caledonia | Moizan Alain | Messi Lionel | Cristiano Ronaldo | van Persie Robin |
| Coach | New Zealand | Herbert Ricki | Iniesta Andrés | Messi Lionel | Xavi |
| Coach | Nicaragua | Llena Leon Enrique | Iniesta Andrés | Xavi | Casillas Iker |
| Coach | Niger | Gernot Rohr | Ibrahimovic Zlatan | Messi Lionel | Cristiano Ronaldo |
| Coach | Nigeria | Keshi Stephen | Touré Yaya | Drogba Didier | van Persie Robin |
| Coach | Northern Ireland | O'neill Michael | Messi Lionel | Cristiano Ronaldo | Pirlo Andrea |
| Coach | Norway | Olsen Egil | Messi Lionel | Falcao Radamel | Ibrahimovic Zlatan |
| Coach | Pakistan | Milosavljevic Zavisa | Ibrahimovic Zlatan | Messi Lionel | Casillas Iker |

| **Vote** | **Country** | **Name** | **First** **(5** **points)** | **Second** **(3** **points)** | **Third** **(1** **point)** |
| --- | --- | --- | --- | --- | --- |
| Coach | Palestine | Hamarshe Jamal | Messi Lionel | Falcao Radamel | Casillas Iker |
| Coach | Panama | Dely Julio | Messi Lionel | Cristiano Ronaldo | Neymar |
| Coach | Papua New Guinea | Farina Frank | Messi Lionel | Cristiano Ronaldo | Casillas Iker |
| Coach | Philippines | Weiss Hans Michael | Messi Lionel | Cristiano Ronaldo | Alonso Xabi |
| Coach | Poland | Fornalik Waldemar | Messi Lionel | Cristiano Ronaldo | Iniesta Andrés |
| Coach | Portugal | Bento Paulo | Cristiano Ronaldo | Falcao Radamel | Messi Lionel |
| Coach | Puerto Rico | Campos Jeaustin | Messi Lionel | Falcao Radamel | Cristiano Ronaldo |
| Coach | Republic of Ireland | Trapattoni Giovanni | Messi Lionel | Cristiano Ronaldo | Xavi |
| Coach | Romania | Pitutca Victor | Messi Lionel | Falcao Radamel | Cristiano Ronaldo |
| Coach | Russia | Capello Fabio | Messi Lionel | Pirlo Andrea | Cristiano Ronaldo |
| Coach | Rwanda | Sredojevic Micho | Messi Lionel | Cristiano Ronaldo | Ibrahimovic Zlatan |
| Coach | Samoa | Vaga Malo | Cristiano Ronaldo | Messi Lionel | Drogba Didier |
| Coach | San Marino | Giampaolo Mazza | Pirlo Andrea | Drogba Didier | Cristiano Ronaldo |
| Coach | São Tomé e Príncipe | Nyoumba Gustave | Messi Lionel | Iniesta Andrés | Cristiano Ronaldo |
| Coach | Saudi Arabia | Rijkaard Frank | Messi Lionel | Xavi | Iniesta Andrés |
| Coach | Scotland | Stark William | Iniesta Andrés | Messi Lionel | Cristiano Ronaldo |
| Coach | Senegal | Mar Mayacine | Touré Yaya | Drogba Didier | Messi Lionel |
| Coach | Serbia | Mihajlovic Siniša | Messi Lionel | Ibrahimovic Zlatan | Cristiano Ronaldo |
| Coach | Seychelles | Jeanne Gavin | Pirlo Andrea | Messi Lionel | Cristiano Ronaldo |
| Coach | Singapore | Avramovic Radojko | Messi Lionel | Cristiano Ronaldo | Pirlo Andrea |
| Coach | Slovakia | Hipp Michal | Messi Lionel | Cristiano Ronaldo | Iniesta Andrés |
| Coach | Slovenia | Stojanovic Slaviša | Cristiano Ronaldo | Messi Lionel | Xavi |
| Coach | Somalia | Ali Abdi | Messi Lionel | Neymar | Buffon Gianluigi |
| Coach | South Africa | Igesund Gordon | Messi Lionel | Xavi | Cristiano Ronaldo |
| Coach | South Sudan | Zoran Djorjvich | Drogba Didier | Iniesta Andrés | Agüero Sergio |
| Coach | Spain | Del Bosque Vicente | Casillas Iker | Xavi | Iniesta Andrés |
| Coach | Sri Lanka | Perera Sampath | Messi Lionel | Iniesta Andrés | van Persie Robin |
| Coach | Suriname | Jaliens Kenneth | Messi Lionel | Cristiano Ronaldo | Falcao Radamel |
| Coach | Swaziland | Billen Valere Josef Raymond | Messi Lionel | Cristiano Ronaldo | Iniesta Andrés |
| Coach | Sweden | Hamrén Erik | Ibrahimovic Zlatan | Casillas Iker | Cristiano Ronaldo |
| Coach | Switzerland | Hitzfeld Ottmar | Messi Lionel | Iniesta Andrés | Cristiano Ronaldo |
| Coach | Syria | Hussam Aldin Alsaed | Messi Lionel | Iniesta Andrés | Casillas Iker |
| Coach | Tahiti | Etaeta Eddy | Xavi | Messi Lionel | Drogba Didier |
| Coach | Tajikistan | Kavazovic Nikola | Messi Lionel | Cristiano Ronaldo | Drogba Didier |
| Coach | Thailand | Winfried Schaefer | Busquets Sergio | Iniesta Andrés | Cristiano Ronaldo |
| Coach | Trinidad and Tobago | Charles Hutson | Messi Lionel | Cristiano Ronaldo | Iniesta Andrés |

| **Vote** | **Country** | **Name** | **First** **(5** **points)** | **Second** **(3** **points)** | **Third** **(1** **point)** |
| --- | --- | --- | --- | --- | --- |
| Coach | Tunisia | Trabelsi Sami | Messi Lionel | Casillas Iker | Falcao Radamel |
| Coach | Turkey | Avci Abdullah | Cristiano Ronaldo | Messi Lionel | Özil Mesut |
| Coach | Turkmenistan | Hojageldiyev Yazguly | Messi Lionel | Cristiano Ronaldo | Ibrahimovic Zlatan |
| Coach | Turks and Caicos Islands | Hurdle Ian | Cristiano Ronaldo | Falcao Radamel | Pirlo Andrea |
| Coach | Uganda | Bobby Williamson Robert | Cristiano Ronaldo | Messi Lionel | Falcao Radamel |
| Coach | Ukraine | Zavarov Oleksandr | Messi Lionel | Xavi | Cristiano Ronaldo |
| Coach | United Arab Emirates | Redha Mahdi Ali | Messi Lionel | Cristiano Ronaldo | Falcao Radamel |
| Coach | Uruguay | Tabarez Oscar Washington | Messi Lionel | Cristiano Ronaldo | Iniesta Andrés |
| Coach | US Virgin Islands | Bailey Eustace | Messi Lionel | Xavi | Drogba Didier |
| Coach | USA | Klinsmann Juergen | Messi Lionel | Pirlo Andrea | Cristiano Ronaldo |
| Coach | Uzbekistan | Kasimov Mirdjalal | Messi Lionel | Iniesta Andrés | Touré Yaya |
| Coach | Vanuatu | Avock Percy | Messi Lionel | Cristiano Ronaldo | Neymar |
| Coach | Venezuela | Farias Cesar Alejandro | Casillas Iker | Messi Lionel | Falcao Radamel |
| Coach | Vietnam | Phan Thanh Hung | Messi Lionel | Cristiano Ronaldo | Xavi |
| Coach | Wales | Chris Coleman | Messi Lionel | Cristiano Ronaldo | Xavi |
| Coach | Yemen | Mebratu Abraham | Messi Lionel | Pirlo Andrea | Cristiano Ronaldo |
| Coach | Zambia | Herve Renard | Messi Lionel | Alonso Xabi | Iniesta Andrés |
| Coach | Zimbabwe | Gumbo Ruhman | Iniesta Andrés | Messi Lionel | van Persie Robin |
| Media | Albania | Besnik Dizdari | Messi Lionel | Iniesta Andrés | Cristiano Ronaldo |
| Media | Algeria | Yazid Ouahib | Messi Lionel | Cristiano Ronaldo | Xavi |
| Media | Andorra | Victor Duaso | Messi Lionel | Xavi | Iniesta Andrés |
| Media | Angola | Mateus Goncalves | Cristiano Ronaldo | Messi Lionel | Falcao Radamel |
| Media | Argentina | Enrique Sacco | Messi Lionel | Cristiano Ronaldo | Iniesta Andrés |
| Media | Armenia | Souren Baghdasarian | Iniesta Andrés | Messi Lionel | Cristiano Ronaldo |
| Media | Australia | Craig Foster | Messi Lionel | Iniesta Andrés | Cristiano Ronaldo |
| Media | Austria | Walter Kowatsch-Schwarz | Messi Lionel | Cristiano Ronaldo | Iniesta Andrés |
| Media | Azerbaijan | Rasim Mövsümov | Casillas Iker | Falcao Radamel | Drogba Didier |
| Media | Bahamas | Sheldon Longley | no vote | no vote | no vote |
| Media | Bahrain | Abdullah Ashoor | Cristiano Ronaldo | Messi Lionel | Pirlo Andrea |
| Media | Bangladesh | Raihan Mahamud | Messi Lionel | Cristiano Ronaldo | Falcao Radamel |
| Media | Barbados | Ezra Stuart | Messi Lionel | no vote | no vote |
| Media | Belarus | Sergey Nikolaev | Messi Lionel | Cristiano Ronaldo | Iniesta Andrés |
| Media | Belgium | Michel Dubois | Messi Lionel | Iniesta Andrés | Cristiano Ronaldo |
| Media | Belize | Ruben Morales Iglesias | Messi Lionel | Iniesta Andrés | Falcao Radamel |
| Media | Benin | René Sagbo | Messi Lionel | Cristiano Ronaldo | Iniesta Andrés |
| Media | Bolivia | Carlos Enrique Rivera | Messi Lionel | Cristiano Ronaldo | Iniesta Andrés |

| **Vote** | **Country** | **Name** | **First** **(5** **points)** | **Second** **(3** **points)** | **Third** **(1** **point)** |
| --- | --- | --- | --- | --- | --- |
| Media | Bosnia-Herzegovina | Ahmed Buric | Messi Lionel | Drogba Didier | Cristiano Ronaldo |
| Media | Botswana | Oaitse Mathala | Cristiano Ronaldo | Messi Lionel | Özil Mesut |
| Media | Brazil | Cleber Machado | Messi Lionel | Xavi | Cristiano Ronaldo |
| Media | Bulgaria | Michel Savov Roumen Paytashev | Falcao Radamel | Cristiano Ronaldo | Messi Lionel |
| Media | Burkina Faso | Victorien Marie Hien | Cristiano Ronaldo | Messi Lionel | Iniesta Andrés |
| Media | Burma | Kyaw Zaw Linn | no vote | no vote | no vote |
| Media | Burundi | Désiré Hatungimana | Messi Lionel | Cristiano Ronaldo | Iniesta Andrés |
| Media | Cambodia | Ung Chamroeun | Messi Lionel | Falcao Radamel | van Persie Robin |
| Media | Cameroon | Brice Mbeze | Cristiano Ronaldo | Drogba Didier | Messi Lionel |
| Media | Canada | Neil Davidson | Cristiano Ronaldo | Messi Lionel | van Persie Robin |
| Media | Cayman Islands | Ron Shillingford | Messi Lionel | Cristiano Ronaldo | Iniesta Andrés |
| Media | Central African Republic | Albert Stanislas Koumbobacko | Messi Lionel | Cristiano Ronaldo | Drogba Didier |
| Media | Chad | Alifa Hissein Atti | Messi Lionel | Cristiano Ronaldo | Ibrahimovic Zlatan |
| Media | Chile | Danilo Diaz | Messi Lionel | Iniesta Andrés | Pirlo Andrea |
| Media | China PR | Luo Ming | Iniesta Andrés | Cristiano Ronaldo | Messi Lionel |
| Media | Colombia | Paché Andrade | Falcao Radamel | Messi Lionel | Drogba Didier |
| Media | Comoros | Abdou Boina | Casillas Iker | Cristiano Ronaldo | Messi Lionel |
| Media | Congo | James Golden Eloue | Cristiano Ronaldo | Messi Lionel | Iniesta Andrés |
| Media | Congo DR | Eddy Kabelu | Messi Lionel | Iniesta Andrés | Cristiano Ronaldo |
| Media | Costa Rica | Rodrigo Antonio Calvo Castro | Messi Lionel | Iniesta Andrés | Falcao Radamel |
| Media | Côte d'Ivoire | Adam Khalil | Drogba Didier | Falcao Radamel | Iniesta Andrés |
| Media | Croatia | Zdravko Reic | Cristiano Ronaldo | Iniesta Andrés | Messi Lionel |
| Media | Cuba | Miguel Hernandez | Messi Lionel | Cristiano Ronaldo | Iniesta Andrés |
| Media | Cyprus | Michel Gavrielides | Drogba Didier | Falcao Radamel | Messi Lionel |
| Media | Czech Republic | Stanislav Hrabe | Cristiano Ronaldo | Iniesta Andrés | Messi Lionel |
| Media | Denmark | Niels Jörgen Larsen | Cristiano Ronaldo | Messi Lionel | Iniesta Andrés |
| Media | Djibouti | Kenedy Mohamed Ali | Xavi | Touré Yaya | Messi Lionel |
| Media | Dominican Republic | Jorge Rolando Bauger | Messi Lionel | Iniesta Andrés | Cristiano Ronaldo |
| Media | Ecuador | Fabricio Zavala Garcia | Messi Lionel | Cristiano Ronaldo | Falcao Radamel |
| Media | Egypt | Inas Mazhar | Cristiano Ronaldo | Messi Lionel | Iniesta Andrés |
| Media | El Salvador | Mario Ernesto Posada Flores | Casillas Iker | Messi Lionel | Falcao Radamel |
| Media | England | Henry Winter | Cristiano Ronaldo | Messi Lionel | Iniesta Andrés |
| Media | Equatorial Guinea | David Monsuy | Messi Lionel | Drogba Didier | Rooney Wayne |
| Media | Estonia | Aet Süvari | Iniesta Andrés | Messi Lionel | Cristiano Ronaldo |
| Media | Ethiopia | Mensur Abdulkeni | Messi Lionel | Cristiano Ronaldo | Falcao Radamel |
| Media | Faroe Islands | Terji Nielsen | Cristiano Ronaldo | Messi Lionel | Iniesta Andrés |

| **Vote** | **Country** | **Name** | **First** **(5** **points)** | **Second** **(3** **points)** | **Third** **(1** **point)** |
| --- | --- | --- | --- | --- | --- |
| Media | Finland | Juha Kanerva | Messi Lionel | Xavi | Neuer Manuel |
| Media | France | Gérard Ejnes | Cristiano Ronaldo | Messi Lionel | Casillas Iker |
| Media | FYR Macedonia | Boro Timkovski Mario Sotirovski | Iniesta Andrés | Cristiano Ronaldo | Messi Lionel |
| Media | Gabon | James Angelo Loundou | Casillas Iker | Messi Lionel | Cristiano Ronaldo |
| Media | Gambia | Baboucar Camara | Cristiano Ronaldo | Iniesta Andrés | Messi Lionel |
| Media | Georgia | Zurab Potskhveria Vakhtang Bzikadze | Cristiano Ronaldo | Messi Lionel | Falcao Radamel |
| Media | Germany | Karl Heinz Wild | Messi Lionel | Iniesta Andrés | Xavi |
| Media | Ghana | Michael Oti Adjei | Cristiano Ronaldo | Messi Lionel | Iniesta Andrés |
| Media | Greece | Manos Staramopoulos | Casillas Iker | Iniesta Andrés | Messi Lionel |
| Media | Grenada | Michael Bascombe | van Persie Robin | Messi Lionel | Cristiano Ronaldo |
| Media | Guatemala | Francisco Aguilar | Iniesta Andrés | Messi Lionel | Casillas Iker |
| Media | Guinea | Ibrahima Diallo | Messi Lionel | Cristiano Ronaldo | Casillas Iker |
| Media | Haiti | Enock Nere | Messi Lionel | Cristiano Ronaldo | Casillas Iker |
| Media | Honduras | Francisco Antonio Rivas Garcia | Cristiano Ronaldo | Falcao Radamel | Messi Lionel |
| Media | Hungary | Péter Csillag | Messi Lionel | Iniesta Andrés | Cristiano Ronaldo |
| Media | Iceland | Vidir Sigurdsson | Messi Lionel | Cristiano Ronaldo | Iniesta Andrés |
| Media | India | Dhiman Sarkar | Pirlo Andrea | Messi Lionel | Cristiano Ronaldo |
| Media | Indonesia | Nurdin Saleh | Messi Lionel | Cristiano Ronaldo | Iniesta Andrés |
| Media | Iran | Siamak Rahmani | Messi Lionel | Pirlo Andrea | Cristiano Ronaldo |
| Media | Iraq | Sami Abdul Emam | Messi Lionel | Cristiano Ronaldo | Falcao Radamel |
| Media | Israel | Noah Klieger Nadav Jacobi | Messi Lionel | Cristiano Ronaldo | Casillas Iker |
| Media | Italy | Paolo Condo | Iniesta Andrés | Cristiano Ronaldo | Pirlo Andrea |
| Media | Jamaica | Ian G Burnett | Messi Lionel | Iniesta Andrés | Cristiano Ronaldo |
| Media | Japan | Shuichi Tamura | Cristiano Ronaldo | Iniesta Andrés | Messi Lionel |
| Media | Jordan | Mohamed Kadre Hassan | Messi Lionel | Iniesta Andrés | Drogba Didier |
| Media | Kazakhstan | Geniy Tulegenov | Iniesta Andrés | Messi Lionel | Drogba Didier |
| Media | Kenya | Charles Nyende | Messi Lionel | Cristiano Ronaldo | Agüero Sergio |
| Media | Korea DPR | Ri Dong Gyu | Messi Lionel | Cristiano Ronaldo | Casillas Iker |
| Media | Korea Republic | Hanseok Kim | Messi Lionel | Iniesta Andrés | Cristiano Ronaldo |
| Media | Kuwait | Abd al aziz al-Attia | Messi Lionel | Cristiano Ronaldo | Buffon Gianluigi |
| Media | Kyrgyzstan | Pavel Louzanov | no vote | no vote | no vote |
| Media | Latvia | Valery Karpoushkin | Messi Lionel | Iniesta Andrés | Cristiano Ronaldo |
| Media | Lebanon | Mohamed Fawaz | Iniesta Andrés | Messi Lionel | Pirlo Andrea |
| Media | Lesotho | Thabang Matjama | Iniesta Andrés | Drogba Didier | Touré Yaya |
| Media | Liberia | J. Burgess Carter | Messi Lionel | van Persie Robin | Cristiano Ronaldo |
| Media | Libya | Joseph Cutajar Mjuftah Beilleid Hussein | Messi Lionel | Cristiano Ronaldo | van Persie Robin |

| **Vote** | **Country** | **Name** | **First** **(5** **points)** | **Second** **(3** **points)** | **Third** **(1** **point)** |
| --- | --- | --- | --- | --- | --- |
| Media | Liechtenstein | Ernst Hasler | Iniesta Andrés | Messi Lionel | Pirlo Andrea |
| Media | Lithuania | Giedrius Janonis | Cristiano Ronaldo | Messi Lionel | Iniesta Andrés |
| Media | Luxembourg | Didier Hiegel Christophe Nadin | Messi Lionel | Cristiano Ronaldo | Iniesta Andrés |
| Media | Madagascar | Clément Rabary | Messi Lionel | Drogba Didier | Neuer Manuel |
| Media | Malawi | Peter Kanjere | Messi Lionel | Iniesta Andrés | Cristiano Ronaldo |
| Media | Malaysia | Rizal Hashim | Iniesta Andrés | Casillas Iker | Pirlo Andrea |
| Media | Maldives | Shimaz Ali | Messi Lionel | Cristiano Ronaldo | Xavi |
| Media | Mali | Souleymane Bobo Tounkara | Messi Lionel | Cristiano Ronaldo | Iniesta Andrés |
| Media | Malta | Charles Camenzuli | Messi Lionel | Cristiano Ronaldo | Pirlo Andrea |
| Media | Mauritania | Mohamed Ould El Hacen | Cristiano Ronaldo | Messi Lionel | Iniesta Andrés |
| Media | Mauritius | Azmaal Hydoo | Messi Lionel | Cristiano Ronaldo | Iniesta Andrés |
| Media | Mexico | Salvador Aguilera | Messi Lionel | Iniesta Andrés | Cristiano Ronaldo |
| Media | Moldova | Sergei Donets | Pirlo Andrea | Messi Lionel | Iniesta Andrés |
| Media | Mongolia | Erdenebat Baljinnyam | Messi Lionel | Iniesta Andrés | van Persie Robin |
| Media | Montenegro | Danilo Mitrovic | Xavi | Cristiano Ronaldo | Messi Lionel |
| Media | Morocco | Mostafa Badri | Cristiano Ronaldo | Messi Lionel | Xavi |
| Media | Mozambique | Alexandre Zandamela | Messi Lionel | Xavi | Cristiano Ronaldo |
| Media | Namibia | Sheefeni Nikodemus | Messi Lionel | Iniesta Andrés | Cristiano Ronaldo |
| Media | Netherlands | Frans Van Den Nieuwenhof | Messi Lionel | Cristiano Ronaldo | Xavi |
| Media | New Caledonia | Alban Colombel | Messi Lionel | van Persie Robin | Casillas Iker |
| Media | New Zealand | Gordon Glen Watson | Cristiano Ronaldo | Messi Lionel | Pirlo Andrea |
| Media | Nicaragua | Osman Rosales Cruz | Cristiano Ronaldo | Casillas Iker | Messi Lionel |
| Media | Niger | Mohamed Silimane Ganoua | Messi Lionel | Casillas Iker | Iniesta Andrés |
| Media | Nigeria | Samm Audu | Messi Lionel | Cristiano Ronaldo | Falcao Radamel |
| Media | Northern Ireland | Jackie Fullerton Joël Taggart | Messi Lionel | Cristiano Ronaldo | Iniesta Andrés |
| Media | Norway | Arild Sandven | Messi Lionel | Pirlo Andrea | Iniesta Andrés |
| Media | Oman | Saleh Al-Barhi | Cristiano Ronaldo | Messi Lionel | Iniesta Andrés |
| Media | Palestine | Mohamed Iraqi | Cristiano Ronaldo | Messi Lionel | van Persie Robin |
| Media | Panama | Campo Elias Estrada | Messi Lionel | Cristiano Ronaldo | Xavi |
| Media | Paraguay | Ruben Dario Da Rosa | Messi Lionel | Cristiano Ronaldo | Falcao Radamel |
| Media | Peru | Carlos Salinas | Messi Lionel | Falcao Radamel | Cristiano Ronaldo |
| Media | Poland | Maciej Iwanski | Casillas Iker | Messi Lionel | Cristiano Ronaldo |
| Media | Portugal | Joaquim Rita | Cristiano Ronaldo | Messi Lionel | Iniesta Andrés |
| Media | Puerto Rico | Luis Santiago Arce | Messi Lionel | Cristiano Ronaldo | Xavi |
| Media | Qatar | Majed M. Alkhalifi | Messi Lionel | Cristiano Ronaldo | Casillas Iker |
| Media | Republic of Ireland | Paul Kelly Jimmy Magee | Cristiano Ronaldo | Iniesta Andrés | Pirlo Andrea |

| **Vote** | **Country** | **Name** | **First** **(5** **points)** | **Second** **(3** **points)** | **Third** **(1** **point)** |
| --- | --- | --- | --- | --- | --- |
| Media | Romania | Emmanuel Rosu | Messi Lionel | Falcao Radamel | Cristiano Ronaldo |
| Media | Russia | Konstantin Kleshchev | Iniesta Andrés | Cristiano Ronaldo | Messi Lionel |
| Media | Rwanda | Bonnie Mugabe | Messi Lionel | Cristiano Ronaldo | Iniesta Andrés |
| Media | San Marino | Elia Gorini | Iniesta Andrés | Pirlo Andrea | Cristiano Ronaldo |
| Media | Saudi Arabia | Rajallah Alsolami | Messi Lionel | Cristiano Ronaldo | Iniesta Andrés |
| Media | Scotland | John Greechan | Messi Lionel | Cristiano Ronaldo | Pirlo Andrea |
| Media | Senegal | Aliou Goloko | Cristiano Ronaldo | Drogba Didier | Iniesta Andrés |
| Media | Serbia | Vladimir Novak | Messi Lionel | Falcao Radamel | Cristiano Ronaldo |
| Media | Seychelles | Gérard Govinden | Messi Lionel | Cristiano Ronaldo | Xavi |
| Media | Sierra Leone | Mohamed Fajah Barrie | Messi Lionel | Cristiano Ronaldo | Iniesta Andrés |
| Media | Slovakia | Peter Surin | Cristiano Ronaldo | Messi Lionel | Iniesta Andrés |
| Media | Slovenia | Andrej Stare | Cristiano Ronaldo | Messi Lionel | Pirlo Andrea |
| Media | Somalia | Abdiaziz Godah | Cristiano Ronaldo | van Persie Robin | Xavi |
| Media | South Africa | Mark Gleeson | Messi Lionel | Cristiano Ronaldo | Iniesta Andrés |
| Media | South Sudan | John Kayanga | Iniesta Andrés | Messi Lionel | Cristiano Ronaldo |
| Media | Spain | Paco Aguilar | Iniesta Andrés | Messi Lionel | Cristiano Ronaldo |
| Media | Sudan | Muzamil Abu Elgassim | Messi Lionel | Iniesta Andrés | Cristiano Ronaldo |
| Media | Suriname | Desney Romeo | Casillas Iker | Iniesta Andrés | Pirlo Andrea |
| Media | Swaziland | Kenneth Dlamini | Messi Lionel | Iniesta Andrés | Casillas Iker |
| Media | Sweden | Henrik Ysten | Casillas Iker | Cristiano Ronaldo | Messi Lionel |
| Media | Switzerland | Pierre-Alain Dupuis | Iniesta Andrés | Cristiano Ronaldo | Casillas Iker |
| Media | Tahiti | Olivier Huc | Cristiano Ronaldo | Messi Lionel | Xavi |
| Media | Tajikistan | Alaveddine Bouriev | Cristiano Ronaldo | Messi Lionel | Casillas Iker |
| Media | Tanzania | Boniface Wambura | Cristiano Ronaldo | Messi Lionel | Iniesta Andrés |
| Media | Thailand | Urai Patoommawatana | Messi Lionel | Cristiano Ronaldo | Iniesta Andrés |
| Media | Togo | Mathias Ayena | Casillas Iker | Iniesta Andrés | Drogba Didier |
| Media | Trinidad and Tobago | Lasana Liburd | Messi Lionel | Cristiano Ronaldo | Iniesta Andrés |
| Media | Tunisia | Abdesslam Dhaifallah | Messi Lionel | Casillas Iker | Cristiano Ronaldo |
| Media | Turkey | Selçuk Manav | Iniesta Andrés | Casillas Iker | Cristiano Ronaldo |
| Media | Turkmenistan | Alexander Vershinin | Iniesta Andrés | Messi Lionel | Falcao Radamel |
| Media | Uganda | Fredrick Musisi Kiyingi | Messi Lionel | Casillas Iker | Cristiano Ronaldo |
| Media | Ukraine | Igor Linnyk | Xavi | Iniesta Andrés | Messi Lionel |
| Media | United Arab Emirates | Dafrallah Mouadhen | Messi Lionel | Xavi | Drogba Didier |
| Media | Uruguay | Ricardo Pineyrua | Messi Lionel | Pirlo Andrea | Falcao Radamel |
| Media | US Virgin Islands | Aaron Gray | no vote | no vote | no vote |
| Media | USA | Paul Kennedy | Messi Lionel | Cristiano Ronaldo | Falcao Radamel |

| **Vote** | **Country** | **Name** | **First** **(5** **points)** | **Second** **(3** **points)** | **Third** **(1** **point)** |
| --- | --- | --- | --- | --- | --- |
| Media | Uzbekistan | Grigoriy Rtveladze | Messi Lionel | Iniesta Andrés | Falcao Radamel |
| Media | Venezuela | Francisco Blavia | Messi Lionel | Cristiano Ronaldo | Falcao Radamel |
| Media | Vietnam | Truong Anh Ngoc | Messi Lionel | Cristiano Ronaldo | Pirlo Andrea |
| Media | Wales | Paul Abbandonato | Messi Lionel | Cristiano Ronaldo | Piqué Gerard |
| Media | Yemen | Abdel Al Hababi | Messi Lionel | Cristiano Ronaldo | Drogba Didier |
| Media | Zambia | Chapadongo Lungu | Messi Lionel | Cristiano Ronaldo | Touré Yaya |
| Media | Zimbabwe | Charles Mabika | Cristiano Ronaldo | Messi Lionel | Casillas Iker |

| Vote | Country | Name | First (5 points) | Second (3 points) | Third (1 point) |
| --- | --- | --- | --- | --- | --- |
| Captain | Afghanistan | Amiri Islam | Cristiano Ronaldo | Messi Lionel | Ibrahimovic Zlatan |
| Captain | Albania | Cana Lorik | Ribéry Franck | Cristiano Ronaldo | Messi Lionel |
| Captain | Algeria | Bougherra Madjid | Ribéry Franck | Cristiano Ronaldo | Ibrahimovic Zlatan |
| Captain | American Samoa | Luvu Rafe Tala | Cristiano Ronaldo | Messi Lionel | Neymar |
| Captain | Andorra | Sonejee Masand Oscar | Cristiano Ronaldo | Ribéry Franck | Messi Lionel |
| Captain | Angola | Galiano Mateus | Messi Lionel | Cristiano Ronaldo | Ribéry Franck |
| Captain | Anguilla | Connor Girdon | Messi Lionel | Cristiano Ronaldo | Neymar |
| Captain | Antigua and Barbuda | Dublin George | Cristiano Ronaldo | Ribéry Franck | Messi Lionel |
| Captain | Argentina | Messi Lionel | Iniesta Andrés | Xavi | Neymar |
| Captain | Armenia | Berezovski Roman | Messi Lionel | Cristiano Ronaldo | Ribéry Franck |
| Captain | Aruba | Breinburg Reinhard | Cristiano Ronaldo | Ibrahimovic Zlatan | Ribéry Franck |
| Captain | Australia | O'Neil Lucas | Ribéry Franck | Neymar | Cristiano Ronaldo |
| Captain | Austria | Fuchs Christian | Ribéry Franck | Lahm Philipp | Bale Gareth |
| Captain | Azerbaijan | Sadigov Rashad | Ribéry Franck | Cristiano Ronaldo | Ibrahimovic Zlatan |
| Captain | Bahamas | Wilson Phieron | Cristiano Ronaldo | Robben Arjen | Van Persie Robin |
| Captain | Bahrain | Husein Mohammed | Cristiano Ronaldo | Ribéry Franck | Messi Lionel |
| Captain | Bangladesh | Islam Mamunul | Messi Lionel | Cristiano Ronaldo | Xavi |
| Captain | Barbados | Williams Rasheed | Cristiano Ronaldo | Messi Lionel | Ribéry Franck |
| Captain | Belarus | Veremko Siarhei | Messi Lionel | Cristiano Ronaldo | Ribéry Franck |
| Captain | Belgium | Kompany Vincent | Ribéry Franck | Touré Yaya | Hazard Eden |
| Captain | Belize | Dalton Eiley | Messi Lionel | Cristiano Ronaldo | Falcao Radamel |
| Captain | Bermuda | Nusum John Barry | Messi Lionel | Cristiano Ronaldo | Ribéry Franck |
| Captain | Bhutan | Passang | Özil Mesut | Messi Lionel | Ibrahimovic Zlatan |
| Captain | Bolivia | Ráldez Ronald | Messi Lionel | Cristiano Ronaldo | Iniesta Andrés |
| Captain | Bosnia-Herzegovina | Emir Spahić | Ribéry Franck | Cristiano Ronaldo | Ibrahimovic Zlatan |
| Captain | Brazil | Da Silva Thiago Emiliano | Messi Lionel | Ibrahimovic Zlatan | Cristiano Ronaldo |
| Captain | British Virgin Islands | Davis Andy | Cristiano Ronaldo | Van Persie Robin | Xavi |
| Captain | Brunei Darussalam | Said Adi | Neymar | Messi Lionel | Cristiano Ronaldo |
| Captain | Bulgaria | Popov Ivelin | Ribéry Franck | Messi Lionel | Cristiano Ronaldo |
| Captain | Cambodia | Sok Rithy | Messi Lionel | Cristiano Ronaldo | Iniesta Andrés |
| Captain | Cameroon | Etoo Fils Samuel | Touré Yaya | Messi Lionel | Hazard Eden |
| Captain | Canada | Hutchinson Atiba | Messi Lionel | Cristiano Ronaldo | Ribéry Franck |
| Captain | Cape Verde Islands | Marco Soares | Cristiano Ronaldo | Messi Lionel | Ribéry Franck |

| Vote | Country | Name | First (5 points) | Second (3 points) | Third (1 point) |
| --- | --- | --- | --- | --- | --- |
| Captain | Cayman Islands | Lindo Ian | Messi Lionel | Cristiano Ronaldo | Ribéry Franck |
| Captain | Chile | Bravo Muñoz Claudio Andrés | Messi Lionel | Cristiano Ronaldo | Van Persie Robin |
| Captain | China PR | Zheng Zhi | Cristiano Ronaldo | Messi Lionel | Silva Thiago |
| Captain | Chinese Taipei | Lee Meng-Chian | Messi Lionel | Cristiano Ronaldo | Lahm Philipp |
| Captain | Colombia | Yepes Mario | Falcao Radamel | Ribéry Franck | Messi Lionel |
| Captain | Congo | Andzouana Kévine | Cristiano Ronaldo | Neymar | Ribéry Franck |
| Captain | Congo DR | Mulumbu Youssouf | Iniesta Andrés | Neymar | Messi Lionel |
| Captain | Cook Islands | Joseph Mii | Messi Lionel | Cristiano Ronaldo | Bale Gareth |
| Captain | Costa Rica | Ruiz Bryan | Cristiano Ronaldo | Ribéry Franck | Falcao Radamel |
| Captain | Côte d'Ivoire | Drogba Didier | Touré Yaya | Messi Lionel | Cristiano Ronaldo |
| Captain | Croatia | Srna Darijo | Cristiano Ronaldo | Messi Lionel | Ibrahimovic Zlatan |
| Captain | Cuba | Molina Marquez Odelin | Messi Lionel | Cristiano Ronaldo | Neymar |
| Captain | Curaçao | Martina Jurensley | Ibrahimovic Zlatan | Messi Lionel | Neymar |
| Captain | Cyprus | Charalambides Constantinos | Cristiano Ronaldo | Messi Lionel | Ribéry Franck |
| Captain | Czech Republic | Rosický Tomáš | Ribéry Franck | Messi Lionel | Cristiano Ronaldo |
| Captain | Denmark | Agger Daniel | Messi Lionel | Cristiano Ronaldo | Ibrahimovic Zlatan |
| Captain | Djibouti | Affasseh Abdi | Messi Lionel | Ribéry Franck | Cristiano Ronaldo |
| Captain | Dominican Republic | Rodriguez Kelvin | Ribéry Franck | Cristiano Ronaldo | Falcao Radamel |
| Captain | Ecuador | Valencia Luis Antonio | Falcao Radamel | Cristiano Ronaldo | Ribéry Franck |
| Captain | Egypt | Gomaa Wael | Cristiano Ronaldo | Ribéry Franck | Ibrahimovic Zlatan |
| Captain | El Salvador | Darwin Adelso Cerén | Cristiano Ronaldo | Messi Lionel | Ribéry Franck |
| Captain | England | Gerrard Steven | Cristiano Ronaldo | Messi Lionel | Suárez Luis |
| Captain | Eritrea | Goitom Haile | Cristiano Ronaldo | Messi Lionel | Hazard Eden |
| Captain | Estonia | Klavan Ragnar | Ribéry Franck | Messi Lionel | Van Persie Robin |
| Captain | Ethiopia | Gebreyes Degu Debebe | Xavi | Pirlo Andrea | Müller Thomas |
| Captain | Faroe Islands | Benjaminsen Fróði | Cristiano Ronaldo | Messi Lionel | Van Persie Robin |
| Captain | Fiji | Tamanisau Simione | Cristiano Ronaldo | Messi Lionel | Ribéry Franck |
| Captain | Finland | Moisander Niklas | Messi Lionel | Cristiano Ronaldo | Ibrahimovic Zlatan |
| Captain | France | Lloris Hugo | Ribéry Franck | Neuer Manuel | Bale Gareth |
| Captain | FYR Macedonia | Pandev Goran | Ribéry Franck | Cavani Edinson | Falcao Radamel |
| Captain | Gabon | Cousin Daniel | Cristiano Ronaldo | Ibrahimovic Zlatan | Ribéry Franck |
| Captain | Gambia | Jarju Mustapha | Cristiano Ronaldo | Messi Lionel | Neymar |
| Captain | Georgia | Kankava Jaba | Iniesta Andrés | Pirlo Andrea | Cavani Edinson |

| Vote | Country | Name | First (5 points) | Second (3 points) | Third (1 point) |
| --- | --- | --- | --- | --- | --- |
| Captain | Germany | Lahm Philipp | Ribéry Franck | Cristiano Ronaldo | Messi Lionel |
| Captain | Greece | Karagounis Georgios | Cristiano Ronaldo | Ribéry Franck | Touré Yaya |
| Captain | Grenada | Marc Marshall | Cristiano Ronaldo | Messi Lionel | Bale Gareth |
| Captain | Guam | Cunliffe Jason | Cristiano Ronaldo | Ribéry Franck | Messi Lionel |
| Captain | Guatemala | Contreras Jose | Messi Lionel | Cristiano Ronaldo | Pirlo Andrea |
| Captain | Guinea | Zayatte Kamil | Ribéry Franck | Cristiano Ronaldo | Messi Lionel |
| Captain | Guinea-Bissau | Nhasse Pansau | Cristiano Ronaldo | Messi Lionel | Ribéry Franck |
| Captain | Guyana | Nurse Christopher | Ribéry Franck | Van Persie Robin | Lewandowski Robert |
| Captain | Honduras | Valladares Noel | Messi Lionel | Cristiano Ronaldo | Ribéry Franck |
| Captain | Hong Kong | Chan Wai Ho | Lewandowski Robert | Cristiano Ronaldo | Falcao Radamel |
| Captain | Hungary | Juhász Roland | Ribéry Franck | Messi Lionel | Ibrahimovic Zlatan |
| Captain | Iceland | Gunnarsson Aron Einar | Cristiano Ronaldo | Messi Lionel | Ribéry Franck |
| Captain | India | Chhetri Sunil | Ribéry Franck | Cristiano Ronaldo | Iniesta Andrés |
| Captain | Indonesia | Salosa Boas | Messi Lionel | Van Persie Robin | Cristiano Ronaldo |
| Captain | Iran | Nekonam Javad | Messi Lionel | Ribéry Franck | Cristiano Ronaldo |
| Captain | Iraq | Khalaf Younis | Cristiano Ronaldo | Messi Lionel | Suárez Luis |
| Captain | Israel | Benayoun Yossi | Ribéry Franck | Messi Lionel | Cristiano Ronaldo |
| Captain | Italy | Buffon Gianluigi | Pirlo Andrea | Cristiano Ronaldo | Messi Lionel |
| Captain | Jamaica | Austin Rodolph | Ribéry Franck | Cristiano Ronaldo | Messi Lionel |
| Captain | Japan | Hasebe Makoto | Cristiano Ronaldo | Müller Thomas | Messi Lionel |
| Captain | Kazakhstan | Nurdauletov Kairat | Ribéry Franck | Müller Thomas | Lewandowski Robert |
| Captain | Kenya | Victor Mugubi Wanyama | Cristiano Ronaldo | Messi Lionel | Ribéry Franck |
| Captain | Korea DPR | Jang Song Hyok | Neymar | Cristiano Ronaldo | Messi Lionel |
| Captain | Korea Republic | Lee Chung Yong | Ribéry Franck | Cristiano Ronaldo | Messi Lionel |
| Captain | Kuwait | Al Mutawaa Bader | Messi Lionel | Özil Mesut | Xavi |
| Captain | Kyrgyzstan | Kharchenko Vadim | Van Persie Robin | Cristiano Ronaldo | Pirlo Andrea |
| Captain | Laos | Hanevilay Khampoumy | Ribéry Franck | Bale Gareth | Cristiano Ronaldo |
| Captain | Latvia | Gorkšs Kaspars | Ribéry Franck | Messi Lionel | Cristiano Ronaldo |
| Captain | Lebanon | Antar Reda | Ribéry Franck | Messi Lionel | Pirlo Andrea |
| Captain | Lesotho | Ntobo Moitheri | Cristiano Ronaldo | Ribéry Franck | Messi Lionel |
| Captain | Liberia | Wesseh Solomon | Messi Lionel | Neymar | Özil Mesut |
| Captain | Liechtenstein | Frick Mario | Ribéry Franck | Ibrahimovic Zlatan | Messi Lionel |
| Captain | Lithuania | Kijanskas Tadas | Messi Lionel | Cristiano Ronaldo | Ribéry Franck |

| Vote | Country | Name | First (5 points) | Second (3 points) | Third (1 point) |
| --- | --- | --- | --- | --- | --- |
| Captain | Luxembourg | Mutsch Mario | Cristiano Ronaldo | Ibrahimovic Zlatan | Özil Mesut |
| Captain | Macau | Che Chi Man | Cristiano Ronaldo | Pirlo Andrea | Ribéry Franck |
| Captain | Madagascar | Randrianarisoa Tahina | Silva Thiago | Bale Gareth | Messi Lionel |
| Captain | Malawi | Sangala James | Messi Lionel | Bale Gareth | Cristiano Ronaldo |
| Captain | Malaysia | Rahim Safiq | Messi Lionel | Cristiano Ronaldo | Neymar |
| Captain | Maldives | Ashfaq Ali | Messi Lionel | Cristiano Ronaldo | Iniesta Andrés |
| Captain | Mali | Keita Seydou | Ribéry Franck | Messi Lionel | Cristiano Ronaldo |
| Captain | Malta | Briffa Roderick | Iniesta Andrés | Ibrahimovic Zlatan | Lahm Philipp |
| Captain | Mauritania | Da Silva Dominique | Touré Yaya | Messi Lionel | Cristiano Ronaldo |
| Captain | Mauritius | Bell Peter Donovan Colin | Ribéry Franck | Messi Lionel | Cristiano Ronaldo |
| Captain | Mexico | Marquez Alvarez Rafael | Messi Lionel | Robben Arjen | Schweinsteiger Bastian |
| Captain | Moldova | Epureanu Alexandru | Cristiano Ronaldo | Lewandowski Robert | Ribéry Franck |
| Captain | Mongolia | Donorov Lumbengarav | Ribéry Franck | Cristiano Ronaldo | Pirlo Andrea |
| Captain | Montenegro | Vucinic Mirko | Cristiano Ronaldo | Messi Lionel | Pirlo Andrea |
| Captain | Mozambique | Khan Dario Ivan | Lahm Philipp | Cristiano Ronaldo | Lewandowski Robert |
| Captain | Myanmar | Zayar Win Kyaw | Messi Lionel | Cristiano Ronaldo | Xavi |
| Captain | Namibia | Ketjijere Ronald | Cristiano Ronaldo | Messi Lionel | Van Persie Robin |
| Captain | Nepal | Sagar Thapa | Cristiano Ronaldo | Messi Lionel | Iniesta Andrés |
| Captain | Netherlands | Van Persie Robin | Robben Arjen | Ibrahimovic Zlatan | Cristiano Ronaldo |
| Captain | New Caledonia | Dokunengo Olivier | Cristiano Ronaldo | Messi Lionel | Ibrahimovic Zlatan |
| Captain | New Zealand | Reid Winston | Bale Gareth | Neymar | Messi Lionel |
| Captain | Nicaragua | Solorzano Sanchez David | Iniesta Andrés | Ribéry Franck | Xavi |
| Captain | Niger | Ouwo Moussa Maazou | Ribéry Franck | Messi Lionel | Lahm Philipp |
| Captain | Nigeria | Enyeama Vincent | Messi Lionel | Cristiano Ronaldo | Ribéry Franck |
| Captain | Northern Ireland | Davis Steven | Messi Lionel | Cristiano Ronaldo | Ribéry Franck |
| Captain | Norway | Hangeland Brede | Schweinsteiger Bastian | Ibrahimovic Zlatan | Cristiano Ronaldo |
| Captain | Oman | Al Gheilani Hassan | Cristiano Ronaldo | Pirlo Andrea | Özil Mesut |
| Captain | Pakistan | Samar Ishaq | Cristiano Ronaldo | Messi Lionel | Robben Arjen |
| Captain | Palestine | Attal Fahed | Cristiano Ronaldo | Ribéry Franck | Xavi |
| Captain | Paraguay | Santa Cruz Roque | Ribéry Franck | Messi Lionel | Cristiano Ronaldo |
| Captain | Peru | Pizarro Claudio | Ribéry Franck | Messi Lionel | Cristiano Ronaldo |
| Captain | Philippines | Caligdong Emelio | Neymar | Messi Lionel | Özil Mesut |
| Captain | Poland | Blaszczykowski Jakub | Cristiano Ronaldo | Messi Lionel | Lewandowski Robert |

| Vote | Country | Name | First (5 points) | Second (3 points) | Third (1 point) |
| --- | --- | --- | --- | --- | --- |
| Captain | Portugal | Cristiano Ronaldo | Falcao Radamel | Bale Gareth | Özil Mesut |
| Captain | Puerto Rico | Delgado Noah | Messi Lionel | Cristiano Ronaldo | Ribéry Franck |
| Captain | Qatar | Rajab Bila | Cristiano Ronaldo | Ibrahimovic Zlatan | Messi Lionel |
| Captain | Republic of Ireland | Keane Robbie | Cristiano Ronaldo | Messi Lionel | Bale Gareth |
| Captain | Romania | Chiriches Vlad | Cristiano Ronaldo | Ribéry Franck | Ibrahimovic Zlatan |
| Captain | Russia | Shirokov Roman | Cristiano Ronaldo | Messi Lionel | Ibrahimovic Zlatan |
| Captain | Samoa | Andrew Setefano | Cristiano Ronaldo | Schweinsteiger Bastian | Messi Lionel |
| Captain | San Marino | Selva Andy | Pirlo Andrea | Messi Lionel | Ibrahimovic Zlatan |
| Captain | São Tomé e Príncipe | Das Neves Derilson | Cristiano Ronaldo | Messi Lionel | Ibrahimovic Zlatan |
| Captain | Saudi Arabia | Kariri Saud | Ribéry Franck | Messi Lionel | Cristiano Ronaldo |
| Captain | Scotland | Brown Scott | Messi Lionel | Cristiano Ronaldo | Ribéry Franck |
| Captain | Senegal | Diamé Mohamed | Messi Lionel | Ribéry Franck | Cristiano Ronaldo |
| Captain | Serbia | Ivanovic Branislav | Cristiano Ronaldo | Messi Lionel | Ribéry Franck |
| Captain | Singapore | Ishak Shahril | Cristiano Ronaldo | Özil Mesut | Messi Lionel |
| Captain | Slovakia | Skrtel Martin | Suárez Luis | Cristiano Ronaldo | Messi Lionel |
| Captain | Slovenia | Cesar Boštjan | Ibrahimovic Zlatan | Cristiano Ronaldo | Ribéry Franck |
| Captain | Solomon Islands | Farodo Henry Junior | Neymar | Pirlo Andrea | Messi Lionel |
| Captain | Somalia | Egal Yasin | Cristiano Ronaldo | Neymar | Touré Yaya |
| Captain | South Africa | Khune Itumeleng | Cristiano Ronaldo | Ribéry Franck | Touré Yaya |
| Captain | South Sudan | Richard Justin | Cristiano Ronaldo | Iniesta Andrés | Van Persie Robin |
| Captain | Spain | Casillas Iker | Cristiano Ronaldo | Ribéry Franck | Robben Arjen |
| Captain | Sri Lanka | Bandara Thilina | Messi Lionel | Neymar | Ribéry Franck |
| Captain | St. Kitts and Nevis | Harris Atiba | Messi Lionel | Cristiano Ronaldo | Özil Mesut |
| Captain | St. Lucia | Joseph Eligah | Messi Lionel | Cristiano Ronaldo | Iniesta Andrés |
| Captain | St. Vincent and the Grenadines | Stewart Cornelius | Messi Lionel | Van Persie Robin | Cristiano Ronaldo |
| Captain | Sudan | Osman Mohamed Tahir | Cristiano Ronaldo | Ribéry Franck | Messi Lionel |
| Captain | Suriname | Huiswoud Obrendo | Ribéry Franck | Cristiano Ronaldo | Messi Lionel |
| Captain | Swaziland | Tsabedze Tony | Messi Lionel | Cristiano Ronaldo | Ribéry Franck |
| Captain | Sweden | Ibrahimovic´ Zlatan | Ribéry Franck | Messi Lionel | Cristiano Ronaldo |
| Captain | Switzerland | Inler Gökhan | Cristiano Ronaldo | Messi Lionel | Ribéry Franck |
| Captain | Syria | Balhus Msab | Messi Lionel | Iniesta Andrés | Cristiano Ronaldo |
| Captain | Tahiti | Nicolas Vallar | Ibrahimovic Zlatan | Messi Lionel | Cristiano Ronaldo |

| Vote | Country | Name | First (5 points) | Second (3 points) | Third (1 point) |
| --- | --- | --- | --- | --- | --- |
| Captain | Tajikistan | Tuychiev Alisher | Messi Lionel | Ribéry Franck | Lewandowski Robert |
| Captain | Tanzania | Kaseja Juma | Cristiano Ronaldo | Messi Lionel | Van Persie Robin |
| Captain | Thailand | Hathairattanakool Sintaweechai | Ribéry Franck | Messi Lionel | Cristiano Ronaldo |
| Captain | Togo | Akakpo Serge | Messi Lionel | Cristiano Ronaldo | Ribéry Franck |
| Captain | Trinidad and Tobago | Kenwyne Jones | Cristiano Ronaldo | Ribéry Franck | Messi Lionel |
| Captain | Tunisia | Chikhaoui Yassine | Ribéry Franck | Cristiano Ronaldo | Messi Lionel |
| Captain | Turkey | Turan Arda | Özil Mesut | Cristiano Ronaldo | Messi Lionel |
| Captain | Turks and Caicos Islands | Rene James | Van Persie Robin | Messi Lionel | Suárez Luis |
| Captain | Uganda | Mwesigwa Andrew | Cristiano Ronaldo | Messi Lionel | Van Persie Robin |
| Captain | Ukraine | Rotan Ruslan | Xavi | Messi Lionel | Cristiano Ronaldo |
| Captain | United Arab Emirates | Aljneibi Ismaeil Matar | Iniesta Andrés | Neymar | Messi Lionel |
| Captain | Uruguay | Lugano Diego | Suárez Luis | Cavani Edinson | Messi Lionel |
| Captain | US Virgin Islands | Vangurp Alberto | Cristiano Ronaldo | Ibrahimovic Zlatan | Ribéry Franck |
| Captain | USA | Dempsey Clint | Cristiano Ronaldo | Messi Lionel | Ibrahimovic Zlatan |
| Captain | Uzbekistan | Djeparov Server | Messi Lionel | Cristiano Ronaldo | Ibrahimovic Zlatan |
| Captain | Vanuatu | Yelou Robert | Neymar | Cristiano Ronaldo | Ibrahimovic Zlatan |
| Captain | Venezuela | Arango Juan | Ribéry Franck | Messi Lionel | Müller Thomas |
| Captain | Vietnam | Le Tan Tai | Ribéry Franck | Messi Lionel | Cristiano Ronaldo |
| Captain | Wales | Williams Ashley | Bale Gareth | Xavi | Cristiano Ronaldo |
| Captain | Zimbabwe | Mambare Masimba | Cristiano Ronaldo | Messi Lionel | Ribéry Franck |
| Coach | Afghanistan | Kargar Mohammad Yosuf | Cristiano Ronaldo | Ibrahimovic Zlatan | Ribéry Franck |
| Coach | Albania | De Biasi Giovanni | Ibrahimovic Zlatan | Cristiano Ronaldo | Messi Lionel |
| Coach | Algeria | Halilhodzic Vahid | Ribéry Franck | Ibrahimovic Zlatan | Messi Lionel |
| Coach | American Samoa | Aliva Uinifareti | Messi Lionel | Cristiano Ronaldo | Neymar |
| Coach | Andorra | Alvarez De Eulate Jesús Luís | Messi Lionel | Cristiano Ronaldo | Ribéry Franck |
| Coach | Angola | Kilamba José | Messi Lionel | Cristiano Ronaldo | Robben Arjen |
| Coach | Anguilla | Johnson Colin | Messi Lionel | Cristiano Ronaldo | Neymar |
| Coach | Antigua and Barbuda | Williams Rolston | Ribéry Franck | Van Persie Robin | Neuer Manuel |
| Coach | Argentina | Sabella Alejandro | Messi Lionel | Ribéry Franck | Cristiano Ronaldo |
| Coach | Armenia | Minasyan Vardan | Cristiano Ronaldo | Ribéry Franck | Pirlo Andrea |
| Coach | Aruba | Franken Giovanni | Cristiano Ronaldo | Ibrahimovic Zlatan | Ribéry Franck |
| Coach | Australia | Postecoglou Ange | Ribéry Franck | Neymar | Cristiano Ronaldo |
| Coach | Austria | Koller Marcel | Ribéry Franck | Lahm Philipp | Ibrahimovic Zlatan |

| Vote | Country | Name | First (5 points) | Second (3 points) | Third (1 point) |
| --- | --- | --- | --- | --- | --- |
| Coach | Azerbaijan | Vogts Hans Hubert | Cristiano Ronaldo | Lahm Philipp | Lewandowski Robert |
| Coach | Bahamas | Niko Mosko | Robben Arjen | Messi Lionel | Cristiano Ronaldo |
| Coach | Bahrain | Hudson Anthony | Schweinsteiger Bastian | Pirlo Andrea | Özil Mesut |
| Coach | Bangladesh | Kruif Lodewijk Darius De | Messi Lionel | Cristiano Ronaldo | Ibrahimovic Zlatan |
| Coach | Barbados | Forde Colin | Ribéry Franck | Ibrahimovic Zlatan | Messi Lionel |
| Coach | Belarus | Kandratsyeu Heorhi | Ribéry Franck | Messi Lionel | Cristiano Ronaldo |
| Coach | Belgium | Wilmots Marc | Ibrahimovic Zlatan | Lewandowski Robert | Hazard Eden |
| Coach | Belize | Ian Mork | Messi Lionel | Cristiano Ronaldo | Falcao Radamel |
| Coach | Bermuda | Bascome Andrew | Messi Lionel | Cristiano Ronaldo | Cavani Edinson |
| Coach | Bhutan | Khandu Dorji | Müller Thomas | Messi Lionel | Xavi |
| Coach | Bolivia | Azcargorta Uriarte Francisco Javier | Iniesta Andrés | Messi Lionel | Cristiano Ronaldo |
| Coach | Bosnia-Herzegovina | Safet Sušić | Ribéry Franck | Cristiano Ronaldo | Ibrahimovic Zlatan |
| Coach | Brazil | Scolari Thiago | Cristiano Ronaldo | Messi Lionel | Ibrahimovic Zlatan |
| Coach | British Virgin Islands | Williams Avondale | Ribéry Franck | Messi Lionel | Cristiano Ronaldo |
| Coach | Brunei Darussalam | Oh Son Kwon | Neymar | Cristiano Ronaldo | Hazard Eden |
| Coach | Bulgaria | Penev Lyuboslav | Ribéry Franck | Lewandowski Robert | Lahm Philipp |
| Coach | Cambodia | Tae Hoon Lee | Messi Lionel | Cristiano Ronaldo | Touré Yaya |
| Coach | Cameroon | Finke Volker | Ribéry Franck | Cristiano Ronaldo | Iniesta Andrés |
| Coach | Canada | Floro Sanz Benito | Cristiano Ronaldo | Iniesta Andrés | Xavi |
| Coach | Cape Verde Islands | Lucio Antunes | Cristiano Ronaldo | Messi Lionel | Ibrahimovic Zlatan |
| Coach | Cayman Islands | Tinoco Marcos | Cristiano Ronaldo | Neymar | Ribéry Franck |
| Coach | Chile | Sampaoli Moya Jorge Luis | Cristiano Ronaldo | Messi Lionel | Robben Arjen |
| Coach | China PR | Fu Bo | Silva Thiago | Cristiano Ronaldo | Messi Lionel |
| Coach | Chinese Taipei | Chen Kuei-Jen | Messi Lionel | Cristiano Ronaldo | Neymar |
| Coach | Colombia | Pekerman Jose | Falcao Radamel | Messi Lionel | Ibrahimovic Zlatan |
| Coach | Congo | Kamel Djabour | Cristiano Ronaldo | Iniesta Andrés | Ribéry Franck |
| Coach | Congo DR | Muntubile Ndjela Santos | Messi Lionel | Iniesta Andrés | Neymar |
| Coach | Cook Islands | Tisam Tuka | Messi Lionel | Cristiano Ronaldo | Bale Gareth |
| Coach | Costa Rica | Pinto Jorge | Cristiano Ronaldo | Ribéry Franck | Neymar |
| Coach | Côte d'Ivoire | Lamouchi Sabri | Ribéry Franck | Cristiano Ronaldo | Touré Yaya |
| Coach | Croatia | Kovac Niko | Ribéry Franck | Ibrahimovic Zlatan | Lewandowski Robert |
| Coach | Cuba | Benitez Rosales Walter Manuel | Messi Lionel | Cristiano Ronaldo | Neymar |
| Coach | Curaçao | Alberto Ludwig | Messi Lionel | Neymar | Bale Gareth |

| Vote | Country | Name | First (5 points) | Second (3 points) | Third (1 point) |
| --- | --- | --- | --- | --- | --- |
| Coach | Cyprus | Hadjipieris Michalis | Cristiano Ronaldo | Messi Lionel | Ribéry Franck |
| Coach | Czech Republic | Bílek Michal | Ribéry Franck | Messi Lionel | Cristiano Ronaldo |
| Coach | Denmark | Olsen Morten | Ibrahimovic Zlatan | Messi Lionel | Cristiano Ronaldo |
| Coach | Djibouti | Mohamed Houssein | Messi Lionel | Ribéry Franck | Ibrahimovic Zlatan |
| Coach | Dominica | Gustarve Ronnie | Neymar | Cristiano Ronaldo | Messi Lionel |
| Coach | Dominican Republic | Hernandez Herez Domingo | Ribéry Franck | Cristiano Ronaldo | Falcao Radamel |
| Coach | Ecuador | Rueda Rivera Reinaldo | Messi Lionel | Falcao Radamel | Cristiano Ronaldo |
| Coach | Egypt | Bradley Bob | Cristiano Ronaldo | Ribéry Franck | Ibrahimovic Zlatan |
| Coach | El Salvador | Castillo Agustin | Ribéry Franck | Cristiano Ronaldo | Messi Lionel |
| Coach | England | Hodgson Roy | Cristiano Ronaldo | Ibrahimovic Zlatan | Van Persie Robin |
| Coach | Eritrea | Teklit Negash | Cristiano Ronaldo | Ribéry Franck | Suárez Luis |
| Coach | Estonia | Rüütli Tarmo | Cristiano Ronaldo | Messi Lionel | Ribéry Franck |
| Coach | Ethiopia | Woube Sewnet Bishaw | Cristiano Ronaldo | Iniesta Andrés | Neymar |
| Coach | Faroe Islands | Olsen Lars | Ibrahimovic Zlatan | Cristiano Ronaldo | Messi Lionel |
| Coach | Fiji | Buzzetti Carlos Juan | Cristiano Ronaldo | Ribéry Franck | Lewandowski Robert |
| Coach | Finland | Paatelainen Mixu | Messi Lionel | Ibrahimovic Zlatan | Cristiano Ronaldo |
| Coach | France | Deschamps Didier | Ribéry Franck | Cristiano Ronaldo | Ibrahimovic Zlatan |
| Coach | FYR Macedonia | Stratev Zoran | Messi Lionel | Ribéry Franck | Cristiano Ronaldo |
| Coach | Gabon | Duarte Paulo | Cristiano Ronaldo | Falcao Radamel | Iniesta Andrés |
| Coach | Gambia | Johnson Peter Bonu | Cristiano Ronaldo | Messi Lionel | Neymar |
| Coach | Georgia | Ketsbaia Temur | Ribéry Franck | Messi Lionel | Cristiano Ronaldo |
| Coach | Greece | Santos Fernando | Cristiano Ronaldo | Ribéry Franck | Messi Lionel |
| Coach | Grenada | John Clarke | Cristiano Ronaldo | Van Persie Robin | Müller Thomas |
| Coach | Guam | White Gary | Messi Lionel | Van Persie Robin | Bale Gareth |
| Coach | Guatemala | Funes Juan Manuel | Iniesta Andrés | Messi Lionel | Pirlo Andrea |
| Coach | Guinea | Dussuyer Michel | Cristiano Ronaldo | Messi Lionel | Ibrahimovic Zlatan |
| Coach | Guinea-Bissau | Mané Abu Bacar | Cristiano Ronaldo | Ribéry Franck | Iniesta Andrés |
| Coach | Guyana | Dover Wayne | Ribéry Franck | Van Persie Robin | Lewandowski Robert |
| Coach | Honduras | Suarez Luis Fernando | Messi Lionel | Cristiano Ronaldo | Ibrahimovic Zlatan |
| Coach | Hong Kong | Kim Pangon | Van Persie Robin | Cristiano Ronaldo | Robben Arjen |
| Coach | Iceland | Lagerback Lars | Ibrahimovic Zlatan | Cristiano Ronaldo | Messi Lionel |
| Coach | India | Koevermans Wilhelmus Jacobus | Iniesta Andrés | Van Persie Robin | Messi Lionel |
| Coach | Indonesia | F Tiago Jacksen | Cristiano Ronaldo | Messi Lionel | Van Persie Robin |

| Vote | Country | Name | First (5 points) | Second (3 points) | Third (1 point) |
| --- | --- | --- | --- | --- | --- |
| Coach | Iran | Brito Leal De Queiroz Carlos Manuel | Messi Lionel | Cristiano Ronaldo | Ribéry Franck |
| Coach | Iraq | Al Azzawi Hakeem | Messi Lionel | Cristiano Ronaldo | Pirlo Andrea |
| Coach | Israel | Gutman Eli | Lahm Philipp | Ribéry Franck | Robben Arjen |
| Coach | Italy | Prandelli Cesare Claudio | Pirlo Andrea | Lahm Philipp | Lewandowski Robert |
| Coach | Jamaica | Schafer Winfried | Lewandowski Robert | Ribéry Franck | Xavi |
| Coach | Japan | Zaccheroni Alberto | Cristiano Ronaldo | Ibrahimovic Zlatan | Messi Lionel |
| Coach | Kazakhstan | Beranek Miroslav | Ribéry Franck | Messi Lionel | Ibrahimovic Zlatan |
| Coach | Kenya | Adel Amrouche | Cristiano Ronaldo | Ribéry Franck | Touré Yaya |
| Coach | Korea DPR | Yun Jong Su | Messi Lionel | Neymar | Ribéry Franck |
| Coach | Korea Republic | Hong Myung Bo | Ribéry Franck | Lewandowski Robert | Neymar |
| Coach | Kuwait | Vieira Jorvan | Messi Lionel | Neymar | Cristiano Ronaldo |
| Coach | Kyrgyzstan | Dvoryankov Sergey | Cristiano Ronaldo | Messi Lionel | Robben Arjen |
| Coach | Laos | Kimura Kokichi | Bale Gareth | Ribéry Franck | Cristiano Ronaldo |
| Coach | Latvia | Pahars Marians | Messi Lionel | Ribéry Franck | Cristiano Ronaldo |
| Coach | Lebanon | Gianinni Giuseppe | Cavani Edinson | Pirlo Andrea | Xavi |
| Coach | Lesotho | Notsi Leslie | Cristiano Ronaldo | Messi Lionel | Ribéry Franck |
| Coach | Liberia | Kojo Thomas | Messi Lionel | Iniesta Andrés | Ibrahimovic Zlatan |
| Coach | Liechtenstein | Pauritsch Rene | Ribéry Franck | Falcao Radamel | Ibrahimovic Zlatan |
| Coach | Lithuania | Pankretjevas Igoris | Ribéry Franck | Cristiano Ronaldo | Ibrahimovic Zlatan |
| Coach | Luxembourg | Holtz Luc | Messi Lionel | Cristiano Ronaldo | Iniesta Andrés |
| Coach | Macau | Leung Sui Wing | Cristiano Ronaldo | Messi Lionel | Ribéry Franck |
| Coach | Madagascar | Rajaonarisamba Franck | Ribéry Franck | Neymar | Messi Lionel |
| Coach | Malawi | Kaputa John | Bale Gareth | Van Persie Robin | Cristiano Ronaldo |
| Coach | Malaysia | R. Krishnasamy Rajagobal | Messi Lionel | Cristiano Ronaldo | Ribéry Franck |
| Coach | Maldives | Nashid Ali | Ribéry Franck | Cristiano Ronaldo | Messi Lionel |
| Coach | Mali | Diallo Amadou Pathé | Cristiano Ronaldo | Messi Lionel | Touré Yaya |
| Coach | Malta | Ghedin Pietro | Pirlo Andrea | Messi Lionel | Cristiano Ronaldo |
| Coach | Mauritania | Neveu Patrice | Ribéry Franck | Van Persie Robin | Messi Lionel |
| Coach | Mauritius | Patel Ebrahim Akbar | Ribéry Franck | Neymar | Van Persie Robin |
| Coach | Mexico | Herrera Aguirre Miguel Ernesto | Ribéry Franck | Robben Arjen | Lahm Philipp |
| Coach | Moldova | Caras Ion | Ribéry Franck | Cristiano Ronaldo | Ibrahimovic Zlatan |
| Coach | Mongolia | Sandagdorj Erdenebat | Cristiano Ronaldo | Ribéry Franck | Ibrahimovic Zlatan |
| Coach | Montenegro | Brnovic Branko | Messi Lionel | Ibrahimovic Zlatan | Cristiano Ronaldo |

| Vote | Country | Name | First (5 points) | Second (3 points) | Third (1 point) |
| --- | --- | --- | --- | --- | --- |
| Coach | Montserrat | Hewlett Lenny | Cristiano Ronaldo | Messi Lionel | Touré Yaya |
| Coach | Mozambique | Chissano João Antonio | Ribéry Franck | Messi Lionel | Cristiano Ronaldo |
| Coach | Myanmar | Sung Wah Park | Messi Lionel | Cristiano Ronaldo | Ribéry Franck |
| Coach | Namibia | Mannetti Ricardo | Messi Lionel | Cristiano Ronaldo | Van Persie Robin |
| Coach | Nepal | Jack Stefanowski | Ribéry Franck | Messi Lionel | Cristiano Ronaldo |
| Coach | Netherlands | Van Gaal Louis | Ribéry Franck | Müller Thomas | Robben Arjen |
| Coach | New Caledonia | Moizan Alain | Cristiano Ronaldo | Messi Lionel | Ibrahimovic Zlatan |
| Coach | New Zealand | Herbert Ricky | Bale Gareth | Neymar | Messi Lionel |
| Coach | Nicaragua | Llena Enrique | Iniesta Andrés | Xavi | Ribéry Franck |
| Coach | Niger | Gernot Rohr | Ribéry Franck | Messi Lionel | Lahm Philipp |
| Coach | Nigeria | Keshi Stephen | Touré Yaya | Ribéry Franck | Suárez Luis |
| Coach | Northern Ireland | O'Neill Michael | Cristiano Ronaldo | Messi Lionel | Ribéry Franck |
| Coach | Norway | Høgmo Per Mathias | Ibrahimovic Zlatan | Cristiano Ronaldo | Messi Lionel |
| Coach | Oman | Le Guen Paul | Cristiano Ronaldo | Ribéry Franck | Lahm Philipp |
| Coach | Pakistan | Shamlan Mohamed | Cristiano Ronaldo | Ribéry Franck | Schweinsteiger Bastian |
| Coach | Palestine | Mahmoud Jamal | Cristiano Ronaldo | Özil Mesut | Schweinsteiger Bastian |
| Coach | Paraguay | Genes Victor | Cristiano Ronaldo | Messi Lionel | Iniesta Andrés |
| Coach | Peru | Markarian Sergio | Messi Lionel | Ribéry Franck | Cavani Edinson |
| Coach | Philippines | Weiss Hans Michael | Cristiano Ronaldo | Özil Mesut | Schweinsteiger Bastian |
| Coach | Poland | Nawalka Adam | Lewandowski Robert | Hazard Eden | Messi Lionel |
| Coach | Portugal | Bento Paulo | Cristiano Ronaldo | Falcao Radamel | Robben Arjen |
| Coach | Puerto Rico | Campos Jeaustin | Ribéry Franck | Cristiano Ronaldo | Falcao Radamel |
| Coach | Qatar | Al Zarraa Fahad | Cristiano Ronaldo | Messi Lionel | Falcao Radamel |
| Coach | Republic of Ireland | O'Neill Martin | Messi Lionel | Cristiano Ronaldo | Bale Gareth |
| Coach | Romania | Piturca Victor | Messi Lionel | Cristiano Ronaldo | Robben Arjen |
| Coach | Russia | Capello Fabio | Messi Lionel | Cristiano Ronaldo | Ibrahimovic Zlatan |
| Coach | Samoa | Vaga Malo | Cristiano Ronaldo | Messi Lionel | Cavani Edinson |
| Coach | San Marino | Mazza Giampaolo | Robben Arjen | Pirlo Andrea | Neymar |
| Coach | São Tomé e Príncipe | Nyoumba Gustave | Cristiano Ronaldo | Messi Lionel | Falcao Radamel |
| Coach | Saudi Arabia | Lopez Caro Juan Ramon | Iniesta Andrés | Messi Lionel | Cristiano Ronaldo |
| Coach | Scotland | Strachan Gordon | Messi Lionel | Cristiano Ronaldo | Suárez Luis |
| Coach | Senegal | Giresse Alain | Messi Lionel | Ribéry Franck | Iniesta Andrés |
| Coach | Serbia | Mihajlovic Siniša | Ibrahimovic Zlatan | Cristiano Ronaldo | Messi Lionel |

| Vote | Country | Name | First (5 points) | Second (3 points) | Third (1 point) |
| --- | --- | --- | --- | --- | --- |
| Coach | Singapore | Stange Bernd | Ribéry Franck | Messi Lionel | Cristiano Ronaldo |
| Coach | Slovakia | Kozak Jan | Cristiano Ronaldo | Ibrahimovic Zlatan | Ribéry Franck |
| Coach | Slovenia | Katanec Srečko | Ibrahimovic Zlatan | Messi Lionel | Cristiano Ronaldo |
| Coach | Solomon Islands | Moli Jacob | Neymar | Messi Lionel | Cristiano Ronaldo |
| Coach | Somalia | Abdulle Farayare | Cristiano Ronaldo | Neymar | Touré Yaya |
| Coach | South Africa | Igesung Gordon | Cristiano Ronaldo | Messi Lionel | Neymar |
| Coach | South Sudan | Salyi Samuel | Cristiano Ronaldo | Iniesta Andrés | Touré Yaya |
| Coach | Spain | Del Bosque Vicente | Xavi | Iniesta Andrés | Ribéry Franck |
| Coach | Sri Lanka | Perera Sampath | Messi Lionel | Bale Gareth | Iniesta Andrés |
| Coach | St. Kitts and Nevis | Hazel Jeffrey | Ribéry Franck | Messi Lionel | Cristiano Ronaldo |
| Coach | St. Lucia | Lastic Francis | Messi Lionel | Cristiano Ronaldo | Iniesta Andrés |
| Coach | St. Vincent and the Grenadines | Huggins Cornelius | Messi Lionel | Van Persie Robin | Cristiano Ronaldo |
| Coach | Sudan | Magzoub Mohamed Abdalla Ahmed | Cristiano Ronaldo | Ribéry Franck | Messi Lionel |
| Coach | Suriname | Godeken Roberto | Cristiano Ronaldo | Robben Arjen | Ibrahimovic Zlatan |
| Coach | Swaziland | Bulunga Harris | Cristiano Ronaldo | Messi Lionel | Ribéry Franck |
| Coach | Sweden | Hamrén Erik | Ibrahimovic Zlatan | Messi Lionel | Cristiano Ronaldo |
| Coach | Switzerland | Hitzfeld Ottmar | Ribéry Franck | Messi Lionel | Cristiano Ronaldo |
| Coach | Syria | Alsayed Husam | Messi Lionel | Ribéry Franck | Cristiano Ronaldo |
| Coach | Tahiti | Etaeta Eddy | Ribéry Franck | Messi Lionel | Neymar |
| Coach | Tajikistan | Muhamadiev Mukhsin | Ribéry Franck | Messi Lionel | Cristiano Ronaldo |
| Coach | Tanzania | Poulsen Kim | Messi Lionel | Cristiano Ronaldo | Ribéry Franck |
| Coach | Thailand | Jaturapattarapong Surachai | Van Persie Robin | Cristiano Ronaldo | Ribéry Franck |
| Coach | Togo | Six Didier | Ribéry Franck | Cristiano Ronaldo | Ibrahimovic Zlatan |
| Coach | Trinidad and Tobago | Stephen Hart | Cristiano Ronaldo | Ribéry Franck | Messi Lionel |
| Coach | Tunisia | Krol Ruud | Ibrahimovic Zlatan | Messi Lionel | Cristiano Ronaldo |
| Coach | Turkey | Terim Fatih | Özil Mesut | Cristiano Ronaldo | Messi Lionel |
| Coach | Turks and Caicos Islands | Flanagan Jon | Messi Lionel | Van Persie Robin | Xavi |
| Coach | Uganda | Sredojevic Milutin | Cristiano Ronaldo | Messi Lionel | Van Persie Robin |
| Coach | Ukraine | Fomenko Mikhail | Cristiano Ronaldo | Messi Lionel | Ribéry Franck |
| Coach | United Arab Emirates | Redha Mahdi Ali | Ribéry Franck | Messi Lionel | Cristiano Ronaldo |
| Coach | Uruguay | Tabárez Oscar | Ribéry Franck | Messi Lionel | Cristiano Ronaldo |
| Coach | US Virgin Islands | Bailey Eustace | Cristiano Ronaldo | Neymar | Ibrahimovic Zlatan |

| Vote | Country | Name | First (5 points) | Second (3 points) | Third (1 point) |
| --- | --- | --- | --- | --- | --- |
| Coach | USA | Klinsmann Jurgen | Ribéry Franck | Bale Gareth | Falcao Radamel |
| Coach | Uzbekistan | Kasimov Mirjalol | Ribéry Franck | Ibrahimovic Zlatan | Messi Lionel |
| Coach | Vanuatu | Avock Percy | Neymar | Cristiano Ronaldo | Messi Lionel |
| Coach | Venezuela | Farias Acosta Cesar Alejandro | Messi Lionel | Falcao Radamel | Cavani Edinson |
| Coach | Vietnam | Hoang Van Phuc | Ribéry Franck | Messi Lionel | Cristiano Ronaldo |
| Coach | Wales | Coleman Chris | Bale Gareth | Messi Lionel | Cristiano Ronaldo |
| Coach | Zimbabwe | Gorowa Ian | Cristiano Ronaldo | Messi Lionel | Ribéry Franck |
| Media | Albania | Dizdari Besnik | Ribéry Franck | Messi Lionel | Neymar |
| Media | Algeria | Ouahib Yazid | Messi Lionel | Robben Arjen | Cristiano Ronaldo |
| Media | Andorra | Duaso Victor | Messi Lionel | Ribéry Franck | Lewandowski Robert |
| Media | Angola | Goncalves Mateus | Cristiano Ronaldo | Ribéry Franck | Messi Lionel |
| Media | Antigua and Barbuda | Neto Baptiste | Cristiano Ronaldo | Messi Lionel | Ribéry Franck |
| Media | Argentina | Sacco Enrique | Messi Lionel | Cristiano Ronaldo | Ribéry Franck |
| Media | Armenia | Baghdasarian Souren | Ribéry Franck | Messi Lionel | Cristiano Ronaldo |
| Media | Australia | Foster Craig | Ribéry Franck | Cristiano Ronaldo | Messi Lionel |
| Media | Austria | Kowatsch-Schwarz Walter | Ribéry Franck | Cristiano Ronaldo | Ibrahimovic Zlatan |
| Media | Azerbaijan | Mövsümov Rasim | Ribéry Franck | Cristiano Ronaldo | Ibrahimovic Zlatan |
| Media | Bahamas | Longley Sheldon | Messi Lionel | Van Persie Robin | Neymar |
| Media | Bahrain | Ashoor Abdullah | Ribéry Franck | Cristiano Ronaldo | Ibrahimovic Zlatan |
| Media | Bangladesh | Mahamud Raihan | Ribéry Franck | Cristiano Ronaldo | Messi Lionel |
| Media | Belarus | Nikolaev Sergey | Ribéry Franck | Cristiano Ronaldo | Messi Lionel |
| Media | Belgium | Dubois Michel | Ribéry Franck | Ibrahimovic Zlatan | Cristiano Ronaldo |
| Media | Belize | Morales Iglesias Ruben | Messi Lionel | Neymar | Falcao Radamel |
| Media | Benin | Sagbo René | Cristiano Ronaldo | Ribéry Franck | Messi Lionel |
| Media | Bermuda | Burton James | Messi Lionel | Cristiano Ronaldo | Ribéry Franck |
| Media | Bhutan | Wangdi Phuntsho | Cristiano Ronaldo | Messi Lionel | Müller Thomas |
| Media | Bolivia | Rivera Carlos Enrique | Messi Lionel | Ribéry Franck | Cavani Edinson |
| Media | Bosnia-Herzegovina | Buric Ahmed | Ibrahimovic Zlatan | Cristiano Ronaldo | Robben Arjen |
| Media | Botswana | Mathala Oaitse | Messi Lionel | Cristiano Ronaldo | Iniesta Andrés |
| Media | Brazil | Machado Cleber | Ribéry Franck | Cristiano Ronaldo | Schweinsteiger Bastian |
| Media | Bulgaria | Savov Michel | Ribéry Franck | Ibrahimovic Zlatan | Cristiano Ronaldo |
| Media | Burkina Faso | Marie Hien Victorien | Iniesta Andrés | Ribéry Franck | Ibrahimovic Zlatan |
| Media | Burundi | Hatungimana Desiré | Ribéry Franck | Messi Lionel | Van Persie Robin |

| Vote | Country | Name | First (5 points) | Second (3 points) | Third (1 point) |
| --- | --- | --- | --- | --- | --- |
| Media | Cambodia | Chamroeun Ung | Ribéry Franck | Ibrahimovic Zlatan | Özil Mesut |
| Media | Cameroon | Mbeze Brice | Cristiano Ronaldo | Ribéry Franck | Ibrahimovic Zlatan |
| Media | Canada | Davidson Neil | Cristiano Ronaldo | Messi Lionel | Ribéry Franck |
| Media | Cape Verde Islands | Amaral André | Cristiano Ronaldo | Xavi | Messi Lionel |
| Media | Cayman Islands | Shillingford Ron | Messi Lionel | Cristiano Ronaldo | Neymar |
| Media | Chad | Hissein Atti Alifa | Ribéry Franck | Touré Yaya | Cristiano Ronaldo |
| Media | Chile | Diaz Danilo | Ribéry Franck | Messi Lionel | Falcao Radamel |
| Media | China PR | Ming Luo | Ribéry Franck | Messi Lionel | Cristiano Ronaldo |
| Media | Colombia | Andrade Paché | Ribéry Franck | Falcao Radamel | Cristiano Ronaldo |
| Media | Comoros | Boina Abdou | Cristiano Ronaldo | Ribéry Franck | Ibrahimovic Zlatan |
| Media | Congo | Golden Eloue James | Ribéry Franck | Robben Arjen | Cristiano Ronaldo |
| Media | Congo DR | Kabelu Eddy | Ribéry Franck | Messi Lionel | Cristiano Ronaldo |
| Media | Costa Rica | Calvo Castro Rodrigo | Ribéry Franck | Ibrahimovic Zlatan | Neymar |
| Media | Côte d'Ivoire | Khalil Adam | Touré Yaya | Ribéry Franck | Silva Thiago |
| Media | Croatia | Reic Zdravko | Ibrahimovic Zlatan | Ribéry Franck | Messi Lionel |
| Media | Cuba | Hernandez Miguel | Messi Lionel | Ribéry Franck | Cristiano Ronaldo |
| Media | Curaçao | Dunker Nino | Ribéry Franck | Neymar | Messi Lionel |
| Media | Cyprus | Gavrielides Michel | Ribéry Franck | Lahm Philipp | Cristiano Ronaldo |
| Media | Czech Republic | Hrabe Stanislav | Ribéry Franck | Cristiano Ronaldo | Ibrahimovic Zlatan |
| Media | Denmark | Bager Thogersen Troels | Ribéry Franck | Cristiano Ronaldo | Messi Lionel |
| Media | Djibouti | Mohamed Kenadid | Messi Lionel | Bale Gareth | Xavi |
| Media | Dominican Republic | Bauger Jorge Rolando | Ibrahimovic Zlatan | Ribéry Franck | Cristiano Ronaldo |
| Media | Ecuador | Zavala Garcia Fabricio | Messi Lionel | Cristiano Ronaldo | Ibrahimovic Zlatan |
| Media | Egypt | Mazhar Inas | Cristiano Ronaldo | Messi Lionel | Ibrahimovic Zlatan |
| Media | El Salvador | Posada Mario Ernesto | Ribéry Franck | Robben Arjen | Xavi |
| Media | England | Winter Henry | Ribéry Franck | Messi Lionel | Cristiano Ronaldo |
| Media | Equatorial Guinea | Monsuy David | Messi Lionel | Cristiano Ronaldo | Touré Yaya |
| Media | Eritrea | Seium Michael | Neymar | Touré Yaya | Ibrahimovic Zlatan |
| Media | Estonia | Süvari Aet | Messi Lionel | Cristiano Ronaldo | Schweinsteiger Bastian |
| Media | Ethiopia | Abdulkeni Mensur | Ribéry Franck | Cristiano Ronaldo | Messi Lionel |
| Media | Finland | Kanerva Juha | Ibrahimovic Zlatan | Messi Lionel | Lahm Philipp |
| Media | France | Ejnes Gérard | Ribéry Franck | Cristiano Ronaldo | Messi Lionel |
| Media | FYR Macedonia | Timkovski Boro | Ribéry Franck | Messi Lionel | Cristiano Ronaldo |

| Vote | Country | Name | First (5 points) | Second (3 points) | Third (1 point) |
| --- | --- | --- | --- | --- | --- |
| Media | Gabon | Loundou James Angelo | Ribéry Franck | Cristiano Ronaldo | Neymar |
| Media | Gambia | Camara Baboucar | Cristiano Ronaldo | Ribéry Franck | Messi Lionel |
| Media | Georgia | Potskhveria Zurab | Cristiano Ronaldo | Messi Lionel | Ribéry Franck |
| Media | Germany | Wild Karlheinz | Ribéry Franck | Lahm Philipp | Ibrahimovic Zlatan |
| Media | Ghana | Oti Adjei Michael | Cristiano Ronaldo | Ribéry Franck | Messi Lionel |
| Media | Greece | Staramopoulos Manos | Ribéry Franck | Neymar | Robben Arjen |
| Media | Grenada | Bascombe Michael | Van Persie Robin | Messi Lionel | Touré Yaya |
| Media | Guatemala | Aguilar Francisco | Robben Arjen | Ribéry Franck | Schweinsteiger Bastian |
| Media | Guinea | Diallo Ibrahima | Ribéry Franck | Cristiano Ronaldo | Messi Lionel |
| Media | Guinea-Bissau | Ucha Etiandro | Cristiano Ronaldo | Pirlo Andrea | Ribéry Franck |
| Media | Guyana | Campbell Emmerson | Messi Lionel | Ribéry Franck | Cristiano Ronaldo |
| Media | Haiti | Nere Enock | Ribéry Franck | Messi Lionel | Cristiano Ronaldo |
| Media | Honduras | Garcia Francisco Antonio Rivas | Ribéry Franck | Messi Lionel | Ibrahimovic Zlatan |
| Media | Hungary | Csillag Péter | Messi Lionel | Ribéry Franck | Lahm Philipp |
| Media | Iceland | Sigurdsson Vidir | Cristiano Ronaldo | Messi Lionel | Ribéry Franck |
| Media | India | Sarkar Dhiman | Cristiano Ronaldo | Ribéry Franck | Van Persie Robin |
| Media | Indonesia | Saleh Nurdin | Messi Lionel | Ribéry Franck | Cristiano Ronaldo |
| Media | Iran | Rahmani Siamak | Messi Lionel | Ribéry Franck | Cristiano Ronaldo |
| Media | Iraq | Abdul Emam Sami | Ribéry Franck | Cristiano Ronaldo | Ibrahimovic Zlatan |
| Media | Ireland | Kelly Paul | Ribéry Franck | Cristiano Ronaldo | Messi Lionel |
| Media | Israel | Klieger Noah | Ribéry Franck | Cristiano Ronaldo | Ibrahimovic Zlatan |
| Media | Italy | Condo Paolo | Ribéry Franck | Cristiano Ronaldo | Lewandowski Robert |
| Media | Japan | Tamura Shuichi | Ribéry Franck | Cristiano Ronaldo | Van Persie Robin |
| Media | Kazakhstan | Tulegenov Geniy | Robben Arjen | Müller Thomas | Ribéry Franck |
| Media | Kenya | Nyende Charles | Ribéry Franck | Messi Lionel | Lewandowski Robert |
| Media | Kuwait | Attia Abd Al Aziz Al | Cristiano Ronaldo | Ribéry Franck | Messi Lionel |
| Media | Kyrgyzstan | Louzanov Pavel | Cristiano Ronaldo | Neymar | Ribéry Franck |
| Media | Lebanon | Fawaz Mohamed | Ribéry Franck | Messi Lionel | Cristiano Ronaldo |
| Media | Lesotho | Matjama Thabang | Ribéry Franck | Messi Lionel | Cristiano Ronaldo |
| Media | Liberia | Carter James Burgess | Cristiano Ronaldo | Messi Lionel | Van Persie Robin |
| Media | Libya | Cutajar Joseph | Cristiano Ronaldo | Messi Lionel | Schweinsteiger Bastian |
| Media | Liechtenstein | Hasler Ernst | Ribéry Franck | Lewandowski Robert | Messi Lionel |
| Media | Lithuania | Janonis Giedrius | Ribéry Franck | Messi Lionel | Cristiano Ronaldo |

| Vote | Country | Name | First (5 points) | Second (3 points) | Third (1 point) |
| --- | --- | --- | --- | --- | --- |
| Media | Luxembourg | Hiegel Didier | Ribéry Franck | Messi Lionel | Cristiano Ronaldo |
| Media | Madagascar | Rabary Clément | Ribéry Franck | Messi Lionel | Cristiano Ronaldo |
| Media | Malawi | Kanjere Peter | Messi Lionel | Cristiano Ronaldo | Ribéry Franck |
| Media | Malaysia | Hashim Rizal | Cristiano Ronaldo | Ribéry Franck | Messi Lionel |
| Media | Maldives | Ali Shimaz | Cristiano Ronaldo | Messi Lionel | Ribéry Franck |
| Media | Mali | Bobo Tounkara Souleymane | Ribéry Franck | Messi Lionel | Cristiano Ronaldo |
| Media | Malta | Camenzuli Charles | Ribéry Franck | Cristiano Ronaldo | Ibrahimovic Zlatan |
| Media | Mauritania | Ould El Hacen Mohamed | Cristiano Ronaldo | Messi Lionel | Robben Arjen |
| Media | Mauritius | Hydoo Azmaal | Ribéry Franck | Messi Lionel | Cristiano Ronaldo |
| Media | Mexico | Aguilera Salvador | Ribéry Franck | Messi Lionel | Neymar |
| Media | Moldova | Donets Serghei | Ribéry Franck | Messi Lionel | Ibrahimovic Zlatan |
| Media | Montenegro | Mitrovic Danilo | Ribéry Franck | Cristiano Ronaldo | Ibrahimovic Zlatan |
| Media | Morocco | Badri Mostafa | Cristiano Ronaldo | Ribéry Franck | Ibrahimovic Zlatan |
| Media | Mozambique | Zandamela Alexandre | Cristiano Ronaldo | Ribéry Franck | Ibrahimovic Zlatan |
| Media | Namibia | Nikodemus Sheefeni | Cristiano Ronaldo | Messi Lionel | Ribéry Franck |
| Media | Nepal | Gautam Biplav | Ribéry Franck | Messi Lionel | Ibrahimovic Zlatan |
| Media | Netherlands | Nieuwenhof Frans Van Den | Cristiano Ronaldo | Ibrahimovic Zlatan | Messi Lionel |
| Media | New Caledonia | Colombel Alban | Messi Lionel | Ibrahimovic Zlatan | Ribéry Franck |
| Media | New Zealand | Watson Gordon Glen | Cristiano Ronaldo | Ibrahimovic Zlatan | Messi Lionel |
| Media | Nicaragua | Rosales Cruz Osman | Cristiano Ronaldo | Messi Lionel | Ribéry Franck |
| Media | Niger | Ganoua Mohamed Silimane | Ribéry Franck | Messi Lionel | Cristiano Ronaldo |
| Media | Nigeria | Audu Samm | Messi Lionel | Cristiano Ronaldo | Falcao Radamel |
| Media | North Korea | Dong Gyu Ri | Ribéry Franck | Messi Lionel | Robben Arjen |
| Media | Northern Ireland | Fullerton Jackie | Cristiano Ronaldo | Messi Lionel | Ribéry Franck |
| Media | Norway | Tjaernaas Lars | Cristiano Ronaldo | Messi Lionel | Ibrahimovic Zlatan |
| Media | Oman | Al-Barhi Saleh | Messi Lionel | Ribéry Franck | Ibrahimovic Zlatan |
| Media | Pakistan | Zafar Emad | Ribéry Franck | Messi Lionel | Cristiano Ronaldo |
| Media | Palestine | Iraqi Mohamad | Cristiano Ronaldo | Ribéry Franck | Messi Lionel |
| Media | Panama | Estrada Campo Elias | Cristiano Ronaldo | Ribéry Franck | Messi Lionel |
| Media | Paraguay | Da Rosa Ruben Dario | Cristiano Ronaldo | Messi Lionel | Ribéry Franck |
| Media | Peru | Salinas Carlos | Ribéry Franck | Cristiano Ronaldo | Messi Lionel |
| Media | Philippines | Fenix Ryan | Ribéry Franck | Robben Arjen | Cristiano Ronaldo |
| Media | Poland | Iwanski Maciej | Ribéry Franck | Cristiano Ronaldo | Lewandowski Robert |

| Vote | Country | Name | First (5 points) | Second (3 points) | Third (1 point) |
| --- | --- | --- | --- | --- | --- |
| Media | Portugal | Rita Joaquim | Cristiano Ronaldo | Messi Lionel | Ribéry Franck |
| Media | Puerto Rico | Arce Luis Santigo | Cristiano Ronaldo | Ribéry Franck | Messi Lionel |
| Media | Qatar | Alkhulaifi Majed | Cristiano Ronaldo | Messi Lionel | Ribéry Franck |
| Media | Romania | Rosu Emmanuel | Ibrahimovic Zlatan | Ribéry Franck | Robben Arjen |
| Media | Russia | Kleshchev Konstantin | Ribéry Franck | Cristiano Ronaldo | Robben Arjen |
| Media | Rwanda | Mugabe Bonnie | Ribéry Franck | Cristiano Ronaldo | Messi Lionel |
| Media | San Marino | Gorini Elia | Ribéry Franck | Robben Arjen | Cristiano Ronaldo |
| Media | Saudi Arabia | Al.Solame Rjallah | Ribéry Franck | Messi Lionel | Cristiano Ronaldo |
| Media | Scotland | Greechan John | Ribéry Franck | Messi Lionel | Cristiano Ronaldo |
| Media | Senegal | Goloko Aliou | Ribéry Franck | Ibrahimovic Zlatan | Touré Yaya |
| Media | Serbia | Novak Vladimir | Ibrahimovic Zlatan | Ribéry Franck | Messi Lionel |
| Media | Seychelles | Govinden Gérard | Messi Lionel | Cristiano Ronaldo | Neymar |
| Media | Sierra Leone | Fajah Barrie Mohamed | Ribéry Franck | Messi Lionel | Cristiano Ronaldo |
| Media | Singapore | Lim Gary | Ribéry Franck | Messi Lionel | Neymar |
| Media | Slovakia | Surin Peter | Ribéry Franck | Cristiano Ronaldo | Messi Lionel |
| Media | Slovenia | Stare Andrej | Cristiano Ronaldo | Ribéry Franck | Cavani Edinson |
| Media | South Africa | Gleeson Mark | Cristiano Ronaldo | Ribéry Franck | Messi Lionel |
| Media | South Korea | Kim Hanseok | Messi Lionel | Van Persie Robin | Neymar |
| Media | Spain | Aguilar Paco | Ribéry Franck | Messi Lionel | Cristiano Ronaldo |
| Media | Sri Lanka | Goonetilleke Asoka | Messi Lionel | Bale Gareth | Neymar |
| Media | St. Lucia | James Lawrence | Messi Lionel | Cristiano Ronaldo | Bale Gareth |
| Media | Sudan | Abu Elgassim Muzamil | Messi Lionel | Robben Arjen | Cristiano Ronaldo |
| Media | Suriname | Romeo Desney | Ribéry Franck | Cristiano Ronaldo | Messi Lionel |
| Media | Swaziland | Dlamini Kenneth | Neymar | Ribéry Franck | Messi Lionel |
| Media | Sweden | Ysten Henrik | Cristiano Ronaldo | Messi Lionel | Ibrahimovic Zlatan |
| Media | Switzerland | Dupuis Pierre-Alain | Cristiano Ronaldo | Ibrahimovic Zlatan | Ribéry Franck |
| Media | Tahiti | Huc Olivier | Ibrahimovic Zlatan | Cristiano Ronaldo | Messi Lionel |
| Media | Tajikistan | Buriev Alaveddine | Ribéry Franck | Messi Lionel | Cristiano Ronaldo |
| Media | Tanzania | Wambura Boniface | Cristiano Ronaldo | Messi Lionel | Van Persie Robin |
| Media | Thailand | Patoommawatana Urai | Ribéry Franck | Messi Lionel | Cristiano Ronaldo |
| Media | Togo | Ayena Mathias | Cristiano Ronaldo | Ribéry Franck | Touré Yaya |
| Media | Trinidad and Tobago | Liburd Lasana | Messi Lionel | Lahm Philipp | Neymar |
| Media | Tunisia | Dhaifallah Abdesslam | Ribéry Franck | Messi Lionel | Ibrahimovic Zlatan |

| Vote | Country | Name | First (5 points) | Second (3 points) | Third (1 point) |
| --- | --- | --- | --- | --- | --- |
| Media | Turkey | Manav Selçuk | Ribéry Franck | Robben Arjen | Falcao Radamel |
| Media | Turkmenistan | Vershinin Alexander | Messi Lionel | Robben Arjen | Iniesta Andrés |
| Media | Uganda | Musisi Kiyingi Fredrick | Ribéry Franck | Messi Lionel | Neymar |
| Media | Ukraine | Linnyk Igor | Ribéry Franck | Neymar | Messi Lionel |
| Media | United Arab Emirates | Mouadhen Dafrallah | Ribéry Franck | Robben Arjen | Ibrahimovic Zlatan |
| Media | Uruguay | Pineyrua Ricardo | Messi Lionel | Suárez Luis | Cristiano Ronaldo |
| Media | US Virgin Islands | Gray Aaron | Cristiano Ronaldo | Messi Lionel | Ibrahimovic Zlatan |
| Media | USA | Kennedy Paul | Cristiano Ronaldo | Messi Lionel | Ribéry Franck |
| Media | Uzbekistan | Rtveladze Grigoriy | Robben Arjen | Cristiano Ronaldo | Ibrahimovic Zlatan |
| Media | Venezuela | Blavia Francisco | Cristiano Ronaldo | Messi Lionel | Ribéry Franck |
| Media | Vietnam | Anh Ngoc Truong | Ribéry Franck | Messi Lionel | Cristiano Ronaldo |
| Media | Wales | Abbandonato Paul | Cristiano Ronaldo | Messi Lionel | Bale Gareth |
| Media | Yemen | Al Hababi Adel | Ribéry Franck | Messi Lionel | Cristiano Ronaldo |
| Media | Zambia | Lungu Chapadongo | Cristiano Ronaldo | Messi Lionel | Ribéry Franck |
| Media | Zimbabwe | Mabika Charles | Cristiano Ronaldo | Ribéry Franck | Lewandowski Robert |

| Vote | Country | Name | First (5 points) | Second (3 points) | Third (1 point) |
| --- | --- | --- | --- | --- | --- |
| Captain | Afghanistan | Amiri Islam | Messi Lionel | Cristiano Ronaldo | Ibrahimovic Zlatan |
| Captain | Albania | Cana Lorik | Cristiano Ronaldo | Robben Arjen | Mueller Thomas |
| Captain | Algeria | Bougherra Madjid | Cristiano Ronaldo | Robben Arjen | Benzema Karim |
| Captain | American Samoa | Luvu Rafe Talalelei | Neymar | Robben Arjen | Cristiano Ronaldo |
| Captain | Andorra | Sonejee Masand Oscar | Cristiano Ronaldo | Mueller Thomas | Kroos Toni |
| Captain | Angola | Amaral Felisberto | Lahm Philipp | Cristiano Ronaldo | Neuer Manuel |
| Captain | Anguilla | Liddie Ryan | Messi Lionel | Cristiano Ronaldo | Iniesta Andres |
| Captain | Antigua and Barbuda | Griffith Quinton | Courtois Thibaut | Messi Lionel | Di Maria Angel |
| Captain | Argentina | Messi Lionel | Di Maria Angel | Iniesta Andres | Mascherano Javier |
| Captain | Armenia | Berezovskiy Roman | Lahm Philipp | Messi Lionel | Neuer Manuel |
| Captain | Aruba | Breinburg Reinhard | Cristiano Ronaldo | Kroos Toni | Neymar |
| Captain | Australia | Jedinak Mile | Cristiano Ronaldo | Kroos Toni | Toure Yaya |
| Captain | Austria | Fuchs Christian | Cristiano Ronaldo | Robben Arjen | Neuer Manuel |
| Captain | Azerbaijan | Sadigov Rashad | Cristiano Ronaldo | Neuer Manuel | Robben Arjen |
| Captain | Bahamas | Gavin Christie | Cristiano Ronaldo | Neymar | Robben Arjen |
| Captain | Bahrain | Hasan Mohamed | Cristiano Ronaldo | Neymar | Ramos Sergio |
| Captain | Bangladesh | Mamun Md Mamnul Islam | Messi Lionel | Cristiano Ronaldo | Robben Arjen |
| Captain | Barbados | Burgess Romelle | Messi Lionel | Cristiano Ronaldo | Neymar |
| Captain | Belarus | Kalachou Tsimafei | Neuer Manuel | Cristiano Ronaldo | Robben Arjen |
| Captain | Belgium | Komany Vincent | Courtois Thibaut | Hazard Eden | Robben Arjen |
| Captain | Belize | Eiley Dalton | Messi Lionel | Cristiano Ronaldo | Neymar |
| Captain | Bermuda | Nusum John | Cristiano Ronaldo | Messi Lionel | Neuer Manuel |
| Captain | Bhutan | Tshering Passang | Cristiano Ronaldo | Hazard Eden | Costa Diego |
| Captain | Bolivia | Raldes Ronald | Di Maria Angel | Cristiano Ronaldo | Messi Lionel |
| Captain | Bosnia and Herzegovina | Džeko Edin | Cristiano Ronaldo | Messi Lionel | Robben Arjen |
| Captain | Brazil | Da Silva Santos Junior Neymar | Messi Lionel | Cristiano Ronaldo | Mascherano Javier |
| Captain | British Virgin Islands | Ceasar Troy | Cristiano Ronaldo | Di Maria Angel | Toure Yaya |
| Captain | Brunei Darussalam | Khairul Shame Bin Suhaimi Mohamad | Lahm Philipp | Robben Arjen | Cristiano Ronaldo |
| Captain | Bulgaria | Popov Ivelin | Cristiano Ronaldo | Ibrahimovic Zlatan | Mueller Thomas |
| Captain | Burkina Faso | Kabore Charles | Cristiano Ronaldo | Messi Lionel | Benzema Karim |
| Captain | Burundi | Ntibazonkiza Saidi | Cristiano Ronaldo | Messi Lionel | Toure Yaya |
| Captain | Cambodia | Kouch Sokumpheak | Messi Lionel | Benzema Karim | Iniesta Andres |

| Vote | Country | Name | First (5 points) | Second (3 points) | Third (1 point) |
| --- | --- | --- | --- | --- | --- |
| Captain | Cameroon | Mbia Etoundi Stephane | Cristiano Ronaldo | Robben Arjen | Neuer Manuel |
| Captain | Canada | De Guzman Julian | Mueller Thomas | Neuer Manuel | Robben Arjen |
| Captain | Cape Verde Islands | Soares Marco | Cristiano Ronaldo | Robben Arjen | Di Maria Angel |
| Captain | Cayman Islands | Suberan Matthew | Cristiano Ronaldo | Messi Lionel | Hazard Eden |
| Captain | Chad | Ngar Ezechiel | Messi Lionel | Cristiano Ronaldo | Neymar |
| Captain | Chile | Bravo Claudio | Messi Lionel | Iniesta Andres | Neuer Manuel |
| Captain | China PR | Zhang Linpeng | Cristiano Ronaldo | Messi Lionel | Mueller Thomas |
| Captain | Chinese Taipei | Lee Meng Chian | Neuer Manuel | Robben Arjen | Neymar |
| Captain | Colombia | Garcia Zarate Radamel Falcao | Rodriguez James | Cristiano Ronaldo | Di Maria Angel |
| Captain | Comoros | Abdou Nadjim | Messi Lionel | Robben Arjen | Lahm Philipp |
| Captain | Congo | Oniangue Prince Alban | Messi Lionel | Toure Yaya | Pogba Paul |
| Captain | Congo DR | Mulumbu Youssouf | Cristiano Ronaldo | Toure Yaya | Messi Lionel |
| Captain | Cook Islands | Joseph Mii | Messi Lionel | Cristiano Ronaldo | Di Maria Angel |
| Captain | Costa Rica | Ruiz Bryan | Cristiano Ronaldo | Iniesta Andres | Robben Arjen |
| Captain | Croatia | Srna Darijo | Cristiano Ronaldo | Schweinsteiger Bastian | Robben Arjen |
| Captain | Cuba | Marquez Yeniel | Cristiano Ronaldo | Neymar | Rodriguez James |
| Captain | Curaçao | Hoyer Ludgino | Messi Lionel | Rodriguez James | Kroos Toni |
| Captain | Cyprus | Charalambides Constantinos | Cristiano Ronaldo | Robben Arjen | Messi Lionel |
| Captain | Czech Republic | Rosicky Tomas | Cristiano Ronaldo | Messi Lionel | Schweinsteiger Bastian |
| Captain | Denmark | Agger Daniel | Cristiano Ronaldo | Messi Lionel | Neuer Manuel |
| Captain | Djibouti | Mohamed Kadar | Messi Lionel | Robben Arjen | Neuer Manuel |
| Captain | Dominica | Prince Glenson | Cristiano Ronaldo | Messi Lionel | Kroos Toni |
| Captain | Dominican Republic | Martinez Hasley | Cristiano Ronaldo | Messi Lionel | Ramos Sergio |
| Captain | Ecuador | Ayovi Corozo Walter Orlando | Robben Arjen | Cristiano Ronaldo | Kroos Toni |
| Captain | Egypt | Fathi Ahmed | Cristiano Ronaldo | Messi Lionel | Neuer Manuel |
| Captain | El Salvador | Flores Andres | Cristiano Ronaldo | Messi Lionel | Mueller Thomas |
| Captain | England | Rooney Wayne | Cristiano Ronaldo | Kroos Toni | Bale Gareth |
| Captain | Estonia | Klavan Ragnar | Lahm Philipp | Cristiano Ronaldo | Neuer Manuel |
| Captain | Ethiopia | Bune Abebaw Butako | Cristiano Ronaldo | Messi Lionel | Neuer Manuel |
| Captain | Faroe Islands | Benjaminsen Fróði | Cristiano Ronaldo | Messi Lionel | Ibrahimovic Zlatan |
| Captain | Fiji | Tamanisau Simione | Messi Lionel | Cristiano Ronaldo | Neuer Manuel |
| Captain | Finland | Moisander Niklas | Cristiano Ronaldo | Messi Lionel | Ibrahimovic Zlatan |
| Captain | France | Lloris Hugo | Cristiano Ronaldo | Benzema Karim | Neuer Manuel |

| Vote | Country | Name | First (5 points) | Second (3 points) | Third (1 point) |
| --- | --- | --- | --- | --- | --- |
| Captain | FYR Macedonia | Pachovski Tome | Cristiano Ronaldo | Neuer Manuel | Hazard Eden |
| Captain | Gabon | Pierre Aubameyang | Messi Lionel | Cristiano Ronaldo | Neymar |
| Captain | Georgia | Kankava Jaba | Iniesta Andres | Cristiano Ronaldo | Messi Lionel |
| Captain | Germany | Schweinsteiger Bastian | Neuer Manuel | Lahm Philipp | Mueller Thomas |
| Captain | Ghana | Asamoah Gyan | Cristiano Ronaldo | Messi Lionel | Kroos Toni |
| Captain | Greece | Torosidis Vasileios | Robben Arjen | Cristiano Ronaldo | Lahm Philipp |
| Captain | Grenada | Rennie Jake | Cristiano Ronaldo | Messi Lionel | Hazard Eden |
| Captain | Guam | Cunliffe Jason | Mueller Thomas | Robben Arjen | Neuer Manuel |
| Captain | Guatemala | Ruiz Carlos | Cristiano Ronaldo | Messi Lionel | Neymar |
| Captain | Guyana | Moore Walter | Messi Lionel | Robben Arjen | Rodriguez James |
| Captain | Honduras | Valladares Bonilla Noel Eduardo | Cristiano Ronaldo | Benzema Karim | Messi Lionel |
| Captain | Hong Kong | Chan Wai Ho | Messi Lionel | Cristiano Ronaldo | Iniesta Andres |
| Captain | Hungary | Dzsudzsák Balázs | Neuer Manuel | Cristiano Ronaldo | Messi Lionel |
| Captain | Iceland | Gunnarsson Aron | Cristiano Ronaldo | Robben Arjen | Messi Lionel |
| Captain | India | Chhetri Sunil | Neuer Manuel | Mueller Thomas | Cristiano Ronaldo |
| Captain | Indonesia | Utina Firman | Cristiano Ronaldo | Neymar | Messi Lionel |
| Captain | Iran | Nekounam Javad | Cristiano Ronaldo | Messi Lionel | Neuer Manuel |
| Captain | Israel | Ben Haim Tal | Cristiano Ronaldo | Messi Lionel | Costa Diego |
| Captain | Italy | Buffon Gianluigi | Cristiano Ronaldo | Messi Lionel | Neuer Manuel |
| Captain | Jamaica | Austin Rodolph | Cristiano Ronaldo | Messi Lionel | Robben Arjen |
| Captain | Japan | Honda Keisuke | Neuer Manuel | Bale Gareth | Cristiano Ronaldo |
| Captain | Jordan | Abdelfattah Hassan | Messi Lionel | Cristiano Ronaldo | Neymar |
| Captain | Kazakhstan | Nuserbayev Tanat | Cristiano Ronaldo | Ibrahimovic Zlatan | Kroos Toni |
| Captain | Kenya | Wanyama Victor | Cristiano Ronaldo | Toure Yaya | Messi Lionel |
| Captain | Korea Republic | Ki Sungyueng | Messi Lionel | Cristiano Ronaldo | Hazard Eden |
| Captain | Kuwait | Al Khaledi Nawaf | Cristiano Ronaldo | Mueller Thomas | Neuer Manuel |
| Captain | Kyrgyzstan | Baimatov Azamat | Cristiano Ronaldo | Lahm Philipp | Messi Lionel |
| Captain | Laos | Souksavanh Ketsada | Neuer Manuel | Goetze Mario | Robben Arjen |
| Captain | Latvia | Gorkšs Kaspars | Cristiano Ronaldo | Messi Lionel | Mueller Thomas |
| Captain | Lebanon | Mehanna Lary | Cristiano Ronaldo | Messi Lionel | Neuer Manuel |
| Captain | Lesotho | Ntobo Moitheri | Cristiano Ronaldo | Messi Lionel | Toure Yaya |
| Captain | Liberia | Lafford Anthony | Cristiano Ronaldo | Messi Lionel | Costa Diego |
| Captain | Liechtenstein | Frick Mario | Neuer Manuel | Cristiano Ronaldo | Robben Arjen |

| Vote | Country | Name | First (5 points) | Second (3 points) | Third (1 point) |
| --- | --- | --- | --- | --- | --- |
| Captain | Lithuania | Kijanskas Tadas | Cristiano Ronaldo | Mueller Thomas | Messi Lionel |
| Captain | Luxembourg | Mutsch Mario | Cristiano Ronaldo | Mueller Thomas | Neuer Manuel |
| Captain | Macau | Cheang Cheng Ieong Paulo | Cristiano Ronaldo | Robben Arjen | Messi Lionel |
| Captain | Madagascar | Rabeson F. Michael | Lahm Philipp | Mascherano Javier | Cristiano Ronaldo |
| Captain | Malawi | Kamwendo Joseph | Cristiano Ronaldo | Ibrahimovic Zlatan | Messi Lionel |
| Captain | Maldives | Ashfaq Ali | Cristiano Ronaldo | Messi Lionel | Neymar |
| Captain | Mali | Keita Seydou | Cristiano Ronaldo | Neuer Manuel | Toure Yaya |
| Captain | Malta | Mifsud Michael | Bale Gareth | Robben Arjen | Messi Lionel |
| Captain | Mauritania | Ndiaye Oumar | Messi Lionel | Cristiano Ronaldo | Neuer Manuel |
| Captain | Mauritius | Bell Collin | Cristiano Ronaldo | Messi Lionel | Neuer Manuel |
| Captain | Mexico | Guardado Hernandez José Andrés | Lahm Philipp | Di Maria Angel | Cristiano Ronaldo |
| Captain | Moldova | Epureanu Alexandru | Neuer Manuel | Mueller Thomas | Robben Arjen |
| Captain | Mongolia | Baysgalan Garidmagnai | Cristiano Ronaldo | Messi Lionel | Neuer Manuel |
| Captain | Montenegro | Vucinic Mirko | Ibrahimovic Zlatan | Cristiano Ronaldo | Mueller Thomas |
| Captain | Montserrat | Griffith Anthony | Costa Diego | Hazard Eden | Neymar |
| Captain | Morocco | Elmoutaqui Mehdi | Neuer Manuel | Cristiano Ronaldo | Lahm Philipp |
| Captain | Mozambique | Khan Dario Ivan | Kroos Toni | Cristiano Ronaldo | Mueller Thomas |
| Captain | Myanmar | Lwin Khin Maung | Cristiano Ronaldo | Neuer Manuel | Robben Arjen |
| Captain | Namibia | Ketjijere Ronald | Cristiano Ronaldo | Mueller Thomas | Messi Lionel |
| Captain | Nepal | Thapa Sagar | Cristiano Ronaldo | Messi Lionel | Neuer Manuel |
| Captain | Netherlands | Van Persie Robin | Robben Arjen | Ibrahimovic Zlatan | Neuer Manuel |
| Captain | New Caledonia | Dokunengo Olivier | Cristiano Ronaldo | Mueller Thomas | Messi Lionel |
| Captain | New Zealand | Wood Chris | Cristiano Ronaldo | Rodriguez James | Costa Diego |
| Captain | Nicaragua | Solórzano Sánchez David | Cristiano Ronaldo | Kroos Toni | Mueller Thomas |
| Captain | Niger | Ouwo Moussa Maazou | Cristiano Ronaldo | Toure Yaya | Benzema Karim |
| Captain | Nigeria | Enyeama Vincent | Cristiano Ronaldo | Ibrahimovic Zlatan | Toure Yaya |
| Captain | Norway | Skjelbred Per Ciljan | Cristiano Ronaldo | Ibrahimovic Zlatan | Messi Lionel |
| Captain | Oman | Al Habsi Ali | Neuer Manuel | Mueller Thomas | Cristiano Ronaldo |
| Captain | Pakistan | Ullah Kaleem | Cristiano Ronaldo | Robben Arjen | Neymar |
| Captain | Palestine | Saleh Ramzi | Messi Lionel | Neuer Manuel | Neymar |
| Captain | Panama | Torres Román | Messi Lionel | Cristiano Ronaldo | Rodriguez James |
| Captain | Paraguay | Santa Cruz Roque | Cristiano Ronaldo | Robben Arjen | Lahm Philipp |
| Captain | Peru | Pizarro Claudio | Neuer Manuel | Cristiano Ronaldo | Di Maria Angel |

| Vote | Country | Name | First (5 points) | Second (3 points) | Third (1 point) |
| --- | --- | --- | --- | --- | --- |
| Captain | Philippines | Gier Robert James | Cristiano Ronaldo | Messi Lionel | Neuer Manuel |
| Captain | Poland | Lewandowski Robert | Cristiano Ronaldo | Neuer Manuel | Schweinsteiger Bastian |
| Captain | Portugal | Ronaldo Cristiano | Ramos Sergio | Bale Gareth | Benzema Karim |
| Captain | Puerto Rico | Ramos Héctor | Neuer Manuel | Neymar | Mueller Thomas |
| Captain | Qatar | Rajab Bilal | Cristiano Ronaldo | Robben Arjen | Lahm Philipp |
| Captain | Republic of Ireland | Keane Robbie | Cristiano Ronaldo | Messi Lionel | Bale Gareth |
| Captain | Romania | Ciprian Marica | Neuer Manuel | Costa Diego | Messi Lionel |
| Captain | Russia | Berezutski Vasili | Neuer Manuel | Cristiano Ronaldo | Robben Arjen |
| Captain | Rwanda | Niyonzima Haruna | Cristiano Ronaldo | Toure Yaya | Messi Lionel |
| Captain | Samoa | Faaiauso Desmond | Messi Lionel | Cristiano Ronaldo | Neymar |
| Captain | San Marino | Selva Andy | Cristiano Ronaldo | Messi Lionel | Robben Arjen |
| Captain | Saudi Arabia | Kariri Saud | Cristiano Ronaldo | Lahm Philipp | Neuer Manuel |
| Captain | Scotland | Brown Scott | Cristiano Ronaldo | Robben Arjen | Neuer Manuel |
| Captain | Senegal | Diame Mohamed | Cristiano Ronaldo | Neuer Manuel | Messi Lionel |
| Captain | Serbia | Ivanovic Branislav | Cristiano Ronaldo | Messi Lionel | Hazard Eden |
| Captain | Seychelles | Hoareau Ronny | Cristiano Ronaldo | Robben Arjen | Messi Lionel |
| Captain | Sierra Leone | Bangura Umaru | Courtois Thibaut | Messi Lionel | Neymar |
| Captain | Singapore | Ishak Shahril | Cristiano Ronaldo | Messi Lionel | Mueller Thomas |
| Captain | Slovakia | Skrtel Martin | Cristiano Ronaldo | Benzema Karim | Hazard Eden |
| Captain | Slovenia | Cesar Boštjan | Cristiano Ronaldo | Costa Diego | Robben Arjen |
| Captain | Solomon Islands | Faro'Odo Junior Henry | Neuer Manuel | Lahm Philipp | Goetze Mario |
| Captain | South Africa | Furman Dean | Cristiano Ronaldo | Messi Lionel | Lahm Philipp |
| Captain | South Sudan | Juma Jenaro | Cristiano Ronaldo | Neuer Manuel | Lahm Philipp |
| Captain | Spain | Casillas Iker | Cristiano Ronaldo | Ramos Sergio | Mueller Thomas |
| Captain | Sri Lanka | Chathuranga Sanjeewa | Messi Lionel | Robben Arjen | Cristiano Ronaldo |
| Captain | St. Kitts and Nevis | Harris Atiba | Messi Lionel | Cristiano Ronaldo | Robben Arjen |
| Captain | St. Lucia | Emmanuel Sheldon | Goetze Mario | Messi Lionel | Cristiano Ronaldo |
| Captain | St. Vincent and the  Grenadines | Hamlet Darren | Messi Lionel | Cristiano Ronaldo | Robben Arjen |
| Captain | Sudan | Abdalla Almoez Mahgoub | Lahm Philipp | Messi Lionel | Mueller Thomas |
| Captain | Suriname | Darson Zhirvano | Neuer Manuel | Mueller Thomas | Messi Lionel |
| Captain | Swaziland | Tsabedze Tony | Cristiano Ronaldo | Neuer Manuel | Messi Lionel |
| Captain | Sweden | Ibrahimovic Zlatan | Messi Lionel | Neuer Manuel | Cristiano Ronaldo |

| Vote | Country | Name | First (5 points) | Second (3 points) | Third (1 point) |
| --- | --- | --- | --- | --- | --- |
| Captain | Switzerland | Inler Gökhan | Cristiano Ronaldo | Robben Arjen | Neymar |
| Captain | Syria | Balhous Mosab | Messi Lionel | Neymar | Ramos Sergio |
| Captain | Tahiti | Vallard Nicolas | Neuer Manuel | Benzema Karim | Iniesta Andres |
| Captain | Tajikistan | Tuychiev Alisher | Messi Lionel | Cristiano Ronaldo | Robben Arjen |
| Captain | Tanzania | Haroub Nadir | Messi Lionel | Cristiano Ronaldo | Mueller Thomas |
| Captain | Thailand | Lahsoh Adul | Cristiano Ronaldo | Robben Arjen | Mascherano Javier |
| Captain | Turkey | Turan Arda | Cristiano Ronaldo | Robben Arjen | Benzema Karim |
| Captain | Turks and Caicos Islands | Clinton Gavin | Cristiano Ronaldo | Bale Gareth | Hazard Eden |
| Captain | Ukraine | Tymoschuk Anatoliy | Neuer Manuel | Mueller Thomas | Cristiano Ronaldo |
| Captain | United Arab Emirates | Aljneibi Ismaeil | Neuer Manuel | Kroos Toni | Cristiano Ronaldo |
| Captain | Uruguay | Godín Diego | Costa Diego | Courtois Thibaut | Robben Arjen |
| Captain | US Virgin Islands | Van Gurp Alberto | Neuer Manuel | Cristiano Ronaldo | Mueller Thomas |
| Captain | USA | Dempsey Clint | Cristiano Ronaldo | Messi Lionel | Mueller Thomas |
| Captain | Uzbekistan | Djeparov Server | Mueller Thomas | Messi Lionel | Cristiano Ronaldo |
| Captain | Venezuela | Rincon Tomas | Cristiano Ronaldo | Neuer Manuel | Robben Arjen |
| Captain | Vietnam | Le Tan Tai | Neuer Manuel | Cristiano Ronaldo | Messi Lionel |
| Captain | Wales | Williams Ashley | Bale Gareth | Schweinsteiger Bastian | Hazard Eden |
| Captain | Zambia | Stopilla Sunzu | Di Maria Angel | Neuer Manuel | Messi Lionel |
| Captain | Zimbabwe | Wisdom Mutasa | Cristiano Ronaldo | Messi Lionel | Hazard Eden |
| Coach | Afghanistan | Kargar Mohammad Yosuf | Ibrahimovic Zlatan | Cristiano Ronaldo | Messi Lionel |
| Coach | Albania | De Biasi Giovanni | Cristiano Ronaldo | Lahm Philipp | Robben Arjen |
| Coach | Algeria | Gourcuff Christian | Lahm Philipp | Messi Lionel | Robben Arjen |
| Coach | American Samoa | Aliva Uinifareti | Neymar | Messi Lionel | Cristiano Ronaldo |
| Coach | Andorra | Alvarez De Eulate Jesus Luis | Mueller Thomas | Cristiano Ronaldo | Messi Lionel |
| Coach | Angola | Filemon Romeu | Cristiano Ronaldo | Robben Arjen | Messi Lionel |
| Coach | Anguilla | Johnson Colin | Cristiano Ronaldo | Messi Lionel | Mueller Thomas |
| Coach | Antigua and Barbuda | Williams Rolston | Neuer Manuel | Cristiano Ronaldo | Messi Lionel |
| Coach | Argentina | Martino Gerardo | Messi Lionel | Di Maria Angel | Mascherano Javier |
| Coach | Armenia | Challandes Bernard | Cristiano Ronaldo | Neuer Manuel | Mueller Thomas |
| Coach | Aruba | Franken Giovanni | Robben Arjen | Costa Diego | Cristiano Ronaldo |
| Coach | Australia | Postecoglou Ange | Cristiano Ronaldo | Messi Lionel | Neuer Manuel |
| Coach | Austria | Koller Marcel | Cristiano Ronaldo | Di Maria Angel | Neuer Manuel |
| Coach | Azerbaijan | Gurbanov Mahmud | Robben Arjen | Di Maria Angel | Cristiano Ronaldo |

| Vote | Country | Name | First (5 points) | Second (3 points) | Third (1 point) |
| --- | --- | --- | --- | --- | --- |
| Coach | Bahamas | Jean Nesley | Neymar | Cristiano Ronaldo | Kroos Toni |
| Coach | Bahrain | Alabassi Adnan | Neuer Manuel | Messi Lionel | Schweinsteiger Bastian |
| Coach | Bangladesh | A.K.M. Saiful Bari Ttiu | Cristiano Ronaldo | Messi Lionel | Neymar |
| Coach | Barbados | Falopa Marcos | Cristiano Ronaldo | Neymar | Messi Lionel |
| Coach | Belarus | Zygmantovitch Andrei | Mascherano Javier | Neuer Manuel | Mueller Thomas |
| Coach | Belgium | Wilmots Marc | Robben Arjen | Neuer Manuel | Courtois Thibaut |
| Coach | Belize | Nunez Jorge | Cristiano Ronaldo | Ibrahimovic Zlatan | Messi Lionel |
| Coach | Bermuda | Bascome Andrew | Cristiano Ronaldo | Messi Lionel | Neuer Manuel |
| Coach | Bhutan | Chokey Nima | Messi Lionel | Robben Arjen | Mueller Thomas |
| Coach | Bolivia | Clausen Nestor | Messi Lionel | Cristiano Ronaldo | Di Maria Angel |
| Coach | Bosnia and Herzegovina | Sušic Safet | Cristiano Ronaldo | Messi Lionel | Robben Arjen |
| Coach | Brazil | Bledorn Verri Carlos Caetano | Neymar | Cristiano Ronaldo | Ibrahimovic Zlatan |
| Coach | British Virgin Islands | Williams Avondale | Neuer Manuel | Messi Lionel | Cristiano Ronaldo |
| Coach | Brunei Darussalam | Bin Haji Ali Dayem | Neymar | Rodriguez James | Mueller Thomas |
| Coach | Bulgaria | Penev Lyuboslav | Cristiano Ronaldo | Neuer Manuel | Kroos Toni |
| Coach | Burkina Faso | Put Paul Joseph François | Cristiano Ronaldo | Toure Yaya | Messi Lionel |
| Coach | Burundi | Willfeld Rainer | Neuer Manuel | Robben Arjen | Lahm Philipp |
| Coach | Cambodia | Lee Tae Hoon | Cristiano Ronaldo | Messi Lionel | Lahm Philipp |
| Coach | Cameroon | Volker Finke | Robben Arjen | Rodriguez James | Toure Yaya |
| Coach | Canada | Floro Sanz Benito | Cristiano Ronaldo | Ramos Sergio | Robben Arjen |
| Coach | Cape Verde Islands | Águas Rui | Cristiano Ronaldo | Robben Arjen | Di Maria Angel |
| Coach | Chad | Emmanuel Tregoat | Cristiano Ronaldo | Robben Arjen | Neuer Manuel |
| Coach | Chile | Sampaoli Jorge | Cristiano Ronaldo | Robben Arjen | Messi Lionel |
| Coach | China PR | Perrin Alain Andre Christian | Mueller Thomas | Messi Lionel | Robben Arjen |
| Coach | Chinese Taipei | Chen Kuei Jen | Bale Gareth | Costa Diego | Messi Lionel |
| Coach | Colombia | Pekerman Jose Nestor | Rodriguez James | Messi Lionel | Cristiano Ronaldo |
| Coach | Comoros | Abdou Amir | Mueller Thomas | Neuer Manuel | Cristiano Ronaldo |
| Coach | Congo | Leroy Claude Marie François | Neuer Manuel | Robben Arjen | Toure Yaya |
| Coach | Congo DR | Ibenge Florent | Messi Lionel | Cristiano Ronaldo | Toure Yaya |
| Coach | Cook Islands | Tisam Tuka | Messi Lionel | Cristiano Ronaldo | Di Maria Angel |
| Coach | Costa Rica | Paulo Wanchope | Cristiano Ronaldo | Kroos Toni | Neuer Manuel |
| Coach | Côte d'Ivoire | Renard Hervé Jean Marie Roger | Cristiano Ronaldo | Neuer Manuel | Messi Lionel |
| Coach | Croatia | Kovac Niko | Lahm Philipp | Neuer Manuel | Schweinsteiger Bastian |

| Vote | Country | Name | First (5 points) | Second (3 points) | Third (1 point) |
| --- | --- | --- | --- | --- | --- |
| Coach | Cuba | Benites Walter | Cristiano Ronaldo | Neymar | Rodriguez James |
| Coach | Curaçao | Pieternella Ingomar | Neuer Manuel | Messi Lionel | Cristiano Ronaldo |
| Coach | Cyprus | Christodoulou Charalampos | Cristiano Ronaldo | Robben Arjen | Neuer Manuel |
| Coach | Czech Republic | Vrba Pavel | Robben Arjen | Lahm Philipp | Neuer Manuel |
| Coach | Denmark | Olsen Morten | Cristiano Ronaldo | Robben Arjen | Messi Lionel |
| Coach | Djibouti | Mohamed Ahmed Badri | Messi Lionel | Neuer Manuel | Robben Arjen |
| Coach | Dominica | Marshall Shane | Neuer Manuel | Cristiano Ronaldo | Messi Lionel |
| Coach | Dominican Republic | Hernandez Heres Clemente Domingo | Cristiano Ronaldo | Messi Lionel | Robben Arjen |
| Coach | Ecuador | Vizuete Toapanta Sixto Rafael | Kroos Toni | Pogba Paul | Rodriguez James |
| Coach | Egypt | Gharib Shawky | Cristiano Ronaldo | Messi Lionel | Neuer Manuel |
| Coach | El Salvador | Roca Albert | Iniesta Andres | Messi Lionel | Neymar |
| Coach | England | Hodgson Roy | Mascherano Javier | Lahm Philipp | Neuer Manuel |
| Coach | Estonia | Pehrsson Magnus | Neuer Manuel | Cristiano Ronaldo | Robben Arjen |
| Coach | Ethiopia | Barreto Mariano Jeronimo | Cristiano Ronaldo | Messi Lionel | Neuer Manuel |
| Coach | Faroe Islands | Olsen Lars | Cristiano Ronaldo | Robben Arjen | Messi Lionel |
| Coach | Fiji | Buzzetti Juan Carlos | Cristiano Ronaldo | Messi Lionel | Neymar |
| Coach | Finland | Paatelainen Mixu | Neuer Manuel | Cristiano Ronaldo | Messi Lionel |
| Coach | France | Deschamps Didier | Cristiano Ronaldo | Neuer Manuel | Benzema Karim |
| Coach | FYR Macedonia | Gjurovski Boshko | Neuer Manuel | Robben Arjen | Cristiano Ronaldo |
| Coach | Gabon | Jorge Costa | Messi Lionel | Cristiano Ronaldo | Rodriguez James |
| Coach | Georgia | Ketsbaia Temur | Cristiano Ronaldo | Neuer Manuel | Robben Arjen |
| Coach | Germany | Löw Joachim | Neuer Manuel | Lahm Philipp | Schweinsteiger Bastian |
| Coach | Ghana | Maxwell Konadu | Cristiano Ronaldo | Messi Lionel | Mueller Thomas |
| Coach | Greece | Ranieri Claudio | Neuer Manuel | Cristiano Ronaldo | Robben Arjen |
| Coach | Grenada | Modeste Anthony | Cristiano Ronaldo | Toure Yaya | Messi Lionel |
| Coach | Guam | White Gary | Neuer Manuel | Robben Arjen | Lahm Philipp |
| Coach | Guatemala | Sopegno Ivan | Messi Lionel | Cristiano Ronaldo | Rodriguez James |
| Coach | Guyana | Thompson Denzil | Robben Arjen | Neymar | Messi Lionel |
| Coach | Honduras | Medford Bryan Hernán Evaristo | Cristiano Ronaldo | Messi Lionel | Neymar |
| Coach | Hong Kong | Kim Pan Gon | Neuer Manuel | Robben Arjen | Toure Yaya |
| Coach | Hungary | Dárdai Pál | Neuer Manuel | Cristiano Ronaldo | Lahm Philipp |
| Coach | Iceland | Hallgrimsson Heimir | Neuer Manuel | Robben Arjen | Cristiano Ronaldo |

| Vote | Country | Name | First (5 points) | Second (3 points) | Third (1 point) |
| --- | --- | --- | --- | --- | --- |
| Coach | India | Medeira Savio Piedade | Cristiano Ronaldo | Neuer Manuel | Mueller Thomas |
| Coach | Indonesia | Riedl Alfred | Cristiano Ronaldo | Messi Lionel | Neuer Manuel |
| Coach | Iran | Queiroz Carlos | Cristiano Ronaldo | Messi Lionel | Neuer Manuel |
| Coach | Israel | Guttman Eli | Cristiano Ronaldo | Robben Arjen | Neuer Manuel |
| Coach | Italy | Conte Antonio | Cristiano Ronaldo | Messi Lionel | Neuer Manuel |
| Coach | Jamaica | Schäfer Winfried | Neuer Manuel | Robben Arjen | Messi Lionel |
| Coach | Japan | Aguirre Javier | Courtois Thibaut | Neuer Manuel | Iniesta Andres |
| Coach | Jordan | Wilkins Ray | Messi Lionel | Cristiano Ronaldo | Neymar |
| Coach | Kazakhstan | Krasnozhan Juri | Robben Arjen | Cristiano Ronaldo | Di Maria Angel |
| Coach | Kenya | Williamson Robert | Mueller Thomas | Iniesta Andres | Costa Diego |
| Coach | Korea Republic | Stielike Ulrich Otto | Cristiano Ronaldo | Neuer Manuel | Di Maria Angel |
| Coach | Kuwait | Vieira Jorvan | Cristiano Ronaldo | Neuer Manuel | Mueller Thomas |
| Coach | Kyrgyzstan | Krestinin Aleksandr | Mueller Thomas | Neuer Manuel | Lahm Philipp |
| Coach | Laos | Booth David | Cristiano Ronaldo | Messi Lionel | Lahm Philipp |
| Coach | Latvia | Pahars Marians | Messi Lionel | Robben Arjen | Neuer Manuel |
| Coach | Lebanon | Giannini Giuseppe | Cristiano Ronaldo | Messi Lionel | Neuer Manuel |
| Coach | Lesotho | Matete Seephephe | Messi Lionel | Cristiano Ronaldo | Toure Yaya |
| Coach | Liberia | Kojo Thomas | Cristiano Ronaldo | Messi Lionel | Costa Diego |
| Coach | Liechtenstein | Pauritsch Rene | Cristiano Ronaldo | Robben Arjen | Neuer Manuel |
| Coach | Lithuania | Pankratjevas Igoris | Messi Lionel | Neuer Manuel | Robben Arjen |
| Coach | Luxembourg | Holtz Luc | Cristiano Ronaldo | Neuer Manuel | Neymar |
| Coach | Macau | Leung Sui Wing | Messi Lionel | Neuer Manuel | Robben Arjen |
| Coach | Madagascar | Rajaonarisamba Franck | Cristiano Ronaldo | Messi Lionel | Neuer Manuel |
| Coach | Malawi | Chimodzi Young | Cristiano Ronaldo | Messi Lionel | Neymar |
| Coach | Maldives | Mamic Drago | Cristiano Ronaldo | Messi Lionel | Neymar |
| Coach | Mali | Kasperczak Henri | Neuer Manuel | Cristiano Ronaldo | Mascherano Javier |
| Coach | Malta | Ghedin Pietro | Goetze Mario | Mueller Thomas | Robben Arjen |
| Coach | Mauritania | Martins Corentin | Cristiano Ronaldo | Messi Lionel | Costa Diego |
| Coach | Mauritius | Patel Ackbar | Cristiano Ronaldo | Messi Lionel | Mueller Thomas |
| Coach | Mexico | Herrera Aguirre Miguel Ernesto | Cristiano Ronaldo | Lahm Philipp | Kroos Toni |
| Coach | Moldova | Curteian Alexandr | Neuer Manuel | Robben Arjen | Messi Lionel |
| Coach | Mongolia | Lkhgvajav Dorjjav | Mueller Thomas | Goetze Mario | Messi Lionel |
| Coach | Montenegro | Brnovic Branko | Cristiano Ronaldo | Mueller Thomas | Messi Lionel |

| Vote | Country | Name | First (5 points) | Second (3 points) | Third (1 point) |
| --- | --- | --- | --- | --- | --- |
| Coach | Montserrat | Hewlett Lenny | Neuer Manuel | Cristiano Ronaldo | Messi Lionel |
| Coach | Morocco | Badou Ezzaki | Cristiano Ronaldo | Messi Lionel | Neuer Manuel |
| Coach | Mozambique | Chissano João António | Robben Arjen | Messi Lionel | Cristiano Ronaldo |
| Coach | Myanmar | Radojko Avremovic | Messi Lionel | Neuer Manuel | Cristiano Ronaldo |
| Coach | Namibia | Mannetti Ricardo | Cristiano Ronaldo | Mueller Thomas | Messi Lionel |
| Coach | Nepal | Stefanovski Jack | Cristiano Ronaldo | Messi Lionel | Costa Diego |
| Coach | Netherlands | Hiddink Guus | Robben Arjen | Mueller Thomas | Cristiano Ronaldo |
| Coach | New Caledonia | Moizan Alain | Cristiano Ronaldo | Neuer Manuel | Mueller Thomas |
| Coach | New Zealand | Hudson Anthony | Cristiano Ronaldo | Costa Diego | Bale Gareth |
| Coach | Nicaragua | Londoño Sánchez Luis Javier | Cristiano Ronaldo | Mueller Thomas | Lahm Philipp |
| Coach | Niger | Diabate Oumarou Saidou | Neuer Manuel | Schweinsteiger Bastian | Toure Yaya |
| Coach | Nigeria | Keshi Stephen | Cristiano Ronaldo | Toure Yaya | Lahm Philipp |
| Coach | Norway | Høgmo Per-Mathias | Cristiano Ronaldo | Robben Arjen | Neuer Manuel |
| Coach | Oman | Le Guen Paul | Cristiano Ronaldo | Neuer Manuel | Benzema Karim |
| Coach | Palestine | Alhasan Ahmed | Messi Lionel | Neuer Manuel | Neymar |
| Coach | Panama | Gomez Hernán Dario | Messi Lionel | Cristiano Ronaldo | Kroos Toni |
| Coach | Paraguay | Genes Vïctor | Cristiano Ronaldo | Messi Lionel | Mueller Thomas |
| Coach | Peru | Bengoechea Pablo | Cristiano Ronaldo | Neymar | Messi Lionel |
| Coach | Philippines | Dooley Thomas Dennis | Messi Lionel | Cristiano Ronaldo | Neuer Manuel |
| Coach | Poland | Nawalka Adam | Neuer Manuel | Cristiano Ronaldo | Messi Lionel |
| Coach | Portugal | Santos Fernando | Cristiano Ronaldo | Neuer Manuel | Robben Arjen |
| Coach | Puerto Rico | Barros Vitor Hugo | Neuer Manuel | Robben Arjen | Cristiano Ronaldo |
| Coach | Qatar | Belmadi Djamel | Cristiano Ronaldo | Neuer Manuel | Kroos Toni |
| Coach | Republic of Ireland | O'Neill Martin | Cristiano Ronaldo | Messi Lionel | Costa Diego |
| Coach | Romania | Anghel Iordanescu | Cristiano Ronaldo | Mueller Thomas | Messi Lionel |
| Coach | Russia | Capello Fabio | Neuer Manuel | Cristiano Ronaldo | Messi Lionel |
| Coach | Rwanda | Constantine Stephen | Cristiano Ronaldo | Messi Lionel | Rodriguez James |
| Coach | Samoa | Young Phineas | Neuer Manuel | Goetze Mario | Messi Lionel |
| Coach | San Marino | Manzaroli Pierangelo | Neuer Manuel | Cristiano Ronaldo | Mueller Thomas |
| Coach | Saudi Arabia | Lopez Caro Juan Ramon | Iniesta Andres | Cristiano Ronaldo | Messi Lionel |
| Coach | Scotland | Strachan Gordon | Cristiano Ronaldo | Costa Diego | Robben Arjen |
| Coach | Senegal | Giresse Alain | Cristiano Ronaldo | Messi Lionel | Neuer Manuel |
| Coach | Serbia | Curcic Radovan | Cristiano Ronaldo | Messi Lionel | Schweinsteiger Bastian |

| Vote | Country | Name | First (5 points) | Second (3 points) | Third (1 point) |
| --- | --- | --- | --- | --- | --- |
| Coach | Seychelles | Mathiot Ulric | Cristiano Ronaldo | Robben Arjen | Messi Lionel |
| Coach | Sierra Leone | Sesay Ajina | Cristiano Ronaldo | Messi Lionel | Neymar |
| Coach | Singapore | Stange Bernd | Neuer Manuel | Mueller Thomas | Messi Lionel |
| Coach | Slovakia | Kozak Jan | Cristiano Ronaldo | Neuer Manuel | Ibrahimovic Zlatan |
| Coach | Slovenia | Katanec Srečko | Mueller Thomas | Cristiano Ronaldo | Neuer Manuel |
| Coach | Solomon Islands | Moli Jacob | Neuer Manuel | Mueller Thomas | Lahm Philipp |
| Coach | South Africa | Mashaba Ephraim | Messi Lionel | Neuer Manuel | Pogba Paul |
| Coach | South Sudan | Lee Sung Jea | Kroos Toni | Messi Lionel | Costa Diego |
| Coach | Spain | Del Bosque Vicente | Cristiano Ronaldo | Lahm Philipp | Ramos Sergio |
| Coach | Sri Lanka | Kavazovic Nicola | Neuer Manuel | Kroos Toni | Mueller Thomas |
| Coach | St. Kitts and Nevis | Hazel Jeffery | Cristiano Ronaldo | Messi Lionel | Mueller Thomas |
| Coach | St. Lucia | Lastic Francis | Goetze Mario | Costa Diego | Rodriguez James |
| Coach | St. Vincent and the  Grenadines | Huggins Cornelius | Messi Lionel | Cristiano Ronaldo | Robben Arjen |
| Coach | Sudan | Ahmed Mohamed Abdalla | Lahm Philipp | Messi Lionel | Mueller Thomas |
| Coach | Suriname | Godeken Roberto | Cristiano Ronaldo | Kroos Toni | Messi Lionel |
| Coach | Swaziland | Bulunga Harries | Cristiano Ronaldo | Neuer Manuel | Messi Lionel |
| Coach | Sweden | Hamrén Erik | Ibrahimovic Zlatan | Neuer Manuel | Messi Lionel |
| Coach | Switzerland | Petkovic Vladimir | Cristiano Ronaldo | Neuer Manuel | Robben Arjen |
| Coach | Syria | Al Fakeer Muhannad | Messi Lionel | Cristiano Ronaldo | Iniesta Andres |
| Coach | Tahiti | Etaeta Eddy | Neuer Manuel | Benzema Karim | Ibrahimovic Zlatan |
| Coach | Tajikistan | Mukhamadiev Mukhsin | Neuer Manuel | Robben Arjen | Mueller Thomas |
| Coach | Tanzania | Nooij Mart | Robben Arjen | Messi Lionel | Cristiano Ronaldo |
| Coach | Thailand | Senamuang Kiatisuk | Cristiano Ronaldo | Messi Lionel | Lahm Philipp |
| Coach | Turkey | Terim Fatih | Cristiano Ronaldo | Robben Arjen | Benzema Karim |
| Coach | Turks and Caicos Islands | Harrington Craig | Cristiano Ronaldo | Bale Gareth | Hazard Eden |
| Coach | Ukraine | Fomenko Mykhailo | Cristiano Ronaldo | Messi Lionel | Neuer Manuel |
| Coach | United Arab Emirates | Redah Mahdi | Cristiano Ronaldo | Robben Arjen | Mueller Thomas |
| Coach | Uruguay | Tabárez Oscar | Cristiano Ronaldo | Neuer Manuel | Robben Arjen |
| Coach | US Virgin Islands | Appleton Leonard | Cristiano Ronaldo | Costa Diego | Messi Lionel |
| Coach | USA | Klinsmann Jurgen | Neuer Manuel | Cristiano Ronaldo | Robben Arjen |
| Coach | Uzbekistan | Kasimov Mirdjalal | Mueller Thomas | Messi Lionel | Robben Arjen |
| Coach | Venezuela | Sanvicente Noel | Rodriguez James | Robben Arjen | Messi Lionel |

| Vote | Country | Name | First (5 points) | Second (3 points) | Third (1 point) |
| --- | --- | --- | --- | --- | --- |
| Coach | Vietnam | Toshiya Miura | Cristiano Ronaldo | Neuer Manuel | Bale Gareth |
| Coach | Wales | Coleman Christopher | Bale Gareth | Cristiano Ronaldo | Schweinsteiger Bastian |
| Coach | Zambia | Honour Janza | Cristiano Ronaldo | Messi Lionel | Neuer Manuel |
| Coach | Zimbabwe | Kalisto Pasuwa | Cristiano Ronaldo | Messi Lionel | Toure Yaya |
| Media | Albania | Dizdari Besnik | Neuer Manuel | Cristiano Ronaldo | Messi Lionel |
| Media | Algeria | Ouahib Yazid | Cristiano Ronaldo | Neuer Manuel | Messi Lionel |
| Media | Andorra | Duaso Victor | Neuer Manuel | Mueller Thomas | Di Maria Angel |
| Media | Angola | Goncalves Mateus | Cristiano Ronaldo | Di Maria Angel | Neuer Manuel |
| Media | Antigua and Barbuda | Neto Baptiste | Goetze Mario | Messi Lionel | Neymar |
| Media | Argentina | Sacco Enrique | Messi Lionel | Cristiano Ronaldo | Neuer Manuel |
| Media | Armenia | Baghdasarian Souren | Neuer Manuel | Cristiano Ronaldo | Messi Lionel |
| Media | Aruba | Kock Ricardo | Mueller Thomas | Messi Lionel | Cristiano Ronaldo |
| Media | Australia | Foster Craig | Neuer Manuel | Cristiano Ronaldo | Lahm Philipp |
| Media | Austria | Kowatsch-Schwarz Walter | Cristiano Ronaldo | Neuer Manuel | Lahm Philipp |
| Media | Azerbaijan | Mövsümov Rasim | Cristiano Ronaldo | Lahm Philipp | Robben Arjen |
| Media | Bahrain | Ashoor Abdullah | Cristiano Ronaldo | Neuer Manuel | Neymar |
| Media | Bangladesh | Mahamud Raihan | Cristiano Ronaldo | Messi Lionel | Neuer Manuel |
| Media | Belarus | Nikolaev Sergey | Cristiano Ronaldo | Mueller Thomas | Robben Arjen |
| Media | Belgium | Dubois Michel | Neuer Manuel | Cristiano Ronaldo | Rodriguez James |
| Media | Belize | Morales Iglesias Ruben | Messi Lionel | Rodriguez James | Cristiano Ronaldo |
| Media | Benin | Sagbo René | Cristiano Ronaldo | Messi Lionel | Neymar |
| Media | Bermuda | Burton James | Cristiano Ronaldo | Messi Lionel | Neuer Manuel |
| Media | Bhutan | Wangdi Phuntsho | Rodriguez James | Pogba Paul | Mueller Thomas |
| Media | Bolivia | Rivera Carlos Enrique | Rodriguez James | Mascherano Javier | Neymar |
| Media | Bosnia-Herzegovina | Buric Ahmed | Mueller Thomas | Cristiano Ronaldo | Ibrahimovic Zlatan |
| Media | Botswana | Mathala Oaitse | Cristiano Ronaldo | Messi Lionel | Robben Arjen |
| Media | Brazil | Machado Cleber | Cristiano Ronaldo | Robben Arjen | Mueller Thomas |
| Media | Brunei Darussalam | Pg Salleh Ak Fauzi | Cristiano Ronaldo | Messi Lionel | Mueller Thomas |
| Media | Bulgaria | Savov Michel | Neuer Manuel | Robben Arjen | Di Maria Angel |
| Media | Burkina Faso | Hien Victorien Marie | Mueller Thomas | Costa Diego | Ramos Sergio |
| Media | Burma | Zaw Linn Kyaw | Cristiano Ronaldo | Mascherano Javier | Kroos Toni |
| Media | Cambodia | Chamroeun Ung | Cristiano Ronaldo | Mueller Thomas | Costa Diego |
| Media | Cameroon | Mbeze Brice | Neuer Manuel | Benzema Karim | Neymar |

| Vote | Country | Name | First (5 points) | Second (3 points) | Third (1 point) |
| --- | --- | --- | --- | --- | --- |
| Media | Canada | Davidson Neil | Cristiano Ronaldo | Mueller Thomas | Messi Lionel |
| Media | Cape Verde Islands | Amaral André | Mueller Thomas | Cristiano Ronaldo | Di Maria Angel |
| Media | Cayman Islands | Shillingford Ron | Cristiano Ronaldo | Messi Lionel | Neymar |
| Media | Chad | Hissein Atti Alifa | Cristiano Ronaldo | Neymar | Benzema Karim |
| Media | Chile | Diaz Danilo | Robben Arjen | Cristiano Ronaldo | Rodriguez James |
| Media | China PR | Ming Luo | Cristiano Ronaldo | Neuer Manuel | Mueller Thomas |
| Media | Colombia | Andrade Paché | Schweinsteiger Bastian | Cristiano Ronaldo | Rodriguez James |
| Media | Comoros | Boina Abdou | Cristiano Ronaldo | Lahm Philipp | Robben Arjen |
| Media | Congo | Eloue James Golden | Cristiano Ronaldo | Messi Lionel | Neymar |
| Media | Congo DR | Kabelu Eddy | Cristiano Ronaldo | Goetze Mario | Robben Arjen |
| Media | Costa Rica | Calvo Castro Rodrigo | Kroos Toni | Cristiano Ronaldo | Rodriguez James |
| Media | Côte d'Ivoire | Khalil Adam | Cristiano Ronaldo | Toure Yaya | Neuer Manuel |
| Media | Croatia | Reic Zdravko | Cristiano Ronaldo | Neuer Manuel | Rodriguez James |
| Media | Cuba | Hernandez Miguel | Cristiano Ronaldo | Messi Lionel | Neuer Manuel |
| Media | Curaçao | Dunker Nino | Cristiano Ronaldo | Robben Arjen | Neuer Manuel |
| Media | Cyprus | Gavrielides Michel | Cristiano Ronaldo | Neuer Manuel | Robben Arjen |
| Media | Czech Republic | Hrabe Stanislav | Mueller Thomas | Robben Arjen | Cristiano Ronaldo |
| Media | Denmark | Thogersen Troels Bager | Cristiano Ronaldo | Robben Arjen | Lahm Philipp |
| Media | Djibouti | Mohamed Kenadid | Benzema Karim | Ibrahimovic Zlatan | Messi Lionel |
| Media | Dominican Republic | Bauger Jorge Rolando | Cristiano Ronaldo | Messi Lionel | Robben Arjen |
| Media | Ecuador | Zavala Garcia Fabricio | Cristiano Ronaldo | Neuer Manuel | Messi Lionel |
| Media | Egypt | Mazhar Inas | Cristiano Ronaldo | Mueller Thomas | Robben Arjen |
| Media | El Salvador | Posada Flores Mario Ernesto | Neuer Manuel | Cristiano Ronaldo | Messi Lionel |
| Media | England | Winter Henry | Cristiano Ronaldo | Neuer Manuel | Lahm Philipp |
| Media | Eritrea | Seium Michael | Neuer Manuel | Robben Arjen | Cristiano Ronaldo |
| Media | Estonia | Süvari Aet | Neuer Manuel | Cristiano Ronaldo | Messi Lionel |
| Media | Ethiopia | Abdulkeni Mensur | Neuer Manuel | Cristiano Ronaldo | Lahm Philipp |
| Media | Faroe Islands | Tróndur Arge | Cristiano Ronaldo | Messi Lionel | Neuer Manuel |
| Media | Fiji | Kumar Rashneel | Cristiano Ronaldo | Neymar | Rodriguez James |
| Media | Finland | Kanerva Juha | Neuer Manuel | Mueller Thomas | Cristiano Ronaldo |
| Media | France | Ejnes Gérard | Cristiano Ronaldo | Robben Arjen | Neuer Manuel |
| Media | FYR Macedonia | Timkovski Boro | Neuer Manuel | Cristiano Ronaldo | Costa Diego |
| Media | Gabon | Loundou James Angelo | Cristiano Ronaldo | Neuer Manuel | Messi Lionel |

| Vote | Country | Name | First (5 points) | Second (3 points) | Third (1 point) |
| --- | --- | --- | --- | --- | --- |
| Media | Gambia | Camara Baboucar | Cristiano Ronaldo | Neuer Manuel | Robben Arjen |
| Media | Georgia | Potskhveria Zurab | Cristiano Ronaldo | Messi Lionel | Bale Gareth |
| Media | Germany | Wild Karlheinz | Neuer Manuel | Mueller Thomas | Lahm Philipp |
| Media | Ghana | Oti Adjei Michael | Cristiano Ronaldo | Lahm Philipp | Messi Lionel |
| Media | Greece | Staramopoulos Manos | Neuer Manuel | Robben Arjen | Cristiano Ronaldo |
| Media | Grenada | Bascombe Michael | Mueller Thomas | Messi Lionel | Toure Yaya |
| Media | Guatemala | Aguilar Francisco | Neuer Manuel | Lahm Philipp | Cristiano Ronaldo |
| Media | Guinea | Diallo Ibrahima | Cristiano Ronaldo | Neuer Manuel | Neymar |
| Media | Guinea-Bissau | Ucha Etiandro | Cristiano Ronaldo | Hazard Eden | Messi Lionel |
| Media | Guyana | Campbell Emmerson | Cristiano Ronaldo | Neymar | Messi Lionel |
| Media | Haiti | Nere Enock | Neuer Manuel | Cristiano Ronaldo | Messi Lionel |
| Media | Honduras | Garcia Francisco Antonio Rivas | Cristiano Ronaldo | Mueller Thomas | Neymar |
| Media | Hong Kong | Alvarado Gabby | Cristiano Ronaldo | Neuer Manuel | Messi Lionel |
| Media | Hungary | Csillag Péter | Cristiano Ronaldo | Lahm Philipp | Neuer Manuel |
| Media | Iceland | Sigurdsson Vidir | Cristiano Ronaldo | Messi Lionel | Neuer Manuel |
| Media | India | Sarkar Dhiman | Mueller Thomas | Lahm Philipp | Neuer Manuel |
| Media | Indonesia | Saleh Nurdin | Cristiano Ronaldo | Neuer Manuel | Robben Arjen |
| Media | Iran | Rahmani Siamak | Cristiano Ronaldo | Mueller Thomas | Messi Lionel |
| Media | Iraq | Abdul Emam Sami | Cristiano Ronaldo | Neuer Manuel | Di Maria Angel |
| Media | Ireland | Kelly Paul | Cristiano Ronaldo | Mueller Thomas | Messi Lionel |
| Media | Israel | Klieger Noah | Cristiano Ronaldo | Neuer Manuel | Robben Arjen |
| Media | Italy | Condo Paolo | Cristiano Ronaldo | Neuer Manuel | Di Maria Angel |
| Media | Japan | Tamura Shuichi | Neuer Manuel | Kroos Toni | Cristiano Ronaldo |
| Media | Kazakhstan | Tulegenov Geniy | Neuer Manuel | Cristiano Ronaldo | Messi Lionel |
| Media | Kenya | Nyende Charles | Cristiano Ronaldo | Neuer Manuel | Messi Lionel |
| Media | Kuwait | Al Attia Abd Al Aziz | Cristiano Ronaldo | Neuer Manuel | Messi Lionel |
| Media | Kyrgyzstan | Louzanov Pavel | Robben Arjen | Mueller Thomas | Neymar |
| Media | Latvia | Koscinkevics Ilvars | Cristiano Ronaldo | Lahm Philipp | Messi Lionel |
| Media | Lebanon | Fawaz Mohamed | Neuer Manuel | Cristiano Ronaldo | Messi Lionel |
| Media | Lesotho | Matjama Thabang | Robben Arjen | Pogba Paul | Bale Gareth |
| Media | Liberia | Carter James Burgess | Cristiano Ronaldo | Messi Lionel | Robben Arjen |
| Media | Libya | Cutajar Joseph | Cristiano Ronaldo | Neuer Manuel | Messi Lionel |
| Media | Liechtenstein | Hasler Ernst | Di Maria Angel | Neuer Manuel | Rodriguez James |

| Vote | Country | Name | First (5 points) | Second (3 points) | Third (1 point) |
| --- | --- | --- | --- | --- | --- |
| Media | Lithuania | Janonis Giedrius | Neuer Manuel | Cristiano Ronaldo | Messi Lionel |
| Media | Luxembourg | Hiegel Didier | Cristiano Ronaldo | Messi Lionel | Neuer Manuel |
| Media | Macau | Santos Pedro André | Cristiano Ronaldo | Robben Arjen | Di Maria Angel |
| Media | Madagascar | Rabary Clément | Neymar | Mueller Thomas | Benzema Karim |
| Media | Malawi | Kanjere Peter | Neuer Manuel | Cristiano Ronaldo | Messi Lionel |
| Media | Malaysia | Hashim Rizal | Lahm Philipp | Neuer Manuel | Cristiano Ronaldo |
| Media | Maldives | Ali Shimaz | Cristiano Ronaldo | Messi Lionel | Neymar |
| Media | Mali | Bobo Tounkara Souleymane | Cristiano Ronaldo | Messi Lionel | Mueller Thomas |
| Media | Malta | Camenzuli Charles | Cristiano Ronaldo | Neuer Manuel | Pogba Paul |
| Media | Mauritania | Ould El Hacen Mohamed | Robben Arjen | Bale Gareth | Di Maria Angel |
| Media | Mauritius | Hydoo Azmaal | Cristiano Ronaldo | Neuer Manuel | Messi Lionel |
| Media | Mexico | Aguilera Salvador | Cristiano Ronaldo | Kroos Toni | Di Maria Angel |
| Media | Moldova | Donets Serghei | Neuer Manuel | Robben Arjen | Messi Lionel |
| Media | Mongolia | Tsagaanbaatar Byambaa | Mueller Thomas | Cristiano Ronaldo | Costa Diego |
| Media | Montenegro | Mitrovic Danilo | Cristiano Ronaldo | Mueller Thomas | Neuer Manuel |
| Media | Morocco | Badri Mostafa | Cristiano Ronaldo | Neuer Manuel | Lahm Philipp |
| Media | Mozambique | Zandamela Alexandre | Cristiano Ronaldo | Mueller Thomas | Messi Lionel |
| Media | Namibia | Nikodemus Sheefeni | Neuer Manuel | Robben Arjen | Cristiano Ronaldo |
| Media | Nepal | Raju Ghising | Neuer Manuel | Messi Lionel | Mueller Thomas |
| Media | Netherlands | Nieuwenhof Frans Van Den | Cristiano Ronaldo | Neuer Manuel | Robben Arjen |
| Media | New Caledonia | Cambefort Yoann | Mueller Thomas | Neuer Manuel | Benzema Karim |
| Media | New Zealand | Watson Gordon Glen | Cristiano Ronaldo | Messi Lionel | Neuer Manuel |
| Media | Nicaragua | Rosales Cruz Osman | Cristiano Ronaldo | Robben Arjen | Mueller Thomas |
| Media | Niger | Ganoua Mohamed Silimane | Kroos Toni | Neuer Manuel | Cristiano Ronaldo |
| Media | Nigeria | Audu Samm | Messi Lionel | Cristiano Ronaldo | Schweinsteiger Bastian |
| Media | North Korea | Dong Gyu Ri | Cristiano Ronaldo | Messi Lionel | Neymar |
| Media | Northern Ireland | Fullerton Jackie | Cristiano Ronaldo | Mueller Thomas | Messi Lionel |
| Media | Norway | Tjaernaas Lars | Cristiano Ronaldo | Neuer Manuel | Messi Lionel |
| Media | Oman | Al-Barhi Saleh | Cristiano Ronaldo | Neuer Manuel | Lahm Philipp |
| Media | Pakistan | Zafar Emad | Cristiano Ronaldo | Messi Lionel | Neuer Manuel |
| Media | Palestine | Iraqi Mohamad | Cristiano Ronaldo | Neuer Manuel | Hazard Eden |
| Media | Panama | Estrada Campo Elias | Cristiano Ronaldo | Robben Arjen | Neuer Manuel |
| Media | Paraguay | Da Rosa Ruben Dario | Cristiano Ronaldo | Messi Lionel | Rodriguez James |

| Vote | Country | Name | First (5 points) | Second (3 points) | Third (1 point) |
| --- | --- | --- | --- | --- | --- |
| Media | Peru | Salinas Carlos | Cristiano Ronaldo | Neuer Manuel | Rodriguez James |
| Media | Philippines | Fenix Ryan | Cristiano Ronaldo | Neuer Manuel | Mueller Thomas |
| Media | Poland | Iwanski Maciej | Cristiano Ronaldo | Messi Lionel | Neuer Manuel |
| Media | Portugal | Rita Joaquim | Cristiano Ronaldo | Robben Arjen | Di Maria Angel |
| Media | Puerto Rico | Arce Luis Santiago | Cristiano Ronaldo | Neuer Manuel | Messi Lionel |
| Media | Qatar | Alkhulaifi Majed | Cristiano Ronaldo | Neuer Manuel | Mueller Thomas |
| Media | Romania | Rosu Emmanuel | Neuer Manuel | Cristiano Ronaldo | Courtois Thibaut |
| Media | Russia | Kleshchev Konstantin | Cristiano Ronaldo | Robben Arjen | Mueller Thomas |
| Media | Rwanda | Mugabe Bonnie | Cristiano Ronaldo | Messi Lionel | Mueller Thomas |
| Media | San Marino | Gorini Elia | Mueller Thomas | Cristiano Ronaldo | Robben Arjen |
| Media | Saudi Arabia | Al.Solame Rjallah | Cristiano Ronaldo | Mueller Thomas | Messi Lionel |
| Media | Scotland | Greechan John | Cristiano Ronaldo | Neuer Manuel | Messi Lionel |
| Media | Senegal | Goloko Aliou | Cristiano Ronaldo | Neuer Manuel | Toure Yaya |
| Media | Serbia | Novak Vladimir | Cristiano Ronaldo | Messi Lionel | Schweinsteiger Bastian |
| Media | Seychelles | Govinden Gérard | Cristiano Ronaldo | Messi Lionel | Lahm Philipp |
| Media | Sierra Leone | Fajah Barrie Mohamed | Cristiano Ronaldo | Messi Lionel | Neuer Manuel |
| Media | Singapore | Lim Gary | Cristiano Ronaldo | Messi Lionel | Neuer Manuel |
| Media | Slovakia | Surin Peter | Cristiano Ronaldo | Neuer Manuel | Messi Lionel |
| Media | Slovenia | Stare Andrej | Cristiano Ronaldo | Neuer Manuel | Robben Arjen |
| Media | South Africa | Gleeson Mark | Lahm Philipp | Cristiano Ronaldo | Messi Lionel |
| Media | South Korea | Wee Won Seok | Cristiano Ronaldo | Neuer Manuel | Messi Lionel |
| Media | Spain | Relano Alfredo | Cristiano Ronaldo | Neuer Manuel | Messi Lionel |
| Media | St Kitts & Nevis | Thompson Merv-Ann | Cristiano Ronaldo | Messi Lionel | Neymar |
| Media | St. Lucia | James Lawrence | Goetze Mario | Rodriguez James | Messi Lionel |
| Media | Sudan | Abu Elgassim Muzamil | Cristiano Ronaldo | Mascherano Javier | Mueller Thomas |
| Media | Suriname | Romeo Desney | Cristiano Ronaldo | Kroos Toni | Neuer Manuel |
| Media | Swaziland | Dlamini Kenneth | Neuer Manuel | Cristiano Ronaldo | Messi Lionel |
| Media | Sweden | Ysten Henrik | Neuer Manuel | Cristiano Ronaldo | Lahm Philipp |
| Media | Switzerland | Cerf Christophe | Cristiano Ronaldo | Neuer Manuel | Robben Arjen |
| Media | Syria | Abou Shameh Louay | Cristiano Ronaldo | Di Maria Angel | Ibrahimovic Zlatan |
| Media | Tahiti | Ollivier Luc | Pogba Paul | Rodriguez James | Neuer Manuel |
| Media | Tajikistan | Buriev Alaveddine | Cristiano Ronaldo | Messi Lionel | Robben Arjen |
| Media | Tanzania | Wambura Boniface | Cristiano Ronaldo | Messi Lionel | Mueller Thomas |

| Vote | Country | Name | First (5 points) | Second (3 points) | Third (1 point) |
| --- | --- | --- | --- | --- | --- |
| Media | Thailand | Patoommawatana Urai | Neuer Manuel | Cristiano Ronaldo | Lahm Philipp |
| Media | Togo | Ayena Mathias | Cristiano Ronaldo | Goetze Mario | Neuer Manuel |
| Media | Trinidad and Tobago | Liburd Lasana | Cristiano Ronaldo | Di Maria Angel | Neymar |
| Media | Tunisia | Dhaifallah Abdesslam | Neuer Manuel | Cristiano Ronaldo | Messi Lionel |
| Media | Turkey | Manav Selçuk | Cristiano Ronaldo | Rodriguez James | Neuer Manuel |
| Media | Turkmenistan | Vershinin Alexander | Messi Lionel | Neuer Manuel | Cristiano Ronaldo |
| Media | Turks and Caicos Islands | Deo Faizool | Rodriguez James | Neuer Manuel | Lahm Philipp |
| Media | Uganda | Musisi Kiyingi Fredrick | Cristiano Ronaldo | Neuer Manuel | Messi Lionel |
| Media | Ukraine | Linnyk Igor | Mueller Thomas | Neuer Manuel | Cristiano Ronaldo |
| Media | United Arab Emirates | Mouadhen Dafrallah | Neuer Manuel | Robben Arjen | Cristiano Ronaldo |
| Media | Uruguay | Pineyrua Ricardo | Schweinsteiger Bastian | Robben Arjen | Messi Lionel |
| Media | USA | Kennedy Paul | Cristiano Ronaldo | Messi Lionel | Lahm Philipp |
| Media | Uzbekistan | Rtveladze Grigoriy | Cristiano Ronaldo | Robben Arjen | Mueller Thomas |
| Media | Vanuatu | Nasse Raymond | Bale Gareth | Pogba Paul | Ibrahimovic Zlatan |
| Media | Venezuela | Blavia Francisco | Cristiano Ronaldo | Messi Lionel | Mueller Thomas |
| Media | Vietnam | Anh Ngoc Truong | Cristiano Ronaldo | Messi Lionel | Neuer Manuel |
| Media | Wales | Abbandonato Paul | Cristiano Ronaldo | Bale Gareth | Mueller Thomas |
| Media | Yemen | Al Hababi Abdel | Cristiano Ronaldo | Messi Lionel | Ibrahimovic Zlatan |
| Media | Zambia | Lungu Chapadongo | Cristiano Ronaldo | Messi Lionel | Mueller Thomas |
| Media | Zimbabwe | Mabika Charles | Cristiano Ronaldo | Messi Lionel | Lahm Philipp |

| Vote | Country | Name | First (5 points) | Second (3 points) | Third (1 point) |
| --- | --- | --- | --- | --- | --- |
| Captain | Afghanistan | Shayesteh Faysal | Cristiano Ronaldo | Messi Lionel | Neymar |
| Captain | Albania | Cana Lorik | Cristiano Ronaldo | Messi Lionel | Neymar |
| Captain | Algeria | Medjani Carl | Messi Lionel | Cristiano Ronaldo | Neymar |
| Captain | American Samoa | Ott Ramin | Neymar | Messi Lionel | Cristiano Ronaldo |
| Captain | Andorra | Sonejee Oscar | Messi Lionel | Suárez Luis | Cristiano Ronaldo |
| Captain | Angola | Cardoso Dário | Messi Lionel | Cristiano Ronaldo | Neymar |
| Captain | Anguilla | Connor Girdon | Messi Lionel | Cristiano Ronaldo | Neymar |
| Captain | Antigua and Barbuda | Parker Joshua | Lewandowski Robert | Neymar | Messi Lionel |
| Captain | Argentina | Lionel Messi | Suárez Luis | Neymar | Iniesta Andrés |
| Captain | Armenia | Mkhitaryan Henrikh | Messi Lionel | Cristiano Ronaldo | Ibrahimovic Zlatan |
| Captain | Aruba | Abdul David | Messi Lionel | Cristiano Ronaldo | Neymar |
| Captain | Australia | Jedinak Mile | Cristiano Ronaldo | Lewandowski Robert | Neymar |
| Captain | Austria | Fuchs Christian | Neuer Manuel | Hazard Eden | Cristiano Ronaldo |
| Captain | Azerbaijan | Sadigov Rashad | Messi Lionel | Cristiano Ronaldo | Suárez Luis |
| Captain | Bahamas | Stfluer Lesly | Messi Lionel | Neymar | Lewandowski Robert |
| Captain | Bangladesh | Mamun Md Mamunul Islam | Messi Lionel | Cristiano Ronaldo | Neymar |
| Captain | Belarus | Martynovich Aliaksandr | Cristiano Ronaldo | Messi Lionel | Lewandowski Robert |
| Captain | Belgium | Company Vincent | Hazard Eden | De Bruyne Kevin | Messi Lionel |
| Captain | Bermuda | Bean Wayne | Messi Lionel | Müller Thomas | Vidal Arturo |
| Captain | Bhutan | Gurung Karun | Cristiano Ronaldo | Messi Lionel | Pogba Paul |
| Captain | Bosnia and Herzegovina | Edin Džeko | Cristiano Ronaldo | Hazard Eden | Messi Lionel |
| Captain | Botswana | Mogorosi Joel | Cristiano Ronaldo | Suárez Luis | Touré Yaya |
| Captain | Brazil | Da Silva Santos Junior Neymar | Messi Lionel | Suárez Luis | Rakitic Ivan |
| Captain | British Virgin Islands | Davis Andy | Messi Lionel | Cristiano Ronaldo | Ibrahimovic Zlatan |
| Captain | Brunei Darussalam | Hamzah Hazwan | Cristiano Ronaldo | Messi Lionel | Iniesta Andrés |
| Captain | Bulgaria | Dyakov Svetoslav | Messi Lionel | Cristiano Ronaldo | Iniesta Andrés |
| Captain | Burkina Faso | Kabore Charles | Cristiano Ronaldo | Messi Lionel | Lewandowski Robert |
| Captain | Burundi | Ndikumana Yamin Selemani | Messi Lionel | Neymar | Cristiano Ronaldo |
| Captain | Cambodia | Theary Bin Chanthavy | Cristiano Ronaldo | Messi Lionel | Neymar |
| Captain | Cameroon | Mbia Etoundi Stephane | Touré Yaya | Ibrahimovic Zlatan | Neuer Manuel |
| Captain | Canada | De Guzman Julian | Messi Lionel | Cristiano Ronaldo | Suárez Luis |
| Captain | Cape Verde Islands | Soares Marco | Cristiano Ronaldo | Messi Lionel | Neymar |
| Captain | Cayman Islands | Rivers Abijah | Messi Lionel | Cristiano Ronaldo | Suárez Luis |

| Vote | Country | Name | First (5 points) | Second (3 points) | Third (1 point) |
| --- | --- | --- | --- | --- | --- |
| Captain | Chad | Ndouassel Ezechiel | Messi Lionel | Cristiano Ronaldo | Neymar |
| Captain | China PR | Zheng Zhi | Messi Lionel | Hazard Eden | Müller Thomas |
| Captain | Colombia | Rodriguez Rubio James David | Cristiano Ronaldo | Benzema Karim | Bale Gareth |
| Captain | Congo DR | Mulumbu Youssouf | Messi Lionel | Cristiano Ronaldo | Touré Yaya |
| Captain | Cook Islands | Stuart Jake | Messi Lionel | Cristiano Ronaldo | Neymar |
| Captain | Costa Rica | Ruiz Bryan | Neymar | Messi Lionel | Cristiano Ronaldo |
| Captain | Côte d'Ivoire | Yao Kouassi Gervais | Messi Lionel | Cristiano Ronaldo | Hazard Eden |
| Captain | Croatia | Srna Darijo | Messi Lionel | Cristiano Ronaldo | Müller Thomas |
| Captain | Cuba | Luis Saez Daniel | Messi Lionel | Cristiano Ronaldo | Neymar |
| Captain | Curaçao | Martina Rhu-Endly | Ibrahimovic Zlatan | Messi Lionel | Cristiano Ronaldo |
| Captain | Cyprus | Charalambides Constantinos | Messi Lionel | Cristiano Ronaldo | Robben Arjen |
| Captain | Czech Republic | Cech Petr | Messi Lionel | Lewandowski Robert | Hazard Eden |
| Captain | Denmark | Agger Daniel | Messi Lionel | Cristiano Ronaldo | Suárez Luis |
| Captain | Djibouti | Mohamed Kadar Ahmed | Messi Lionel | Agüero Sergio | Cristiano Ronaldo |
| Captain | Dominica | Glenson Prince | Messi Lionel | Cristiano Ronaldo | Agüero Sergio |
| Captain | Dominican Republic | Odalis Baez | Cristiano Ronaldo | Suárez Luis | Neuer Manuel |
| Captain | Ecuador | Ayovi Corozo Walter | Neymar | Messi Lionel | Cristiano Ronaldo |
| Captain | Egypt | Ghaly Hossam | Messi Lionel | Cristiano Ronaldo | Agüero Sergio |
| Captain | England | Rooney Wayne | Messi Lionel | Müller Thomas | Cristiano Ronaldo |
| Captain | Estonia | Klavan Ragnar | Messi Lionel | Hazard Eden | Lewandowski Robert |
| Captain | Faroe Islands | Benjaminsen Fróði | Messi Lionel | Cristiano Ronaldo | Neymar |
| Captain | Fiji | Roy Krishna | Messi Lionel | Cristiano Ronaldo | Robben Arjen |
| Captain | Finland | Moisander Niklas | Messi Lionel | Cristiano Ronaldo | Ibrahimovic Zlatan |
| Captain | France | Lloris Hugo | Messi Lionel | Cristiano Ronaldo | Neymar |
| Captain | FYR Macedonia | Shikov Vanche | Cristiano Ronaldo | Pogba Paul | Lewandowski Robert |
| Captain | Georgia | Kankava Jaba | Messi Lionel | Cristiano Ronaldo | Neuer Manuel |
| Captain | Germany | Schweinsteiger Bastian | Neuer Manuel | Müller Thomas | Neymar |
| Captain | Ghana | Gyan Asamoah | Messi Lionel | Neymar | Cristiano Ronaldo |
| Captain | Guam | Cunliffe Jason | Messi Lionel | Cristiano Ronaldo | Sánchez Alexis |
| Captain | Guatemala | Ruiz Carlos Humberto | Mascherano Javier | Cristiano Ronaldo | Rodríguez James |
| Captain | Guinea | Pogba Florentin | Messi Lionel | Cristiano Ronaldo | Neuer Manuel |
| Captain | Guinea-Bissau | Cá Bocundji | Cristiano Ronaldo | Messi Lionel | Ibrahimovic Zlatan |
| Captain | Honduras | Valladares Noel Eduardo | Cristiano Ronaldo | Messi Lionel | Neymar |

| Vote | Country | Name | First (5 points) | Second (3 points) | Third (1 point) |
| --- | --- | --- | --- | --- | --- |
| Captain | Hong Kong | Yapp Hung Fai | Messi Lionel | Neuer Manuel | Pogba Paul |
| Captain | Hungary | Dzsudzsák Balázs | Messi Lionel | Cristiano Ronaldo | Neymar |
| Captain | Iceland | Gunnarsson Aron | Cristiano Ronaldo | Messi Lionel | Müller Thomas |
| Captain | India | Chettri Sunil | Messi Lionel | Cristiano Ronaldo | Neymar |
| Captain | Iran | Timotian Andranik | Messi Lionel | Cristiano Ronaldo | Neymar |
| Captain | Israel | Ben Haim Tal | Cristiano Ronaldo | Messi Lionel | Agüero Sergio |
| Captain | Jamaica | Rodolph Austin | Messi Lionel | Cristiano Ronaldo | Suárez Luis |
| Captain | Japan | Hasebe Makoto | Messi Lionel | Cristiano Ronaldo | Neuer Manuel |
| Captain | Jordan | Shafei Amer | Cristiano Ronaldo | Kroos Toni | Agüero Sergio |
| Captain | Kazakhstan | Smakov Samat | Messi Lionel | Cristiano Ronaldo | Hazard Eden |
| Captain | Korea Republic | Ki Sungyueng | Messi Lionel | Mascherano Javier | Neymar |
| Captain | Kyrgyzstan | Samsaliev Talant | Ibrahimovic Zlatan | Neuer Manuel | Müller Thomas |
| Captain | Laos | Sayavouthi Khampeng | Messi Lionel | Ibrahimovic Zlatan | Müller Thomas |
| Captain | Latvia | Gorkšs Kaspars | Lewandowski Robert | Messi Lionel | Müller Thomas |
| Captain | Lebanon | Antar Roda | Cristiano Ronaldo | Sánchez Alexis | Messi Lionel |
| Captain | Liberia | Dennis Teah | Cristiano Ronaldo | Benzema Karim | Kroos Toni |
| Captain | Liechtenstein | Frick Mario | Messi Lionel | Lewandowski Robert | Cristiano Ronaldo |
| Captain | Lithuania | Panka Mindaugas | Messi Lionel | Cristiano Ronaldo | Mascherano Javier |
| Captain | Luxembourg | Mutsch Mario | Messi Lionel | Iniesta Andrés | Neuer Manuel |
| Captain | Macau | Cheang Cheng Leong Paulo | Messi Lionel | Cristiano Ronaldo | Neuer Manuel |
| Captain | Madagascar | Paul Johann | Messi Lionel | Neymar | Cristiano Ronaldo |
| Captain | Malaysia | Rahim Safiq | Cristiano Ronaldo | Sánchez Alexis | Neymar |
| Captain | Maldives | Ashfaq Ali | Messi Lionel | Cristiano Ronaldo | Suárez Luis |
| Captain | Malta | Mifsud Michael | Müller Thomas | Neuer Manuel | Iniesta Andrés |
| Captain | Mauritania | N'Diaye Oumar | Mascherano Javier | Cristiano Ronaldo | Neymar |
| Captain | Mauritius | Bru Jonathan | Cristiano Ronaldo | Messi Lionel | Vidal Arturo |
| Captain | Mexico | Guardado Hernández José Andrés | Messi Lionel | Cristiano Ronaldo | Neymar |
| Captain | Moldova | Armas Igor | Messi Lionel | Cristiano Ronaldo | Neymar |
| Captain | Mongolia | Baysgalan Garidmagnai | Cristiano Ronaldo | Messi Lionel | Iniesta Andrés |
| Captain | Montenegro | Vucinic Mirko | Messi Lionel | Cristiano Ronaldo | Ibrahimovic Zlatan |
| Captain | Montserrat | Griffith Anthony | Lewandowski Robert | Agüero Sergio | Cristiano Ronaldo |
| Captain | Morocco | El Moutaqui Mehdi | Lewandowski Robert | Vidal Arturo | Pogba Paul |
| Captain | Mozambique | Pelembe Elias Gaspar | Messi Lionel | Cristiano Ronaldo | Neymar |

| Vote | Country | Name | First (5 points) | Second (3 points) | Third (1 point) |
| --- | --- | --- | --- | --- | --- |
| Captain | Myanmar | Kyaw Yan Aung | Cristiano Ronaldo | Neymar | Suárez Luis |
| Captain | Namibia | Ketjijere Ronald | Messi Lionel | Cristiano Ronaldo | Lewandowski Robert |
| Captain | Nepal | Gurung Anil | Cristiano Ronaldo | Messi Lionel | De Bruyne Kevin |
| Captain | Netherlands | Robben Arjen | Messi Lionel | Neymar | Suárez Luis |
| Captain | New Caledonia | Wadriako Jean Brice | Iniesta Andrés | Ibrahimovic Zlatan | Benzema Karim |
| Captain | New Zealand | Wood Chris | Lewandowski Robert | Pogba Paul | De Bruyne Kevin |
| Captain | Nicaragua | Barrera Juan | Cristiano Ronaldo | Rodríguez James | Robben Arjen |
| Captain | Nigeria | Ahmed Musa | Messi Lionel | Cristiano Ronaldo | Touré Yaya |
| Captain | Northern Ireland | Davis Steven | Messi Lionel | Cristiano Ronaldo | Neymar |
| Captain | Norway | Skjelbred Per Ciljan | Mascherano Javier | Cristiano Ronaldo | Bale Gareth |
| Captain | Palestine | Albahdari Abdel Latif | Messi Lionel | Neymar | Cristiano Ronaldo |
| Captain | Panama | Baloy Felipe | Cristiano Ronaldo | Messi Lionel | Suárez Luis |
| Captain | Paraguay | Da Silva Paulo | Messi Lionel | Cristiano Ronaldo | Suárez Luis |
| Captain | Peru | Pizarro Bossio Claudio | Neuer Manuel | Müller Thomas | Kroos Toni |
| Captain | Philippines | Younghusband Philip James | Neymar | Messi Lionel | Hazard Eden |
| Captain | Poland | Lewandowski Robert | Neuer Manuel | Vidal Arturo | Müller Thomas |
| Captain | Portugal | Ronaldo Cristiano | Benzema Karim | Rodríguez James | Bale Gareth |
| Captain | Puerto Rico | Ramos Héctor | Messi Lionel | Agüero Sergio | Suárez Luis |
| Captain | Qatar | Abdulmajed Ibrahim Majed | Messi Lionel | Cristiano Ronaldo | Neymar |
| Captain | Republic of Ireland | Keane Robbie | Cristiano Ronaldo | Messi Lionel | Neymar |
| Captain | Romania | Rat Razvan | Messi Lionel | Cristiano Ronaldo | Agüero Sergio |
| Captain | Russia | Roman Shirokov | Messi Lionel | Cristiano Ronaldo | De Bruyne Kevin |
| Captain | Samoa | Faaiuaso Desmond | Messi Lionel | Cristiano Ronaldo | Rodríguez James |
| Captain | San Marino | Selva Andy | Cristiano Ronaldo | Neuer Manuel | Messi Lionel |
| Captain | São Tomé e Príncipe | Sousa Pontes Joazhifel | Messi Lionel | Cristiano Ronaldo | Neymar |
| Captain | Scotland | Brown Scott | Lewandowski Robert | Cristiano Ronaldo | Müller Thomas |
| Captain | Senegal | Kouyate Cheikhou | Messi Lionel | Cristiano Ronaldo | Touré Yaya |
| Captain | Serbia | Ivanovic Branislav | Messi Lionel | Hazard Eden | Neymar |
| Captain | Seychelles | Manou Yaninick | Messi Lionel | Cristiano Ronaldo | Neymar |
| Captain | Sierra Leone | Bangura Umaru | Messi Lionel | Lewandowski Robert | Cristiano Ronaldo |
| Captain | Singapore | Ishak Shahril | Cristiano Ronaldo | Messi Lionel | Lewandowski Robert |
| Captain | Slovakia | Skrtel Martin | Cristiano Ronaldo | Suárez Luis | Messi Lionel |
| Captain | Slovenia | Cesar Boštjan | Cristiano Ronaldo | Hazard Eden | Messi Lionel |

| Vote | Country | Name | First (5 points) | Second (3 points) | Third (1 point) |
| --- | --- | --- | --- | --- | --- |
| Captain | Solomon Islands | Faarodo Henry | Neymar | Lewandowski Robert | Messi Lionel |
| Captain | Somalia | Ali Hassan | Messi Lionel | Neuer Manuel | Neymar |
| Captain | South Africa | Mabokgwane Jackson | Messi Lionel | Cristiano Ronaldo | Neymar |
| Captain | Sri Lanka | Roshan Nalaka | Iniesta Andrés | Messi Lionel | Neymar |
| Captain | Sudan | Idriss Saif Eldin | Cristiano Ronaldo | Ibrahimovic Zlatan | Rodríguez James |
| Captain | Swaziland | Tsabedze Thulani | Messi Lionel | Cristiano Ronaldo | Hazard Eden |
| Captain | Sweden | Ibrahimovic´ Zlatan | Messi Lionel | Suárez Luis | Neymar |
| Captain | Switzerland | Inler Gökhan | Cristiano Ronaldo | Messi Lionel | Neymar |
| Captain | Syria | Al Husein Abdulrazak | Messi Lionel | Suárez Luis | Vidal Arturo |
| Captain | Tahiti | Vallar Nicolas | Lewandowski Robert | Messi Lionel | Müller Thomas |
| Captain | Tajikistan | Makhmudov Khurshed | Cristiano Ronaldo | Messi Lionel | Lewandowski Robert |
| Captain | Thailand | Bunmathan Theerathon | Messi Lionel | Cristiano Ronaldo | Neymar |
| Captain | Togo | Akakpo Serge | Messi Lionel | Cristiano Ronaldo | Suárez Luis |
| Captain | Tunisia | Chikhaoui Yassine | Iniesta Andrés | Cristiano Ronaldo | Messi Lionel |
| Captain | Turkey | Turan Arda | Messi Lionel | Neymar | Suárez Luis |
| Captain | Turkmenistan | Atayev Ahmet | Messi Lionel | Cristiano Ronaldo | Neymar |
| Captain | Turks and Caicos Islands | Forbes Billy | Messi Lionel | Sánchez Alexis | Cristiano Ronaldo |
| Captain | Uganda | Massa Geofrey | Cristiano Ronaldo | Messi Lionel | Sánchez Alexis |
| Captain | Ukraine | Tymoshchuk Anatoliy | Messi Lionel | Cristiano Ronaldo | Müller Thomas |
| Captain | United Arab Emirates | Alhosani Ali Khaseif | Cristiano Ronaldo | Agüero Sergio | Touré Yaya |
| Captain | Uruguay | Godín Diego | Suárez Luis | Müller Thomas | Lewandowski Robert |
| Captain | US Virgin Islands | Goods Dusty | Messi Lionel | Müller Thomas | Agüero Sergio |
| Captain | USA | Bradley Michael | Messi Lionel | Cristiano Ronaldo | Suárez Luis |
| Captain | Uzbekistan | Odil Akhmedov | Messi Lionel | Cristiano Ronaldo | Neymar |
| Captain | Vanuatu | Masauvakalo Fenedy | Pogba Paul | Müller Thomas | Sánchez Alexis |
| Captain | Venezuela | Rincon Hernandez Tomas Eduardo | Messi Lionel | Neymar | Müller Thomas |
| Captain | Vietnam | Cong Vinh Le | Messi Lionel | Suárez Luis | Cristiano Ronaldo |
| Captain | Wales | Williams Ashley | Bale Gareth | Messi Lionel | Lewandowski Robert |
| Captain | Zambia | Kalaba Rainford | Neymar | Cristiano Ronaldo | Messi Lionel |
| Captain | Zimbabwe | Phiri Danny | Cristiano Ronaldo | Messi Lionel | Suárez Luis |
| Coach | Afghanistan | Segrt Petar | Neuer Manuel | Cristiano Ronaldo | Suárez Luis |
| Coach | Albania | De Biasi Giovanni | Messi Lionel | Cristiano Ronaldo | Müller Thomas |
| Coach | Algeria | Gourcuff Christian | Messi Lionel | Neymar | Müller Thomas |

| Vote | Country | Name | First (5 points) | Second (3 points) | Third (1 point) |
| --- | --- | --- | --- | --- | --- |
| Coach | American Samoa | Mana'O Larry | Sánchez Alexis | Lewandowski Robert | Neymar |
| Coach | Andorra | Alvarez De Eulate Jesus Luis | Messi Lionel | Neymar | Suárez Luis |
| Coach | Angola | Filemon Romeu | Cristiano Ronaldo | Messi Lionel | Hazard Eden |
| Coach | Antigua and Barbuda | Williams Rolston | Messi Lionel | Cristiano Ronaldo | Suárez Luis |
| Coach | Argentina | Martino Gerardo | Messi Lionel | Agüero Sergio | Mascherano Javier |
| Coach | Armenia | Hovsepyan Sargis | Messi Lionel | Cristiano Ronaldo | Suárez Luis |
| Coach | Aruba | Coolen Rini | Messi Lionel | Cristiano Ronaldo | Müller Thomas |
| Coach | Australia | Postecoglou Ange | Messi Lionel | Cristiano Ronaldo | Neymar |
| Coach | Austria | Koller Marcel | Cristiano Ronaldo | Hazard Eden | De Bruyne Kevin |
| Coach | Azerbaijan | Prosinecki Robert | Messi Lionel | Cristiano Ronaldo | Müller Thomas |
| Coach | Bahamas | Godet Dion | Messi Lionel | Cristiano Ronaldo | Hazard Eden |
| Coach | Bangladesh | Fopez Fabio | Messi Lionel | Cristiano Ronaldo | Lewandowski Robert |
| Coach | Belarus | Khatskevich Aliaksandr | Messi Lionel | Cristiano Ronaldo | Neuer Manuel |
| Coach | Belgium | Wilmots Marc | Neuer Manuel | De Bruyne Kevin | Hazard Eden |
| Coach | Belize | Nunez Jorge | Cristiano Ronaldo | Neymar | Lewandowski Robert |
| Coach | Bermuda | Bascome Andrew | Lewandowski Robert | Müller Thomas | Agüero Sergio |
| Coach | Bhutan | Pema Pema | Cristiano Ronaldo | Müller Thomas | Messi Lionel |
| Coach | Bosnia and Herzegovina | Mehmed Baždarević | Cristiano Ronaldo | Iniesta Andrés | Müller Thomas |
| Coach | Botswana | Butler Peter James | Cristiano Ronaldo | Messi Lionel | Touré Yaya |
| Coach | Brazil | Bledorn Verri Carlos Caetano | Neymar | Messi Lionel | Cristiano Ronaldo |
| Coach | British Virgin Islands | Williams Avondale | Messi Lionel | Cristiano Ronaldo | Iniesta Andrés |
| Coach | Brunei Darussalam | Wong Mun Heng Mike | Bale Gareth | Cristiano Ronaldo | Lewandowski Robert |
| Coach | Bulgaria | Petev Ivaylo | Cristiano Ronaldo | Iniesta Andrés | Müller Thomas |
| Coach | Burkina Faso | Gernot Rhor | Müller Thomas | Messi Lionel | Cristiano Ronaldo |
| Coach | Burundi | Abdel Malek Ait | Messi Lionel | Cristiano Ronaldo | Neymar |
| Coach | Cambodia | Lee Tae Hoon | Cristiano Ronaldo | Ibrahimovic Zlatan | Touré Yaya |
| Coach | Cameroon | Belinga Belinga Alexandre | Cristiano Ronaldo | Messi Lionel | Müller Thomas |
| Coach | Canada | Floro Sanz Benito | Cristiano Ronaldo | Messi Lionel | Rodríguez James |
| Coach | Cape Verde Islands | Aguas Rui | Cristiano Ronaldo | Messi Lionel | Neymar |
| Coach | Cayman Islands | Gonzalez Alexander | Messi Lionel | Cristiano Ronaldo | Vidal Arturo |
| Coach | Chad | Mahamat Oumar Yaya | Messi Lionel | Cristiano Ronaldo | Müller Thomas |
| Coach | Chile | Jorge Sampaoli | Messi Lionel | Sánchez Alexis | Vidal Arturo |
| Coach | China PR | Perrin Alain | Cristiano Ronaldo | Messi Lionel | Müller Thomas |

| Vote | Country | Name | First (5 points) | Second (3 points) | Third (1 point) |
| --- | --- | --- | --- | --- | --- |
| Coach | Colombia | Pekerman Krimen Jose Nestor | Messi Lionel | Rodríguez James | Suárez Luis |
| Coach | Congo DR | Ibenge Florent | Messi Lionel | Cristiano Ronaldo | Touré Yaya |
| Coach | Cook Islands | Sherman Drew | Bale Gareth | Messi Lionel | Cristiano Ronaldo |
| Coach | Costa Rica | Ramirez Oscar | Iniesta Andrés | Müller Thomas | Cristiano Ronaldo |
| Coach | Côte d'Ivoire | Dussuyer Michel | Messi Lionel | Cristiano Ronaldo | Touré Yaya |
| Coach | Croatia | Cacic Ante | Messi Lionel | Cristiano Ronaldo | Ibrahimovic Zlatan |
| Coach | Cuba | Gonzalez Triana Raul | Messi Lionel | Cristiano Ronaldo | Neymar |
| Coach | Curaçao | Kluivert Patrick | Neymar | Suárez Luis | Lewandowski Robert |
| Coach | Cyprus | Christodoulou Charalampos | Cristiano Ronaldo | Messi Lionel | Lewandowski Robert |
| Coach | Czech Republic | Vrba Pavel | Messi Lionel | Cristiano Ronaldo | Lewandowski Robert |
| Coach | Denmark | Olsen Morten | Messi Lionel | Neymar | Suárez Luis |
| Coach | Djibouti | Nourredine Ghrsali | Messi Lionel | Cristiano Ronaldo | Neymar |
| Coach | Dominica | Marshall Shane | Agüero Sergio | Messi Lionel | Cristiano Ronaldo |
| Coach | Dominican Republic | Mojica Juan | Cristiano Ronaldo | Messi Lionel | Iniesta Andrés |
| Coach | Ecuador | Quinteros Gustavo | Messi Lionel | Neuer Manuel | Agüero Sergio |
| Coach | Egypt | Cuper Hector | Messi Lionel | Cristiano Ronaldo | Agüero Sergio |
| Coach | England | Hodgson Roy | Cristiano Ronaldo | Messi Lionel | Hazard Eden |
| Coach | Estonia | Pehrsson Magnus | Messi Lionel | Cristiano Ronaldo | Müller Thomas |
| Coach | Faroe Islands | Olsen Lars | Messi Lionel | Cristiano Ronaldo | Neymar |
| Coach | Fiji | Farina Frank | Cristiano Ronaldo | Messi Lionel | Hazard Eden |
| Coach | Finland | Kanerva Markku | Messi Lionel | Cristiano Ronaldo | Hazard Eden |
| Coach | France | Deschamps Didier | Cristiano Ronaldo | Messi Lionel | Müller Thomas |
| Coach | FYR Macedonia | Angelovski Igor | Messi Lionel | Iniesta Andrés | Cristiano Ronaldo |
| Coach | Georgia | Tskhadadze Kakhaber | Messi Lionel | Cristiano Ronaldo | Lewandowski Robert |
| Coach | Germany | Löw Joachim | Neuer Manuel | Müller Thomas | Kroos Toni |
| Coach | Ghana | Grant Abraham | Neymar | Messi Lionel | Hazard Eden |
| Coach | Guam | White Gary | Messi Lionel | Cristiano Ronaldo | Suárez Luis |
| Coach | Guatemala | Sopegno Ivan Franco | Mascherano Javier | Cristiano Ronaldo | Rodríguez James |
| Coach | Guinea | Fernandez Luis | Messi Lionel | Cristiano Ronaldo | Neuer Manuel |
| Coach | Honduras | Pinto Afanador Jorge Luis | Neymar | Lewandowski Robert | Müller Thomas |
| Coach | Hong Kong | Kim Pangon | Cristiano Ronaldo | Neymar | Sánchez Alexis |
| Coach | Hungary | Storck Bernd | Cristiano Ronaldo | Müller Thomas | Messi Lionel |
| Coach | Iceland | Hallgrimsson Heimir | Cristiano Ronaldo | Suárez Luis | Messi Lionel |

| Vote | Country | Name | First (5 points) | Second (3 points) | Third (1 point) |
| --- | --- | --- | --- | --- | --- |
| Coach | India | Constantine Stephen Phillip | Sánchez Alexis | Neuer Manuel | Agüero Sergio |
| Coach | Iran | Queiroz Carlos | Messi Lionel | Cristiano Ronaldo | Neymar |
| Coach | Israel | Gutman Eli | Cristiano Ronaldo | Bale Gareth | Müller Thomas |
| Coach | Jamaica | Schaefer Winfried | Neuer Manuel | Ibrahimovic Zlatan | Neymar |
| Coach | Japan | Halilhodzic Vahid | Messi Lionel | Cristiano Ronaldo | Neymar |
| Coach | Jordan | Put Paul | Messi Lionel | Cristiano Ronaldo | Neymar |
| Coach | Kazakhstan | Krasnozhan Yuri | Messi Lionel | Cristiano Ronaldo | Neymar |
| Coach | Korea Republic | Stielike Ulrich Otto | Cristiano Ronaldo | Messi Lionel | De Bruyne Kevin |
| Coach | Kyrgyzstan | Krestinin Aleksandr | Lewandowski Robert | Neuer Manuel | Kroos Toni |
| Coach | Laos | Darby Steve | Messi Lionel | Lewandowski Robert | Bale Gareth |
| Coach | Latvia | Pahars Marians | Messi Lionel | Neymar | Robben Arjen |
| Coach | Lebanon | Radulovic Miodrag | Cristiano Ronaldo | Messi Lionel | Rakitic Ivan |
| Coach | Lesotho | Maliehe Moses | Messi Lionel | Neymar | Cristiano Ronaldo |
| Coach | Liberia | Debbah James Salinsa | Cristiano Ronaldo | Benzema Karim | Kroos Toni |
| Coach | Liechtenstein | Pauritsch Rene | Messi Lionel | Lewandowski Robert | Iniesta Andrés |
| Coach | Lithuania | Pankratjevas Igoris | Messi Lionel | Cristiano Ronaldo | Lewandowski Robert |
| Coach | Luxembourg | Holtz Luc | Messi Lionel | Suárez Luis | Cristiano Ronaldo |
| Coach | Macau | Tam Iao San | Messi Lionel | Cristiano Ronaldo | Neymar |
| Coach | Madagascar | Rajaonarisamba Franck | Messi Lionel | Cristiano Ronaldo | Müller Thomas |
| Coach | Malaysia | Kim Swee Ong | Touré Yaya | Cristiano Ronaldo | Messi Lionel |
| Coach | Maldives | Herbert Ricki Lloyd | Lewandowski Robert | Messi Lionel | Müller Thomas |
| Coach | Malta | Ghedin Pietro | Messi Lionel | Cristiano Ronaldo | Bale Gareth |
| Coach | Mauritania | Corentin Martins | Messi Lionel | Cristiano Ronaldo | Neymar |
| Coach | Mauritius | Tshupula Joe | Messi Lionel | Cristiano Ronaldo | Müller Thomas |
| Coach | Mexico | Osorio Arbelaez Juan Carlos | Müller Thomas | Neuer Manuel | Vidal Arturo |
| Coach | Moldova | Stoica Stefan | Messi Lionel | Vidal Arturo | Hazard Eden |
| Coach | Mongolia | Sanjmyatav Purevsukh | Cristiano Ronaldo | Messi Lionel | Robben Arjen |
| Coach | Montenegro | Brnovic Branko | Cristiano Ronaldo | Neymar | Lewandowski Robert |
| Coach | Montserrat | Dublin Geroge | Messi Lionel | Cristiano Ronaldo | Pogba Paul |
| Coach | Morocco | Badou Ezaki | Messi Lionel | Cristiano Ronaldo | Neymar |
| Coach | Mozambique | Muianga Helder Carlos | Messi Lionel | Cristiano Ronaldo | Lewandowski Robert |
| Coach | Myanmar | Zeise Gerd | De Bruyne Kevin | Kroos Toni | Müller Thomas |
| Coach | Namibia | Mannetti Ricardo | Messi Lionel | Cristiano Ronaldo | Neymar |

| Vote | Country | Name | First (5 points) | Second (3 points) | Third (1 point) |
| --- | --- | --- | --- | --- | --- |
| Coach | Nepal | Aussems Patrick | Cristiano Ronaldo | Messi Lionel | Sánchez Alexis |
| Coach | Netherlands | Blind Danny | Messi Lionel | Cristiano Ronaldo | Müller Thomas |
| Coach | New Caledonia | Sardo Thierry | Neymar | Lewandowski Robert | Pogba Paul |
| Coach | New Zealand | Hudson Anthony | Cristiano Ronaldo | Sánchez Alexis | Pogba Paul |
| Coach | Nicaragua | Duarte Molina Henry | Cristiano Ronaldo | Neymar | Robben Arjen |
| Coach | Nigeria | Oliseh Sunday | Messi Lionel | Neymar | Suárez Luis |
| Coach | Northern Ireland | O'Neill Michael | Suárez Luis | Lewandowski Robert | Messi Lionel |
| Coach | Norway | Høgmo Per-Mathias | Cristiano Ronaldo | Messi Lionel | Ibrahimovic Zlatan |
| Coach | Pakistan | Basheer Al Shamlan Mohammad Shamlan  Mubarak | Robben Arjen | Benzema Karim | Cristiano Ronaldo |
| Coach | Palestine | Barkat Abdelnasser | Messi Lionel | Neymar | Cristiano Ronaldo |
| Coach | Panama | Gomez Hernan Dario | Messi Lionel | Neymar | Suárez Luis |
| Coach | Paraguay | Diaz Ramon | Messi Lionel | Rodríguez James | Neymar |
| Coach | Peru | Gareca Ricardo | Messi Lionel | Mascherano Javier | Agüero Sergio |
| Coach | Philippines | Dooley Thomas Dennis | Cristiano Ronaldo | Messi Lionel | Neuer Manuel |
| Coach | Poland | Nawalka Adam | Lewandowski Robert | Messi Lionel | Müller Thomas |
| Coach | Portugal | Santos Fernando | Cristiano Ronaldo | Messi Lionel | Neymar |
| Coach | Puerto Rico | Avedissian Garabet | Messi Lionel | Agüero Sergio | Suárez Luis |
| Coach | Qatar | Carreño José Daniel | Suárez Luis | Messi Lionel | Pogba Paul |
| Coach | Republic of Ireland | O'Neill Martin | Messi Lionel | Hazard Eden | Cristiano Ronaldo |
| Coach | Romania | Iordanescu Anghel | Messi Lionel | Cristiano Ronaldo | Neymar |
| Coach | Russia | Leonid Slutskiy | Messi Lionel | Cristiano Ronaldo | Neuer Manuel |
| Coach | Samoa | Young Phineas | Messi Lionel | Cristiano Ronaldo | Neuer Manuel |
| Coach | San Marino | Manzaroli Pierangelo | Iniesta Andrés | Touré Yaya | Sánchez Alexis |
| Coach | São Tomé e Príncipe | Fernandes Do Rosário António | Messi Lionel | Cristiano Ronaldo | Neymar |
| Coach | Scotland | Strachan Gordon | Cristiano Ronaldo | Müller Thomas | Messi Lionel |
| Coach | Senegal | Cisse Aliou | Touré Yaya | Cristiano Ronaldo | Messi Lionel |
| Coach | Serbia | Curcic Radovan | Messi Lionel | Cristiano Ronaldo | Neymar |
| Coach | Seychelles | Jean Louis Ralph | Messi Lionel | Cristiano Ronaldo | Neymar |
| Coach | Sierra Leone | Teivi Sellas Tetteh | Messi Lionel | Lewandowski Robert | Cristiano Ronaldo |
| Coach | Singapore | Stange Bernd | Messi Lionel | Neuer Manuel | Cristiano Ronaldo |
| Coach | Slovakia | Kozak Jan | Cristiano Ronaldo | Messi Lionel | Pogba Paul |
| Coach | Slovenia | Katanec Srečko | Messi Lionel | Cristiano Ronaldo | Ibrahimovic Zlatan |

| Vote | Country | Name | First (5 points) | Second (3 points) | Third (1 point) |
| --- | --- | --- | --- | --- | --- |
| Coach | Solomon Islands | Toata Moses | Messi Lionel | Cristiano Ronaldo | Neymar |
| Coach | Somalia | Livingstone Charles | Messi Lionel | Neuer Manuel | Neymar |
| Coach | South Africa | Mashaba Ephrahim | Cristiano Ronaldo | Vidal Arturo | Touré Yaya |
| Coach | Spain | Del Bosque González Vicente | Messi Lionel | Cristiano Ronaldo | Iniesta Andrés |
| Coach | Sri Lanka | Perera Sampath | Iniesta Andrés | Messi Lionel | Neymar |
| Coach | Sudan | Hamad Hamdan | Cristiano Ronaldo | Ibrahimovic Zlatan | Rodríguez James |
| Coach | Swaziland | Bulunga Harris | Messi Lionel | Cristiano Ronaldo | Neymar |
| Coach | Sweden | Hamrén Erik | Ibrahimovic Zlatan | Cristiano Ronaldo | Messi Lionel |
| Coach | Switzerland | Petkovic Vladimir | Messi Lionel | Cristiano Ronaldo | Müller Thomas |
| Coach | Syria | Ebrahim Fajer | Messi Lionel | Neymar | Iniesta Andrés |
| Coach | Tahiti | Graugnard Ludovic | Messi Lionel | Lewandowski Robert | Pogba Paul |
| Coach | Tajikistan | Ergashev Mubin | Cristiano Ronaldo | Lewandowski Robert | Messi Lionel |
| Coach | Thailand | Senamuang Kiatisuk | Messi Lionel | Neymar | Cristiano Ronaldo |
| Coach | Togo | Saintfiet Tom | De Bruyne Kevin | Hazard Eden | Touré Yaya |
| Coach | Tunisia | Kasperczak Henri | Lewandowski Robert | Cristiano Ronaldo | Messi Lionel |
| Coach | Turkey | Terim Fatih | Messi Lionel | Cristiano Ronaldo | Robben Arjen |
| Coach | Turkmenistan | Kochumov Amangylych | Messi Lionel | Suárez Luis | Robben Arjen |
| Coach | Turks and Caicos Islands | Smith Oliver | Messi Lionel | Sánchez Alexis | Cristiano Ronaldo |
| Coach | Uganda | Sredojevic Milutin | Messi Lionel | Cristiano Ronaldo | Suárez Luis |
| Coach | Ukraine | Fomenko Mykhailo | Messi Lionel | Cristiano Ronaldo | Suárez Luis |
| Coach | United Arab Emirates | Redha Mahdi Ali | Cristiano Ronaldo | Messi Lionel | Müller Thomas |
| Coach | Uruguay | Tabárez Oscar | Messi Lionel | Suárez Luis | Cristiano Ronaldo |
| Coach | US Virgin Islands | Richards Dale | Messi Lionel | Neymar | Kroos Toni |
| Coach | USA | Klinsmann Juergen | Messi Lionel | Neymar | Cristiano Ronaldo |
| Coach | Uzbekistan | Samvel Babayan | Messi Lionel | Cristiano Ronaldo | Neymar |
| Coach | Vanuatu | Poida Moise | Messi Lionel | Cristiano Ronaldo | Pogba Paul |
| Coach | Venezuela | Sanvicente Bethelmy Noel Argerio | Messi Lionel | Neymar | Müller Thomas |
| Coach | Vietnam | Toshiya Miura | Messi Lionel | Cristiano Ronaldo | Neymar |
| Coach | Wales | Coleman Christopher | Bale Gareth | Cristiano Ronaldo | Hazard Eden |
| Coach | Zambia | Lwandamina George | Neymar | Cristiano Ronaldo | Messi Lionel |
| Coach | Zimbabwe | Pasuwa Kalisto | Messi Lionel | Cristiano Ronaldo | Neymar |
| Media | Albania | Dizdari Besnik | Messi Lionel | Cristiano Ronaldo | Sánchez Alexis |
| Media | Algeria | Ouahib Yazid | Messi Lionel | Cristiano Ronaldo | Neymar |

| Vote | Country | Name | First (5 points) | Second (3 points) | Third (1 point) |
| --- | --- | --- | --- | --- | --- |
| Media | Andorra | Duaso Victor | Messi Lionel | Neymar | Suárez Luis |
| Media | Angola | Goncalves Mateus | Messi Lionel | Cristiano Ronaldo | Pogba Paul |
| Media | Argentina | Sacco Enrique | Messi Lionel | Cristiano Ronaldo | Suárez Luis |
| Media | Armenia | Baghdasarian Souren | Messi Lionel | Cristiano Ronaldo | Neymar |
| Media | Aruba | Kock Ricardo | Messi Lionel | Neymar | Rodríguez James |
| Media | Australia | Foster Craig | Messi Lionel | Neymar | Cristiano Ronaldo |
| Media | Austria | Kowatsch-Schwarz Walter | Messi Lionel | Cristiano Ronaldo | Neymar |
| Media | Azerbaijan | Mövsümov Rasim | Neymar | Suárez Luis | Lewandowski Robert |
| Media | Bahrain | Ashoor Abdullah | Cristiano Ronaldo | Messi Lionel | Neymar |
| Media | Bangladesh | Mahamud Raihan | Messi Lionel | Cristiano Ronaldo | Sánchez Alexis |
| Media | Belarus | Nikolaev Sergey | Messi Lionel | Neymar | Cristiano Ronaldo |
| Media | Belgium | Larsimont Frédéric | Messi Lionel | Cristiano Ronaldo | Suárez Luis |
| Media | Belize | Morales Iglesias Ruben | Messi Lionel | Neymar | Suárez Luis |
| Media | Benin | Sagbo René | Messi Lionel | Cristiano Ronaldo | Suárez Luis |
| Media | Bermuda | Burton James | Messi Lionel | Cristiano Ronaldo | Lewandowski Robert |
| Media | Bolivia | Rivera Carlos Enrique | Suárez Luis | Messi Lionel | Mascherano Javier |
| Media | Bosnia and Herzegovina | Aletic Emir | Messi Lionel | Cristiano Ronaldo | Hazard Eden |
| Media | Botswana | Mathala Oaitse | Cristiano Ronaldo | Messi Lionel | Neymar |
| Media | Brazil | Machado Cleber | Messi Lionel | Cristiano Ronaldo | Neymar |
| Media | Bulgaria | Savov Michel | Messi Lionel | Cristiano Ronaldo | Lewandowski Robert |
| Media | Cambodia | Chamroeun Ung | Messi Lionel | Cristiano Ronaldo | Lewandowski Robert |
| Media | Cameroon | Samnick Gustave | Cristiano Ronaldo | Messi Lionel | Neymar |
| Media | Canada | Davidson Neil | Messi Lionel | Cristiano Ronaldo | Neymar |
| Media | Cape Verde Islands | Amaral André | Messi Lionel | Pogba Paul | Vidal Arturo |
| Media | Cayman Islands | Shillingford Ron | Cristiano Ronaldo | Messi Lionel | Müller Thomas |
| Media | Chile | Diaz Danilo | Messi Lionel | Sánchez Alexis | Lewandowski Robert |
| Media | China PR | Ming Luo | Messi Lionel | Suárez Luis | Cristiano Ronaldo |
| Media | Colombia | Andrade Paché | Messi Lionel | Cristiano Ronaldo | Rodríguez James |
| Media | Congo | Eloue James Golden | Messi Lionel | Cristiano Ronaldo | Neymar |
| Media | Congo DR | Kabelu Eddy | Messi Lionel | Cristiano Ronaldo | Neymar |
| Media | Cook Islands | Kumar Rashneel | Cristiano Ronaldo | Messi Lionel | Suárez Luis |
| Media | Costa Rica | Calvo Castro Rodrigo | Messi Lionel | Neymar | Suárez Luis |
| Media | Côte d'Ivoire | Khalil Adam | Touré Yaya | Messi Lionel | Vidal Arturo |

| Vote | Country | Name | First (5 points) | Second (3 points) | Third (1 point) |
| --- | --- | --- | --- | --- | --- |
| Media | Croatia | Reic Zdravko | Messi Lionel | Cristiano Ronaldo | Neymar |
| Media | Cuba | Hernandez Miguel | Messi Lionel | Cristiano Ronaldo | Iniesta Andrés |
| Media | Curaçao | Dunker Nino | Messi Lionel | Cristiano Ronaldo | Neymar |
| Media | Cyprus | Gavrielides Michel | Messi Lionel | Neymar | Cristiano Ronaldo |
| Media | Czech Republic | Hrabe Stanislav | Messi Lionel | Vidal Arturo | Lewandowski Robert |
| Media | Denmark | Thogersen Troels Bager | Messi Lionel | Suárez Luis | Cristiano Ronaldo |
| Media | Djibouti | Mohamed Kenadid | Messi Lionel | Cristiano Ronaldo | Müller Thomas |
| Media | Dominican Republic | Bauger Jorge Rolando | Messi Lionel | Neymar | Pogba Paul |
| Media | Ecuador | Zavala Garcia Fabricio | Messi Lionel | Cristiano Ronaldo | Sánchez Alexis |
| Media | Egypt | Mazhar Inas | Cristiano Ronaldo | Messi Lionel | Neymar |
| Media | El Salvador | Posada Flores Mario Ernesto | Messi Lionel | Cristiano Ronaldo | Suárez Luis |
| Media | England | Winter Henry | Messi Lionel | Cristiano Ronaldo | Neymar |
| Media | Eritrea | Seium Michael | Cristiano Ronaldo | Sánchez Alexis | Hazard Eden |
| Media | Estonia | Suvari Aet | Messi Lionel | Cristiano Ronaldo | Neymar |
| Media | Ethiopia | Abdulkeni Mensur | Messi Lionel | Neymar | Cristiano Ronaldo |
| Media | Faroe Islands | Arge Trondur | Messi Lionel | Cristiano Ronaldo | Suárez Luis |
| Media | Fiji | Prasad Sanju | Mascherano Javier | Müller Thomas | Kroos Toni |
| Media | Finland | Kanerva Juha | Messi Lionel | Pogba Paul | Cristiano Ronaldo |
| Media | France | Ejnes Gérard | Messi Lionel | Cristiano Ronaldo | Suárez Luis |
| Media | FYR Macedonia | Sotirovski Mario | Messi Lionel | Hazard Eden | Cristiano Ronaldo |
| Media | Gabon | Loundou James Angelo | Messi Lionel | Cristiano Ronaldo | Neymar |
| Media | Gambia | Camara Baboucar | Cristiano Ronaldo | Messi Lionel | Suárez Luis |
| Media | Georgia | Potskhveria Zurab | Cristiano Ronaldo | Messi Lionel | Suárez Luis |
| Media | Germany | Wild Karlheinz | Messi Lionel | Müller Thomas | Cristiano Ronaldo |
| Media | Ghana | Oti Adjei Michael | Messi Lionel | Cristiano Ronaldo | Neymar |
| Media | Greece | Staramopoulos Manos | Messi Lionel | Suárez Luis | Vidal Arturo |
| Media | Grenada | Bascombe Michael | Touré Yaya | Messi Lionel | Agüero Sergio |
| Media | Guatemala | Aguilar Chang Francisco | Messi Lionel | Neymar | Vidal Arturo |
| Media | Guinea | Diallo Ibrahima | Messi Lionel | Cristiano Ronaldo | Neymar |
| Media | Guinea-Bissau | Ucha Etiandro | Messi Lionel | Cristiano Ronaldo | Neymar |
| Media | Guyana | Campbell Emmerson | Messi Lionel | Cristiano Ronaldo | Neymar |
| Media | Haiti | Nere Enock | Messi Lionel | Neymar | Cristiano Ronaldo |
| Media | Honduras | Rivas Francisco Antonio | Messi Lionel | Cristiano Ronaldo | Lewandowski Robert |

| Vote | Country | Name | First (5 points) | Second (3 points) | Third (1 point) |
| --- | --- | --- | --- | --- | --- |
| Media | Hong Kong | Alvarado Gabby | Messi Lionel | Lewandowski Robert | Iniesta Andrés |
| Media | Hungary | Csillag Péter | Messi Lionel | Suárez Luis | Cristiano Ronaldo |
| Media | Iceland | Sigurdsson Vidir | Messi Lionel | Cristiano Ronaldo | Neymar |
| Media | India | Sarkar Dhiman | Cristiano Ronaldo | Lewandowski Robert | Neymar |
| Media | Indonesia | Saleh Nurdin | Messi Lionel | Cristiano Ronaldo | Neymar |
| Media | Iran | Rahmani Siamak | Messi Lionel | Cristiano Ronaldo | Pogba Paul |
| Media | Iraq | Abdul Emam Sami | Messi Lionel | Cristiano Ronaldo | Neymar |
| Media | Israel | Klieger Noah | Messi Lionel | Neymar | Lewandowski Robert |
| Media | Italy | Condo Paolo | Messi Lionel | Lewandowski Robert | Neymar |
| Media | Japan | Tamura Shuichi | Messi Lionel | Neymar | Cristiano Ronaldo |
| Media | Kazakhstan | Tulegenov Geniy | Messi Lionel | Sánchez Alexis | Iniesta Andrés |
| Media | Kenya | Nyende Charles | Suárez Luis | Messi Lionel | Hazard Eden |
| Media | Korea Republic | Won Seok Wee | Messi Lionel | Cristiano Ronaldo | Sánchez Alexis |
| Media | Kuwait | Al Aziz Al Attia Abd | Messi Lionel | Neymar | Cristiano Ronaldo |
| Media | Kyrgyzstan | Louzanov Pavel | Messi Lionel | Suárez Luis | Ibrahimovic Zlatan |
| Media | Latvia | Koscinkevic Ilvars | Messi Lionel | Cristiano Ronaldo | Sánchez Alexis |
| Media | Lebanon | Fawaz Mohamed | Messi Lionel | Neymar | Cristiano Ronaldo |
| Media | Liberia | Carter James Burgess | Cristiano Ronaldo | Messi Lionel | Neymar |
| Media | Libya | Cutajar Joseph | Messi Lionel | Cristiano Ronaldo | Lewandowski Robert |
| Media | Liechtenstein | Hasler Ernst | Messi Lionel | Lewandowski Robert | Cristiano Ronaldo |
| Media | Lithuania | Janonis Giedrius | Messi Lionel | Cristiano Ronaldo | Suárez Luis |
| Media | Luxembourg | Hiegel Didier | Messi Lionel | Cristiano Ronaldo | Lewandowski Robert |
| Media | Macau | Santos Pedro André | Cristiano Ronaldo | Hazard Eden | Messi Lionel |
| Media | Madagascar | Rabary Clément | Cristiano Ronaldo | Messi Lionel | Lewandowski Robert |
| Media | Malawi | Kanjere Peter | Messi Lionel | Cristiano Ronaldo | Sánchez Alexis |
| Media | Malaysia | Hashim Rizal | Messi Lionel | Suárez Luis | Mascherano Javier |
| Media | Maldives | Ali Shimaz | Messi Lionel | Cristiano Ronaldo | Neymar |
| Media | Mali | Bobo Tounkara Souleymane | Messi Lionel | Cristiano Ronaldo | Neymar |
| Media | Malta | Camenzuli Charles | Cristiano Ronaldo | Lewandowski Robert | Messi Lionel |
| Media | Mauritania | Ould El Hacen Mohamed | Messi Lionel | Hazard Eden | Cristiano Ronaldo |
| Media | Mauritius | Hydoo Azmaal | Messi Lionel | Cristiano Ronaldo | Neymar |
| Media | Mexico | Aguilera Salvador | Messi Lionel | Cristiano Ronaldo | Suárez Luis |
| Media | Moldova | Donets Serghei | Messi Lionel | Lewandowski Robert | Cristiano Ronaldo |

| Vote | Country | Name | First (5 points) | Second (3 points) | Third (1 point) |
| --- | --- | --- | --- | --- | --- |
| Media | Mongolia | Tsagaanbaatar Byambaa | Cristiano Ronaldo | Lewandowski Robert | Messi Lionel |
| Media | Montenegro | Mitrovic Danilo | Messi Lionel | Cristiano Ronaldo | Müller Thomas |
| Media | Morocco | Badri Mostafa | Messi Lionel | Cristiano Ronaldo | Pogba Paul |
| Media | Mozambique | Zandamela Alexandre | Messi Lionel | Neymar | Cristiano Ronaldo |
| Media | Namibia | Nikodemus Sheefeni | Messi Lionel | Neymar | Cristiano Ronaldo |
| Media | Nepal | Ghising Raju | Messi Lionel | Cristiano Ronaldo | Vidal Arturo |
| Media | Netherlands | Van Den Nieuwenhof Frans | Cristiano Ronaldo | Suárez Luis | Messi Lionel |
| Media | New Zealand | Watson Gordon Glen | Messi Lionel | Cristiano Ronaldo | Suárez Luis |
| Media | Nicaragua | Rosales Cruz Osman | Messi Lionel | Cristiano Ronaldo | Lewandowski Robert |
| Media | Niger | Ganoua Mohamed Silimane | Messi Lionel | Cristiano Ronaldo | Neymar |
| Media | Nigeria | Audu Samm | Messi Lionel | Cristiano Ronaldo | Neymar |
| Media | Northern Ireland | Fullerton Jackie | Cristiano Ronaldo | Ibrahimovic Zlatan | Lewandowski Robert |
| Media | Norway | Tjaernaas Lars | Messi Lionel | Cristiano Ronaldo | Müller Thomas |
| Media | Oman | Al-Barhi Saleh | Messi Lionel | Cristiano Ronaldo | Neymar |
| Media | Pakistan | Zafar Emad | Messi Lionel | Cristiano Ronaldo | Lewandowski Robert |
| Media | Palestine | Iraqi Mohamad | Cristiano Ronaldo | Messi Lionel | Sánchez Alexis |
| Media | Panama | Estrada Campo Elias | Messi Lionel | Cristiano Ronaldo | Neymar |
| Media | Paraguay | Da Rosa Ruben Dario | Messi Lionel | Cristiano Ronaldo | Neymar |
| Media | Peru | Salinas Carlos | Messi Lionel | Cristiano Ronaldo | Suárez Luis |
| Media | Philippines | Fenix Ryan | Messi Lionel | Cristiano Ronaldo | Neymar |
| Media | Poland | Iwanski Maciej | Lewandowski Robert | Messi Lionel | Cristiano Ronaldo |
| Media | Portugal | Rita Joaquim | Messi Lionel | Cristiano Ronaldo | Neymar |
| Media | Puerto Rico | Arce Luis Santiago | Messi Lionel | Cristiano Ronaldo | Neymar |
| Media | Qatar | Alkhelaifi Majed | Messi Lionel | Cristiano Ronaldo | Neymar |
| Media | Republic of Ireland | Kelly Paul | Messi Lionel | Suárez Luis | Cristiano Ronaldo |
| Media | Romania | Rosu Emmanuel | Messi Lionel | Neymar | Lewandowski Robert |
| Media | Russia | Kleshchev Konstantin | Messi Lionel | Cristiano Ronaldo | Lewandowski Robert |
| Media | Rwanda | Mugabe Bonnie | Messi Lionel | Cristiano Ronaldo | Neymar |
| Media | San Marino | Gorini Elia | Messi Lionel | Cristiano Ronaldo | Neymar |
| Media | Saudi Arabia | Al.Solame Rjallah | Messi Lionel | Cristiano Ronaldo | Neymar |
| Media | Scotland | Greechan John | Messi Lionel | Cristiano Ronaldo | Lewandowski Robert |
| Media | Senegal | Goloko Aliou | Messi Lionel | Cristiano Ronaldo | Neymar |
| Media | Serbia | Novak Vladimir | Messi Lionel | Cristiano Ronaldo | Neymar |

| Vote | Country | Name | First (5 points) | Second (3 points) | Third (1 point) |
| --- | --- | --- | --- | --- | --- |
| Media | Seychelles | Govinden Gérard | Messi Lionel | Cristiano Ronaldo | Suárez Luis |
| Media | Sierra Leone | Fajah Barrie Mohamed | Messi Lionel | Cristiano Ronaldo | Neymar |
| Media | Singapore | Lim Gary | Messi Lionel | Cristiano Ronaldo | Lewandowski Robert |
| Media | Slovakia | Ondrejicka Slavomir | Messi Lionel | Cristiano Ronaldo | Suárez Luis |
| Media | Slovenia | Stare Andrej | Messi Lionel | Cristiano Ronaldo | Lewandowski Robert |
| Media | South Africa | Gleeson Mark | Messi Lionel | Cristiano Ronaldo | Neymar |
| Media | South Sudan | Kayanga John | Messi Lionel | Sánchez Alexis | Cristiano Ronaldo |
| Media | Spain | Relano Alfredo | Messi Lionel | Cristiano Ronaldo | Neymar |
| Media | Sudan | Abu Elgassim Muzamil | Messi Lionel | Cristiano Ronaldo | Touré Yaya |
| Media | Suriname | Romeo Desney | Messi Lionel | Cristiano Ronaldo | Suárez Luis |
| Media | Swaziland | Dlamini Kenneth | Messi Lionel | Neymar | Suárez Luis |
| Media | Sweden | Bengtsson Anders | Cristiano Ronaldo | Lewandowski Robert | Neuer Manuel |
| Media | Switzerland | Cerf Christophe | Messi Lionel | Cristiano Ronaldo | Lewandowski Robert |
| Media | Syria | Abou Shameh Louay | Messi Lionel | Cristiano Ronaldo | Hazard Eden |
| Media | Tahiti | Huc Olivier | Messi Lionel | Cristiano Ronaldo | Neymar |
| Media | Tajikistan | Buriev Alaveddine | Cristiano Ronaldo | Neymar | Messi Lionel |
| Media | Tanzania | Wambura Boniface | Cristiano Ronaldo | Agüero Sergio | Messi Lionel |
| Media | Thailand | Patoommawatana Urai | Messi Lionel | Cristiano Ronaldo | Lewandowski Robert |
| Media | Togo | Ayena Mathias | Cristiano Ronaldo | Messi Lionel | Neymar |
| Media | Trinidad and Tobago | Liburd Lasana | Messi Lionel | Neymar | Pogba Paul |
| Media | Tunisia | Dhaifallah Abdesslam | Messi Lionel | Neymar | Cristiano Ronaldo |
| Media | Turkey | Manav Selçuk | Messi Lionel | Cristiano Ronaldo | Neymar |
| Media | Turkmenistan | Vershinin Alexander | Messi Lionel | Neymar | Suárez Luis |
| Media | Turks and Caicos Islands | Deo Faizool | Neymar | Messi Lionel | Lewandowski Robert |
| Media | Uganda | Musisi Kiyingi Fredrick | Messi Lionel | Cristiano Ronaldo | Neymar |
| Media | Ukraine | Linnyk Igor | Neymar | Cristiano Ronaldo | Lewandowski Robert |
| Media | United Arab Emirates | Mouadhen Dafrallah | Messi Lionel | Neymar | Lewandowski Robert |
| Media | Uruguay | Pineyrua Ricardo | Suárez Luis | Lewandowski Robert | Neuer Manuel |
| Media | USA | Kennedy Paul | Messi Lionel | Cristiano Ronaldo | Neymar |
| Media | Uzbekistan | Rtveladze Grigoriy | Cristiano Ronaldo | Suárez Luis | Müller Thomas |
| Media | Vanuatu | Nase Raymond | Lewandowski Robert | Cristiano Ronaldo | Messi Lionel |
| Media | Venezuela | Blavia Francisco | Messi Lionel | Cristiano Ronaldo | Lewandowski Robert |
| Media | Vietnam | Anh Ngoc Truong | Messi Lionel | Cristiano Ronaldo | Neymar |

| Vote | Country | Name | First (5 points) | Second (3 points) | Third (1 point) |
| --- | --- | --- | --- | --- | --- |
| Media | Wales | Abbandonato Paul | Messi Lionel | Lewandowski Robert | Bale Gareth |
| Media | Yemen | Al Hababi Adel | Messi Lionel | Cristiano Ronaldo | Ibrahimovic Zlatan |
| Media | Zambia | Lungu Chapadongo | Messi Lionel | Cristiano Ronaldo | Ibrahimovic Zlatan |
| Media | Zimbabwe | Mabika Charles | Messi Lionel | Cristiano Ronaldo | Neymar |

| Vote | Country | Name | First (5 points) | Second (3 points) | Third (1 point) |
| --- | --- | --- | --- | --- | --- |
| Captain | Albania | Agolli Ansi | Cristiano Ronaldo | Messi Lionel | Griezmann Antoine |
| Captain | American Samoa | Ott Ramin | Neymar | Cristiano Ronaldo | Messi Lionel |
| Captain | Andorra | Lima Ildefons | Messi Lionel | Neymar | Cristiano Ronaldo |
| Captain | Angola | Cardoso Dario | Cristiano Ronaldo | Messi Lionel | Sánchez Alexis |
| Captain | Antigua and Barbuda | Joshua Parker | Iniesta Andrés | Cristiano Ronaldo | Neymar |
| Captain | Argentina | Messi Lionel | Suárez Luis | Neymar | Iniesta Andrés |
| Captain | Armenia | Mkhitaryan Henrikh | Cristiano Ronaldo | Messi Lionel | Griezmann Antoine |
| Captain | Aruba | Abdul David | Messi Lionel | Cristiano Ronaldo | Griezmann Antoine |
| Captain | Australia | Jedinak Mile | Messi Lionel | Cristiano Ronaldo | Bale Gareth |
| Captain | Austria | Baumgartlinger Julian | Kroos Toni | Cristiano Ronaldo | Messi Lionel |
| Captain | Azerbaijan | Sadigov Rashad | Cristiano Ronaldo | Messi Lionel | Griezmann Antoine |
| Captain | Bahamas | St. Fleur Lesly | Messi Lionel | Neymar | Lewandowski Robert |
| Captain | Bahrain | Mahfoodh Mohamed | Cristiano Ronaldo | Bale Gareth | Messi Lionel |
| Captain | Belgium | Hazard Eden | Cristiano Ronaldo | Messi Lionel | Griezmann Antoine |
| Captain | Bermuda | Lambe Reginald | Messi Lionel | Suárez Luis | Özil Mesut |
| Captain | Bosnia and Herzegovina | Džeko Edin | Cristiano Ronaldo | Messi Lionel | Suárez Luis |
| Captain | Botswana | Mogorosi Joel | Cristiano Ronaldo | Neymar | Mahrez Riyad |
| Captain | Brazil | Alves Da Silva Daniel | Messi Lionel | Neymar | Suárez Luis |
| Captain | British Virgin Islands | Ceasar Troy | Cristiano Ronaldo | Messi Lionel | Neymar |
| Captain | Bulgaria | Dyakov Svetoslav | Buffon Gianluigi | Cristiano Ronaldo | Messi Lionel |
| Captain | Burundi | Duhayindavyi Gael | Cristiano Ronaldo | Griezmann Antoine | Messi Lionel |
| Captain | Cambodia | Um Sereirath | Cristiano Ronaldo | Messi Lionel | Neymar |
| Captain | Cameroon | Moukandjo Benjamin | Messi Lionel | Cristiano Ronaldo | Neymar |
| Captain | Canada | De Guzman Julian | Cristiano Ronaldo | Messi Lionel | Suárez Luis |
| Captain | Cape Verde Islands | Soares Marco | Cristiano Ronaldo | Messi Lionel | Neymar |
| Captain | Chad | Doumnan Herman | Cristiano Ronaldo | Griezmann Antoine | Mahrez Riyad |
| Captain | China PR | Zheng Zhi | Messi Lionel | Cristiano Ronaldo | Buffon Gianluigi |
| Captain | Chinese Taipei | Chen Yi-Wei | Messi Lionel | Buffon Gianluigi | Ibrahimovic Zlatan |
| Captain | Colombia | Rodriguez James | Cristiano Ronaldo | Modric Luka | Bale Gareth |
| Captain | Congo | Oniangue Prince | Cristiano Ronaldo | Neymar | Griezmann Antoine |
| Captain | Cook Islands | Jake Stuart | Cristiano Ronaldo | Messi Lionel | Suárez Luis |
| Captain | Costa Rica | Ruiz Bryan | Messi Lionel | Cristiano Ronaldo | Suárez Luis |
| Captain | Côte d'Ivoire | Yao Kouassi Gervais | Cristiano Ronaldo | Griezmann Antoine | Mahrez Riyad |

| Vote | Country | Name | First (5 points) | Second (3 points) | Third (1 point) |
| --- | --- | --- | --- | --- | --- |
| Captain | Croatia | Modric Luka | Cristiano Ronaldo | Messi Lionel | Bale Gareth |
| Captain | Cuba | Reyes Azcuy Maykel | Cristiano Ronaldo | Messi Lionel | Neymar |
| Captain | Curaçao | Martina Rhu-Endly | Messi Lionel | Lewandowski Robert | Özil Mesut |
| Captain | Cyprus | Charalambides Constantinos | Cristiano Ronaldo | Messi Lionel | Griezmann Antoine |
| Captain | Czech Republic | Sivok Tomáš | Cristiano Ronaldo | Messi Lionel | Suárez Luis |
| Captain | Denmark | Kjær Simon | Cristiano Ronaldo | Messi Lionel | Griezmann Antoine |
| Captain | Djibouti | Mohamed Kadar Ahmed | Messi Lionel | Cristiano Ronaldo | Neymar |
| Captain | Dominica | Prince Glenson | Cristiano Ronaldo | Messi Lionel | Bale Gareth |
| Captain | Dominican Republic | Faña Jonathan | Cristiano Ronaldo | Messi Lionel | Suárez Luis |
| Captain | Egypt | El Hadary Essam | Messi Lionel | Cristiano Ronaldo | Iniesta Andrés |
| Captain | England | Rooney Wayne | Cristiano Ronaldo | Suárez Luis | Vardy Jamie |
| Captain | Estonia | Klavan Ragnar | Messi Lionel | Cristiano Ronaldo | Griezmann Antoine |
| Captain | Faroe Islands | Gregersen Atli | Cristiano Ronaldo | Messi Lionel | Bale Gareth |
| Captain | Finland | Moisander Niklas | Messi Lionel | Cristiano Ronaldo | Suárez Luis |
| Captain | France | Lloris Hugo | Griezmann Antoine | Messi Lionel | Cristiano Ronaldo |
| Captain | FYR Macedonia | Pandev Goran | Cristiano Ronaldo | Messi Lionel | Griezmann Antoine |
| Captain | Gabon | Aubameyang Pierre-Emerick | Neymar | Cristiano Ronaldo | Messi Lionel |
| Captain | Gambia | Colley Omar | Cristiano Ronaldo | Neymar | Messi Lionel |
| Captain | Georgia | Kankava Jaba | Griezmann Antoine | Messi Lionel | Cristiano Ronaldo |
| Captain | Germany | Neuer Manuel | Kroos Toni | Özil Mesut | Lewandowski Robert |
| Captain | Ghana | Asamoah Gyan | Cristiano Ronaldo | Messi Lionel | Neymar |
| Captain | Gibraltar | Chipolina Roy | Cristiano Ronaldo | Messi Lionel | Suárez Luis |
| Captain | Greece | Torosidis Vasileios | Messi Lionel | Cristiano Ronaldo | Griezmann Antoine |
| Captain | Grenada | Rennie Jake | Bale Gareth | Cristiano Ronaldo | Neymar |
| Captain | Guam | Cunliffe Jason | Cristiano Ronaldo | Messi Lionel | Neymar |
| Captain | Guinea | Camara Fode | Cristiano Ronaldo | Messi Lionel | Griezmann Antoine |
| Captain | Guinea-Bissau | Ca Bocundji | Cristiano Ronaldo | Messi Lionel | Griezmann Antoine |
| Captain | Honduras | Figueroa Maynor | Cristiano Ronaldo | Griezmann Antoine | Suárez Luis |
| Captain | Hong Kong | Yapp Hung Fai | Cristiano Ronaldo | Messi Lionel | Griezmann Antoine |
| Captain | Hungary | Dzsudzsák Balázs | Cristiano Ronaldo | Messi Lionel | Neymar |
| Captain | Iceland | Gunnarsson Aron Einar | Cristiano Ronaldo | Suárez Luis | Griezmann Antoine |
| Captain | India | Chhetri Sunil | Cristiano Ronaldo | Messi Lionel | Griezmann Antoine |
| Captain | Iran | Hosseini Seyed Jalal | Cristiano Ronaldo | Messi Lionel | Neymar |

| Vote | Country | Name | First (5 points) | Second (3 points) | Third (1 point) |
| --- | --- | --- | --- | --- | --- |
| Captain | Israel | Zahavi Eran | Cristiano Ronaldo | Messi Lionel | Suárez Luis |
| Captain | Italy | Buffon Gianluigi | Messi Lionel | Bale Gareth | Cristiano Ronaldo |
| Captain | Jamaica | Blake Andre | Messi Lionel | Cristiano Ronaldo | Suárez Luis |
| Captain | Japan | Hasebe Makoto | Suárez Luis | Griezmann Antoine | Messi Lionel |
| Captain | Jordan | Shafi Amer | Buffon Gianluigi | Messi Lionel | Cristiano Ronaldo |
| Captain | Kazakhstan | Islamkhan Baurzhan | Iniesta Andrés | Messi Lionel | Cristiano Ronaldo |
| Captain | Kenya | Mugabe Victor | Cristiano Ronaldo | Messi Lionel | Griezmann Antoine |
| Captain | Korea DPR | Ri Myong Guk | Messi Lionel | Cristiano Ronaldo | Neymar |
| Captain | Korea Republic | Sungyueng Ki | Messi Lionel | Cristiano Ronaldo | Griezmann Antoine |
| Captain | Kosovo | Ujkani Samir | Cristiano Ronaldo | Neymar | Messi Lionel |
| Captain | Kyrgyzstan | Shamshiev Islam | Cristiano Ronaldo | Messi Lionel | Suárez Luis |
| Captain | Laos | Souliyavong Paseuthsack | Cristiano Ronaldo | Neymar | Messi Lionel |
| Captain | Latvia | Gorkšs Kaspars | Cristiano Ronaldo | Griezmann Antoine | Buffon Gianluigi |
| Captain | Lebanon | Maatouk Hassan | Cristiano Ronaldo | Messi Lionel | Lewandowski Robert |
| Captain | Liberia | Dennis Teah | Cristiano Ronaldo | Ramos Sergio | Messi Lionel |
| Captain | Liechtenstein | Jehle Peter | Cristiano Ronaldo | Messi Lionel | Griezmann Antoine |
| Captain | Lithuania | Cernych Fedor | Cristiano Ronaldo | Griezmann Antoine | Messi Lionel |
| Captain | Luxembourg | Mutsch Mario | Cristiano Ronaldo | Kroos Toni | Griezmann Antoine |
| Captain | Macau | Cheang Cheng Ieong Paulo | Neymar | Cristiano Ronaldo | Kroos Toni |
| Captain | Madagascar | Andriatsima Faneva | Cristiano Ronaldo | Suárez Luis | Messi Lionel |
| Captain | Malaysia | Yahyah Mohd Amri | Cristiano Ronaldo | Özil Mesut | Vardy Jamie |
| Captain | Maldives | Ashfaq Ali | Cristiano Ronaldo | Messi Lionel | Suárez Luis |
| Captain | Malta | Mifsud Michael | Iniesta Andrés | Neymar | Sánchez Alexis |
| Captain | Mauritania | Guidileye Dialo | Cristiano Ronaldo | Messi Lionel | Neymar |
| Captain | Mauritius | Langue Jean Anderson Adel Bruano | Messi Lionel | Kanté N'Golo | Cristiano Ronaldo |
| Captain | Mexico | Marquez Rafael | Messi Lionel | Griezmann Antoine | Suárez Luis |
| Captain | Moldova | Epureanu Alexandru | Cristiano Ronaldo | Messi Lionel | Griezmann Antoine |
| Captain | Mongolia | Daginaa Turbat | Suárez Luis | Pogba Paul | Cristiano Ronaldo |
| Captain | Montenegro | Jovetic Stevan | Messi Lionel | Neymar | Cristiano Ronaldo |
| Captain | Montserrat | Griffith Anthony | Suárez Luis | Bale Gareth | Griezmann Antoine |
| Captain | Morocco | Benatia Mehdi | Buffon Gianluigi | Cristiano Ronaldo | Neymar |
| Captain | Mozambique | Pelembe Elias | Cristiano Ronaldo | Messi Lionel | Agüero Sergio |
| Captain | Myanmar | Kyaw Yan Aung | Cristiano Ronaldo | Messi Lionel | Griezmann Antoine |

| Vote | Country | Name | First (5 points) | Second (3 points) | Third (1 point) |
| --- | --- | --- | --- | --- | --- |
| Captain | Namibia | Ketjijere Ronald | Messi Lionel | Cristiano Ronaldo | Iniesta Andrés |
| Captain | Nepal | Gurung Anil | Cristiano Ronaldo | Neymar | Messi Lionel |
| Captain | Netherlands | Robben Arjen | Cristiano Ronaldo | Kroos Toni | Lewandowski Robert |
| Captain | New Caledonia | Kai Bertrand | Messi Lionel | Neymar | Lewandowski Robert |
| Captain | New Zealand | Wood Chris | De Bruyne Kevin | Messi Lionel | Griezmann Antoine |
| Captain | Nigeria | Mikel John Obi | Cristiano Ronaldo | Griezmann Antoine | Neuer Manuel |
| Captain | Northern Ireland | Davis Steven | Messi Lionel | Cristiano Ronaldo | Griezmann Antoine |
| Captain | Norway | Skjelbred Per Ciljan | Messi Lionel | Cristiano Ronaldo | Ibrahimovic Zlatan |
| Captain | Oman | Muqbali Abdulaziz | Messi Lionel | Cristiano Ronaldo | Bale Gareth |
| Captain | Pakistan | Hussain Saddam | Cristiano Ronaldo | Messi Lionel | Bale Gareth |
| Captain | Palestine | Bahdari Abdellatef | Mahrez Riyad | Cristiano Ronaldo | Messi Lionel |
| Captain | Panama | Torres Roman | Messi Lionel | Cristiano Ronaldo | Neymar |
| Captain | Papua New Guinea | Muta David | Messi Lionel | Suárez Luis | Iniesta Andrés |
| Captain | Paraguay | Da Silva Barrios Paulo | Cristiano Ronaldo | Neymar | Messi Lionel |
| Captain | Peru | Guerrero Gonzales Jose Paolo | Messi Lionel | Cristiano Ronaldo | De Bruyne Kevin |
| Captain | Philippines | Younghusband Phil | Messi Lionel | Neymar | Suárez Luis |
| Captain | Poland | Lewandowski Robert | Neuer Manuel | Bale Gareth | Cristiano Ronaldo |
| Captain | Portugal | Ronaldo Cristiano | Bale Gareth | Modric Luka | Ramos Sergio |
| Captain | Puerto Rico | Cabrero Gómez Andrés Nicolás | Ramos Sergio | Griezmann Antoine | Vardy Jamie |
| Captain | Qatar | Al Haydos Hasan Kahlid | Messi Lionel | Bale Gareth | Griezmann Antoine |
| Captain | Republic of Ireland | Coleman Seamus | Cristiano Ronaldo | Messi Lionel | Suárez Luis |
| Captain | Romania | Dragos Grigore | Messi Lionel | Cristiano Ronaldo | Ramos Sergio |
| Captain | Russia | Akinfeev Igor | Messi Lionel | Cristiano Ronaldo | Bale Gareth |
| Captain | Rwanda | Haruna Niyonzima | Cristiano Ronaldo | Messi Lionel | Sánchez Alexis |
| Captain | Samoa | Desmond Faaiuaso | Messi Lionel | Neymar | Cristiano Ronaldo |
| Captain | San Marino | Matteo Vitaioli | Cristiano Ronaldo | Buffon Gianluigi | Messi Lionel |
| Captain | São Tomé e Príncipe | Sousa Pontes Joazhifel | Cristiano Ronaldo | Messi Lionel | Neymar |
| Captain | Saudi Arabia | Hawsawi Osama | Cristiano Ronaldo | Neymar | Messi Lionel |
| Captain | Scotland | Fletcher Darren | Cristiano Ronaldo | Suárez Luis | Messi Lionel |
| Captain | Senegal | Kouyate Cheikhou | Cristiano Ronaldo | Messi Lionel | Griezmann Antoine |
| Captain | Serbia | Branislav Ivanovic | Cristiano Ronaldo | Messi Lionel | Neymar |
| Captain | Singapore | Ishak Shahril | Cristiano Ronaldo | Messi Lionel | Özil Mesut |
| Captain | Slovakia | Skrtel Martin | Suárez Luis | Ramos Sergio | Cristiano Ronaldo |

| Vote | Country | Name | First (5 points) | Second (3 points) | Third (1 point) |
| --- | --- | --- | --- | --- | --- |
| Captain | Slovenia | Cesar Boštjan | Cristiano Ronaldo | Griezmann Antoine | Ramos Sergio |
| Captain | Somalia | Ali Hassan | Buffon Gianluigi | Iniesta Andrés | Neuer Manuel |
| Captain | Spain | Ramos García Sergio | Cristiano Ronaldo | Messi Lionel | Iniesta Andrés |
| Captain | Sri Lanka | Perera Sujan | Messi Lionel | Cristiano Ronaldo | Neymar |
| Captain | St. Kitts and Nevis | Harris Atiba | Messi Lionel | Pogba Paul | Cristiano Ronaldo |
| Captain | Sudan | Salim Akram Alhadi | Cristiano Ronaldo | Messi Lionel | Griezmann Antoine |
| Captain | Suriname | Eind Gilberto | Cristiano Ronaldo | Messi Lionel | Griezmann Antoine |
| Captain | Sweden | Granqvist Andreas | Ibrahimovic Zlatan | Cristiano Ronaldo | Bale Gareth |
| Captain | Switzerland | Lichtsteiner Stephan | Cristiano Ronaldo | Messi Lionel | Ibrahimovic Zlatan |
| Captain | Syria | Al Saleh Ahmad | Messi Lionel | Suárez Luis | Cristiano Ronaldo |
| Captain | Tahiti | Tehau Alvin | Messi Lionel | Neymar | Cristiano Ronaldo |
| Captain | Tajikistan | Davronov Nuriddin | Cristiano Ronaldo | Ibrahimovic Zlatan | Kroos Toni |
| Captain | Tanzania | Samatta Mbwana Ally | Messi Lionel | Cristiano Ronaldo | Neymar |
| Captain | Thailand | Bunmathan Theerathon | Cristiano Ronaldo | Messi Lionel | Suárez Luis |
| Captain | Togo | Adebayor Sheyi | Cristiano Ronaldo | Mahrez Riyad | Messi Lionel |
| Captain | Turkey | Turan Arda | Messi Lionel | Neymar | Iniesta Andrés |
| Captain | Turkmenistan | Amanov Arslanmyrat | Messi Lionel | Cristiano Ronaldo | Pogba Paul |
| Captain | Turks and Caicos Islands | Forbes Billy | Vardy Jamie | Cristiano Ronaldo | Messi Lionel |
| Captain | Uganda | Massa Geofrey | Cristiano Ronaldo | Messi Lionel | Neuer Manuel |
| Captain | Ukraine | Kucher Olexandr | Cristiano Ronaldo | Messi Lionel | Buffon Gianluigi |
| Captain | Uruguay | Diego Godin | Suárez Luis | Griezmann Antoine | Iniesta Andrés |
| Captain | USA | Bradley Michael | Cristiano Ronaldo | Messi Lionel | Griezmann Antoine |
| Captain | Uzbekistan | Akhmedov Odil | Messi Lionel | Cristiano Ronaldo | Griezmann Antoine |
| Captain | Venezuela | Rincon Tomas | Cristiano Ronaldo | Messi Lionel | Neymar |
| Captain | Vietnam | Le Cong Vinh | Cristiano Ronaldo | Messi Lionel | Bale Gareth |
| Captain | Wales | Williams Ashley | Bale Gareth | Messi Lionel | Lewandowski Robert |
| Captain | Zambia | Kalaba Rainford | Cristiano Ronaldo | Messi Lionel | Neymar |
| Coach | Albania | De Biasi Giovanni | Buffon Gianluigi | Cristiano Ronaldo | Messi Lionel |
| Coach | American Samoa | Mana'O Larry | Sánchez Alexis | Lewandowski Robert | Neymar |
| Coach | Andorra | Alvarez De Eulate Jesus Luís | Messi Lionel | Griezmann Antoine | Bale Gareth |
| Coach | Angola | Kilamba José | Messi Lionel | Cristiano Ronaldo | Bale Gareth |
| Coach | Antigua and Barbuda | Williams Rolston | Cristiano Ronaldo | Neymar | Pogba Paul |
| Coach | Argentina | Bauza Edgardo | Messi Lionel | Agüero Sergio | Griezmann Antoine |

| Vote | Country | Name | First (5 points) | Second (3 points) | Third (1 point) |
| --- | --- | --- | --- | --- | --- |
| Coach | Armenia | Petrosyan Artur | Neymar | Cristiano Ronaldo | Messi Lionel |
| Coach | Aruba | Coolen Rini | Messi Lionel | Cristiano Ronaldo | Lewandowski Robert |
| Coach | Australia | Postecoglou Ange | Cristiano Ronaldo | Griezmann Antoine | Sánchez Alexis |
| Coach | Austria | Koller Marcel | Cristiano Ronaldo | Bale Gareth | Griezmann Antoine |
| Coach | Azerbaijan | Prosinecki Robert | Messi Lionel | Cristiano Ronaldo | Griezmann Antoine |
| Coach | Bahamas | Godet Dion | Messi Lionel | Cristiano Ronaldo | Vardy Jamie |
| Coach | Bahrain | Soukup Miroslav | Messi Lionel | Cristiano Ronaldo | Griezmann Antoine |
| Coach | Belgium | Martinez Roberto | Cristiano Ronaldo | Messi Lionel | Neymar |
| Coach | Bermuda | Bascome Andrew | Neymar | Cristiano Ronaldo | Suárez Luis |
| Coach | Bosnia and Herzegovina | Baždarevic Mehmed | Cristiano Ronaldo | Griezmann Antoine | Messi Lionel |
| Coach | Botswana | Butler Peter | Cristiano Ronaldo | Messi Lionel | Vardy Jamie |
| Coach | Brazil | Bachi Adenor Leonardo | Cristiano Ronaldo | Neymar | Griezmann Antoine |
| Coach | British Virgin Islands | Williams Avondale | Messi Lionel |  | Pogba Paul |
| Coach | Bulgaria | Houbtchev Petar | Messi Lionel | Cristiano Ronaldo | Griezmann Antoine |
| Coach | Burundi | Niyungeko Alain Olivier | Messi Lionel | Cristiano Ronaldo | Neymar |
| Coach | Cambodia | Lee Taehoon | Messi Lionel | Lewandowski Robert | Sánchez Alexis |
| Coach | Cameroon | Hugo Broos | Messi Lionel | Griezmann Antoine | Cristiano Ronaldo |
| Coach | Canada | Findlay Michael | Cristiano Ronaldo | Messi Lionel | Lewandowski Robert |
| Coach | Chad | Mahamat Oumar Yaya | Cristiano Ronaldo | Messi Lionel | Neymar |
| Coach | Chile | Pizzi Juan Antonio | Sánchez Alexis |  |  |
| Coach | China PR | Lippi Marcello | Cristiano Ronaldo | Griezmann Antoine | Buffon Gianluigi |
| Coach | Chinese Taipei | Kuroda Kazuo | Vardy Jamie | Kanté N'Golo | Cristiano Ronaldo |
| Coach | Colombia | Pekerman José | Messi Lionel | Suárez Luis | Sánchez Alexis |
| Coach | Congo | Tchangana Gaston (Dtn) | Cristiano Ronaldo | Neymar | Griezmann Antoine |
| Coach | Cook Islands | Brom Jess | Cristiano Ronaldo | Messi Lionel | Ibrahimovic Zlatan |
| Coach | Costa Rica | Ramirez Oscar | Iniesta Andrés | Modric Luka | Sánchez Alexis |
| Coach | Côte d'Ivoire | Dussuyer Michel | Cristiano Ronaldo | Messi Lionel | Griezmann Antoine |
| Coach | Croatia | Cacic Ante | Cristiano Ronaldo | Messi Lionel | Modric Luka |
| Coach | Cuba | Mederos Sosa Raul | Messi Lionel | Cristiano Ronaldo | Neymar |
| Coach | Curaçao | Bicentini Remko | Messi Lionel | Griezmann Antoine | Suárez Luis |
| Coach | Cyprus | Christoforou Christakis | Cristiano Ronaldo | Bale Gareth | Buffon Gianluigi |
| Coach | Czech Republic | Jarolím Karel | Messi Lionel | Cristiano Ronaldo | Bale Gareth |
| Coach | Denmark | Hareide Åge | Messi Lionel | Cristiano Ronaldo | Neymar |

| Vote | Country | Name | First (5 points) | Second (3 points) | Third (1 point) |
| --- | --- | --- | --- | --- | --- |
| Coach | Djibouti | Ali Affasseh Abdourahman | Messi Lionel | Cristiano Ronaldo | Neymar |
| Coach | Dominica | Sabin Ellington | Cristiano Ronaldo | Messi Lionel | Bale Gareth |
| Coach | Dominican Republic | Díaz Bernabé Roberto | Messi Lionel | Iniesta Andrés | Griezmann Antoine |
| Coach | Egypt | Cúper Héctor | Messi Lionel | Cristiano Ronaldo | Iniesta Andrés |
| Coach | El Salvador | Lara Eduardo | Cristiano Ronaldo | Messi Lionel | Kroos Toni |
| Coach | England | Southgate Gareth | Cristiano Ronaldo | Griezmann Antoine | Kanté N'Golo |
| Coach | Estonia | Reim Martin | Cristiano Ronaldo | Messi Lionel | Griezmann Antoine |
| Coach | Faroe Islands | Olsen Lars | Cristiano Ronaldo | Messi Lionel | Suárez Luis |
| Coach | Fiji | Dutt Yogendra | Cristiano Ronaldo | Neymar | Vardy Jamie |
| Coach | Finland | Backe Hans | Cristiano Ronaldo | Messi Lionel | Bale Gareth |
| Coach | France | Deschamps Didier | Griezmann Antoine | Cristiano Ronaldo | Messi Lionel |
| Coach | FYR Macedonia | Angelovski Igor | Cristiano Ronaldo | Messi Lionel | Griezmann Antoine |
| Coach | Gambia | Ndong Sang | Cristiano Ronaldo | Mahrez Riyad | Messi Lionel |
| Coach | Georgia | Weiss Vladimír | Messi Lionel | Cristiano Ronaldo | Bale Gareth |
| Coach | Germany | Löw Joachim | Kroos Toni | Özil Mesut | Neuer Manuel |
| Coach | Ghana | Grant Abraham | Neymar | Messi Lionel | Cristiano Ronaldo |
| Coach | Gibraltar | Wood Jeff | Griezmann Antoine | Cristiano Ronaldo | Bale Gareth |
| Coach | Greece | Skibbe Michael | Cristiano Ronaldo | Griezmann Antoine | Kroos Toni |
| Coach | Grenada | N\|A N\|A | Griezmann Antoine | Cristiano Ronaldo | Kanté N'Golo |
| Coach | Guam | Darren Sawatzky | Messi Lionel | Cristiano Ronaldo | Ibrahimovic Zlatan |
| Coach | Guinea | Bangoura Mohamed Kanfory | Cristiano Ronaldo | Griezmann Antoine | Messi Lionel |
| Coach | Guinea-Bissau | Cande Baciro | Cristiano Ronaldo | Messi Lionel | Griezmann Antoine |
| Coach | Haiti | Neveu Patrice | Cristiano Ronaldo | Neymar | Neuer Manuel |
| Coach | Honduras | Pinto Afanador Jorge Luis | Suárez Luis | Messi Lionel | Modric Luka |
| Coach | Hong Kong | Kim Pangon | Cristiano Ronaldo | Messi Lionel | Bale Gareth |
| Coach | Hungary | Storck Bernd | Cristiano Ronaldo | Kroos Toni | Messi Lionel |
| Coach | Iceland | Hallgrimsson Heimir | Suárez Luis | Cristiano Ronaldo | Griezmann Antoine |
| Coach | India | Stephen Phillip Constantine | Bale Gareth | Lewandowski Robert | Suárez Luis |
| Coach | Iran | Queiroz Carlos | Messi Lionel | Cristiano Ronaldo | Suárez Luis |
| Coach | Israel | Levi Elisha | Cristiano Ronaldo | Griezmann Antoine | Messi Lionel |
| Coach | Italy | Ventura Giampiero | Buffon Gianluigi | Neuer Manuel | Cristiano Ronaldo |
| Coach | Jamaica | Whitmore Theodore | Messi Lionel | Neymar | Vardy Jamie |
| Coach | Japan | Halilhodzic Vahid | Cristiano Ronaldo | Messi Lionel | Mahrez Riyad |

| Vote | Country | Name | First (5 points) | Second (3 points) | Third (1 point) |
| --- | --- | --- | --- | --- | --- |
| Coach | Jordan | Gelling Stuart | Suárez Luis | De Bruyne Kevin | Vardy Jamie |
| Coach | Kazakhstan | Baisufinov Talgat | Messi Lionel | Cristiano Ronaldo | Buffon Gianluigi |
| Coach | Kenya | Okumbi Stanley | Messi Lionel | Cristiano Ronaldo | Griezmann Antoine |
| Coach | Korea DPR | Andersen Joern | Messi Lionel | Cristiano Ronaldo | Suárez Luis |
| Coach | Korea Republic | Ulrich Otto Stielike | Bale Gareth | Griezmann Antoine | Cristiano Ronaldo |
| Coach | Kosovo | Bunjaki Albert | Cristiano Ronaldo | Messi Lionel | Griezmann Antoine |
| Coach | Kyrgyzstan | Krestinin Alexander | Özil Mesut | Cristiano Ronaldo | Messi Lionel |
| Coach | Laos | Phomphakdy Valakone | Griezmann Antoine | Özil Mesut | Neymar |
| Coach | Latvia | Pahars Marians | Cristiano Ronaldo | Messi Lionel | Griezmann Antoine |
| Coach | Lebanon | Radulovic Miodrag | Cristiano Ronaldo | Messi Lionel | Modric Luka |
| Coach | Liberia | Debbah James Salinsa | Cristiano Ronaldo | Ramos Sergio | Messi Lionel |
| Coach | Liechtenstein | Pauritsch René | Cristiano Ronaldo | Neymar | Messi Lionel |
| Coach | Lithuania | Jankauskas Edgaras | Cristiano Ronaldo | Messi Lionel | Bale Gareth |
| Coach | Luxembourg | Holtz Luc | Messi Lionel | Cristiano Ronaldo | Suárez Luis |
| Coach | Macau | Tam Iao San | Cristiano Ronaldo | Messi Lionel | Bale Gareth |
| Coach | Madagascar | Raux Auguste | Messi Lionel | Cristiano Ronaldo | Neymar |
| Coach | Malaysia | Kim Swee Ong | Cristiano Ronaldo | Messi Lionel | Özil Mesut |
| Coach | Maldives | Stewart Darren Raymond | Bale Gareth | Cristiano Ronaldo | Messi Lionel |
| Coach | Malta | Ghedin Pietro | Buffon Gianluigi | Iniesta Andrés | Messi Lionel |
| Coach | Mauritania | Martins Corentin | Cristiano Ronaldo | Messi Lionel | Griezmann Antoine |
| Coach | Mauritius | Tshupula Kande Joe Hubert | Cristiano Ronaldo | Suárez Luis | Ramos Sergio |
| Coach | Mexico | Osorio Juan Carlos | Cristiano Ronaldo | Sánchez Alexis | Messi Lionel |
| Coach | Moldova | Dobrovolskiy Igor | Griezmann Antoine | Lewandowski Robert | Neymar |
| Coach | Mongolia | Zorigt Battulga | Griezmann Antoine | Cristiano Ronaldo | Pogba Paul |
| Coach | Montenegro | Tumbakovic Ljubiša | Messi Lionel | Griezmann Antoine | Neymar |
| Coach | Montserrat | Mendes Junior | Bale Gareth | De Bruyne Kevin | Vardy Jamie |
| Coach | Morocco | Renard Hervé | Neymar | Kanté N'Golo | Cristiano Ronaldo |
| Coach | Mozambique | Xavier Abel | Cristiano Ronaldo | Messi Lionel | De Bruyne Kevin |
| Coach | Myanmar | Zeise Gerd Friedrich Horst | Messi Lionel | Kroos Toni | Lewandowski Robert |
| Coach | Namibia | Mannetti Ricardo | Cristiano Ronaldo | Messi Lionel | Griezmann Antoine |
| Coach | Nepal | Gyotoku Koji | Messi Lionel | Bale Gareth | Pogba Paul |
| Coach | Netherlands | Blind Danny | Cristiano Ronaldo | Kroos Toni | Griezmann Antoine |
| Coach | New Caledonia | Sardo Thierry | Cristiano Ronaldo | Griezmann Antoine | Bale Gareth |

| Vote | Country | Name | First (5 points) | Second (3 points) | Third (1 point) |
| --- | --- | --- | --- | --- | --- |
| Coach | New Zealand | Hudson Anthony | Cristiano Ronaldo | Griezmann Antoine | Payet Dimitri |
| Coach | Nigeria | Rohr Gernot | Griezmann Antoine | Messi Lionel | Neuer Manuel |
| Coach | Northern Ireland | O'Neill Michael | Cristiano Ronaldo | Messi Lionel | Griezmann Antoine |
| Coach | Norway | Høgmo Per-Mathias | Cristiano Ronaldo | Messi Lionel | Neuer Manuel |
| Coach | Oman | Lopez Caro Juan Ramon | Messi Lionel | Iniesta Andrés | Lewandowski Robert |
| Coach | Pakistan | Mehmood Sajjad | Cristiano Ronaldo | Messi Lionel | Neymar |
| Coach | Palestine | Barakat Abdel Nasser | Mahrez Riyad | Cristiano Ronaldo | Messi Lionel |
| Coach | Panama | Gomez Hernan Dario | Messi Lionel | Iniesta Andrés | Lewandowski Robert |
| Coach | Papua New Guinea | Serritslev Flemming | Messi Lionel | Cristiano Ronaldo | Neuer Manuel |
| Coach | Paraguay | Arce Rolón Francisco | Messi Lionel | Cristiano Ronaldo | Iniesta Andrés |
| Coach | Peru | Gareca Nardi Ricardo | Messi Lionel | Suárez Luis | Cristiano Ronaldo |
| Coach | Philippines | Dooley Thomas | Kroos Toni | Neuer Manuel | Cristiano Ronaldo |
| Coach | Poland | Nawalka Adam | Lewandowski Robert | Griezmann Antoine | Neuer Manuel |
| Coach | Portugal | Santos Fernando | Cristiano Ronaldo | Bale Gareth | Griezmann Antoine |
| Coach | Puerto Rico | García Cantarero Carlos | Griezmann Antoine | Bale Gareth | Messi Lionel |
| Coach | Qatar | Fossati Lurachi Jorge Daniel | Mahrez Riyad | Cristiano Ronaldo | Ramos Sergio |
| Coach | Republic of Ireland | O'Neill Martin | Bale Gareth | Buffon Gianluigi | Kanté N'Golo |
| Coach | Romania | Christoph Daum | Kroos Toni | Neuer Manuel | Özil Mesut |
| Coach | Russia | Cherchesov Stanislav | Cristiano Ronaldo | Messi Lionel | Buffon Gianluigi |
| Coach | Rwanda | Jimmy Mulisa | Cristiano Ronaldo | Griezmann Antoine | Mahrez Riyad |
| Coach | Samoa | Easthope Scott | Sánchez Alexis | Pogba Paul | Payet Dimitri |
| Coach | San Marino | Pierangelo Manzaroli | Buffon Gianluigi | Cristiano Ronaldo | Bale Gareth |
| Coach | São Tomé e Príncipe | Rosário António | Cristiano Ronaldo | Messi Lionel | Neymar |
| Coach | Saudi Arabia | Van Marwijk Lambertus | Messi Lionel | Cristiano Ronaldo | Neymar |
| Coach | Scotland | Strachan Gordon | Messi Lionel | Suárez Luis | Agüero Sergio |
| Coach | Senegal | Cisse Aliou | Cristiano Ronaldo | Messi Lionel | Griezmann Antoine |
| Coach | Serbia | Muslin Slavoljub | Messi Lionel | Griezmann Antoine | Kanté N'Golo |
| Coach | Singapore | Moorthy Sundram | Cristiano Ronaldo | Messi Lionel | Bale Gareth |
| Coach | Slovakia | Kozak Jan | Cristiano Ronaldo | Messi Lionel | Bale Gareth |
| Coach | Slovenia | Katanec Srečko | Messi Lionel | Cristiano Ronaldo | Bale Gareth |
| Coach | Somalia | Abdulle Mohamed | Cristiano Ronaldo | Messi Lionel | Mahrez Riyad |
| Coach | Spain | Lopetegui Agote Julen | Messi Lionel | Cristiano Ronaldo | Ramos Sergio |
| Coach | Sri Lanka | Steinwall Dudley Linclon | Messi Lionel | Cristiano Ronaldo | Neymar |

| Vote | Country | Name | First (5 points) | Second (3 points) | Third (1 point) |
| --- | --- | --- | --- | --- | --- |
| Coach | St. Kitts and Nevis | Hazel Jeffrey | Messi Lionel | Cristiano Ronaldo | Neuer Manuel |
| Coach | Sudan | Magzoub Mohamed Abdalla Ahmed | Cristiano Ronaldo | Messi Lionel | Griezmann Antoine |
| Coach | Suriname | Godeken Roberto | Griezmann Antoine | Messi Lionel | Cristiano Ronaldo |
| Coach | Sweden | Andersson Jan | Ibrahimovic Zlatan | Messi Lionel | Cristiano Ronaldo |
| Coach | Switzerland | Petkovic Vladimir | Cristiano Ronaldo | Buffon Gianluigi | Lewandowski Robert |
| Coach | Syria | Al Hakeem Ayman | Messi Lionel | Cristiano Ronaldo | De Bruyne Kevin |
| Coach | Tahiti | Graugnard Ludovic | Messi Lionel | Neymar | Griezmann Antoine |
| Coach | Tajikistan | Fuzaylov Khakim | Cristiano Ronaldo | Griezmann Antoine | Messi Lionel |
| Coach | Tanzania | Mkwasa Charles | Cristiano Ronaldo | Messi Lionel | Kroos Toni |
| Coach | Thailand | Senamuang Kiatisuk | Cristiano Ronaldo | Neymar | Sánchez Alexis |
| Coach | Togo | Le Roy Claude | Cristiano Ronaldo | Griezmann Antoine | Mahrez Riyad |
| Coach | Trinidad and Tobago | Hart Stephen | Cristiano Ronaldo | Messi Lionel | Sánchez Alexis |
| Coach | Turkey | Terim Fatih | Messi Lionel | Modric Luka | Özil Mesut |
| Coach | Turkmenistan | Kochumov Amangylych | Cristiano Ronaldo | Messi Lionel | Griezmann Antoine |
| Coach | Turks and Caicos Islands | Smith Oliver | Vardy Jamie | Cristiano Ronaldo | Messi Lionel |
| Coach | Uganda | Sredojevic Milutin | Cristiano Ronaldo | Messi Lionel | Suárez Luis |
| Coach | Ukraine | Shevchenko Andrii | Cristiano Ronaldo | Messi Lionel | Griezmann Antoine |
| Coach | Uruguay | Oscar Tabarez | Suárez Luis | Messi Lionel | Cristiano Ronaldo |
| Coach | USA | Arena Bruce | Cristiano Ronaldo | Griezmann Antoine | Messi Lionel |
| Coach | Uzbekistan | Babayan Samvel | Messi Lionel | Kroos Toni | Griezmann Antoine |
| Coach | Venezuela | Dudamel Rafael | Messi Lionel | Cristiano Ronaldo | Neymar |
| Coach | Vietnam | Nguyen Huu Thang | Cristiano Ronaldo | Messi Lionel | Griezmann Antoine |
| Coach | Wales | Coleman Christopher | Bale Gareth | Sánchez Alexis | Vardy Jamie |
| Coach | Zambia | Nyirenda Wedson | Messi Lionel | Neymar | Bale Gareth |
| Coach | Zimbabwe | Pasuwa Kalisto | Messi Lionel | Cristiano Ronaldo | Neuer Manuel |
| Media | Albania | Bejzade Dhurata | Messi Lionel | Neuer Manuel | Cristiano Ronaldo |
| Media | Algeria | Saad Mohamed | Mahrez Riyad | Messi Lionel | Griezmann Antoine |
| Media | Andorra | Duaso Victor | Messi Lionel | Suárez Luis | Neymar |
| Media | Angola | Quiala Geraldo | Cristiano Ronaldo | Messi Lionel | Neymar |
| Media | Antigua and Barbuda | Neto Baptiste | Cristiano Ronaldo | Modric Luka | Messi Lionel |
| Media | Argentina | Macaya Márquez Enrique | Messi Lionel | Suárez Luis | Cristiano Ronaldo |
| Media | Armenia | Gasparyan Robert | Cristiano Ronaldo | Buffon Gianluigi | Messi Lionel |
| Media | Aruba | Thomas Kurt | Messi Lionel | Cristiano Ronaldo | Neuer Manuel |

| Vote | Country | Name | First (5 points) | Second (3 points) | Third (1 point) |
| --- | --- | --- | --- | --- | --- |
| Media | Australia | Larkin Steve | Messi Lionel | Neymar | Cristiano Ronaldo |
| Media | Austria | Prohaska Herbert | Cristiano Ronaldo | Messi Lionel | Bale Gareth |
| Media | Bahamas | Smith Randy | Neymar | Cristiano Ronaldo | Agüero Sergio |
| Media | Bahrain | Ashoor Abdullah | Cristiano Ronaldo | Neymar | Mahrez Riyad |
| Media | Bangladesh | Mahamud Raihan | Cristiano Ronaldo | Messi Lionel | Neymar |
| Media | Barbados | Pile Kevin | Cristiano Ronaldo | Vardy Jamie | Neymar |
| Media | Belgium | Terreur Kristof | Cristiano Ronaldo | Messi Lionel | Griezmann Antoine |
| Media | Belize | Cantillano Fredy | Messi Lionel | Neymar | Suárez Luis |
| Media | Brazil | Coelho Paulo Vinicius | Cristiano Ronaldo | Messi Lionel | Neymar |
| Media | Burkina Faso | Hien Victorien | Cristiano Ronaldo | Neymar | Griezmann Antoine |
| Media | Cambodia | Chamroeun Ung | Cristiano Ronaldo | Mahrez Riyad | Griezmann Antoine |
| Media | Cameroon | Samnick Emmanuel Gustave | Cristiano Ronaldo | Buffon Gianluigi | Bale Gareth |
| Media | Canada | Davidson Neil | Cristiano Ronaldo | Messi Lionel | Suárez Luis |
| Media | Cape Verde Islands | Amaral André | Cristiano Ronaldo | Bale Gareth | Pogba Paul |
| Media | Chad | Hissein Atti Alifa | Cristiano Ronaldo | Neymar | Griezmann Antoine |
| Media | Chile | Diaz Danilo | Cristiano Ronaldo | Messi Lionel | Sánchez Alexis |
| Media | Colombia | Rueda Diego | Messi Lionel | Suárez Luis | Neymar |
| Media | Congo DR | Kabulo François | Cristiano Ronaldo | Griezmann Antoine | Messi Lionel |
| Media | Cook Islands | Kumar Rashneel | Cristiano Ronaldo | Messi Lionel | Suárez Luis |
| Media | Costa Rica | Goldberg David | Cristiano Ronaldo | Neymar | Messi Lionel |
| Media | Côte d'Ivoire | Gnae Oulidehi Magloire | Cristiano Ronaldo | Messi Lionel | Griezmann Antoine |
| Media | Cuba | Cancio Yasiel | Mahrez Riyad | Messi Lionel | Cristiano Ronaldo |
| Media | Denmark | Olsen Allan | Cristiano Ronaldo | Griezmann Antoine | Neymar |
| Media | Djibouti | Mohamed Kenadid | Cristiano Ronaldo | Griezmann Antoine | Bale Gareth |
| Media | Dominican Republic | Bauger Jorge Rolando | Cristiano Ronaldo | Messi Lionel | Suárez Luis |
| Media | Ecuador | Rosales Cox Tito | Cristiano Ronaldo | Messi Lionel | Neymar |
| Media | Egypt | Gamil Hany Danial | Cristiano Ronaldo | Mahrez Riyad | Neymar |
| Media | El Salvador | Campos Madrid Gabriel | Cristiano Ronaldo | Suárez Luis | Messi Lionel |
| Media | England | Rumsby Ben | Cristiano Ronaldo | Messi Lionel | Kanté N'Golo |
| Media | Equitorial Guinea | Essiane Fabien | Cristiano Ronaldo | Neymar | Messi Lionel |
| Media | Eritrea | Seium Michael | Cristiano Ronaldo | Sánchez Alexis | Mahrez Riyad |
| Media | Ethiopia | Abdulkeni Hussen | Cristiano Ronaldo | Griezmann Antoine | Suárez Luis |
| Media | Faroe Islands | Olsen Símun Christian | Messi Lionel | Cristiano Ronaldo | Suárez Luis |

| Vote | Country | Name | First (5 points) | Second (3 points) | Third (1 point) |
| --- | --- | --- | --- | --- | --- |
| Media | Fiji | Singh Indra | Cristiano Ronaldo | Messi Lionel | Özil Mesut |
| Media | France | Resseguie Jean | Cristiano Ronaldo | Griezmann Antoine | Messi Lionel |
| Media | FYR Macedonia | Durgutov Mijalce | Buffon Gianluigi | Modric Luka | Messi Lionel |
| Media | Gambia | Camara Baboucar | Cristiano Ronaldo | Mahrez Riyad | Suárez Luis |
| Media | Georgia | Potskhveria Zurab | Cristiano Ronaldo | Griezmann Antoine | Messi Lionel |
| Media | Germany | Neumann Claudia | Cristiano Ronaldo | Griezmann Antoine | Kroos Toni |
| Media | Gibraltar | Ignacio Stephen | Lewandowski Robert | Cristiano Ronaldo | Messi Lionel |
| Media | Greece | Kotsis Nikolaos | Cristiano Ronaldo | Griezmann Antoine | Messi Lionel |
| Media | Grenada | Bascombe Michael | Agüero Sergio | Neymar | De Bruyne Kevin |
| Media | Guatemala | Solares Lucho | Cristiano Ronaldo | Messi Lionel | Sánchez Alexis |
| Media | Guinea-Bissau | Ucha Etiandro | Cristiano Ronaldo | Griezmann Antoine | Messi Lionel |
| Media | Haiti | Leon Lutherson | Cristiano Ronaldo | Griezmann Antoine | Messi Lionel |
| Media | Hungary | Szeli Mátyás | Cristiano Ronaldo | Suárez Luis | Griezmann Antoine |
| Media | Iceland | Sigurdsson Vidir | Messi Lionel | Cristiano Ronaldo | Suárez Luis |
| Media | India | Sarkar Dhiman | Cristiano Ronaldo | Messi Lionel | Neymar |
| Media | Indonesia | Widakdo Gatot | Bale Gareth | Cristiano Ronaldo | Griezmann Antoine |
| Media | Iran | Chia Foadi | Cristiano Ronaldo | Messi Lionel | Griezmann Antoine |
| Media | Israel | Dagon Uri | Messi Lionel | Cristiano Ronaldo | Suárez Luis |
| Media | Italy | Licari Fabio | Cristiano Ronaldo | Griezmann Antoine | Neymar |
| Media | Japan | Tsuchiya Kentaro | Cristiano Ronaldo | Griezmann Antoine | Bale Gareth |
| Media | Kazakhstan | Tulegenov Geniy | Cristiano Ronaldo | Griezmann Antoine | Messi Lionel |
| Media | Kenya | Toskin Robin | Cristiano Ronaldo | Mahrez Riyad | Messi Lionel |
| Media | Korea Republic | Yoo Jeeho | Cristiano Ronaldo | Neymar | Messi Lionel |
| Media | Kosovo | Konuschevci Valon | Cristiano Ronaldo | Messi Lionel | Griezmann Antoine |
| Media | Kuwait | Adnad Yousif | Cristiano Ronaldo | Messi Lionel | Suárez Luis |
| Media | Latvia | Koscinkevic Ilvars | Messi Lionel | Cristiano Ronaldo | Griezmann Antoine |
| Media | Lesotho | Kabi Pascalina | Pogba Paul | Neymar | Messi Lionel |
| Media | Liechtenstein | Hasler Ernst | Griezmann Antoine | Bale Gareth | Cristiano Ronaldo |
| Media | Lithuania | Mindaugas Augustis | Cristiano Ronaldo | Messi Lionel | Bale Gareth |
| Media | Luxembourg | Lahure Petz Antoine | Cristiano Ronaldo | Messi Lionel | Suárez Luis |
| Media | Macau | Maia Pedro | Cristiano Ronaldo | Griezmann Antoine | Messi Lionel |
| Media | Malawi | Kanjere Peter | Messi Lionel | Cristiano Ronaldo | Griezmann Antoine |
| Media | Maldives | Ali Shimaz | Cristiano Ronaldo | Messi Lionel | Vardy Jamie |

| Vote | Country | Name | First (5 points) | Second (3 points) | Third (1 point) |
| --- | --- | --- | --- | --- | --- |
| Media | Mali | Diakite Boubacar | Cristiano Ronaldo | Lewandowski Robert | Griezmann Antoine |
| Media | Malta | Moore Jesmond | Cristiano Ronaldo | Messi Lionel | Suárez Luis |
| Media | Mauritania | Brahim Sow | Griezmann Antoine | Cristiano Ronaldo | Mahrez Riyad |
| Media | Mauritius | Azmaal Hydoo | Cristiano Ronaldo | Griezmann Antoine | Messi Lionel |
| Media | Mexico | Blancas Avalos Ricardo Amado | Suárez Luis | Cristiano Ronaldo | Griezmann Antoine |
| Media | Moldova | Goihman Miron | Griezmann Antoine | Cristiano Ronaldo | Bale Gareth |
| Media | Nepal | Rijal Ritesh | Messi Lionel | Cristiano Ronaldo | Bale Gareth |
| Media | Netherlands | De Groot Jaap | Cristiano Ronaldo | Neymar | Griezmann Antoine |
| Media | New Caledonia | Cambefort Yoann | Griezmann Antoine | Payet Dimitri | Cristiano Ronaldo |
| Media | New Zealand | Burgess Michael | Griezmann Antoine | Bale Gareth | Mahrez Riyad |
| Media | Niger | Ganoua Mohamed Silimane | Cristiano Ronaldo | Messi Lionel | Griezmann Antoine |
| Media | Oman | Purohit Ashok | Cristiano Ronaldo | Messi Lionel | Vardy Jamie |
| Media | Paraguay | Cazenave Gabriel | Griezmann Antoine | Messi Lionel | Cristiano Ronaldo |
| Media | Peru | Canelo Quispe Pedro Eduardo | Messi Lionel | Cristiano Ronaldo | Griezmann Antoine |
| Media | Philippines | Fenix Ryan | Cristiano Ronaldo | Messi Lionel | Griezmann Antoine |
| Media | Poland | Pol Michal | Cristiano Ronaldo | Messi Lionel | Lewandowski Robert |
| Media | Portugal | Serpa Vitor | Cristiano Ronaldo | Griezmann Antoine | Messi Lionel |
| Media | Puerto Rico | Pagan Esteban | Messi Lionel | Cristiano Ronaldo | Griezmann Antoine |
| Media | Qatar | Anil John | Messi Lionel | Cristiano Ronaldo | Neymar |
| Media | Republic of Ireland | Quinn Philip | Cristiano Ronaldo | Messi Lionel | Neymar |
| Media | Romania | Gafiuc Justin Paul | Cristiano Ronaldo | Messi Lionel | Bale Gareth |
| Media | Russia | Stognienko Vladimir | Cristiano Ronaldo | Bale Gareth | Buffon Gianluigi |
| Media | San Marino | Gorini Elia | Buffon Gianluigi | Cristiano Ronaldo | Bale Gareth |
| Media | São Tomé e Príncipe | Barros Manuel | Messi Lionel | Neymar | Iniesta Andrés |
| Media | Saudi Arabia | Al Muhawas Sultan | Messi Lionel | Neymar | Özil Mesut |
| Media | Scotland | Castles Duncan | Cristiano Ronaldo | Suárez Luis | Neuer Manuel |
| Media | Senegal | Ndiaye Babacar Dit Khalifa | Cristiano Ronaldo | Messi Lionel | Mahrez Riyad |
| Media | Serbia | Rasevic Milan | Cristiano Ronaldo | Messi Lionel | Sánchez Alexis |
| Media | Sierra Leone | Sahr Morris Jr | Cristiano Ronaldo | Griezmann Antoine | Mahrez Riyad |
| Media | Slovakia | Zeman Michal | Cristiano Ronaldo | Messi Lionel | Griezmann Antoine |
| Media | Slovenia | Sabadin Denis | Cristiano Ronaldo | Neuer Manuel | Pogba Paul |
| Media | Somalia | Guurre Gamadiid | Messi Lionel | Neymar | Suárez Luis |
| Media | South Africa | Marawa Robert | Cristiano Ronaldo | Suárez Luis | Messi Lionel |

| Vote | Country | Name | First (5 points) | Second (3 points) | Third (1 point) |
| --- | --- | --- | --- | --- | --- |
| Media | Spain | Torres Diego | Messi Lionel | Griezmann Antoine | Iniesta Andrés |
| Media | St. Lucia | Finisterre Terry | Cristiano Ronaldo | Messi Lionel | Sánchez Alexis |
| Media | Sudan | Awadalla Mohammed Al-Khatim | Cristiano Ronaldo | Mahrez Riyad | Griezmann Antoine |
| Media | Suriname | Romeo Desney | Cristiano Ronaldo | Bale Gareth | Messi Lionel |
| Media | Swaziland | Dlamini Kenneth | Griezmann Antoine | Neymar | Bale Gareth |
| Media | Sweden | Bank Simon | Cristiano Ronaldo | Messi Lionel | Griezmann Antoine |
| Media | Switzerland | Clalüna Flurin | Griezmann Antoine | Cristiano Ronaldo | Bale Gareth |
| Media | Syria | Abou Shameh Louay | Cristiano Ronaldo | Griezmann Antoine | Bale Gareth |
| Media | Tajikistan | Soliev Faridun | Cristiano Ronaldo | Messi Lionel | Griezmann Antoine |
| Media | Thailand | Chittinand Tor | Messi Lionel | Bale Gareth | Griezmann Antoine |
| Media | Togo | Ayena Mathias | Cristiano Ronaldo | Bale Gareth | Payet Dimitri |
| Media | Turkey | Arslan Mehmet | Cristiano Ronaldo | De Bruyne Kevin | Buffon Gianluigi |
| Media | Turkmenistan | Vershinin Alexander | Messi Lionel | Suárez Luis | Neymar |
| Media | Turks and Caicos Islands | Deo Faizool | Neymar | Cristiano Ronaldo | Messi Lionel |
| Media | Uganda | Kaweesi Fred | Cristiano Ronaldo | Griezmann Antoine | Kanté N'Golo |
| Media | Uruguay | Bernardoni Muller Didier | Suárez Luis | Griezmann Antoine | Iniesta Andrés |
| Media | USA | Bell Arch | Suárez Luis | Bale Gareth | Messi Lionel |
| Media | Vanuatu | Nase Raymond | Vardy Jamie | Cristiano Ronaldo | Neuer Manuel |
| Media | Venezuela | Perozo Suárez Humberto | Messi Lionel | Suárez Luis | Neymar |
| Media | Vietnam | Nguyen Minh Viet | Cristiano Ronaldo | Griezmann Antoine | Messi Lionel |
| Media | Wales | Abbandonato Paul | Bale Gareth | Cristiano Ronaldo | Griezmann Antoine |
| Media | Zambia | Chipepo Elias | Cristiano Ronaldo | Messi Lionel | Suárez Luis |
| Media | Zimbabwe | Locadia Sabeta Chip | Cristiano Ronaldo | Messi Lionel | Kanté N'Golo |
